# Safety of COVID-19 vaccines during pregnancy: A systematic review and meta-analysis

**DOI:** 10.1016/j.vaccine.2023.03.038

**Published:** 2023-06-07

**Authors:** Agustín Ciapponi, Mabel Berrueta, Edward P.K. Parker, Ariel Bardach, Agustina Mazzoni, Steven A. Anderson, Fernando J. Argento, Jamile Ballivian, Karin Bok, Daniel Comandé, Erin Goucher, Beate Kampmann, Flor M. Munoz, Federico Rodriguez Cairoli, Victoria Santa María, Andy S. Stergachis, Gerald Voss, Xu Xiong, Natalia Zamora, Sabra Zaraa, Pierre M Buekens

**Affiliations:** a Centro de Investigación de Epidemiología y Salud Pública (CIESP) - Instituto de Efectividad Clínica y Sanitaria (IECS-CONICET), Argentina; b The Vaccine Centre, London School of Hygiene and Tropical Medicine, London WC1E 7HT, UK; c US Food & Drug Administration, 10903 New Hampshire Avenue, Silver Spring, MD 20993, USA; d National Institute of Allergy and Infectious Diseases, National Institutes of Health, 31 Center Dr # 7A03, Bethesda, MD 20892, USA; e School of Public Health and Tropical Medicine, Tulane University, New Orleans, USA; f Vaccines & Immunity Theme, MRC Unit The Gambia at the London School of Hygiene and Tropical Medicine, Banjul, the Gambia; g Charité Centre for Global Health, Universitätsmedizin Charité Berlin, Germany; h Baylor College of Medicine, Texas Children's Hospital, 6621 Fannin St, Houston, TX 77030, USA; i School of Pharmacy and School of Public Health, University of Washington, 1959 NE Pacific St, BOX 357631, Seattle, WA, USA; j Coalition for Epidemic Preparedness Innovations (CEPI), Oslo, Norway

**Keywords:** Pregnancy, COVID-19, Vaccine safety, Adjuvant, Systematic review, Meta-analysis

## Abstract

**Background:**

Assessment of COVID-19 vaccines safety during pregnancy is urgently needed.

**Methods:**

We conducted a systematic review and *meta*-analysis to evaluate the safety of COVID-19 vaccines, including their components and technological platforms used in other vaccines during pregnancy and animal studies to complement direct evidence. We searched literature databases from its inception to September 2021 without language restriction, COVID-19 vaccine websites, and reference lists of other systematic reviews and the included studies. Pairs of reviewers independently selected, data extracted, and assessed the risk of bias of the studies. Discrepancies were resolved by consensus. (PROSPERO CRD42021234185).

**Results:**

We retrieved 8,837 records from the literature search; 71 studies were included, involving 17,719,495 pregnant persons and 389 pregnant animals. Most studies (94%) were conducted in high-income countries, were cohort studies (51%), and 15% were classified as high risk of bias. We identified nine COVID-19 vaccine studies, seven involving 309,164 pregnant persons, mostly exposed to mRNA vaccines. Among non-COVID-19 vaccines, the most frequent exposures were AS03 and aluminum-based adjuvants.

A *meta*-analysis of studies that adjusted for potential confounders showed no association with adverse outcomes, regardless of the vaccine or the trimester of vaccination.

Neither the reported rates of adverse pregnancy outcomes nor reactogenicity exceeded expected background rates, which was the case for ASO3- or aluminum-adjuvanted non-COVID-19 vaccines in the proportion *meta*-analyses of uncontrolled studies/arms. The only exception was postpartum hemorrhage after COVID-19 vaccination (10.40%; 95% CI: 6.49–15.10%), reported by two studies; however, the comparison with non-exposed pregnant persons, available for one study, found non-statistically significant differences (adjusted OR 1.09; 95% CI 0.56–2.12). Animal studies showed consistent results with studies in pregnant persons.

**Conclusion:**

We found no safety concerns for currently administered COVID-19 vaccines during pregnancy. Additional experimental and real-world evidence could enhance vaccination coverage. Robust safety data for non-mRNA-based COVID-19 vaccines are still needed.

## Highlights

• COVID-19 during pregnancy imposes a risk of severe disease and adverse birth outcomes.
• Data about the safety of vaccination against COVID-19 for pregnant women is limited.
• COVID-19 vaccination coverage among pregnant persons is suboptimal.
• We found no safety concerns for COVID-19 vaccination during pregnancy.
• Our findings support authorized or approved COVID-19 vaccines for pregnant persons.

## 1 Background

The unique physiological changes that occur in the immune and cardiopulmonary systems during pregnancy place pregnant persons at a higher risk of severe outcomes if infected with viral respiratory pathogens.[1], [2], [3], [4], [5] Reviews addressing pregnancy and COVID-19 report that intensive care unit (ICU) admission rates and requirements for invasive ventilation in this population are higher than in nonpregnant persons.[1] Pregnant persons with pre-existing comorbidities, obesity, or advanced maternal age are at an even higher risk of severe COVID-19.[6], [7] Although initial phase 1–3 studies of COVID-19 vaccines excluded pregnant persons, more recently, efficacy trials of COVID-19 vaccines considered including pregnant persons[8], and only limited human data on COVID-19 vaccine safety when used during pregnancy were available for individual products at the time of emergency use authorization.[6] A survey conducted in 16 countries[9] reported that one of the top three reasons for pregnant persons declining COVID-19 vaccination, even if the vaccine was safe and freely available, was that they did not want to expose their developing baby to any possible harmful side effects.

As of the start of 2023, 10 vaccines have received an Emergency Use Listing from the WHO. Despite limited available safety data, many countries recommend COVID-19 vaccination in pregnant persons [10], [11], [12] on the basis that the benefits of vaccination are likely to outweigh the theoretical risks. Consequently, it is imperative to identify and evaluate safety signals for COVID-19 vaccines as early as possible. Given the urgency of the issue for current public health practice globally, we performed a rapid review and interim analysis of the components and platforms of selected vaccines for the COVID-19 Vaccines Global Access-Maternal Immunization Working Group (COVAX-MIWG) up to August 2020 and found no safety concerns (albeit predominantly based on indirect evidence from COVID-19 components and platforms used in other vaccines).[13] Some studies reported that low income and low education are associated with vaccine hesitancy during pregnancy.[14] More high-quality safety and surveillance studies plus clear communication to pregnant persons and health providers are needed and the results of this systematic review can support informed communication.To assist pregnant persons and their health care providers in making informed decisions, we now report a systematic review and *meta*-analysis of adverse events and pregnancy outcomes associated with COVID-19 vaccination in pregnancy. Our primary objective considered authorized COVID-19 vaccines in pregnant persons. As a secondary objective, we considered COVID-19 vaccine components and platforms used in other maternal vaccines, including animal studies, in order to address direct evidence gaps. A living systematic review and *meta*-analysis of Covid-19 vaccines for pregnant persons, by our author group, can be found in its Internet link (https://safeinpregnancy.org/lsr/).

### 1.1 Objectives

To study the association of COVID-19 vaccines, or their components/platforms used in other vaccines, with vaccine-related adverse events, including reactogenicity, and adverse obstetric and neonatal outcomes.

## 2 Methods

For this systematic review and *meta*-analysis, we followed the Cochrane methods[15], [16] and the 2020 Preferred Reporting Items for Systematic Reviews and Meta-Analyses (PRISMA) statement[17] for reporting results (see **Appendix A, Supplementary 1** PRISMA checklist). This review was registered in PROSPERO (CRD42021234185).

### 2.1 Inclusion criteria

We included comparative and non-comparative study designs in pregnant persons. Case-series were only included if they reported>50 exposed pregnant persons. We also included experimental studies of any sample size with exposed pregnant animals. We excluded systematic reviews (SRs) and narrative reviews but explored their reference lists as an additional primary study source. Passive surveillance studies with no clear denominator and articles with unavailable full text were also excluded from the review. No language or geographic restrictions were applied.

**Fig. 1:**
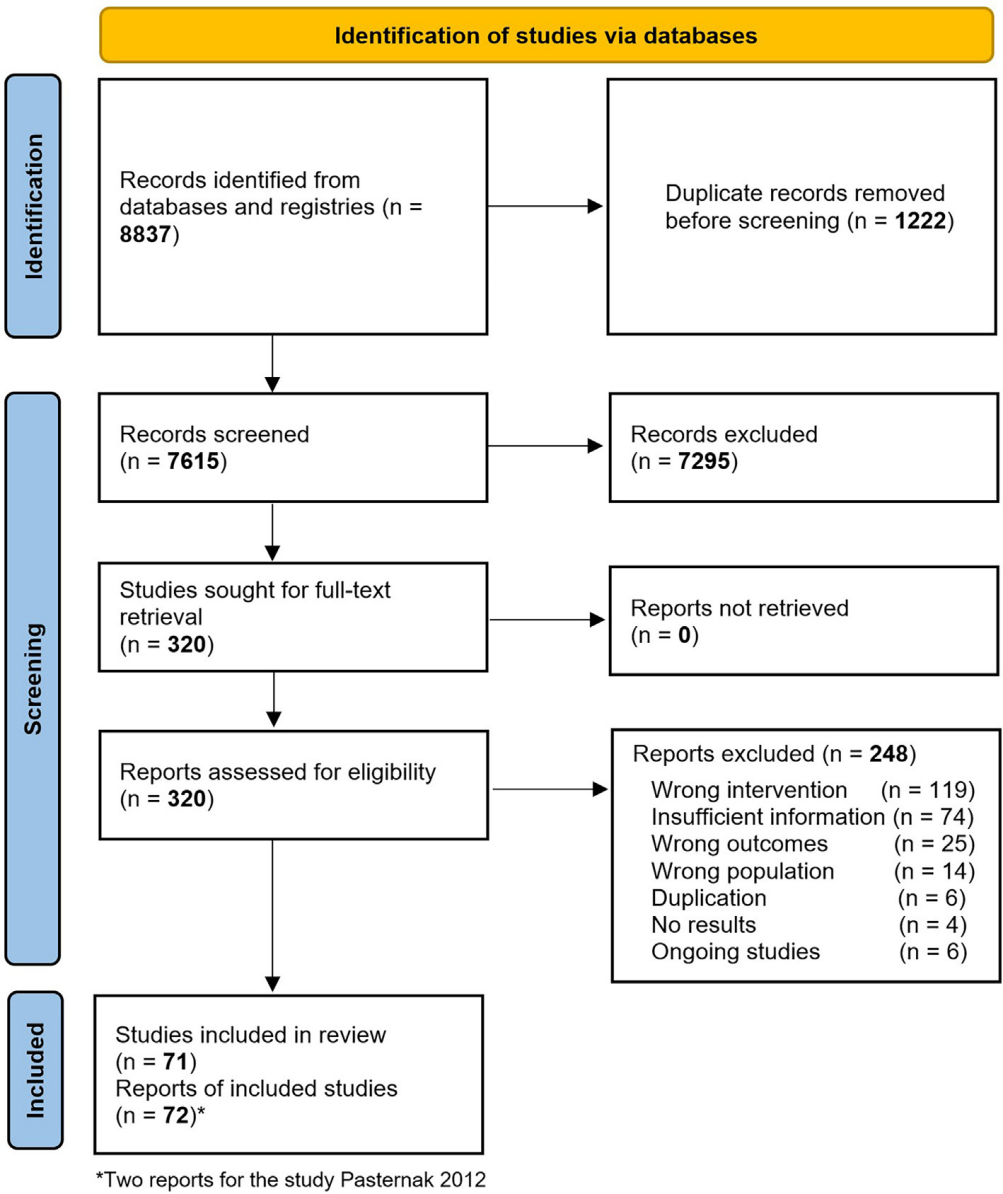
Study flow diagram.

The primary exposures or interventions of interest were vaccination with a COVID-19 vaccine authorized by the World Health Organization (WHO) and/or authorized or approved by any national regulatory authority up to December 2021. As a secondary objective, we considered exposure to other vaccines used during pregnancy that use the platforms (protein/subunit, vectored, nucleic acid/mRNA-LNP) or components (antigen, vehicle, construct, adjuvants, lipid nanoparticles, etc.) used by COVID-19 vaccines in use or late-stage development, including AS03 (used in the CoV2 preS dTM-AS03 vaccine [Sanofi Pasteur]), aluminum (used in multiple vaccines including CoronaVac [Sinovac] and Covaxin [Bharat Biotech]), CpG 1018 and Matrix-M (see **Appendix A, Supplementary** Table 2 for full details).[18] These components can be found in influenza vaccines (including Arepanrix, Pandemrix, FLUAD), Tdap, and hepatitis A and B vaccines (Havrix, Engerix-B, Twinrix, Recombivax, etc.). The study was included if at least one of these exposures was explicitly described in the report. This approach, illustrated in Fig. 1 of the **Appendix A, Supplementary 1**, is aimed to provide the most complete direct and indirect evidence to assist health decision-making.

Primary studies using experimental and observational epidemiological study designs were deemed eligible for inclusion if adverse obstetric or neonatal outcomes (e.g., miscarriage, stillbirth, preterm birth, low birth weight, neonatal death, birth defects, congenital infections) were assessed in pregnant persons exposed to any of the defined exposures during pregnancy and compared to pregnant persons with no such exposure. Perinatal outcomes were based on 25 standardized case definitions developed by the Global Alignment of Immunization Safety Assessment in Pregnancy (GAIA) of prioritized obstetric and neonatal outcomes.[19] We considered outcomes concerning exposure to the vaccines based on the reported gestational age at vaccination (based on validated methods including ultrasound or last menstrual period [LMP] for human studies).

Other vaccine-related adverse events (AEs) and serious adverse events unrelated to pregnancy were also assessed. An AE was defined as any untoward medical occurrence in a patient or clinical investigation subject administered a pharmaceutical product regardless of its causal relationship to the study treatment[20]. Injection site reactions, systemic reactions, medically attended adverse events (MAEs), adverse events of special interest (AESIs), anaphylaxis, and other post-vaccination medical events were also assessed. The operative definition of each specific AE has been reported elsewhere (PROSPERO- CRD42021234185).

### 2.2 Search strategy

We searched published and unpublished studies, without restrictions on language or publication status, from inception date to September 15, 2021 (see **Appendix A, Supplementary Methods** for search terms) in the Cochrane Library databases (from 1996), MEDLINE (from 1946), EMBASE (from 1974), Latin American and Caribbean Health Sciences Literature (LILACS; (from 1982), Science Citation Index Expanded (SCI-EXPANDED; from 19964), Global Health (from 1910), China Network Knowledge Information (CNKI; from 2015), WHO Database of publications on SARS CoV2 (from 2020), TOXLine (from 1972), preprint servers (ArXiv, biorXiv, medRxiv, search.bioPreprint), and COVID-19 research websites (PregCOV-19LSR, Maternal and Child Health, Nutrition: John Hopkins Centre for Humanitarian health, the LOVE database). The reference lists of relevant primary studies in systematic reviews retrieved by the search strategy and active COVID-19 pregnancy registries with data on vaccination during pregnancy were assessed to capture reported adverse events/safety data. The US FDA, the European Medicines Agency (EMA), and clinical trials websites were also searched. Additionally, we contacted authors and experts in the field to obtain extra information, as necessary.

## 3 Selection of studies, data extraction, and assessment of the risk of bias

Pairs of authors independently screened each identified record by title and abstract and retrieved full texts of the potentially eligible studies. Pairs of review authors independently examined the full‐text articles for compliance with the inclusion criteria. We resolved any disagreements by discussion. The selection process was conducted through COVIDENCE[21], a software for systematic reviews.[17] If studies had multiple publications, we collated the various reports of the same study under a single study ID with multiple references.

Pairs of review authors independently extracted data from eligible studies using a data extraction form designed and pilot‐tested by the authors. Any disagreements were resolved by discussion. Extracted data included study methods, population, exposures, comparisons and outcome data. We also extracted the risk of bias items for each study type described in the **Appendix A, Supplementary Methods**. We independently assessed the risk of bias of the included clinical trials using the Cochrane risk of bias assessment tool for Randomized Controlled Trials (RCT).[22] We used the Cochrane EPOC group tools[23] to assess controlled before‐after studies (CBAs), nationwide uncontrolled before‐after studies (UBAs), interrupted time series (ITSs), and controlled-ITSs (CITSs). We rated the risk of bias in each domain as “low”, “high”, or “unclear”. For observational cohort, case-control, cross-sectional, and case-series studies we used the NIH Quality Assessment Tool.[24] After answering the different signaling questions “Yes”, “No”, “Cannot determine”, “Not applicable”, or “Not reported”, the raters classified the study quality as “good”, “fair”, or “poor”. For consistency with the other designs, we use the classifications “low”, “unclear” or “high” risk of bias, respectively. Full assessment criteria for each study type are provided in the **Appendix A, Supplementary Methods**.

### 3.1 Data synthesis

Data were collected from RCTs, observational, comparative, and non-comparative studies, including registries. We conducted a paired *meta*-analysis for outcomes for which studies were deemed comparable. Given the potentially different effects on pregnancy outcomes, we conducted a subgroup analyses by the trimester of exposure and by adjuvant/component (for non-COVID-19 maternal vaccines). For maternal vaccines containing AS03 or aluminum, we *meta*-analyzed outcomes compared with no-exposure or exposure to other vaccines without these adjuvants. Sensitivity analyses were conducted, excluding studies with high risk of bias.

We described the effect estimates as reported by the authors of the included studies. For dichotomous data, we used the numbers of events in the control and intervention groups of each study to calculate risk ratios (RRs), hazard ratios (HRs), or Mantel‐Haenszel odds ratios (ORs). We applied the generic inverse variance method in RevMan 5.4 and DerSimonian-Laird weights in random effects models.[25], [26] For each study included in the *meta*-analyses, we opted to use adjusted effect measures (e.g. by age, smoking status, parity, body mass index, etc.) over unadjusted estimates where possible. We summarized the GRADE certainty of evidence from comparative studies in Summary of findings' tables[15], [27] – estimates were downgraded for serious and very serious imprecision if 95% confidence intervals (CIs) crossed the null effect and the limits were < 0.95 and/or > 1.05, and < 0.5 and/or > 1.50, respectively; and for serious and very serious inconsistency if I^2^ values were > 60% and > 75%, respectively.

To estimate the prevalence of the adverse events and pregnancy-related outcomes across the included studies, we conducted a proportional *meta*-analysis using the StatsDirect statistical software that applies an arcsine transformation to stabilize variance in proportions[28]. These data were analyzed in the context of background rates for neonatal and obstetric outcomes.

### 3.2 Results

We retrieved 8,837 records, of which 320 were eligible for full-text review. A total of 248 studies were excluded, mainly because they did not meet the inclusion criteria of exposure or intervention (1 1 9) or insufficient information (74). We included 71 clinical and preclinical studies (72 reports), 61 involving pregnant persons (N = 17,719,495)[29], [30], [31], [32], [33], [34], [35], [36], [37], [38], [39], [40], [41], [42], [43], [44], [45], [46], [47], [48], [49], [50], [51], [52], [53], [54], [55], [56], [57], [58], [59], [60], [61], [62], [63], [64], [65], [66], [67], [68], [69], [70], [71], [72], [73], [74], [75], [76], [77], [78], [79], [80], [81], [82], [83], [84], [85], [86], [87], [88], [89], [90], and 10 involving pregnant animals (N = 389)[91], [92], [93], [94], [95], [96], [97], [98], [99], [100] (The framework for evidence presentation is illustrated in **Appendix A,** Supplementary Fig. 1). The list of excluded studies and the reasons for exclusion are presented in **Appendix A, Supplementary** Table 3.

## 4 Description of studies

The characteristics of included studies are described in Table 1 (for COVID-19 vaccines) and **Appendix A,** Supplementary Table 4 (for non-COVID-19 vaccines). The most frequent study design was cohort studies (n = 37), followed by RCTs (n = 13), surveillance studies (n = 12), registry analyses (n = 7), cross-sectional study (n = 1), and non-RCT (n = 1). All studies reported a follow-up until delivery, but eight reported a mean follow-up of two months beyond delivery. Twenty-one (30%) of the included studies reported a relative measure effect comparing vaccinated and unvaccinated pregnant persons.[32], [33], [38], [39], [45], [46], [48], [50], [52], [53], [54], [60], [61], [66], [67], [76], [79], [80], [81], [82], [83] Ten out of the 71 studies (14%) were abstracts[41], [47], [51], [55], [56], [58], [73], [79], [80], [82], and ten were conducted on animals (14%).[91], [92], [93], [94], [95], [96], [97], [98], [99], [100] Only six out of 71 studies (8%) involved low- and middle-income countries (LMICs).

**Table 1:** Characteristic of included studies for COVID-19 vaccines.

ID	Country	Year	Type of surveillance	Trimes- ter	Vaccine	Population	N (Ex/No Ex)	Study design	Effect measures	Outcomes	Safety concerns
Pregnant persons											
Bookstein 2021[31]	Israel	2021	Active	1 + 2 + 3	BNT162b2	Pregnant p	390	Surveillance	IR	Mat, Neo, AEFI	No
Zauche 2021[85]	USA	2020–21	Active	1 + 2	BNT162b2, mRNA-1273	Pregnant p	2,456	Registry	IR	Mat.	No
Goldshtein 2021[40]	Israel	2020–21	Active	1 + 2 + 3	BNT162b2	Pregnant p	15,060 (7,530/7,530)	Cohort study	IR	Mat, Neo	No
Gray 2021[41]	USA	2021	Active	1 + 2 + 3	BNT162b2, mRNA-1273	Pregnant p	84	Cohort study	IR	Mat, Neo, AEFI	No
Kharbanda 2021[49]	USA	2020	Active	1 + 2	BNT162b2, mRNA-1273, Ad26.COV2.S	Pregnant p	271,083 (20,139/250,944)	Surveillance	IR, aOR	Mat.	No
Shimabukuro 2021[82]	USA	2020–21	Passive	1 + 2 + 3	BNT162b2, mRNA-1273	Pregnant p	19,252 (16,522/2,730)	Registry	IR	Mat, Neo, AEFI	No
Blakeway 2021[87]	UK	2021	Active	1 + 2	BNT162b2, mRNA-1273, ChAdOx1	Pregnant p	532 (133/399)	Cohort study	IR, aOR	Mat, Neo	No
Pregnant animals											
Stebbings 2021[94]	UK	2020	NA	NA	ChAdOx1	Animals	50 (25/25)	Non-RCT	IR	Mat, Neo.	No
Bowman 2021[89]	USA	2019-	NA	NA	BNT162b2	Animals	88 (44/44)	RCT	IR	Neo.	No

**NA**: Not available; **Pregnant p**: Pregnant person; **IR**: Incidence rate; **aOR**: adjusted Odds Ratio; **Mat**: Maternal; **Neo**: Neonatal; **AEFI**: Adverse Event Following Immunization.

We identified nine COVID-19 vaccine studies, of which seven were conducted in pregnant persons and two were conducted in pregnant animals, with total population sizes of 309,164 and 133, respectively (Table 1). The seven studies in pregnant persons were conducted in the USA (n = 4), Israel (n = 2), and the UK (n = 1), and reported on exposures concerning any of the three trimesters of pregnancy (n = 4) or the first and second trimester (n = 3). Five studies reported exclusively on exposure to mRNA-containing lipid nanoparticle (LNP) vaccines (BNT162b2 and mRNA-1273);[34], [43], [44], [85], [88] and two reported on multiple platforms (mRNA-LNP vaccines alongside the vectored vaccines Ad26.COV2-S and ChAdOx1 nCoV-19).[52], [90] However, Kharbanda et al. excluded Ad26.COV2-S given that it was received only by 308 (0.2%) of pregnant persons in the study population.[52] Only 13 pregnant persons were exposed to ChAdOx1 nCoV-19), and we did not find evidence for other COVID-19 vaccines authorized by WHO or individual countries (e.g., Novavax, Sinopharm, Ad5-nCOV, or Sputnik).

A summary of the 62 studies reporting on COVID-19 vaccine components used in other vaccines is provided in **Appendix A,** Supplementary Table 4. The most frequent exposures were to the AS03 adjuvant (23 studies involving 13,846,993 pregnant persons) and aluminum-based adjuvants (31 clinical and involving 3,499,809 pregnant persons and five preclinical studies involving 214 pregnant animals).

### 4.1 Risk of bias in included studies

The risk of bias for the included clinical studies of COVID-19 vaccines in pregnant persons by study design is presented in **Appendix A,** Supplementary Table 5.1. After considering the responses to the 14 signaling questions, of the seven observational studies, four were rated as being of “good' overall quality, while three were rated as ”fair“. The risk of bias for the included studies of non-COVID-19 vaccines in pregnant persons is presented in **Appendix A,** Supplementary Table 6.1 (for observational studies) and **6.2** (for clinical trials). Out of the 57 non-COVID-19 vaccine observational studies, 19 were rated as being of ”good' overall quality, 31 as “fair” and seven as “poor” quality.

### 4.2 Controlled studies of COVID-19 vaccines in pregnant persons

**Fig. 2:**
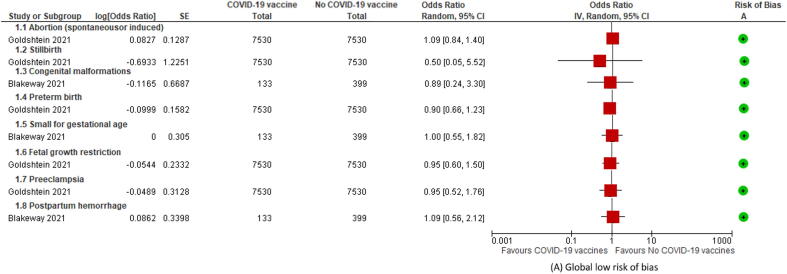
Forest plots of pregnancy outcomes comparing exposure with no exposure to COVID-19 vaccines.

Two out of seven studies compared exposure to COVID-19 vaccines with no exposure in pregnant persons and attempted to control for potential confounders.[43], [90] Blakeway et al. compared mostly mRNA-LNP vaccines (120/133; 90%) during the second and third trimester and used propensity scores calculated from the index of multiple deprivation quintile, self-reported ethnicity, antenatal medication, pregestational diabetes mellitus, maternal age, and antihypertensive medication.[90] This study reported aORs for congenital malformations (0.89; 95% CI 0.24–3.31), small for gestational age (1.00; 95% CI 0.55–1.82), and postpartum hemorrhage (PPH; 1.09; 95% CI 0.56–2.12) in exposed versus unexposed pregnant persons. The second study conducted by Goldshtein et al. compared pregnant persons receiving BNT162b2 with a matched population by age, gestational age, residential area, population subgroup, number of prior children, and having seasonal influenza vaccine.[43] The ORs were calculated for five pregnancy outcomes: spontaneous or induced abortion (1.09; 95% CI 0.84–1.40), stillbirth (0.50; 95% CI 0.05–5.51), preterm birth (0.90; 95% CI 0.66–1.23), fetal growth restriction (0.95; 95% CI 0.60–1.50), and pre-eclampsia (0.95; 95% CI 0.52–1.76). None of the estimates reported by either study (see Table 2 and Fig. 2) showed statistically nor clinically significant association with adverse outcomes, but the certainty of evidence, according to the GRADE approach, was classified as “very low”.

**Table 2:** Meta-analysis and certainty of evidence for controlled studies involving COVID-19 vaccines in pregnant persons.

Outcome	Trimester Ex.	aOR (95% CI)	# Studies (Ex vs. no-Ex)	I^2^	GRADE
Abortion, spontaneous or induced^mOR^[40]	All	1.09 (0.84, 1.40)	1 (7530 vs 7530)	NA	Very low^1^
Stillbirth^mOR^[40]	All	0.50 (0.05, 5.51)	1 (7530 vs 7530)	NA	Very low^1^
Congenital malformations[87]	2nd/3rd	0.89 (0.24 to 3.31)	1 (133 vs 399)	NA	Very low^1^
Preterm birth^mOR^[40]	All	0.90 (0.66, 1.23)	1 (7530 vs 7530)	NA	Very low^1^
Small for gestational age[87]	2nd/3rd	1.00 (0.55 to 1.82)	1 (133 vs 399)	NA	Very low^1^
Fetal growth restriction^mOR^[40]	All	0.95 (0.60, 1.50)	1 (7530 vs 7530)	NA	Very low^1^
Pre-eclampsia^mOR^[40]	All	0.95 (0.52, 1.76)	1 (7530 vs 7530)	NA	Very low^1^
Postpartum hemorrhage[87]	2nd/3rd	1.09 (0.56 to 2.12)	1 (133 vs 399)	NA	Very low^1^

**aOR**: adjusted Odds Ratio; **mOR**: OR calculated from the matched study population by age, gestational age, residential area, population subgroup, number of prior children, and having a seasonal influenza vaccine (Goldshtein 2021).

in the last year; **CI**: Confidence interval; **NA**; Not applicable; **Ex**: Exposure; **1st**: first; **2nd**: second; **3rd**: third. 1. **GRADE** certainty of evidence: started “Low” due to the observational designs, and was downgraded two levels due to very serious imprecision (crossing the null effect, and 95%CI < 0.5 and/or > 1.50.

Kharbanda et al. reported raw data on spontaneous abortions between vaccinated pregnant persons with BNT162b2 (1,128 / 20,139: 8.6%) and non-vaccinated ones (13,160 / 250,944: 8.0%).[52] Bookstein et al.[34] and Gray et al.[44] compared pregnant with nonpregnant persons. Bookstein et al.[34] found similar or lower rates of rash, fever, severe fatigue, myalgia, arthralgia, headache, local pain, swelling, and axillary lymphadenopathy following vaccination in pregnant persons than nonpregnant persons. After the second dose, paresthesia was significantly more common among pregnant persons than the nonpregnant population. There were no important differences in the rates of side effects regardless of the trimester of pregnancy, except for uterine contractions, which were significantly more common after the second dose when administered during the third trimester than during the other trimesters. No differences were noted in reactogenicity across pregnant, lactating, and nonpregnant groups by Gray et al.[44].

### 4.3 Uncontrolled studies of COVID-19 vaccines in pregnant persons

In Table 3**,** we present a proportional *meta*-analysis from uncontrolled studies or from the active arms of RCTs regarding pregnancy and safety outcomes for COVID-19 vaccines, alongside background rates (global and from the same countries where the studies were conducted). The estimated prevalence was 4.97% (95% CI 2.40–8.39, 5 studies) for spontaneous abortion/miscarriage (background rate [BR] 10%)[101]; 0.05 (0.00–0.20, 3 studies) for stillbirth (BR 2.9–9.6%)[102], [103]; 0.49 (0.34–0.66, 2 studies) for fetal growth retardation (BR 23.8% newborns per year[104]); 7.74% (2.31–15.97, 2 studies) for gestational diabetes mellitus (BR 6–7%)[105]; 2.37% (0.15–7.17, 2 studies) for hypertensive disorders in pregnancy ranged from (BR 4–10%)[106]; 12.30% (7.31–18.4, 1 study) for small for gestational age (BR 1.2–41.5%)[107]; 5.61% (2.58–9.72, 4 studies) for preterm birth (BR 2.58–9.72)[108]; 0% (1 study) for maternal death (BR 2/100,000 livebirths)[109]; 0.43% (0.40–3.76, 1 study) for neonatal death (BR 1.4–6%)[110]; 2.32% (1.42–3.44, 2 studies) for any congenital malformation from (BR 2–4%)[111]; 0.19% (0.17–1.63, 1 study) for suspected chorioamnionitis (BR 1–4%)[112]; 0.37% (0.01–1.22, 1 study) for antenatal bleeding (BR 6%)[113]; 3.24% (2.08–4.65, 1 study) for neonatal infections (BR not available); and 1.82% (1.72–15.31, 1 study) for respiratory distress newborn (BR: 1.2–10%)[114], [115].

**Table 3:** Proportional *meta*-analysis for uncontrolled studies involving COVID-19 vaccines in pregnant persons.

Outcomes		Rate associated with mRNA COVID-19^#^ vaccines % (95 CI)	N Studies	Historical rates
Spontaneous abortion/miscarriage		4.97 (2.40–8.39)	5	10% (in clinically recognized pregnancies)[98]
Stillbirth		0.05 (0.00–0.20)	3	Finland 2010: 2.9%[110] Belgium 2010: 3.1%[99] UK 2017: 4.2%[100] Argentina, USA 2019: 3.0%[99] Australia 2010: 2.9%[99] Vietnam 2010: 9.6%[99] New Zealand 2010: 3.3%[99]
Fetal growth retardation		0.49 (0.34–0.66)	2	23.8% newborns per year[101]
Gestational diabetes		7.74 (2.31–15.97)	2	6–7%[102]
Hypertensive disorders		2.37 (0.15–7.17)	2	Hypertensive disorders: 10% Preeclampsia: 4–5%[103]
Small for gestational age		12.3 (7.31–18.4)	1	Asia: 5.3 to 41.5% Africa: 1.2 to 3.0% livebirths[104]
Preterm birth		5.61 (2.58–9.72)	4	10%[105]
Maternal death		0 (0.00–0.03)*	1	Israel: 2/100,000 livebirths[106]
Any congenital malformation		2.32 (1.42–3.44)	2	2–4%[108]
Neonatal death		0.43 (0.40–3.76)	1	Estimations for 2019[107] Argentina: 6% USA: 3.6% Australia: 2.3% UK: 2.7% Finland: 1.4% New Zealand: 2.6% Israel: 1.9%
Postpartum hemorrhage		10.40 (6.49–15.10)	2	2–5%[109]
Suspected Chorioamnionitis		0.19 (0.17–1.63)	1	USA: 1–4%[111]
Antenatal bleeding		0.37 (0.01–1.22)	1	6%[112]
Neonatal infections		3.24 (2.08–4.65)	1	NA
Respiratory distress newborn		1.82 (1.72–15.31)	1	LMICs: 1.2% to 7.2%[113] USA: 10%[114]
Injection site reactions	LMICs	58.86 (30.08–84.66)	4	–
	HICs			–
Fever		19.28 (0.78–53.55)	3	–
Headache		25.31 (1.95–62.75)	4	–
Gastrointestinal		9.47 (3.33–18.29)	1	–
Myalgia		33.08 (3.51–74.13)	3	–
Fatigue		50.03 (17.52–82.53)	3	–
Vomiting		4.55 (4.19–4.93)	1	–
Nausea		26.61 (25.83–27.39)	1	–
Chills		36.66 (35.81–37.51)	2	–
Joint pain		25.57 (24.80–26.35)	1	–

**MAEs**: Medically attended adverse events; **SAEs**: Serious adverse events; **NA**: Not available; **HICs**: High Income Countries; **LMIC**s: Low-to-Middle-Income Countries; **#**Pfizer/BioNTech and Moderna/NIH (only 0.5% of pregnant persons were exposed to Janssen and Oxford/AstraZeneca vaccines vectored).; *None maternal death the estimated 95% CI is 3.1 to 29.1 / 100,000.

We found a high incidence of PPH (10.40% [6.49–15.10]; 2 studies) in women who received COVID-19 vaccines during pregnancy, in comparison with the published referenced evidence (2–5%).[116] Bookstein et al.[34] included only 57 pregnant persons (of which 10.5% experienced PPH) who received two doses of the BNT162b2 vaccine and delivered during the study period. Blakeway et al. reported that 13 out of the 133 (9.8%) pregnant persons who received mRNA/viral vectored vaccines presented with PPH.[90] No maternal death rate was associated with mRNA vaccines in one study[43]. The estimated 95% CI by the random effect *meta*-analysis could range from 3.1 to 29.1 per 100,000 – very similar in absolute number to the background rate.[109].

The three most frequent non-serious adverse events with mRNA COVID-19 vaccines exposure were fatigue (50.03%), chills (36.66%), and myalgia (33.08%). Data are presently lacking on fetal death, low birth weight, neonatal encephalopathy, failure to thrive, microcephaly, systemic reactions systemic reactions, anaphylaxis, MAEs, SAEs.

We present the related forest plots, the contributing studies, and numerical outputs in **Appendix B**. **Forest-plots of *meta*-analysis of pregnancy outcomes**.

### 4.4 Controlled studies of COVID-19 vaccines in pregnant animals

Two preclinical comparative studies were conducted on pregnant animals. Following vaccination of rats with the mRNA-LNP vaccine BNT162b2, Bowman et al. documented no detrimental effects on fertility, embryo-fetal or postnatal survival, growth, physical development, or neurofunctional development in the offspring through the end of lactation.[92] Following vaccination of mice with the vectored vaccine ChAdOx1 nCoV-19, Stebbings et al. found no detrimental effects on female reproduction, fetal or pup survival, fetal external, visceral, or skeletal findings, pup physical development, and no abnormal gross pathology findings in pups or dams.[97].

### 4.5 Controlled studies of non-COVID-19 vaccines in pregnant persons

A paired *meta*-analysis for the pregnancy outcomes was carried out with data from studies that adjusted for potential confounders. Results are presented by type of exposure (AS03 or aluminum adjuvants) and trimester of exposure in **Appendix A,** Supplementary Table 7 and **Appendix B**. **Forest-plots of *meta*-analysis of pregnancy outcomes**). None of the exposures showed statistically significant associations with adverse outcomes, regardless of the trimester of vaccination. However, exposure to vaccines containing AS03 (mainly influenza H1N1, Tdap, and Hepatitis A/B) was associated with a statistically significant protective effect for four outcomes: stillbirth (aHR 0.67; 95% CI 0.47–0.96) for the second trimester; fetal death (aRR 0.66; 95% CI 0.47–0.93) for the full pregnancy; low birth weight (aOR 0.88; 95% CI 0.81–0.96) for the full pregnancy; cesarean section (aOR 0.93; 95% CI 0.87–0.99) for the second/third trimester. Aluminum was associated with a statistically significant reduction in preterm birth (aHR 0.88; 95% CI 0.80–0.97) for the third trimester. The full pregnancy exposure (any trimester) to aluminum only showed a trend of a small and non-statistically significant unfavorable effect for small for gestational age outcome (aOR 1.18; 95% CI 1.00–1.39) but not in the first trimester (aOR 1.03; 95% CI 0.96–1.11) with a moderate subgroup difference by trimester of exposure (test for subgroup differences I^2^ = 55%). Across maternal and fetal outcomes, the certainty of evidence, according to the GRADE approach, was classified as “low” to “very low”, mainly due to imprecision or inconsistency (**Appendix A,** Supplementary Table 6).

The sensitivity analysis for AS03 exposure, restricted to studies with a low risk of bias, did not allow us to evaluate stillbirth, preeclampsia, and gestational diabetes, which were assessed via aOR by single studies of moderate or high risk of bias. The sensitivity analysis did not change the effect of full pregnancy exposure to AS03 on congenital malformations (the eliminated study[50] only represented 6.4% of the weight of the *meta*-analysis) nor preterm birth or low birth weight. After excluding Ray 2014[79], the vaccine containing AS03 showed a protective effect over the full pregnancy on very preterm birth (aOR 0.73; 95% CI 0.58–0.92) and did not change the effect on cesarean section (measured by aOR).

### 4.6 Uncontrolled studies of non-COVID-19 vaccines in pregnant persons

In **Appendix A,** Supplementary Table 8**,** we present the proportional *meta*-analysis from uncontrolled studies or from the active arms of RCTs regarding pregnancy and safety outcomes for AS03 and aluminum adjuvants in pregnant persosn. As observed for COVID-19 vaccines, the estimated rates of maternal and fetal outcomes were generally consistent with global or population-specific background rates. We present the related forest plots, the contributing studies, and numerical outputs in **Appendix B**. **Forest-plots of *meta*-analysis of pregnancy outcomes**.

Data on medically attended adverse events (MAEs) and serious adverse events (SAEs) were only available for aluminum exposure (proportion pooled estimates of 33.90% [11.79–99.95; 2 studies] and 12.31% [1.27–32.34; 6 studies], respectively). For exposure to AS03, the estimated prevalence of systemic reactions was 41.22% [39.48–42.97; 1 study], 25.82% [17.38–35.29; 1 study] for non-minor systemic reactions, and 0.023% [0.001–0.003; 1 study] for anaphylaxis. The three most frequent non-serious adverse events with aluminum exposure were gastrointestinal issues (17.51% [13.75–21.61]; 2 studies), fatigue (15.32% [4.57–30.84]; 6 studies), and headache (14.32% [2.37–33.92]; 9 studies); and with AS03 exposure these were headache (2.20% [0.19–0.25]; 1 study), fever (0.23% [0.20–0.27]; 1 study), and joint pain (0.05% [0.04–0.07]; 1 study).

### 4.7 Uncontrolled studies of non-COVID-19 vaccines in pregnant animals

Animal studies showed a smaller proportion of abortion with the ChAdOx1 vectored vaccine[97] (3.69%) than with aluminum-adjuvanted vaccines (11.81%) and the same finding was reported for any congenital malformations (0.73 and 5.22%, respectively) [92]. A novel inactivated equine influenza vaccine containing a second-generation ISCOM-Matrix as an adjuvant appear to be safe in pregnant mares.[94]. (See **Appendix B**. **Proportion *meta*-analysis forest plots**).

### 4.8 Ongoing studies in pregnant persons

As of December 2021, we identified five ongoing clinical trials recruiting pregnant persons in the COVID-19 vaccine tracker developed by the Vaccine Centre at the London School of Hygiene and Tropical Medicine databases.[117] These studies may contribute to future *meta*-analyses on this topic and include a phase 2 trial assessing the Ad26.COV2.S vaccine in the USA, Brazil, and South Africa (recruiting) [118]; a phase 2/3 trial assessing the BNT162b2 in the USA, Brazil, South Africa, Spain, and the UK (active, not recruiting) [119]; a phase 2 trial evaluating BNT162b2, mRNA-1273, ChAdOx1-S, and NVX-CoV2373 in the UK (recruiting) [120]; and two ongoing or complete phase 4 open-label trials being conducted in Belgium (evaluating BNT162 and mRNA-1273) and the Netherlands (evaluating BNT162b2, mRNA-1273, and ChAdOx1-S).[121], [122] We also identified a phase 3 open-label RCT evaluating a vaccine regimen of the Ebola vaccine Ad26.ZEBOV in Rwanda (active, not recruiting).[123].

## 5 Discussion

This systematic review and *meta*-analysis evaluated the effects of COVID-19 vaccines administered during pregnancy, or their components/platforms used in other maternal vaccines on adverse pregnancy and non-pregnancy outcomes. Among 71 studies included in the review, there were seven studies of COVID-19 vaccine in humans. Regarding the list of authorized or approved COVID-19 vaccines up to December 2021 that guided our study, in January 2023 Shifa Pharmed - Barkat CovIran® vaccine (Inactivated, produced in Vero cell) from Iran, Nuvaxovid (recombinant nanoparticle prefusion spike protein formulated with Matrix-M™ adjuvant) and GBP510 MFDS (recombinant protein subunit) from RoKorea, and Corbevax (RBD antigen of SARS CoV-2) from India were the only vaccines with dossier accepted for review added to the list.[124] These candidate vaccines should be considered for future systematic reviews. The vast majority of pregnant persons included (>99%) were exposed to mRNA vaccines and were recruited from high-income countries. Only two comparative studies comparing exposure with non-exposure to COVID-19 vaccines were identified, covering seven perinatal outcomes (spontaneous abortion, congenital malformation, preterm birth, small for gestational age, fetal growth restriction, preeclampsia, and postpartum hemorrhage). None of the adjusted relative effects were significantly associated with adverse outcomes between exposed and unexposed pregnant persons, either clinically or statistically.

This review also presents indirect evidence regarding COVID vaccine components used in other vaccines (platform, components, or adjuvants) in pregnant humans and animals. We mostly found exposures to AS03 or aluminum-based adjuvants in maternal vaccines, and none were statistically significantly associated with adverse pregnancy outcomes. For some outcomes (stillbirth, fetal death, 5-minute Apgar score < 7, low birth weight, cesarean section, and preterm birth), these vaccines even showed protective effects associated with specific exposure times. These findings were supported by the sensitivity analysis, restricted to studies with a low risk of bias, that showed minimal to no important differences and a slightly more protective effect than in the primary analysis. These effects cannot be separated from the vaccines themselves, e.g., the influenza vaccine resulting in reductions in adverse pregnancy outcomes, or from its adjuvants. However, this apparent protective effect might be because the likelihood of being vaccinated increases with the length of gestation.[125].

We also performed proportional *meta*-analyses of safety and reactogenicity. The proportions of adverse maternal and neonatal outcomes were similar to background rates published before the pandemic.[101], [102], [103], [104], [105], [106], [107], [108], [109], [110], [111], [113], [114], [115], [116], [126], [127], [128], [129], [130], [131] The only outcome that exceeded the expected background rates of 2.0 to 5%[116] was PPH with a pooled proportion of 10.40% (data from Bookstein et al.[34] and Blakeway et al.[90]). However, Blakeway et al. also compared exposed with non-exposed pregnant persons, with similar rates in these two groups (9.8% vs. 9.0%, respectively; aOR after adjusting for propensity score of 1.09 [95% CI 0.56–2.12]). The width of this confidence interval, related to the small sample size, suggests a cautious interpretation but deserves further research. Reactogenicity was similar following exposure to COVID-19 vaccines, AS03, and aluminum except for fatigue, chills, and myalgia, which were more frequently reported after mRNA vaccines than other adjuvanted maternal vaccines.[85].

Our study shows no safety concerns with mRNA vaccines in pregnant persons in high-income countries. Most available studies are limited by small sample size or the lack of an unvaccinated comparison group. We found scarce data on the safety of adenoviral and protein subunit-adjuvant vaccines in pregnancy. However, after closing our literature search, a few studies provide some insight into these vaccines. A cross-sectional study using data from the Brazilian surveillance information system estimated an incidence of adverse events of 309.37/100,000 doses (95% CI 297.23–321.51), with a lower incidence associated with the Sinovac/Butantan inactivated vaccine (74.08/100,000 doses; 95% CI 63.47–84.69) than the other administered mRNA vaccines and vectored vaccines (Pfizer/BioNTech, AstraZeneca, and Janssen).[132] Since the start of a COVID-19 vaccine program in Scotland, fewer pregnant persons required hospitalization and critical care admission within 28 days of COVID-19 vaccination than within 28 days of SARS-CoV-2 infection. However, the vaccine uptake rate in pregnant persons was lower than in all women.[133] A case-control study with data from Norwegian registries on first-trimester pregnancies found that among persons with miscarriages, the adjusted odds ratios for Covid-19 vaccination were 0.81 (95% CI 0.69–0.95) for vaccination in the previous five weeks, consistent with a protective effect of vaccination. The results were similar across vaccine types (Pfizer/BioNTech, Moderna, AstraZeneca) and the number of doses received (one or two).[134] A retrospective cohort study including all persons who delivered between January and June 2021 at the largest birth center in Israel and applying multivariable analyses found no differences between vaccinated (second or third trimesters) and unvaccinated persons for delivery and newborn complications, the incidence of small for gestational age, and newborn respiratory complications.[135] Two recent observational studies in Israel, reported that maternal outcomes were comparable among vaccinated and non-vaccinated pregnant persons and that uptake of the mRNA COVID‐19 vaccines was not associated with worse maternal outcomes.[136], [137] A large, multisite, retrospective cohort study in the USA found that receipt of mRNA COVID-19 vaccine during pregnancy was not associated with increased risk for preterm birth or small for gestational age at birth. In that study, only 4.2% of pregnant persons received a vectored vaccine.[138] In a birth cohort from the Mayo Clinic Health System in the USA, COVID-19 vaccination during pregnancy was not associated with increased pregnancy or delivery complications.[139].

Concerning data from LMICs, there are some safety data regarding pregnant persons receiving Ad26-based Ebola vaccines and the vesicular stomatitis virus-vectored vaccines[140] (using components/platforms also used by COVID-19 vaccines), but no data on the safety of COVID-19 vaccination. Considerable evidence supports the safety of non-COVID-19 vaccines that use similar adjuvants (AS03 and aluminum) during pregnancy like influenza, hepatitis A and B, and Tdap. An overview of systematic reviews on adverse events and maternal immunization did not identify any risks for any vaccine and perinatal outcome.[141] Ten systematic reviews supported the safety of influenza vaccines during pregnancy.[142], [143], [144], [145], [146], [147], [148], [149], [150], [151] One systematic review evaluated the safety of the hepatitis B vaccine, the pneumococcal polysaccharide vaccine, and the meningococcal polysaccharide vaccine during pregnancy and found no clear association with teratogenic effects, preterm labor, or spontaneous abortion.[152] Another systematic found moderate- to high-certainty evidence supporting the safety of vaccines frequently given to travelers, such as yellow fever, MMR (measles, mumps and rubella), influenza, Tdap, meningococcus, or hepatitis A and B in the context of pregnancy. [153]A recent rapid review published by our group showed that no safety concerns were associated with exposure to maternal vaccines with adjuvants used in COVID-19 vaccines.[154] Our findings in this full systematic review reinforce these initial preliminary conclusions.

We also found that the local and systemic reactogenicity observed with COVID-19 vaccines (mostly mRNA) are more prevalent than other adjuvanted maternal vaccines analyzed in this study including influenza, Tdap, and hepatitis A and B vaccines, in outcomes such as fatigue, chills, joint pain, and myalgia. However, the overall reactogenicity profile of mRNA vaccines seems similar between pregnant and nonpregnant populations in the literature.[85].

This systematic review has several strengths. First, we included studies from humans and animals to provide a complete and timely response to an important topic, regardless of time, language, or publication type, and spanning a wide range of maternal and neonatal outcomes. Second, we adhered to strict recommended quality standards for conducting systematic reviews, including independent data extraction and risk of bias assessment, and a comprehensive search strategy. Third, considering the still relatively scarce direct evidence of the safety of COVID-19 for pregnant persons, we categorized exposure to its vaccine components and platforms used in non-COVID-19 vaccines. Additionally, to avoid immortal time bias, a common problem in observational studies involving exposure in late pregnancy,[155] we only *meta*-analyzed comparative studies that adjusted for potential confounders. Since not all the studies adjusted for the time of exposure, we presented *meta*-analyses by trimester of exposure, and we did not find evidence of important differences between subgroups. We also provided estimations and historical rates for each region or country to to compare the safety outcomes. In summary, we synthesized, all currently available data, and critically appraised a substantial body of evidence to assess the COVID-19 vaccines in pregnant human and in animal and also other maternal non-COVID-19 vaccines.

Our study is not exempt from limitations. The majority of included studies are observational study designs, and only 30% allowed for comparisons between vaccinated and unvaccinated pregnant persons. Nevertheless, regardless of the study design and publication type, the consistent absence of safety concerns suggests that findings are robust to this limitation. Only seven studies (9.8%) reported safety information regarding COVID-19 vaccines in pregnant persons, almost exclusively exposed to mRNA-LNP vaccines, and only two studies allowed comparison of perinatal outcomes.[43], [90] The paucity of direct safety data on non-mRNA vaccines is an important issue for pregnant persons and policymakers, especially in settings without access to BNT162b2 and mRNA-1273. Remarkably, only 9% of the total body of evidence comes from LMICs, limiting the generalizability to these settings, and more data from LMICs are urgently required.

Maternal immunization policies relating to COVID-19 have varied across different countries, ranging from restrictive policies that do not allow the vaccination of pregnant persons to inclusive policies that recommend or encourage maternal vaccination. From the information gathered via the COVID-19 Maternal Immunization Tracker,[12] as of July 12, 2022 COVID-19 immunization for some or all pregnant persons is recommended by 50% (n = 56) high-income countries, 30% (n = 33) upper-middle-income countries, 18% (n = 20) lower-middle-income countries, and only 5% (n = 5) low-income countries. Additionally, access, acceptance, and availability of vaccines remain very uneven worldwide.[12].

In summary, based on the available data, there is no evidence of obvious safety risks associated with the use of COVID-19 vaccines. Therefore, it is reasonable to recommend mRNA COVID-19 vaccines for pregnant persons as they are at higher risk for adverse outcomes. Data for non-mRNA vaccines are sparse, and further studies are urgently needed, especially in LMICs. Until new evidence is available, all local contextual factors should be considered for decision-making. Confidence in vaccine safety and effectiveness are known to be key predictors for vaccine acceptance,[14] and the ongoing studies will contribute additional data on vaccine safety and efficacy in both mothers and infants.

Considering the growing body of evidence on this topic, we are conducting a living systematic review to evaluate the safety, immunogenicity, and efficacy/effectiveness of COVID-19 vaccines for pregnant persons.[156] The results are being presented in a web-based, highly parameterizable interface is provided that yields available estimates and live *meta*-analyses of the above outcomes of interest by relevant subgroups.[157] We included 134 studies (87% RNA vaccines) published until December 31, 2022. We found>20 new studies that our present review evaluating the safety of COVID-19 vaccines during pregnancy [158], [159], [160], [161], [162], [163], [164], [165], [166], [167], [168], [169], [170], [171], [172], [173], [174], [175], [176], [88], [177]. Most of studies were conducted in Europe[159], [160], [162], [169], [170], [171], [177], North America[163], [168], [172], [173], [175], [88] and Asia[158], [161], [164], [167], [174], [176].

Only one study, conducted in the UK, Brazil, and South Africa, reported data on women who had participated in COVID-19 vaccine trials and had unintentionally become pregnant[165]. Almost all studies assessed the safety of mRNA COVID-19 vaccines, although there is some information on recombinant vaccine safety (ChAdOx1 nCoV-19)[8]. All except one study[161] reported no concerning trends of AEFIs or maternal-neonatal complications. In contrast with the rest of the evidence, Dick et al.[161] reported that there might be an increase in the rate of preterm birth in pregnant persons who received the vaccine during their second trimester, but there was no direct association between vaccination and adverse pregnancy and infant outcomes.

In conclusion, we found no evidence of safety concerns for currently used COVID-19 vaccines during pregnancy. The evidence provided should assist pregnant persons, healthcare providers, and policymakers in supporting COVID-19 vaccination for this group.

## Declaration of Competing Interest

The authors declare the following financial interests/personal relationships which may be considered as potential competing interests: [Pierre M. Buekens reports financial support was provided by Bill & Melinda Gates Foundation].

## Data Availability

Data will be made available on request.
