# Supplementary material for: Safety of COVID-19 vaccines during pregnancy: A systematic review and meta-analysis
Source: Vaccine. 2023 Jun 7;41(25):3688–700. doi: 10.1016/j.vaccine.2023.03.038 (PMC10040368; doi:10.1016/j.vaccine.2023.03.038)
Supplement: Supplementary data 1 [file mmc1.docx]

**Index Supplememntary Material** **Appendix A**(with hyperlinks to specific sections)

[**Table 1. PRISMA checklist** 2](#_Toc108382755)

[**Table 2. COVID-19 vaccines components** 5](#_Toc108382756)

[**Methods.** 6](#_Toc108382757)

[Search strategy 6](#_Toc108382758)

[Risk of bias assessment tools by study design 13](#_Toc108382759)

[**Table 3. List and exclusion reasons of excluded studies** 20](#_Toc108382760)

[**Table 4. Characteristics of included studies for non-COVID-19 vaccines** 23](#_Toc108382761)

[**Table 5. Risk of bias assessments for COVID-19 vaccines** 26](#_Toc108382762)

[**Table 6. Risk of bias assessments for non-COVID-19 vaccines** 26](#_Toc108382763)

[**Table 7. Meta-analysis and certainty of evidence for controlled studies involving non-COVID-19 vaccines in pregnant persons** 28](#_Toc108382764)

[**Table 8. Proportional meta-analysis for uncontrolled studies involving non-COVID-19 vaccines in pregnant persons** 30](#_Toc108382765)

[**References** 33](#_Toc108382766)

# **Table 1.** **PRISMA checklist**

| **Section and Topic** | **Item #** | **Checklist item** | **Location where item is reported** |
| --- | --- | --- | --- |
| **TITLE** | | |  |
| Title | 1 | Identify the report as a systematic review. | 1 |
| **ABSTRACT** | | |  |
| Abstract | 2 | See the PRISMA 2020 for Abstracts checklist. | 1-2 |
| **INTRODUCTION** | | |  |
| Rationale | 3 | Describe the rationale for the review in the context of existing knowledge. | 2-3 |
| Objectives | 4 | Provide an explicit statement of the objective(s) or question(s) the review addresses. | 4 |
| **METHODS** | | |  |
| Eligibility criteria | 5 | Specify the inclusion and exclusion criteria for the review and how studies were grouped for the syntheses. | 4-5 |
| Information sources | 6 | Specify all databases, registers, websites, organisations, reference lists and other sources searched or consulted to identify studies. Specify the date when each source was last searched or consulted. | 5 |
| Search strategy | 7 | Present the full search strategies for all databases, registers and websites, including any filters and limits used. | 5 and Appendix 1 |
| Selection process | 8 | Specify the methods used to decide whether a study met the inclusion criteria of the review, including how many reviewers screened each record and each report retrieved, whether they worked independently, and if applicable, details of automation tools used in the process. | 6 |
| Data collection process | 9 | Specify the methods used to collect data from reports, including how many reviewers collected data from each report, whether they worked independently, any processes for obtaining or confirming data from study investigators, and if applicable, details of automation tools used in the process. | 6 |
| Data items | 10a | List and define all outcomes for which data were sought. Specify whether all results that were compatible with each outcome domain in each study were sought (e.g. for all measures, time points, analyses), and if not, the methods used to decide which results to collect. | 6 |
|  | 10b | List and define all other variables for which data were sought (e.g. participant and intervention characteristics, funding sources). Describe any assumptions made about any missing or unclear information. | 6 |
| Study risk of bias assessment | 11 | Specify the methods used to assess risk of bias in the included studies, including details of the tool(s) used, how many reviewers assessed each study and whether they worked independently, and if applicable, details of automation tools used in the process. | Appendix 2 |
| Effect measures | 12 | Specify for each outcome the effect measure(s) (e.g. risk ratio, mean difference) used in the synthesis or presentation of results. | 6 |
| Synthesis methods | 13a | Describe the processes used to decide which studies were eligible for each synthesis (e.g. tabulating the study intervention characteristics and comparing against the planned groups for each synthesis (item #5)). | 6-7 |
|  | 13b | Describe any methods required to prepare the data for presentation or synthesis, such as handling of missing summary statistics, or data conversions. | 6 |
|  | 13c | Describe any methods used to tabulate or visually display results of individual studies and syntheses. | 6 |
|  | 13d | Describe any methods used to synthesize results and provide a rationale for the choice(s). If meta-analysis was performed, describe the model(s), method(s) to identify the presence and extent of statistical heterogeneity, and software package(s) used. | NA |
|  | 13e | Describe any methods used to explore possible causes of heterogeneity among study results (e.g. subgroup analysis, meta-regression). | NA |
|  | 13f | Describe any sensitivity analyses conducted to assess robustness of the synthesized results. | NA |
| Reporting bias assessment | 14 | Describe any methods used to assess risk of bias due to missing results in a synthesis (arising from reporting biases). | Not described |
| Certainty assessment | 15 | Describe any methods used to assess certainty (or confidence) in the body of evidence for an outcome. | Not described |
| **RESULTS** | | |  |
| Study selection | 16a | Describe the results of the search and selection process, from the number of records identified in the search to the number of studies included in the review, ideally using a flow diagram. | Figure 1 |
|  | 16b | Cite studies that might appear to meet the inclusion criteria, but which were excluded, and explain why they were excluded. | Appendix 3 |
| Study characteristics | 17 | Cite each included study and present its characteristics. | Table 2. 3 and 4 |
| Risk of bias in studies | 18 | Present assessments of risk of bias for each included study. | Table 5 and 6 |
| Results of individual studies | 19 | For all outcomes, present, for each study: (a) summary statistics for each group (where appropriate) and (b) an effect estimate and its precision (e.g., confidence/credible interval), ideally using structured tables or plots. | Table 2 and 3 |
| Results of syntheses | 20a | For each synthesis, briefly summarise the characteristics and risk of bias among contributing studies. | NA |
|  | 20b | Present results of all statistical syntheses conducted. If meta-analysis was done, present for each the summary estimate and its precision (e.g. confidence/credible interval) and measures of statistical heterogeneity. If comparing groups, describe the direction of the effect. | NA |
|  | 20c | Present results of all investigations of possible causes of heterogeneity among study results. | Not described |
|  | 20d | Present results of all sensitivity analyses conducted to assess the robustness of the synthesized results. | Not described |
| Reporting biases | 21 | Present assessments of risk of bias due to missing results (arising from reporting biases) for each synthesis assessed. | NA |
| Certainty of evidence | 22 | Present assessments of certainty (or confidence) in the body of evidence for each outcome assessed. | Not described |
| **DISCUSSION** | | |  |
| Discussion | 23a | Provide a general interpretation of the results in the context of other evidence. | 17 |
|  | 23b | Discuss any limitations of the evidence included in the review. | 18 |
|  | 23c | Discuss any limitations of the review processes used. | 18 |
|  | 23d | Discuss implications of the results for practice, policy, and future research. | 18-19 |
| **OTHER INFORMATION** | | |  |
| Registration and protocol | 24a | Provide registration information for the review, including register name and registration number, or state that the review was not registered. | 7 |
|  | 24b | Indicate where the review protocol can be accessed, or state that a protocol was not prepared. | 7 |
|  | 24c | Describe and explain any amendments to information provided at registration or in the protocol. | NA |
| Support | 25 | Describe sources of financial or non-financial support for the review, and the role of the funders or sponsors in the review. | 19 |
| Competing interests | 26 | Declare any competing interests of review authors. | 19 |
| Availability of data, code and other materials | 27 | Report which of the following are publicly available and where they can be found: template data collection forms; data extracted from included studies; data used for all analyses; analytic code; any other materials used in the review. | NA |

# **Table 2.** **COVID-19 vaccines components**

| Platform | Details | Name of the Vaccine | Developer/ manufacturer | Adjuvant |
| --- | --- | --- | --- | --- |
| Protein subunit | Recombinant nanoparticle prefusion spike protein | NVX-CoV2373/Nuvaxovid | Novavax | Matrix-M™ |
|  |  | NVX-CoV2373/Covovax | Serum Institute of India | Matrix-M™ |
|  | Recombinant protein vaccine | CoV2 preS dTM-AS03 vaccine | Sanofi | AS03 |
|  | Recombinant protein of the dimeric-RBD of SARS-CoV-2 virus | Soberana 01.  Soberana 02  Soberana Plus | Finlay Institute de Vaccines | Alum |
|  | Recombinant protein of the dimeric-RBD of SARS-CoV-2 virus | Abdala | Center for Genetic Engineering and Biotechnology (CIGB) | Alum |
|  | Peptide antigens of SARS-CoV-2 proteins | EpiVacCorona | Vector State Research Centre of Viralogy and Biotechnology | Alum |
|  | Recombinant protein subunit of dimeric-RBD of SARS-CoV-2 virus | Recombinant Novel Coronavirus Vaccine (CHO Cell) | Zhifei Longcom, China | Alum |
|  | Novel Recombinant SARS-CoV-2 Spike (S)- trimer fusion protein | SCB-2019 | Clover Biopharmaceuticals | CpG1018/Alum |
| Vectored | Recombinant, replication-incompetent Ad26 vectored vaccine encoding the (SARS-CoV-2) Spike (S) protein | Ad26.COV.2S | Johnson & Johnson/Janssen | No |
|  | Human Adenovirus Vector-based Covid-19 vaccine | Sputnik V | Russian Direct Investment Fund | No |
|  | Recombinant Novel Coronavirus Vaccine (Adenovirus Type 5 Vector) | Ad5-CoV | CanSinoBIO | No |
|  | Recombinant ChAdOx1 adenoviral vector encoding the Spike protein antigen of the SARS-CoV-2. | AZD1222 Vaxzevria | U Oxford / AstraZeneca | No |
|  |  | Covishield (ChAdOx1_nCov-19) | Serum Institute of India | No |
| Nucleic acid / mRNA-LNP | mRNA: encodes 2P-stabilized Spike, TM, FI | mRNA-1273 | Moderna | No |
|  | mRNA: encodes stabilized SARS-CoV-2 Spike | BNT162a b2  COMIRNATY | BioNTech / Pfizer | No |
|  | mRNA/LNP full-length S-protein stabilized | CVnCoV/CV07050101 mRNAnCoV-19 | CureVac | No |
| Inactivated virus | Inactivated virus, produced in Vero cells | SARS-CoV-2 Vaccine (Vero Cell), Inactivated (lnCoV) | Sinopharm / Beijing Institute of Biological Products Co., Ltd. (BIBP) | Alum |
|  |  | SARS-CoV-2 Vaccine (Vero Cell), Inactivated (lnCoV) | Sinopharm / Wuhan Institute of Biological Products Co Ltd  (WIBP) | Alum |
|  |  | COVID-19 Vaccine (Vero Cell), Inactivated/ Coronavac^TM^ | Sinovac Life Sciences Co., Ltd | Alum |
|  |  | SARS-CoV-2 Vaccine, Inactivated (Vero Cell) | IMBCAMS, China | Alum |
|  | Whole virion inactivated  SARS-CoV-2 | SARS-CoV-2 Vaccine, Inactivated (Vero Cell)/  COVAXIN | Bharat Biotech, India | Alum |

**LNP**: lipid nanoparticle; **AS:** Adjuvant System; **CpG**: Cytosine phosphoGuanosine; **MRC**: Human Fetal Lung Fibroblast Cells; **CHO**: Chinese hamster ovary; **TM**: transmembrane domain; **S**: Spike; **FI**: formalin-inactivated; **rS**: recombinant Spike: **RBD:** receptor-binding domain of the SARS-CoV-2 virus

# **Methods.**

## **Search strategy**

**Ovid MEDLINE**

1 exp Pregnancy

2 Pregnan*.ti,ab.

3 exp Pregnancy Complications

4 exp Abortion, Spontaneous

5 Abortion*.ti,ab.

6 Miscarriage*.ti,ab.

7 Gestational.ti,ab.

8 exp Parturition/

9 Childbirth*.ti,ab.

10 Parturition*.ti,ab.

11 Partum.ti,ab.

12 exp Fetus/

13 Fetal.ti,ab.

14 Fetus.ti,ab.

15 Maternofetal.ti,ab.

16 (Materno adj1 Fetal).ti,ab.

17 Fetomaternal.ti,ab.

18 DART.ti,ab.

19 or/1-18

20 exp Adjuvants, Immunologic

21 Immunoadjuvant*.ti,ab.

22 (Immunologic* adj1 Adjuvant*).ti,ab.

23 Matrix-M*.mp.

24 Alhydrogel.mp.

25 Aluminum*.mp.

26 AS03.mp.

27 MF59.mp.

28 CpG1.mp.

29 (Recombinant adj1 Spike-Protein).mp.

30 Baculo*.mp.

31 (Stabilized adj1 Spike).mp.

32 S-Protein*.mp.

33 (Molecular adj1 Clamp).mp.

34 (Full adj1 Length).mp.

35 (Replication adj1 Incompetent).mp.

36 Ad26*.mp.

37 Adenovirus-26.mp.

38 Ad5*.mp.

39 Adenovirus-5.mp.

40 ChAdOx1.mp.

41 Measles-Vector.mp.

42 V591.mp.

43 AZD1222.mp.

44 BNT162b2.mp.

45 mRNA*.mp.

46 (Vector adj1 Expressing).mp.

47 exp Influenza Vaccines/

48 (Influenza adj1 Vaccine*).mp.

49 (Flu adj1 Vaccine*).mp.

50 exp Saponins/

51 Saponin*.mp.

52 exp Nanoparticles/

53 Nanoparticle*.mp.

54 LNP.mp.

55 Nanocrystal*.mp.

56 Arepanrix.mp.

57 Pandemrix.mp.

58 FLUAD.mp.

59 Dynavax.mp.

60 exp Hepatitis B Vaccines/

61 (Hepatitis-B adj1 Vaccin*).mp.

62 HEPLISAV-B.mp.

63 (Polyethylene adj1 Glycol).mp.

64 or/20-63

65 exp Vaccination/

66 exp Vaccines/

67 Vaccin*.ti,ab.

68 or/65-67

69 19 and 64 and 68

70 exp Nanoparticles/

71 Nanoparticle*.mp.

72 LNP.mp.

73 Nanocrystal*.mp.

74 or/70-73

75 exp Pregnancy/

76 Pregnan*.ti,ab.

77 DART.ti,ab.

78 or/75-77

79 exp Animals/

80 74 and 78 and 79

81 69 or 80

**EMBase**

#83. #70 OR #82

#82. #81 NOT #80

#81. #75 AND #79

#80. 'animal'/exp

#79. #76 OR #77 OR #78

#78. dart:ti,ab

#77. pregnan*:ti,ab

#76. 'pregnancy'/exp

#75. #71 OR #72 OR #73 OR #74

#74. nanocrystal*:ti,ab

#73. lnp:ti,ab

#72. nanoparticle*:ti,ab

#71. 'nanoparticle'/exp

#70. #19 AND #65 AND #69

#69. #66 OR #67 OR #68

#68. vaccin*:ti,ab

#67. 'vaccine'/exp

#66. 'vaccination'/exp

#65. #20 OR #21 OR #22 OR #23 OR #24 OR #25 OR #26 OR #27 OR #28 OR #29 OR #30 OR #31 OR #32 OR #33 OR #34 OR #35 OR #36 OR #37 OR #38 OR #39 OR #40 OR #41 OR #42 OR #43 OR #44 OR #45 OR #46 OR #47 OR #48 OR #49 OR #50 OR #51 OR #52 OR #53 OR #54 OR #55 OR #56 OR #57 OR #58 OR #59 OR #60 OR #61 OR #62 OR #63 OR #64

#64. (polyethylene NEAR/1 glycol):ti,ab

#63. 'heplisav b':ti,ab

#62. ('hepatitis b' NEAR/1 vaccin*):ti,ab

#61. 'hepatitis b vaccine'/exp

#60. dynavax:ti,ab

#59. fluad:ti,ab

#58. pandemrix:ti,ab

#57. arepanrix:ti,ab

#56. nanocrystal*:ti,ab

#55. lnp:ti,ab

#54. nanoparticle*:ti,ab

#53. 'nanoparticle'/exp

#52. saponin*:ti,ab

#51. 'saponin'/exp

#50. (flu NEAR/1 vaccine*):ti,ab

#49. (influenza NEAR/1 vaccine*):ti,ab

#48. 'influenza vaccine'/exp

#47. (vector NEAR/1 expressing):ti,ab

#46. 'mrna lnp':ti,ab

#45. mrna*:ti,ab

#44. bnt162b2:ti,ab

#43. azd1222:ti,ab

#42. v591:ti,ab

#41. 'measles vector':ti,ab

#40. chadox1:ti,ab

#39. 'adenovirus 5':ti,ab

#38. ad5*:ti,ab

#37. 'adenovirus 26':ti,ab

#36. ad26*:ti,ab

#35. (replication NEAR/1 incompetent):ti,ab

#34. (full NEAR/1 length):ti,ab

#33. (molecular NEAR/1 clamp):ti,ab

#32. 's protein*':ti,ab

#31. (stabilized NEAR/1 spike):ti,ab

#30. baculo*:ti,ab

#29. (recombinant NEAR/1 'spike protein'):ti,ab

#28. cpg1:ti,ab

#27. mf59:ti,ab

#26. as03:ti,ab

#25. aluminum*:ti,ab

#24. alhydrogel:ti,ab

#23. 'matrix m*':ti,ab

#22. (immunologic* NEAR/1 adjuvant*):ti,ab

#21. immunoadjuvant*:ti,ab

#20. 'immunological adjuvant'/exp

#19. #1 OR #2 OR #3 OR #4 OR #5 OR #6 OR #7 OR #8 OR #9 OR #10 OR #11 OR #12 OR #13 OR #14 OR #15 OR #16 OR #17 OR #18

#18. dart:ti,ab

#17. fetomaternal:ti,ab

#16. (materno NEAR/1 fetal):ti,ab

#15. maternofetal:ti,ab

#14. fetus:ti,ab

#13. fetal:ti,ab

#12. 'fetus'/exp

#11. partum:ti,ab

#10. parturition*:ti,ab

#9. childbirth*:ti,ab

#8. 'birth'/exp

#7. gestational:ti,ab

#6. miscarriage*:ti,ab

#5. abortion*:ti,ab

#4. 'spontaneous abortion'/exp

#3. 'pregnancy complication'/exp

#2. pregnan*:ti,ab

#1. 'pregnancy'/exp

**Cochrane Library (Wiley)**

#1 MeSH descriptor: [Pregnancy] explode all trees

#2 Pregnan*:ti,ab,kw

#3 MeSH descriptor: [Pregnancy Complications] explode all trees

#4 MeSH descriptor: [Abortion, Spontaneous] explode all trees

#5 Abortion*:ti,ab,kw

#6 Miscarriage*:ti,ab,kw

#7 Gestational:ti,ab,kw

#8 MeSH descriptor: [Parturition] explode all trees

#9 Childbirth*:ti,ab,kw

#10 Parturition*:ti,ab,kw

#11 Partum:ti,ab,kw

#12 MeSH descriptor: [Fetus] explode all trees

#13 Fetal:ti,ab,kw

#14 Fetus:ti,ab,kw

#15 Maternofetal:ti,ab,kw

#16 (Materno NEAR/1 Fetal):ti,ab,kw

#17 Fetomaternal:ti,ab,kw

#18 DART:ti,ab,kw

#19 #1 OR #2 OR #3 OR #4 OR #5 OR #6 OR #7 OR #8 OR #9 OR #10 OR #11 OR #12 OR #13 OR #14 OR #15 OR #16 OR #17 OR #18

#20 MeSH descriptor: [Adjuvants, Immunologic] explode all trees

#21 Immunoadjuvant*:ti,ab,kw

#22 (Immunologic* NEAR/1 Adjuvant*):ti,ab,kw

#23 Matrix-M*:ti,ab,kw

#24 Alhydrogel:ti,ab,kw

#25 Aluminum*:ti,ab,kw

#26 AS03:ti,ab,kw

#27 MF59:ti,ab,kw

#28 CpG1:ti,ab,kw

#29 (Recombinant NEAR/1 Spike-Protein):ti,ab,kw

#30 Baculo*:ti,ab,kw

#31 (Stabilized NEAR/1 Spike):ti,ab,kw

#32 S-Protein*:ti,ab,kw

#33 (Molecular NEAR/1 Clamp):ti,ab,kw

#34 (Full NEAR/1 Length):ti,ab,kw

#35 (Replication NEAR/1 Incompetent):ti,ab,kw

#36 Ad26*:ti,ab,kw

#37 Adenovirus-26:ti,ab,kw

#38 Ad5*:ti,ab,kw

#39 Adenovirus-5:ti,ab,kw

#40 ChAdOx1:ti,ab,kw

#41 (Measles NEAR/1 Vector):ti,ab,kw

#42 V591:ti,ab,kw

#43 AZD1222:ti,ab,kw

#44 BNT162b2:ti,ab,kw

#45 mRNA*:ti,ab,kw

#46 mRNA-LNP:ti,ab,kw

#47 (Vector NEAR/1 Expressing):ti,ab,kw

#48 MeSH descriptor: [Influenza Vaccines] explode all trees

#49 (Influenza NEAR/1 Vaccine*):ti,ab,kw

#50 (Flu NEAR/1 Vaccine*):ti,ab,kw

#51 MeSH descriptor: [Saponins] explode all trees

#52 Saponin*:ti,ab,kw

#53 MeSH descriptor: [Nanoparticles] explode all trees

#54 Nanoparticle*:ti,ab,kw

#55 LNP:ti,ab,kw

#56 Nanocrystal*:ti,ab,kw

#57 Arepanrix:ti,ab,kw

#58 Pandemrix:ti,ab,kw

#59 FLUAD:ti,ab,kw

#60 Dynavax:ti,ab,kw

#61 MeSH descriptor: [Hepatitis B Vaccines] explode all trees

#62 (Hepatitis-B NEAR/1 Vaccin*):ti,ab,kw

#63 HEPLISAV-B:ti,ab,kw

#64 (Polyethylene NEAR/1 Glycol):ti,ab,kw

#65 #20 OR #21 OR #22 OR #23 OR #24 OR #25 OR #26 OR #27 OR #28 OR #29 OR #30 OR #31 OR #32 OR #33 OR #34 OR #35 OR #36 OR #37 OR #38 OR #39 OR #40 OR #41 OR #42 OR #43 OR #44 OR #45 OR #46 OR #47 OR #48 OR #49 OR #50 OR #51 OR #52 OR #53 OR #54 OR #55 OR #56 OR #57 OR #58 OR #59 OR #60 OR #61 OR #62 OR #63 OR #64

#66 MeSH descriptor: [Vaccines] explode all trees

#67 MeSH descriptor: [Vaccination] explode all trees

#68 Vaccin*:ti,ab,kw

#69 #66 OR #67 OR #68

#70 #19 AND #65 AND #69

**CINAHL (EBSCO)**

S85 S72 OR S84

S84 S82 AND S83

S83 (MH "Animals+")

S82 S77 AND S81

S81 S78 OR S79 OR S80

S80 TI DART OR AB DART

S79 TI Pregnan* OR AB Pregnan*

S78 (MH "Pregnancy+")

S77 S73 OR S74 OR S75 OR S76

S76 TI Nanocrystal* OR AB Nanocrystal*

S75 TI LNP OR AB LNP

S74 TI Nanoparticle* OR AB Nanoparticle*

S73 (MH "Nanoparticles")

S72 S19 AND S67 AND S71

S71 S68 OR S69 OR S70

S70 TI Vaccin* OR AB Vaccin*

S69 (MH "Immunization+")

S68 (MH "Vaccines+")

S67 S20 OR S21 OR S22 OR S23 OR S24 OR S25 OR S26 OR S27 OR S28 OR S29 OR S30 OR S31 OR S32 OR S33 OR S34 OR S35 OR S36 OR S37 OR S38 OR S39 OR S40 OR S41 OR S42 OR S43 OR S44 OR S45 OR S46 OR S47 OR S48 OR S49 OR S50 OR S51 OR S52 OR S53 OR S54 OR S55 OR S56 OR S57 OR S58 OR S59 OR S60 OR S61 OR S62 OR S63 OR S64 OR S65 OR S66

S66 TI (Polyethylene N1 Glycol) OR AB (Polyethylene N1 Glycol)

S65 TI HEPLISAV-B OR AB HEPLISAV-B

S64 TI (Hepatitis-B N1 Vaccin*) OR AB (Hepatitis-B N1 Vaccin*)

S63 (MH "Hepatitis B Vaccines+")

S62 TI Dynavax OR AB Dynavax

S61 TI FLUAD OR AB FLUAD

S60 TI Pandemrix OR AB Pandemrix

S59 TI Arepanrix OR AB Arepanrix

S58 TI Nanocrystal* OR AB Nanocrystal*

S57 TI LNP OR AB LNP

S56 TI Nanoparticle* OR AB Nanoparticle*

S55 (MH "Nanoparticles")

S54 TI Saponin* OR AB Saponin*

S53 TI (Flu N1 Vaccine*) OR AB (Flu N1 Vaccine*)

S52 TI (Influenza N1 Vaccine*) OR AB (Influenza N1 Vaccine*)

S51 (MH "Influenza Vaccine")

S50 TI (Vector N1 Expressing) OR AB (Vector N1 Expressing)

S49 TI mRNA-LNP OR AB mRNA-LNP

S48 TI mRNA* OR AB mRNA*

S47 TI BNT162b2 OR AB BNT162b2

S46 TI AZD1222 OR AB AZD1222

S45 TI V591 OR AB V591

S44 TI V591 OR AB V591

S43 TI (Measles N1 Vector) OR AB (Measles N1 Vector)

S42 TI ChAdOx1 OR AB ChAdOx1

S41 TI Adenovirus-5 OR AB Adenovirus-5

S40 TI Ad5* OR AB Ad5*

S39 TI Adenovirus-26 OR AB Adenovirus-26

S38 TI Adenovirus-26 OR AB Adenovirus-26

S37 TI Adenovirus-26 OR AB Adenovirus-26

S36 TI Adenovirus-26 OR AB Adenovirus-26

S35 TI Ad26* OR AB Ad26*

S34 TI (Replication N1 Incompetent) OR AB (Replication N1 Incompetent)

S33 TI (Full N1 Length) OR AB (Full N1 Length)

S32 TI (Molecular N1 Clamp) OR AB (Molecular N1 Clamp)

S31 TI S-Protein* OR AB S-Protein*

S30 TI (Stabilized N1 Spike) OR AB (Stabilized N1 Spike)

S29 TI Baculo* OR AB Baculo*

S28 TI (Recombinant N1 Spike-Protein) OR AB (Recombinant N1 Spike-Protein)

S27 TI CpG1 OR AB CpG1

S26 TI MF59 OR AB MF59

S25 TI AS03 OR AB AS03

S24 TI Aluminum* OR AB Aluminum*

S23 TI Alhydrogel OR AB Alhydrogel

S22 TI Matrix-M* OR AB Matrix-M*

S21 TI (Immunologic* N1 Adjuvant*) OR AB (Immunologic* N1 Adjuvant*)

S20 TI Immunoadjuvant* OR AB Immunoadjuvant*

S19 S1 OR S2 OR S3 OR S4 OR S5 OR S6 OR S7 OR S8 OR S9 OR S10 OR S11 OR S12 OR S13 OR S14 OR S15 OR S16 OR S17 OR S18

S18 TI DART OR AB DART

S17 TI Fetomaternal OR AB Fetomaternal

S16 TI (Materno N1 Fetal) OR AB (Materno N1 Fetal)

S15 TI Maternofetal OR AB Maternofetal

S14 TI Fetus OR AB Fetus

S13 TI Fetal OR AB Fetal

S12 (MH "Fetus+")

S11 TI Partum OR AB Partum

S10 TI Parturition* OR AB Parturition*

S9 TI Childbirth* OR AB Childbirth*

S8 (MH "Labor+")

S7 TI Gestational OR AB Gestational

S6 TI Miscarriage* OR AB Miscarriage*

S5 TI Abortion* OR AB Abortion*

S4 (MH "Abortion, Spontaneous+")

S3 (MH "Pregnancy Complications+")

S2 TI Pregnan* OR AB Pregnan*

S1 (MH "Pregnancy+")

**Global Health (OVID)**

1 exp Pregnancy/

2 Pregnan*.ti,ab.

3 exp Pregnancy Complications/

4 Abortion*.ti,ab.

5 Miscarriage*.ti,ab.

6 Gestational.ti,ab.

7 exp Parturition/

8 Childbirth*.ti,ab.

9 Parturition*.ti,ab.

10 Partum.ti,ab.

11 exp Fetus/

12 Fetal.ti,ab.

13 Fetus.ti,ab.

14 Maternofetal.ti,ab.

15 (Materno adj1 Fetal).ti,ab.

16 Fetomaternal.ti,ab.

17 DART.ti,ab.

18 or/1-17

19 Immunoadjuvant*.ti,ab.

20 (Immunologic* adj1 Adjuvant*).ti,ab.

21 Matrix-M*.mp.

22 Alhydrogel.mp.

23 Aluminum*.mp.

24 AS03.mp.

25 MF59.mp.

26 CpG1.mp.

27 (Recombinant adj1 Spike-Protein).mp.

28 Baculo*.mp.

29 (Stabilized adj1 Spike).mp.

30 S-Protein*.mp.

31 (Molecular adj1 Clamp).mp.

32 (Full adj1 Length).mp.

33 (Replication adj1 Incompetent).mp.

34 Ad26*.mp.

35 Adenovirus-26.mp.

36 Ad5*.mp.

37 Adenovirus-5.mp.

38 ChAdOx1.mp.

39 Measles-Vector.mp.

40 V591.mp.

41 AZD1222.mp.

42 BNT162b2.mp.

43 mRNA*.mp.

44 (Vector adj1 Expressing).mp.

45 (Influenza adj1 Vaccine*).mp.

46 (Flu adj1 Vaccine*).mp.

47 exp Saponins/

48 Saponin*.mp.

49 exp Nanoparticles/

50 Nanoparticle*.mp.

51 LNP.mp.

52 Nanocrystal*.mp.

53 Arepanrix.mp.

54 Pandemrix.mp.

55 FLUAD.mp.

56 Dynavax.mp.

57 (Hepatitis-B adj1 Vaccin*).mp.

58 HEPLISAV-B.mp.

59 (Polyethylene adj1 Glycol).mp.

60 or/19-59

61 exp Vaccination/

62 exp Vaccines/

63 Vaccin*.ti,ab.

64 or/61-63

65 18 and 60 and 64

66 Animal*.mp.

67 exp Nanoparticles/

68 Nanoparticle*.mp.

69 LNP.mp.

70 Nanocrystal*.mp.

71 or/67-70

72 exp Pregnancy/

73 exp Pregnancy/

74 Pregnan*.ti,ab.

75 DART.ti,ab.

76 or/72-74

77 66 and 71 and 76

78 65 or 77

## **Risk of bias assessment tools by study design**

**Criteria for judging risk of bias in the ‘Risk of bias’ assessment tool for RCTs**

| **RANDOM SEQUENCE GENERATION**  **Selection bias (biased allocation to interventions) due to inadequate generation of a randomized sequence** | |
| --- | --- |
| **Criteria for a judgement of ‘Low risk’ of bias.** | The investigators describe a random component in the sequence generation process such as:   - Referring to a random number table; - Using a computer random number generator; - Coin tossing; - Shuffling cards or envelopes; - Throwing dice; - Drawing of lots; - Minimization.*   **Minimization may be implemented without a random element, and this is considered to be equivalent to being random.* |
| **‘High risk’ of bias.** | The investigators describe a non-random component in the sequence generation process. Usually, the description would involve some systematic, non-random approach, for example:   - Sequence generated by odd or even date of birth; - Sequence generated by some rule based on date (or day) of admission; - Sequence generated by some rule based on hospital or clinic record number.   Other non-random approaches happen much less frequently than the systematic approaches mentioned above and tend to be obvious. They usually involve judgement or some method of non-random categorization of participants, for example:   - Allocation by judgement of the clinician; - Allocation by preference of the participant; - Allocation based on the results of a laboratory test or a series of tests; - Allocation by availability of the intervention. |
| **‘Unclear risk’ of bias.** | Insufficient information about the sequence generation process to permit judgement of ‘Low risk’ or ‘High risk’. |
| **ALLOCATION CONCEALMENT**  **Selection bias (biased allocation to interventions) due to inadequate concealment of allocations prior to assignment** | |
| ‘Low risk’ of bias. | Participants and investigators enrolling participants could not foresee assignment because one of the following, or an equivalent method, was used to conceal allocation:   - Central allocation (including telephone, web-based and pharmacy-controlled randomization); - Sequentially numbered drug containers of identical appearance; - Sequentially numbered, opaque, and sealed envelopes. |
| ‘High risk’ of bias. | Participants or investigators enrolling participants could possibly foresee assignments and thus introduce selection bias, such as allocation based on:   - Using an open random allocation schedule (e.g. a list of random numbers); - Assignment envelopes that were used without appropriate safeguards (e.g. if envelopes were unsealed, non-opaque, or not sequentially numbered); - Alternation or rotation; - Date of birth; - Case record number; - Any other explicitly unconcealed procedure. |
| ‘Unclear risk’ of bias. | Insufficient information to permit judgement of ‘Low risk’ or ‘High risk’. This is usually the case if the method of concealment is not described or not described in sufficient detail to allow a definite judgement – for example, if the use of assignment envelopes is described, but it remains unclear whether envelopes were sequentially numbered, opaque, and sealed. |
| **BLINDING OF PARTICIPANTS AND PERSONNEL**  **Performance bias due to knowledge of the allocated interventions by participants and personnel during the study** | |
| **‘Low risk’ of bias.** | Any one of the following:   - No blinding or incomplete blinding, but the review authors judge that the outcome is not likely to be influenced by lack of blinding; - Blinding of participants and key study personnel is ensured and it is unlikely that the blinding could have been broken. |
| **‘High risk’ of bias.** | Any one of the following:   - No blinding or incomplete blinding, and the outcome is likely to be influenced by lack of blinding; - Blinding of key study participants and personnel is attempted, but it is likely that the blinding could have been broken, and the outcome is likely to be influenced by lack of blinding. |
| **‘Unclear risk’ of bias.** | Any one of the following:   - Insufficient information to permit judgement of ‘Low risk’ or ‘High risk’; - The study did not address this outcome. |
| **BLINDING OF OUTCOME ASSESSMENT**  **Detection bias due to knowledge of the allocated interventions by outcome assessors** | |
| **‘Low risk’ of bias.** | Any one of the following:   - No blinding of outcome assessment, but the review authors judge that the outcome measurement is not likely to be influenced by lack of blinding; - Blinding of outcome assessment is ensured and it is unlikely that the blinding could have been broken. |
| **‘High risk’ of bias.** | Any one of the following:   - No blinding of outcome assessment, and the outcome measurement is likely to be influenced by lack of blinding; - Blinding of outcome assessment, but it is likely that the blinding could have been broken, and the outcome measurement is likely to be influenced by lack of blinding. |
| **‘Unclear risk’ of bias.** | Any one of the following:   - Insufficient information to permit judgement of ‘Low risk’ or ‘High risk’; - The study did not address this outcome. |
| **INCOMPLETE OUTCOME DATA**  **Attrition bias due to amount, nature or handling of incomplete outcome data** | |
| **‘Low risk’ of bias.** | Any one of the following:   - No missing outcome data; - Reasons for missing outcome data are unlikely to be related to true outcome (for survival data, censoring is unlikely to introduce bias); - Missing outcome data are balanced in numbers across intervention groups, with similar reasons for missing data across groups; - For dichotomous outcome data, the proportion of missing outcomes compared with observed event risk not enough to have a clinically relevant impact on the intervention effect estimate; - For continuous outcome data, plausible effect size (difference in means or standardized difference in means) among missing outcomes not enough to have a clinically relevant impact on observed effect size; - Missing data have been imputed using appropriate methods. |
| **‘High risk’ of bias.** | Any one of the following:   - Reason for missing outcome data is likely to be related to true outcome, with either imbalance in numbers or reasons for missing data across intervention groups; - For dichotomous outcome data, the proportion of missing outcomes compared with observed event risk enough to induce clinically relevant bias in intervention effect estimate; - For continuous outcome data, plausible effect size (difference in means or standardized difference in means) among missing outcomes enough to induce clinically relevant bias in observed effect size; - ‘As-treated’ analysis done with substantial departure of the intervention received from that assigned at randomization; - Potentially inappropriate application of simple imputation. |
| **‘Unclear risk’ of bias.** | Any one of the following:   - Insufficient reporting of attrition/exclusions to permit judgement of ‘Low risk’ or ‘High risk’ (e.g. number randomized not stated, no reasons for missing data provided); - The study did not address this outcome. |
| **SELECTIVE REPORTING**  **Reporting bias due to selective outcome reporting** | |
| **‘Low risk’ of bias.** | Any of the following:   - The study protocol is available and all of the study’s pre-specified (primary and secondary) outcomes that are of interest in the review have been reported in the pre-specified way; - The study protocol is not available but it is clear that the published reports include all expected outcomes, including those that were pre-specified (convincing text of this nature may be uncommon). |
| **‘High risk’ of bias.** | Any one of the following:   - Not all of the study’s pre-specified primary outcomes have been reported; - One or more primary outcomes is reported using measurements, analysis methods or subsets of the data (e.g. subscales) that were not pre-specified; - One or more reported primary outcomes were not pre-specified (unless clear justification for their reporting is provided, such as an unexpected adverse effect); - One or more outcomes of interest in the review are reported incompletely so that they cannot be entered in a meta-analysis; - The study report fails to include results for a key outcome that would be expected to have been reported for   such a study. |
| **‘Unclear risk’ of bias.** | - Insufficient information to permit judgement of ‘Low risk’ or ‘High risk’. It is likely that the majority of studies will fall into this category. |
| **OTHER BIAS**  **Bias due to problems not covered elsewhere in the table** | |
| **‘Low risk’ of bias.** | The study appears to be free of other sources of bias. |
| **‘High risk’ of bias.** | There is at least one important risk of bias. For example, the study:   - Had a potential source of bias related to the specific study design used; or - Has been claimed to have been fraudulent; or - Had some other problem. |
| **‘Unclear’ risk of bias.** | There may be a risk of bias, but there is either:   - Insufficient information to assess whether an important risk of bias exists; or - Insufficient rationale or evidence that an identified problem will introduce bias. |

**Criteria for judging risk of bias in quasi-experimental studies (Cochrane EPOC assessment tool**

**QUALITY CRITERIA FOR CONTROLLED BEFORE AND AFTER (CBA) DESIGNS**

Seven standard criteria are used for CBAs included in EPOC reviews:

**a) Baseline measurement:**

LOW RISK if performance or patient outcomes were measured prior to the intervention, and no substantial differences were present across study groups (e.g. where multiple pre-intervention measures describe similar trends in intervention and control groups);

UNCLEAR RISK if baseline measures are not reported, or if it is unclear whether baseline measures are substantially different across study groups;

HIGH RISK if there are differences at baseline in main outcome measures likely to undermine the post-intervention differences (e.g. are differences between the groups before the intervention similar to those found post-intervention).

**b) Characteristics for studies using second site as control:**

LOW RISK if characteristics of study and control providers are reported and similar;

UNCLEAR RISK if it is not clear in the paper e.g. characteristics are mentioned in the text but no data are presented;

HIGH RISK if there is no report of characteristics either in the text or a table OR if baseline characteristics are reported and there are differences between study and control providers.

**c) Blinded assessment of primary outcome(s)* (protection against detection bias):**

LOW RISK if the authors state explicitly that the primary outcome variables were assessed blindly OR the outcome variables are objective e.g., length of hospital stay, drug levels as assessed by a standardized test;

UNCLEAR RISK if not specified in the paper;

HIGH RISK if the outcomes were not assessed blindly.

** Primary outcome(s) are those variables that correspond to the primary hypothesis or question as defined by the authors. In the event that some of the primary outcome variables were assessed in a blind fashion and others were not, score each separately and label each outcome variable clearly.*

**d) Protection against contamination:**

*Studies using second site as control:*

LOW RISK if allocation was by community, institution, or practice and is unlikely that the control group received the intervention;

UNCLEAR RISK if providers were allocated within a clinic or practice and communication between experimental and group providers was likely to occur;

HIGH RISK if it is likely that the control group received the intervention (e.g., cross-over studies or if patients rather than providers were randomized).

**e) Reliable primary outcome measure(s):**

LOW RISK if two or more raters with at least 90% agreement or kappa greater than or equal to 0.8 OR the outcome is obtained from some automated system e.g., length of hospital stay, drug levels as assessed by a standardized test;

UNCLEAR RISK if reliability is not reported for outcome measures that are obtained by chart extraction or collected by an individual;

HIGH RISK if agreement is less than 90% or kappa is less than 0.8.

** In the event that some outcome variables were assessed in a reliable fashion and others were not, score each separately and label each outcome variable clearly.*

**f) Follow-up of professionals (protection against exclusion bias):**

LOW RISK if outcome measures obtained 80-100% subjects allocated to groups. (Do not assume 100% follow-up unless stated explicitly.);

UNCLEAR RISK if not specified in the paper;

HIGH RISK if outcome measures obtained for less than 80% of patients allocated to groups.

**g) Follow-up of patients:**

LOW RISK if outcome measures obtained 80-100% of patients allocated to groups or for patients who entered the study. (Do not assume 100% follow-up unless stated explicitly.);

UNCLEAR RISK if not specified in the paper;

HIGH RISK if outcome measures obtained for less than 80% of patients allocated to groups or for less than 80% of patients who entered the study.

**QUALITY CRITERIA FOR INTERRUPTED TIME SERIES (ITS)**

The following seven standard criteria should be used to assess the methodology quality of ITS designs included in EPOC reviews. Each criterion is scored DONE, NOT CLEAR or NOT DONE but here we use 'low risk', 'unclear risk', and 'high risk' respectively to be consistent with the 'Risk of bias' assessment tool for RCTs (Appendix 2.1).

*Protection against secular changes:*

**a) The intervention is independent of other changes.**

LOW RISK if the intervention occurred independently of other changes over time;

UNCLEAR RISK if not specified (will be treated as HIGH RISK if information cannot be obtained from the authors);

HIGH RISK if reported that intervention was not independent of other changes in time.

**b) Data were analyzed appropriately:**

LOW RISK if ARIMA models were used OR time series regression models were used to analyze the data and serial correlation was adjusted or tested for;

UNCLEAR RISK if not specified (will be treated as HIGH RISK if information cannot be obtained from the authors);

HIGH RISK if it is clear that neither of the conditions above not met.

**c) Reason for the number of points pre- and post-intervention given:**

LOW RISK if rationale for the number of points stated (e.g. monthly data for 12 months post-intervention was used because the anticipated effect was expected to decay) OR sample size calculation performed;

UNCLEAR RISK if not specified (will be treated as HIGH RISK if information cannot be obtained from the authors);

HIGH RISK if it is clear that neither of the conditions above met.

**d) Shape of the intervention effect was specified:**

LOW RISK if a rational explanation for the shape of intervention effect was given by the author(s);

UNCLEAR RISK if not specified (will be treated as HIGH RISK if information cannot be obtained from the authors);

HIGH RISK if it is clear that the condition above is not met.

*Protection against detection bias:*

**e) Intervention unlikely to affect data collection:**

LOW RISK if reported that intervention itself was unlikely to affect data collection (for example, sources and methods of data collection were the same before and after the intervention);

UNCLEAR RISK if not reported (will be treated as HIGH RISK if information cannot be obtained from the authors);

HIGH RISK if the intervention itself was likely to affect data collection (for example, any change in source or method of data collection reported).

**f) Blinded assessment of primary outcome(s)*:**

LOW RISK if the authors state explicitly that the primary outcome variables were assessed blindly OR the outcome variables are objective e.g., length of hospital stay, drug levels as assessed by a standardized test;

UNCLEAR RISK if not specified (will be treated as HIGH RISK if information cannot be obtained from the authors);

HIGH RISK if the outcomes were not assessed blindly.

** Primary outcome(s) are those variables that correspond to the primary hypothesis or question as defined by the authors. In the event that some of the primary outcome variables were assessed in a blind fashion and others were not, score each separately and label each outcome variable clearly.*

**g) Completeness of data set:**

LOW RISK if data set covers 80-100% of total number of participants or episodes of care in the study;

UNCLEAR RISK if not specified (will be treated as HIGH RISK if information cannot be obtained from the authors);

HIGH RISK if data set covers less than 80% of the total number of participants or episodes of care in the study.

**h) Reliable primary outcome measure(s)*:**

LOW RISK if two or more raters with at least 90% agreement or kappa greater than or equal to 0.8 OR the outcome is obtained from some automated system e.g., length of hospital stay, drug levels as assessed by a standardized test;

UNCLEAR RISK if reliability is not reported for outcome measures that are obtained by chart extraction or collected by an individual (will be treated as HIGH RISK if information cannot be obtained from the authors);

HIGH RISK if agreement is less than 90% or kappa is less than 0.8.

** In the event that some outcome variables were assessed in a reliable fashion and others were not, score each separately.*

**QUALITY CRITERIA FOR CONTROLLED INTERRUPTED TIME SERIES (CITS)**

**a) Protection against secular changes:**

The intervention is independent of other changes.

LOW RISK if the intervention occurred independently of other changes over time;

UNCLEAR RISK if not specified (will be treated as HIGH RISK if information cannot be obtained from the authors);

HIGH RISK if reported that intervention was not independent of other changes in time.

**b) Data were analyzed appropriately:**

LOW RISK if ARIMA models were used OR time series regression models were used to analyze the data and serial correlation was adjusted or tested for;

UNCLEAR RISK if not specified (will be treated as HIGH RISK if information cannot be obtained from the authors);

HIGH RISK if it is clear that neither of the conditions above not met.

**c) Reason for the number of points pre- and post-intervention given:**

LOW RISK if rationale for the number of points stated (e.g. monthly data for 12 months post-intervention was used because the anticipated effect was expected to decay) OR sample size calculation performed;

UNCLEAR RISK if not specified (will be treated as HIGH RISK if information cannot be obtained from the authors);

HIGH RISK if it is clear that neither of the conditions above met.

**d) Shape of the intervention effect was specified:**

LOW RISK if a rational explanation for the shape of intervention effect was given by the author(s);

UNCLEAR RISK if not specified (will be treated as HIGH RISK if information cannot be obtained from the authors);

HIGH RISK if it is clear that the condition above is not met.

*Intervention unlikely to affect data collection:*

**e) Protection against detection bias:**

LOW RISK if reported that intervention itself was unlikely to affect data collection (for example, sources and methods of data collection were the same before and after the intervention);

UNCLEAR RISK if not reported (will be treated as HIGH RISK if information cannot be obtained from the authors);

HIGH RISK if the intervention itself was likely to affect data collection (for example, any change in source or method of data collection reported).

**f) Blinded assessment of primary outcome(s)*:**

LOW RISK if the authors state explicitly that the primary outcome variables were assessed blindly OR the outcome variables are objective e.g., length of hospital stay, drug levels as assessed by a standardized test;

UNCLEAR RISK if not specified (will be treated as HIGH RISK if information cannot be obtained from the authors);

HIGH RISK if the outcomes were not assessed blindly.

* Primary outcome(s) are those variables that correspond to the primary hypothesis or question as defined by the authors. In the event that some of the primary outcome variables were assessed in a blind fashion and others were not, score each separately and label each outcome variable clearly.

**g) Completeness of data set:**

LOW RISK if data set covers 80-100% of total number of participants or episodes of care in the study;

UNCLEAR RISK if not specified (will be treated as HIGH RISK if information cannot be obtained from the authors);

HIGH RISK if data set covers less than 80% of the total number of participants or episodes of care in the study.

**h) Reliable primary outcome measure(s)*:**

LOW RISK if two or more raters with at least 90% agreement or kappa greater than or equal to 0.8 OR the outcome is obtained from some automated system e.g., length of hospital stay, drug levels as assessed by a standardized test;

UNCLEAR RISK if reliability is not reported for outcome measures that are obtained by chart extraction or collected by an individual (will be treated as HIGH RISK if information cannot be obtained from the authors);

HIGH RISK if agreement is less than 90% or kappa is less than 0.8.

* In the event that some outcome variables were assessed in a reliable fashion and others were not, score each separately.

For CITSs, as for CBAs, we will include three additional domains that assess design-specific threats to validity covered by the Cochrane EPOC group: imbalance of outcome measures at baseline; comparability of intervention and control group characteristics at baseline; and protection against contamination.

**i) Baseline measurement:**

LOW RISK if performance or patient outcomes were measured prior to the intervention, and no substantial differences were present across study groups (e.g. where multiple pre-intervention measures describe similar trends in intervention and control groups);

UNCLEAR RISK if baseline measures are not reported, or if it is unclear whether baseline measures are substantially different across study groups;

HIGH RISK if there are differences at baseline in main outcome measures likely to undermine the post-intervention differences (e.g. are differences between the groups before the intervention similar to those found post-intervention).

**j) Characteristics for studies using second site as control:**

LOW RISK if characteristics of study and control providers are reported and similar;

UNCLEAR RISK if it is not clear in the paper e.g. characteristics are mentioned in the text but no data are presented;

HIGH RISK if there is no report of characteristics either in the text or a table OR if baseline characteristics are reported and there are differences between study and control providers.

*Studies using second site as control:*

**k) Protection against contamination:**

LOW RISK if allocation was by community, institution, or practice and is unlikely that the control group received the intervention;

UNCLEAR RISK if providers were allocated within a clinic or practice and communication between experimental and group providers was likely to occur;

HIGH RISK if it is likely that the control group received the intervention (e.g., cross-over studies or if patients rather than providers were randomized).

**QUALITY CRITERIA FOR UNCONTROLLED BEFORE AND AFTER (UBA) DESIGNS**

Four standard criteria are used for UBAs (Derived from CBAs EPOC criteria):

**a) Blinded assessment of primary outcome(s)* (protection against detection bias):**

LOW RISK if the authors state explicitly that the primary outcome variables were assessed blindly OR the outcome variables are objective e.g., length of hospital stay, drug levels as assessed by a standardized test;

UNCLEAR RISK if not specified in the paper;

HIGH RISK if the outcomes were not assessed blindly.

** Primary outcome(s) are those variables that correspond to the primary hypothesis or question as defined by the authors. In the event that some of the primary outcome variables were assessed in a blind fashion and others were not, score each separately and label each outcome variable clearly.*

**b) Reliable primary outcome measure(s):**

LOW RISK if two or more raters with at least 90% agreement or kappa greater than or equal to 0.8 OR the outcome is obtained from some automated system e.g., length of hospital stay, drug levels as assessed by a standardized test;

UNCLEAR RISK if reliability is not reported for outcome measures that are obtained by chart extraction or collected by an individual;

HIGH RISK if agreement is less than 90% or kappa is less than 0.8.

** In the event that some outcome variables were assessed in a reliable fashion and others were not, score each separately and label each outcome variable clearly.*

**c) Follow-up of professionals (protection against exclusion bias):**

LOW RISK if outcome measures obtained 80-100% subjects at baseline. (Do not assume 100% follow-up unless stated explicitly.);

UNCLEAR RISK if not specified in the paper;

HIGH RISK if outcome measures obtained for less than 80% of patients at baseline.

**d) Follow-up of patients:**

LOW RISK if outcome measures obtained 80-100% of patients who entered the study. (Do not assume 100% follow-up unless stated explicitly.);

UNCLEAR RISK if not specified in the paper;

HIGH RISK if outcome measures obtained for less than 80% of patients who entered the study.

**NIH Quality Assessment Tool for observational studies**

<https://www.nhlbi.nih.gov/health-topics/study-quality-assessment-tools>

| **Criteria for cohort and cross-sectional studies** | **Judgement*** |
| --- | --- |
| 1. Was the research question or objective in this paper clearly stated? |  |
| 2. Was the study population clearly specified and defined? |  |
| 3. Was the participation rate of eligible persons at least 50%? |  |
| 4. Were all the subjects selected or recruited from the same or similar populations (including the same time period)? Were inclusion and exclusion criteria for being in the study prespecified and applied uniformly to all participants? |  |
| 5. Was a sample size justification, power description, or variance and effect estimates provided? |  |
| 6. For the analyses in this paper, were the exposure(s) of interest measured prior to the outcome(s) being measured? |  |
| 7. Was the timeframe sufficient so that one could reasonably expect to see an association between exposure and outcome if it existed? |  |
| 8. For exposures that can vary in amount or level, did the study examine different levels of the exposure as related to the outcome (e.g., categories of exposure, or exposure measured as continuous variable)? |  |
| 9. Were the exposure measures (independent variables) clearly defined, valid, reliable, and implemented consistently across all study participants? |  |
| 10. Was the exposure(s) assessed more than once over time? |  |
| 11. Were the outcome measures (dependent variables) clearly defined, valid, reliable, and implemented consistently across all study participants? |  |
| 12. Were the outcome assessors blinded to the exposure status of participants? |  |
| 13. Was loss to follow-up after baseline 20% or less? |  |
| 14. Were key potential confounding variables measured and adjusted statistically for their impact on the relationship between exposure(s) and outcome(s)? |  |

***Yes, No, CD, cannot determine; NA, not applicable; NR, not reported**

| **Criteria for cohort and case-control studies** | **Judgement*** |
| --- | --- |
| 1. Was the research question or objective in this paper clearly stated and appropriate? |  |
| 2. Was the study population clearly specified and defined? |  |
| 3. Did the authors include a sample size justification? |  |
| 4. Were controls selected or recruited from the same or similar population that gave rise to the cases (including the same timeframe)? |  |
| 5. Were the definitions, inclusion and exclusion criteria, algorithms or processes used to identify or select cases and controls valid, reliable, and implemented consistently across all study participants? |  |
| 6. Were the cases clearly defined and differentiated from controls? |  |
| 7. If less than 100 percent of eligible cases and/or controls were selected for the study, were the cases and/or controls randomly selected from those eligible? |  |
| 8. Was there use of concurrent controls? |  |
| 9. Were the investigators able to confirm that the exposure/risk occurred prior to the development of the condition or event that defined a participant as a case? |  |
| 10. Were the measures of exposure/risk clearly defined, valid, reliable, and implemented consistently (including the same time period) across all study participants? |  |
| 11. Were the assessors of exposure/risk blinded to the case or control status of participants? |  |
| 12. Were key potential confounding variables measured and adjusted statistically in the analyses? If matching was used, did the investigators account for matching during study analysis? |  |

***Yes, No, CD, cannot determine; NA, not applicable; NR, not reported**

| **Criteria for case-series studies** | **Judgement*** |
| --- | --- |
| 1. Was the study question or objective clearly stated? |  |
| 2. Was the study population clearly and fully described, including a case definition? |  |
| 3. Were the cases consecutive? |  |
| 4. Were the subjects comparable? |  |
| 5. Was the intervention clearly described? |  |
| 6. Were the outcome measures clearly defined, valid, reliable, and implemented consistently across all study participants? |  |
| 7. Was the length of follow-up adequate? |  |
| 8. Were the statistical methods well-described? |  |
| 9. Were the results well-described? |  |

***Yes, No, CD, cannot determine; NA, not applicable; NR, not reported**

# **Table 3. List and exclusion reasons of excluded studies**

| **Author** | **Title** | **Exclusion reason** |
| --- | --- | --- |
| Adeel 2021[[1](#_ENREF_1)] | Effectiveness of the SARS-CoV-2-mRNA Vaccines in Pregnant Women | Wrong outcome |
| Alguacil-Ramos 2015[[2](#_ENREF_2)] | [Safety of influenza vaccines in risk groups: analysis of adverse events following immunization reported in Valencian Community from 2005 to 2011] | Wrong exposure |
| Arriola 2017[[3](#_ENREF_3)] | Association of influenza vaccination during pregnancy with birth outcomes in Nicaragua | Wrong exposure |
| Beau 2014[[4](#_ENREF_4)] | Pandemic A/H1N1 influenza vaccination during pregnancy: a comparative study using the EFEMERIS database | Wrong exposure |
| Beharier 2021[[5](#_ENREF_5)] | Efficiente maternal to neonatal transfer of antibodies against SARS-CoV-2 and BNT162b2 mRNA COVID-19 vaccine | Wrong outcome |
| Burd 2021[[6](#_ENREF_6)] | The Israeli study of Pfizer BNT16n2 vaccine in pregnancy: Considering maternal and neonatal benefits | No results |
| Carcione 2013[[7](#_ENREF_7)] | Safety surveillance of influenza vaccine in pregnant women | Wrong exposure |
| Chambers 2013[[8](#_ENREF_8)] | Risks and safety of pandemic H1N1 influenza vaccine in pregnancy: birth defects, spontaneous abortion, preterm delivery, and small for gestational age infants | Wrong exposure |
| Chambers 2015[[9](#_ENREF_9)] | Safety of seasonal influenza vaccines in pregnancy: VAMPSS update | Wrong exposure |
| Chambers 2016[[10](#_ENREF_10)] | Safety of the 2010-11. 2011-12. 2012-13. and 2013-14 seasonal influenza vaccines in pregnancy: birth defects, spontaneous abortion, preterm delivery, and small for gestational age infants, a study from the cohort arm of VAMPSS | Wrong exposure |
| Choe 2011[[11](#_ENREF_11)] | Active surveillance of adverse events following immunization against pandemic influenza A (H1N1) in Korea | Wrong exposure |
| Conlin 2013[[12](#_ENREF_12)] | Safety of the pandemic H1N1 influenza vaccine among pregnant U.S. military women and their newborns | Wrong exposure |
| Covington 2019[[13](#_ENREF_13)] | PIN66 Vaccine pregnancy registries: methods and impact on findings | Wrong exposure |
| Cross 2020[[14](#_ENREF_14)] | Adverse events of interest vary by influenza vaccine type and brand: Sentinel network study of eight seasons (2010-2018) | Wrong patient population |
| Dodds 2011[[15](#_ENREF_15)] | Influenza vaccination in pregnancy | Wrong exposure |
| Donahue 2017[[16](#_ENREF_16)] | Association of spontaneous abortion with receipt of inactivated influenza vaccine containing H1N1pdm09 in 2010-11 and 2011-12 | Wrong exposure |
| Donahue 2019[[17](#_ENREF_17)] | Inactivated influenza vaccine and spontaneous abortion in the Vaccine Safety Datalink in 2012-13. 2013-14. and 2014-15 | Wrong exposure |
| Durrieu 2011[[18](#_ENREF_18)] | Safety surveillance of influenza A(H1N1)v monovalent vaccines during the 2009-2010 mass vaccination campaign in France | Wrong exposure |
| Eaton 2018[[19](#_ENREF_19)] | Birth outcomes following immunization of pregnant women with pandemic H1N1 influenza vaccine 2009-2010 | Wrong exposure |
| Golan 2021[[20](#_ENREF_20)] | COVID-19 mRNA Vaccination in Lactation: Assessment of adverse effects and transfer of anti-SARS-CoV2 antibodies from mother to child. | Wrong exposure |
| Goldman 2013[[21](#_ENREF_21)] | Comparison of VAERS fetal-loss reports during three consecutive influenza seasons: was there a synergistic fetal toxicity associated with the two-vaccine 2009/2010 season? | Wrong exposure |
| Kankawinpong 2012[[22](#_ENREF_22)] | Immunogenicity and safety of an inactivated pandemic H1N1 vaccine provided by the Thai ministry of public health as a routine public health service | Wrong exposure |
| Kharbanda 2013[[23](#_ENREF_23)] | Inactivated influenza vaccine during pregnancy and risks for adverse obstetric events | Wrong exposure |
| Kharbanda 2017[[24](#_ENREF_24)] | First trimester influenza vaccination and risks for major structural birth defects in offspring | Wrong exposure |
| Kozuki 2018[[25](#_ENREF_25)] | Impact of maternal vaccination timing and influenza virus circulation on birth outcomes in rural Nepal | Wrong exposure |
| Louik 2013[[26](#_ENREF_26)] | Risks and safety of pandemic H1N1 influenza vaccine in pregnancy: exposure prevalence, preterm delivery, and specific birth defects | Wrong exposure |
| Louik 2016[[27](#_ENREF_27)] | Safety of the 2011-12. 2012-13. and 2013-14 seasonal influenza vaccines in pregnancy: preterm delivery and specific malformations, a study from the case-control arm of VAMPSS | Wrong exposure |
| Lylianou 2012[[28](#_ENREF_28)] | Adverse events following immunization from pandemic influenza A (H1N1)-Laos 2010 | Wrong exposure |
| McHugh 2017[[29](#_ENREF_29)] | Birth outcomes for Australian mother-infant pairs who received an influenza vaccine during pregnancy, 2012-2014: the FluMum study | Wrong exposure |
| McHugh 2019[[30](#_ENREF_30)] | Influenza vaccination in pregnancy among a group of remote dwelling Aboriginal and Torres Strait Islander mothers in the Northern Territory: The 1+1 Healthy Start to Life study | Wrong exposure |
| MaciasSaint-Gerons 2021[[31](#_ENREF_31)] | Adverse events associated with the use of recommended vaccines during pregnancy: an overview of systematic reviews | Wrong study design |
| Mohammed 2020[[32](#_ENREF_32)] | Safety and protective effects of maternal influenza vaccination on pregnancy and birth outcomes: A prospective cohort study | Wrong exposure |
| Moro 2017[[33](#_ENREF_33)] | Surveillance of Adverse Events After Seasonal Influenza Vaccination in Pregnant Women and Their Infants in the Vaccine Adverse Event Reporting System, July 2010-May 2016 | Wrong exposure |
| Moro 2020[[34](#_ENREF_34)] | Monitoring the safety of high-dose, trivalent inactivated influenza vaccine in the vaccine adverse event reporting system (VAERS), 2011 - 2019 | Wrong exposure |
| Nordin 2013[[35](#_ENREF_35)] | Maternal safety of trivalent inactivated influenza vaccine in pregnant women | Wrong exposure |
| Ohfuji 2020[[36](#_ENREF_36)] | Safety of influenza vaccination on adverse birth outcomes among pregnant women: A prospective cohort study in Japan | Wrong exposure |
| Omer 2011[[37](#_ENREF_37)] | Maternal influenza immunization and reduced likelihood of prematurity and small for gestational age births: a retrospective cohort study | Wrong exposure |
| Panagiotakopoulos 2020[[38](#_ENREF_38)] | Evaluating the association of stillbirths after maternal vaccination in the vaccine safety datalink | Not enough information |
| Peppa 2020[[39](#_ENREF_39)] | Seasonal influenza vaccination during pregnancy and the risk of major congenital malformations in live-born infants: A 2010-2016 historical cohort study | Wrong exposure |
| Phengxay 2015[[40](#_ENREF_40)] | Introducing seasonal influenza vaccine in low-income countries: an adverse events following immunization survey in the Lao People's Democratic Republic | Wrong exposure |
| Ran 2021[[41](#_ENREF_41)] | Effectiveness of the BNT16b2 mRNA COVID-19 Vaccine in Pregnancy | Wrong outcome |
| Regan 2014[[42](#_ENREF_42)] | Using SMS to monitor adverse events following trivalent influenza vaccination in pregnant women | Wrong exposure |
| Regan 2016[[43](#_ENREF_43)] | Seasonal trivalent influenza vaccination during pregnancy and the incidence of stillbirth: population-based retrospective cohort study | Wrong exposure |
| Regan 2018[[44](#_ENREF_44)] | Birth outcomes associated with seasonal influenza vaccination during first trimester of pregnancy | Wrong exposure |
| Richner 2017[[45](#_ENREF_45)] | Vaccine mediated protection against Zika virus-induced congenital disease | Wrong outcomes |
| Rottenstreich 2021[[46](#_ENREF_46)] | Efficient maternofetal transplacental transfer of anti-SARS-CoV-2 spike antibodies after antenatal SARS-CoV-2 BNT162b2mRNA vaccination | Wrong outcome |
| Shatla 2016[[47](#_ENREF_47)] | Effect of maternal antenatal influenza vaccination on adverse neonatal outcomes in terms of premature birth, small-for-gestational age and low birth weight: a comparative study | Wrong exposure |
| Vazquez-Benitez 2016[[48](#_ENREF_48)] | Risk of preterm or small-for-gestational-age birth after influenza vaccination during pregnancy: caveats when conducting retrospective observational studies | Wrong exposure |
| Walsh 2019[[49](#_ENREF_49)] | Health outcomes of young children born to mothers who received 2009 pandemic H1N1 influenza vaccination during pregnancy: retrospective cohort study | Wrong exposure |
| Wijnans 2017[[50](#_ENREF_50)] | Bell's palsy and influenza(H1N1)pdm09 containing vaccines: a self-controlled case series | Wrong patient population |
| Zdanowski 2021[[51](#_ENREF_51)] | Evaluation of SARS-CoV-2 Spike Protein Antibody Titers in Cord Blood after COVID-19 Vaccination during Pregnancy in Polish Healthcare Workers: Preliminary Results | Wrong outcome |

# **Table 4. Characteristics of included studies for non-COVID-19 vaccines**

| **ID** | **Country** | **Year** | **Type of surveillance** | **Trimes-**  **ter** | **Vaccine** | **Population** | N  **(Ex/No Ex)** | **Study design** | **Effect measures** | **Outcomes** | **Safety concerns** |
| --- | --- | --- | --- | --- | --- | --- | --- | --- | --- | --- | --- |
| **Pregnant persons** | | | | | | | | | | | |
| *Vaccines containing AS03 adjuvant* | | | | | | | | | | | |
| **Guo 2010**^[^[^52^](#_ENREF_52)^]^ | Canada | 2009-10 | Active | NA | H1N1 | Pregnant p | 875 (550/325) | Cohort study | IR | Mat, Neo | No |
| **Tavares 2011**^[^[^53^](#_ENREF_53)^]^ | UK-Belgium | 2009 | Active | 1+2+3 | H1N1 | Pregnant p | 267 | Cohort study | IR | Mat, Neo, AEFI | No |
| **Baum 2015**^[^[^54^](#_ENREF_54)^]^ | Finland | 2009-10 | Active | 2+3 | H1N1 | Pregnant p | 43,604 (34,241/9,363) | Cohort study | IR, HR | Mat, Neo | No |
| **Chavant 2013**^[^[^55^](#_ENREF_55)^]^ | France | 2009-10 | Active | 1+2+3 | H1N1 (Pandemrix) | Pregnant p | 56 | Surveillance | IR | Mat, Neo, AEFI | No |
| **Fell 2012**^[^[^56^](#_ENREF_56)^]^ | Canada | 2009-10 | Active | 2+3 | H1N1 & seasonal flu | Pregnant p | 55,570  (23,340/32,230) | Registry | IR, aRR | Mat, Neo. | No |
| **GalindoSantana 2011**^[^[^57^](#_ENREF_57)^]^ | Cuba | 2009-10 | Active | NA | H1N1 (Pandemrix) | Pregnant p | 80,317 | Cohort study | IR | Mat, AEFI | No |
| **Källén 2012**^[^[^58^](#_ENREF_58)^]^ | Sweden | 2009-10 | Active | 1+2+3 | H1N1 (Pandemrix) | Pregnant p | 155,526  (18,612/136,914) | Cohort study | IR, OR | Mat, Neo | No |
| **Lacroix 2010**^[^[^59^](#_ENREF_59)^]^ | France | 2009 | Active | 1+2+3 | H1N1 | Pregnant p | NA | Surveillance | IR | Mat, Neo | No |
| **Layton 2011**^[^[^60^](#_ENREF_60)^]^ | UK | 2010 | Active | 1+2+3 | H1N1 (Pandemrix) | Pregnant p | 92 (92/38) | Cohort study | IR | Mat, Neo | No |
| **Levi 2012**^[^[^61^](#_ENREF_61)^]^ | Denmark | 2012 | Active | 1+2+3 | H1N1 (Pandemrix) | Pregnant p | 13,924 (6,962/6,962) | Cohort study | IR | Mat, Neo | No |
| **Ludvigsson 2013**^[^[^62^](#_ENREF_62)^]^ | Sweden | 2009-10 | Active | 1+2+3 | H1N1 (Pandemrix) | Pregnant p | 21,087 (13,297/7,790) | Cohort study | IR, aOR | Neo | No |
| **Ludvigsson 2016**^[^[^63^](#_ENREF_63)^]^ | Sweden | 2009-11 | Active | 1 | H1N1 (Pandemrix) | Pregnant p | 11,480 (2,037/9,443) | Cohort study | aOR | Neo | No |
| **Mackenzie 2012**^[^[^64^](#_ENREF_64)^]^ | UK | 2009-10 | Active | 1+2+3 | H1N1 (Pandemrix) | Pregnant p | 143 (130/13) | Cohort study | IR | Mat, Neo | No |
| **NúñezRojas 2010**^[^[^65^](#_ENREF_65)^]^ | Cuba | 2010 | Active | 1 | H1N1 (Pandemrix) | Pregnant p | 656 (451/205) | Cohort study | IR | Neo, AEFI | No |
| **Oppermann 2012**^[^[^66^](#_ENREF_66)^]^ | Germany | 2009-10 | Active | 1 | H1N1 (Pandemrix) | Pregnant p | 306 (90/216) | Cohort study | IR | Mat, Neo | No |
| **Pasternak 2012**[[67](#_ENREF_67), [68](#_ENREF_68)] | Denmark | 2009-10 | Active | 1+2+3 | H1N1 (Pandemrix) | Pregnant p | 36,546 (7,062/47,523) | Cohort study | aHR, IR,POR | Mat, Neo | No |
| **Ray 2014**^[^[^69^](#_ENREF_69)^]^ | Canada | 2009 | Active | 1+2+3 | H1N1 (Pandemrix) | Pregnant p | 708 (509/199) | Cohort study | aOR | Mat, Neo | No |
| **Sammon 2012**^[^[^70^](#_ENREF_70)^]^ | UK | 2009-10 | Active | 1+2+3 | H1N1 (Pandemrix) | Pregnant p | 9,026 (9,445/30,418) | Cohort study | IR, aHR | Neo | No |
| **Folkenberg 2011**^[^[^71^](#_ENREF_71)^]^ | Denmark | 2009-10 | Passive | NA | H1N1 (Pandemrix) | Pregnant p | 5,772 | Surveillance | IR | Mat, Neo | No |
| **Haberg 2013**^[^[^72^](#_ENREF_72)^]^ | Norway | 2009-10 | Passive | 2+3 | H1N1 (Pandemrix) | Pregnant p | 13,435,383  (3,020,750/10,414,633) | Registry | aHR | Mat, Neo | No |
| **Läkemedelsverket 2010**^[^[^73^](#_ENREF_73)^]^ | Sweden | 2010 | Passive | 1+2+3 | H1N1 (Pandemrix) | Pregnant p | > 30,000 | Surveillance | IR | Mat, Neo | No |
| **Ludvigsson 2015**^[^[^74^](#_ENREF_74)^]^ | Sweden | 2009-10 | Passive | 1+2+3 | H1N1 (Pandemrix) | Pregnant p. | 275,500  (41,138/234,317) | Cohort study | aHR | Neo | No |
| **Sammon 2011**^[^[^75^](#_ENREF_75)^]^ | UK | 2010 | Passive | 1+2+3 | H1N1 (Pandemrix) | Pregnant p | 36,438 | Cohort study | IR, RR | Mat | ? |
| *Vaccines containing Aluminum adjuvant* | | | | | | | | | | | |
| **Groom 2019**^[^[^76^](#_ENREF_76)^]^ | USA | 2004-15 | Active | 1+2+3 | Hepatitis A | Pregnant p | 653,826  (1,140/652,686) | Cohort study | IR, aOR | Mat, Neo. | No^#^ |
| **Hoang 2016**[[77](#_ENREF_77)] | Vietnam | 2015 | Active | 2+3 | Tdap | Pregnant p | 103 (52/51) | RCT | IR | Neo, AEFI | No |
| **Katz 2016** ^[^[^78^](#_ENREF_78)^]^ | Argentina | 2011-15 | Active | 2+3 | Tdap | Pregnant p | 1,845,379 | Registry | IR | AEFI | No |
| **Madhi 2020**^[^[^79^](#_ENREF_79)^]^ | Multicountry* | 2015-18 | Active | 3 | RSV-F | Pregnant p | 4,626 (3,045/1,581) | RCT | IR | Neo, AEFI | No |
| **Maertens 2016**^[^[^80^](#_ENREF_80)^]^ | Belgium | 2016 | Active | 2+3 | Tdap | Pregnant p | 98 (57/41) | Cohort study | IR | Mat, AEFI | No |
| **McHugh 2019**^[^[^81^](#_ENREF_81)^]^ | Australia | 2012-15 | Active | 1+2+3 | Pertussis | Pregnant p | 8,827 (2,706/6,121) | Cohort study | HR | Neo. | No |
| **Mohamed 2021**[[82](#_ENREF_82)] | Australia | 2015-17 | Active | 1+2+3 | Tdap | Pregnant p | 1,272 (1,019/253) | Cohort study | IR, aHR, aRR | Mat, Neo. | No |
| **Swamy 2020**[[83](#_ENREF_83)] | USA | 2014-15 | Active | 2+3 | Trivalent Group B Streptococcus | Pregnant p | 75 (49/26) | RCT | IR | Mat, Neo, AEFI | No |
| **Zheteyeva 2012[**[**84**](#_ENREF_84)**]** | USA | 2005-10 | Active | 1+2+3 | Tdap | Pregnant p | 180 (48/132) | Registry | IR | Mat, Neo, AEFI | No |
| **Muñoz 2014**[[85](#_ENREF_85)] | USA | 2008-12 | Active | 3 | Tdap | Pregnant p | 48 (33/15) | RCT | IR | AEFI | No |
| **Kushner 2020**^[^[^86^](#_ENREF_86)^]^ | USA | 2019 | Active | 1+2+3 | Hepatitis B (Engerix-B) | Pregnant p | 59 (40/19) | Cohort study | IR | Mat, Neo. | No |
| **Rega 2016**^[^[^87^](#_ENREF_87)^]^ | Australia | 2012-15 | Active | NA | Influenza vaccine | Pregnant p | 4,347 | Surveillance | IR, aHR | Mat, Neo. | No |
| **Muñoz 2019**^[^[^88^](#_ENREF_88)^]^ | USA | 2014-15 | Active | 3 | RSV-F | Pregnant p | 50 (22/28) | RCT | IR | Mat, Neo, AEFI | No |
| **Perrett 2020**[[89](#_ENREF_89)] | Multicountry* | 2015-17 | Active | 3 | Tdap | Pregnant p | 687 (341/346) | RCT | IR | Mat, Neo, AEFI | No |
| **Petousis-Harris 2016**[[90](#_ENREF_90)] | New Zealand | 2012-14 | Active | 3 | Tdap | Pregnant p | 793 | Cohort study | IR | Mat, Neo, AEFI | No |
| **Regan 2016**[[43](#_ENREF_43)] | Australia | 2012-15 | Active | 3 | Tdap | Pregnant p | 2,841 (1,257/1,584) | Cohort study | IR, OR | AEFI | No |
| **Fortner 2016**[[91](#_ENREF_91)] | USA | 2016 | Active | 2+3 | Tdap | Pregnant p | 522 (361/161) | Cohort study | IR | AEFI | No |
| **Angelo 2014**[[92](#_ENREF_92)] | Belgium | 2013 | Passive | 1+2+3 | HPV 16/18 (Cervarix) | Pregnant p | 146 | Surveillance | IR | Mat | No |
| **Angelo 2014**[[93](#_ENREF_93)] | Belgium | 2007-11 | Passive | 1+2+3 | HPV 16, 18  (Cervarix) | Pregnant p | 160 | Surveillance | IR | Mat, Neo | No |
| **Baril 2015**[[94](#_ENREF_94)] | UK | 2008-11 | Passive | 1 | HPV 16, 18  (Cervarix) | Pregnant p | 962 (330/632) | Cohort study | IR | Mat, Neo | No |
| **Berenson 2016**^[^[^95^](#_ENREF_95)^]^ | USA | 2012-14 | Passive | 3 | Tdap | Pregnant p | 1,759 (650/1,109) | Cross-sectional | IR, aOR | Neo | No |
| **Celzo 2020**^[^[^96^](#_ENREF_96)^]^ | Belgium | 2018 | Passive | 1+2+3 | Hepatitis B (Engerix-B); A&B (Twinrix); A (Havrix) | Pregnant p | 339 | Surveillance | IR | Mat, Neo | No |
| **Dana 2009**[[97](#_ENREF_97)] | USA, Canada, and France | 2006-08 | Passive | 1+2+3 | HPV 6/11/16/18 | Pregnant p | 593 | Registry | IR | Mat, Neo | No |
| **Donegan 2014**^[^[^98^](#_ENREF_98)^]^ | UK | 2010-12 | Passive | 3 | Tdap | Pregnant p | 24,708 (6,185/18,523) | Cohort study | IR, IRR | Mat, Neo | No |
| **Groom 2018**^[^[^99^](#_ENREF_99)^]^ | USA | 2004-15 | Passive | 1+2+3 | Hepatitis B (Engerix-B, Recombivax) A&B (Twinrix) | Pregnant p | 653,826  (1,399/652,432) | Cohort study | IR, aOR | Mat, Neo | No |
| **Kharbanda 2014**^[^[^100^](#_ENREF_100)^]^ | USA | 2010-12 | Passive | 1+2+3 | Tdap | Pregnant p | 123,494  (26,229/97,265) | Cohort study | IR, aHR, aRR | Mat, Neo | No |
| **Kharbanda 2016**^[^[^101^](#_ENREF_101)^]^ | USA | 2007-13 | Passive | 1+2+3 | Tdap | Pregnant p | 163,138 (53,885/109,253) | Cohort study | IR, aRR | Mat, AEFI | No |
| **Morgan 2015** ^[^[^102^](#_ENREF_102)^]^ | USA | 2013-14 | Passive | 3 | Tdap | Pregnant p | 7,378 | Cohort study | IR | Neo | No |
| **Moro 2018**^[^[^103^](#_ENREF_103)^]^ | USA | 1990-16 | Passive | 1+2+3 | Hepatitis A (VAQTA, Havrix), A&B (Twinrix) | Pregnant p | 192 | Surveillance | IR | Mat, Neo, AEFI | No |
| **Moro 2018**^[^[^102^](#_ENREF_102)^]^ | USA | 1996-13 | Passive | 1+2+3 |  | Pregnant p | 139 | Surveillance | IR | Mat, Neo | No |
| **Shakib 2013**^[^[^104^](#_ENREF_104)^]^ | USA | 2005-09 | Passive | 1+2+3 | Tdap | Pregnant p | 690 (138/552) | Cohort study | IR | Mat, Neo | No |
| *Vaccines containing* *CpG adjuvant* | | | | | | | | | | | |
| **Kushner 2020**^[^[^86^](#_ENREF_86)^]^ | USA | 2019 | Active | 1+2+3 | Hepatitis B (HEPLISAV-B) | Pregnant p | 59 (40/19) | Cohort study | IR | Mat, Neo | No |
| **Pregnant animals** | | | | | | | | | | | |
| *Vaccines containing Aluminum adjuvant* | | | | | | | | | | | |
| **Bielefeldt-Ohmann 2014**^[^[^105^](#_ENREF_105)^]^ | Australia | 2011 | NA | NA | Japanese Encephalitis (JE-ADVAX) | Animals | 25 (19/6) | Cohort study | IR | Mat, Neo, AEFI | No |
| **Warda 2021**^[^[^106^](#_ENREF_106)^]^ | Egypt | 2019 | NA | NA | Equine Herpesvirus (EHV) | Animals | 15 | Cohort study | IR | Neo | No |
| **Wise 2008**^[^[^107^](#_ENREF_107)^]^ | USA | 2008 | NA | NA | HPV 6/11/16/18 | Animals | 42 (22/20) | RCT | IR | Neo | No |
| **Segal 201**^[^[^108^](#_ENREF_108)^]^ | UK | 2010 | NA | NA | HPV 16, 18  (Cervarix) | Animals | 112 (56/56) | RCT | IR | Neo | No |
| **Glenn 2015**^[^[^109^](#_ENREF_109)^]^ | USA | 2015 | NA | NA | RSV-F | Animals | 20 (10/10) | RCT | IR | Mat, Neo | No |
| *Vaccines containing Matrix-M adjuvant* | | | | | | | | | | | |
| **Heldens 2009**^[^[^110^](#_ENREF_110)^]^ | The Netherlands | 2009 | Active | NA | Equine influenza - Equillis Prequenza T | Animals | 11 (10/1) | Non RCT | IR | AEFI | No |
| *Vaccines using RNA platform* | | | | | | | | | | | |
| **Shan 2019**^[^[^111^](#_ENREF_111)^]^ | USA | 2019 | Active | NA | ZIKA vaccine | Animals | 10 (5/5) | RCT | IR | Mat, Neo | No |
| *Vaccines using viral vector platform* | | | | | | | | | | | |
| **Stedman 2019**^[^[^112^](#_ENREF_112)^]^ | The Netherlands | 2019 | Active | NA | ChAdOx1 Rift Valley fever virus | Animals | 16 (8/8) | RCT | IR | Mat | No |

**NA**: Not available; **HPV**: Human Papillomavirus vaccine; **Tdap**: Tetanus-Diphtheria-Pertussis; **RSV-F**: Respiratory Syncytial Virus – Fusion; **Pregnant p**: Pregnant person; **IR**: Incidence rate; **aHR**: adjusted Hazard Ratio; **aRR**: adjusted Risk Ratio; **aOR**: adjusted Odds Ratio; **POR**: adjusted Prevalence Odds Ratio **Mat**: Maternal; **Neo**: Neonatal; **AEFI**: Adverse Event Following Immunization; ?: Yes, but probably due to residual confounding; **#** Association between maternal Hepatitis A vaccination and small-for-gestational-age; **RCT**: Randomized Controlled Trial; **Ex/NoEx:** Exposed/No exposed in comparative studies.

*Argentina, Australia, Chile, Bangladesh, Mexico, New Zealand, the Philippines, South Africa, Spain, the United Kingdom, and USA; **Australia, Canada, Czech Republic, Finland, Italy and Spain.

# **Table 5. Risk of bias assessments for COVID-19 vaccines**

**5.1 Risk of bias of observational studies**

| **Study ID** | **Study design** | **Signaling questions*** | | | | | | | | | | | | | | **Global Quality** |
| --- | --- | --- | --- | --- | --- | --- | --- | --- | --- | --- | --- | --- | --- | --- | --- | --- |
|  |  | **1** | **2** | **3** | **4** | **5** | **6** | **7** | **8** | **9** | **10** | **11** | **12** | **13** | **14** |  |
| Gray 2021 | Cohort studies | Yes | Yes | Yes | Yes | No | Yes | Yes | NA | Yes | NA | Yes | NR | Yes | No | **Fair** |
| Bookstein Peretz 2021 | Surveillance | Yes | Yes | Yes | Yes | No | Yes | Yes | NA | Yes | Yes | Yes | No | NR | Yes | **Good** |
| Kharbanda 2014 | Cohort studies | Yes | Yes | Yes | No | No | Yes | Yes | Yes | Yes | NA | Yes | NA | NA | Yes | **Good** |
| Goldstein 2021 | Cohort studies | Yes | Yes | Yes | Yes | No | Yes | Yes | NA | Yes | No | Yes | No | No | Yes | **Good** |
| Shimabukuro 2021 | Cohort studies | Yes | Yes | No | Yes | No | Yes | Yes | NA | Yes | Yes | Yes | No | NA | No | **Fair** |
| Zauche 2021 | Registry analysis | Yes | Yes | Yes | Yes | No | Yes | Yes | NA | Yes | Yes | Yes | No | NR | No | **Fair** |
| Blakeway 2021 | Cohort studies | Yes | Yes | No | Yes | No | Yes | Yes | NA | Yes | No | Yes | No | No | Yes | **Good** |

***Signaling questions**

1. Was the research question or objective clearly stated in this study?

2. Was the study population clearly specified and defined?

3. Was the participation of eligible persons at least 50%?

4. Were all subjects selected or recruited from the same or similar populations (including the same time frame)? Were the inclusion and exclusion criteria pre-specified and applied to participate in the study of uniformly to all participants?

5. Was a justification of the sample size, a description of the power, or estimates of variance provided and effect?

6. For the analysis in this study, were the exposure(s) of interest measured before the outcome(s) were measured?

7. Was the follow-up period long enough for one to reasonably expect to see an association between exposure and result if it exists?

8. For exposures that can vary in quantity or level, did the study examine different levels of exposure in relation to with the outcome (for example, exposure categories or exposure measured as a continuous variable)?

9. Were the exposure measures (independent variables) clearly defined, valid, reliable and implemented consistently across all study participants?

10. Were the exposure(s) evaluated more than once over time?

11. Were the outcome measures (dependent variables) clearly defined, valid, reliable and implemented consistently across all study participants?

12. Were the outcome assessors blinded to the exposure status of the participants?

13. Was the loss to follow-up after the start of the study 20% or less?

14. Were potential key confounding variables statistically measured and adjusted for their impact on the relationship between exposure(s) and outcome(s)?

**5.2 Risk of bias of clinical trials**

| **Study ID** | **Adequate sequence generation** | **Allocation concealment** | **Blinding of participant & personnel** | **Blinding of outcome assessment** | **Incomplete outcome data** | **Selective reporting** | **Other sources of bias** |
| --- | --- | --- | --- | --- | --- | --- | --- |
| Bowman 2021 | Unclear | Low | Low | Low | Unclear | Low | Low |
| Stebbings 2021 | Unclear | Unclear | Unclear | Unclear | Low | Low | Low |

# **Table 6. Risk of bias assessments for non-COVID-19 vaccines**

**6.1 Risk of bias of observational studies**

| **Study ID** | **Study design** | **Signaling questions*** | | | | | | | | | | | | | | **Global Quality** |
| --- | --- | --- | --- | --- | --- | --- | --- | --- | --- | --- | --- | --- | --- | --- | --- | --- |
|  |  | **1** | **2** | **3** | **4** | **5** | **6** | **7** | **8** | **9** | **10** | **11** | **12** | **13** | **14** |  |
| Baum 2015 | Cohort studies | Yes | Yes | Yes | Yes | No | Yes | Yes | NA | Yes | No | Yes | No | Yes | Yes | **Good** |
| Galindo Santana 2011 | Cohort studies | Yes | Yes | Yes | Yes | No | Yes | Yes | NA | Yes | No | Yes | No | NR | No | **Fair** |
| Gray 2021 | Cohort studies | Yes | Yes | Yes | Yes | No | Yes | Yes | NA | Yes | NA | Yes | NR | Yes | No | **Fair** |
| Groom 2018 | Cohort studies | Yes | Yes | Yes | Yes | No | Yes | Yes | NA | Yes | NA | Yes | NR | Yes | Yes | **Good** |
| Guo 2010 | Cohort studies | Yes | CD | CD | CD | CD | Yes | Yes | NA | CD | NA | CD | NR | Yes | No | **Fair** |
| Jonas 2015 | Cohort studies | Yes | Yes | Yes | Yes | NR | Yes | Yes | NA | Yes | NA | Yes | NR | Yes | Yes | **Good** |
| Källén 2012 | Cohort studies | Yes | Yes | Yes | Yes | NR | Yes | Yes | NA | Yes | NA | Yes | NR | Yes | NR | **Fair** |
| Kushner 2020 | Cohort studies | Yes | Yes | Yes | NR | No | Yes | Yes | No | Yes | Yes | Yes | NR | NR | No | **Fair** |
| Layton 2011 | Cohort studies | Yes | CD | CD | CD | CD | Yes | CD | NA | CD | NA | CD | CD | NR | No | **Fair** |
| Levi 2012 | Cohort studies | Yes | Yes | CD | CD | CD | Yes | Yes | NA | CD | NA | CD | CD | NR | CD | **Fair** |
| Ludvigsson 2013 | Cohort studies | Yes | Yes | Yes | Yes | NR | Yes | Yes | NA | Yes | NA | Yes | NR | No | Yes | **Good** |
| Ludvigsson 2016 | Cohort studies | Yes | Yes | Yes | Yes | No | Yes | Yes | NA | Yes | NA | Yes | NR | Yes | Yes | **Good** |
| Mackenzie 2012 | Cohort studies | Yes | Yes | Yes | Yes | NR | Yes | Yes | NA | Yes | No | Yes | NR | Yes | No | **Fair** |
| McHugh 2019 | Cohort studies | Yes | Yes | Yes | Yes | No | Yes | Yes | NA | Yes | NA | Yes | NR | Yes | Yes | **Good** |
| Núñez Rojas 2010 | Cohort studies | Yes | Yes | NR | No | No | Yes | Yes | NA | Yes | NA | No | NR | NR | No | **Poor** |
| Oppermann 2012 | Cohort studies | Yes | Yes | Yes | Yes | Yes | Yes | Yes | NA | Yes | NA | Yes | No | Yes | Yes | **Good** |
| Pasternak 2012 | Cohort studies | Yes | Yes | Yes | Yes | NR | Yes | Yes | NA | Yes | No | Yes | NR | Yes | Yes | **Good** |
| Ray 2014 | Cohort studies | No | No | CD | Yes | NR | Yes | CD | NA | No | No | No | NR | CD | NR | **Poor** |
| Sammon 2011 | Cohort studies | Yes | Yes | Yes | Yes | No | Yes | CD | NA | Yes | NA | No | NA | NA | No | **Poor** |
| Sammon 2012 | Cohort studies | Yes | Yes | Yes | Yes | No | Yes | Yes | NA | Yes | No | Yes | No | NR | Yes | **Good** |
| Tavares 2011 | Cohort studies | Yes | Yes | CD | Yes | No | Yes | Yes | NA | Yes | No | Yes | No | No | No | **Fair** |
| Fell 2012 | Registry analysis | Yes | Yes | Yes | Yes | No | Yes | Yes | NA | Yes | NA | Yes | NA | Yes | Yes | **Good** |
| Haberg 2013 | Registry analysis | Yes | Yes | Yes | Yes | No | Yes | Yes | NA | Yes | NA | Yes | NA | No | Yes | **Good** |
| Katz 2016 | Registry analysis | Yes | Yes | CD | Yes | No | Yes | CD | NA | Yes | NA | Yes | NA | NA | NA | **Fair** |
| Celzo 2020 | Surveillance | Yes | Yes | CD | No | No | Yes | NR | NR | Yes | NA | Yes | NA | NA | No | **Fair** |
| Chavant 2013 | Surveillance | Yes | Yes | NA | Yes | No | Yes | Yes | Yes | Yes | No | Yes | No | CD | No | **Poor** |
| Folkenberg 2011 | Surveillance | Yes | Yes | CD | Yes | No | Yes | Yes | NA | Yes | NA | Yes | NA | NA | NA | **Fair** |
| Lacroix 2010 | Surveillance | Yes | Yes | CD | Yes | No | No | Yes | NA | Yes | No | Yes | NA | NA | No | **Fair** |
| Läkemedelsverket 2010 | Surveillance | Yes | Yes | CD | CD | No | Yes | Yes | NA | Yes | NA | No | No | NA | No | **Poor** |
| Moro 2014 | Surveillance | Yes | Yes | CD | Yes | No | Yes | Yes | NA | Yes | NA | Yes | NA | NA | NA | **Fair** |
| Moro 2018 | Surveillance | Yes | Yes | NA | Yes | No | Yes | Yes | NA | No | No | Yes | NA | NA | NA | **Poor** |
| Rega 2016 | Surveillance | No | No | Yes | NR | No | Yes | NR | NA | Yes | NA | NR | NA | NA | No | **Poor** |
| Bookstein Peretz 2021 | Surveillance | Yes | Yes | Yes | Yes | No | Yes | Yes | NA | Yes | Yes | Yes | No | NR | Yes | **Good** |
| Groom 2019 | Cohort studies | Yes | Yes | Yes | Yes | No | Yes | Yes | NA | Yes | NA | Yes | NA | Yes | Yes | **Good** |
| Berenson 2016 | Cross-sectional | Yes | No | Yes | No | No | Yes | Yes | No | Yes | NA | Yes | No | NA | Yes | **Fair** |
| Kharbanda 2014 | Cohort studies | Yes | Yes | Yes | No | No | Yes | Yes | Yes | Yes | NA | Yes | NA | NA | Yes | **Good** |
| Mohamed 2021 | Cohort studies | Yes | Yes | Yes | No | No | Yes | Yes | Yes | Yes | NA | Yes | NA | NA | Yes | **Good** |
| Angelo 2014 | Surveillance | Yes | Yes | NA | No | No | Yes | NR | NA | Yes | NA | Yes | No | NA | No | **Fair** |
| Angelo 2014 | Surveillance | Yes | Yes | NA | No | No | Yes | NR | NA | Yes | NA | Yes | No | NA | No | **Fair** |
| Baril 2015 | Cohort studies | Yes | Yes | Yes | Yes | Yes | Yes | Yes | NA | Yes | NA | Yes | No | Yes | Yes | **Good** |
| Bielefeldt-Ohmann 2014 | Cohort studies | Yes | Yes | NA | Yes | No | Yes | Yes | NA | Yes | NA | Yes | No | No | No | **Fair** |
| Dana 2009 | Registry analysis | Yes | Yes | NA | Yes | No | Yes | Yes | NA | Yes | Yes | Yes | No | Yes | No | **Fair** |
| Donegan 2014 | Cohort studies | Yes | Yes | Yes | Yes | No | Yes | Yes | NA | Yes | No | Yes | No | NA | No | **Fair** |
| Fortner 2016 | Cohort studies | Yes | Yes | NA | Yes | No | Yes | Yes | NA | Yes | No | Yes | No | NA | No | **Fair** |
| Goldstein 2021 | Cohort studies | Yes | Yes | Yes | Yes | No | Yes | Yes | NA | Yes | No | Yes | No | No | Yes | **Good** |
| Kharbanda 2016 | Cohort studies | Yes | Yes | Yes | Yes | No | Yes | Yes | NA | Yes | No | Yes | No | NR | Yes | **Fair** |
| Kharbanda 2021 | Surveillance | Yes | Yes | CD | Yes | No | Yes | Yes | NA | Yes | No | Yes | No | NR | NR | **Fair** |
| Maertens 2016 | Cohort studies | Yes | Yes | NA | Yes | Yes | Yes | Yes | NA | Yes | No | Yes | No | NR | Yes | **Fair** |
| Morgan 2015 | Cohort studies | Yes | Yes | Yes | Yes | No | Yes | Yes | NA | Yes | No | Yes | No | NR | Yes | **Good** |
| Petousis-Harris 2016 | Cohort studies | Yes | Yes | Yes | No | No | Yes | Yes | NA | Yes | No | Yes | No | NR | No | **Fair** |
| Regan 2016 | Cohort studies | Yes | Yes | Yes | Yes | No | Yes | Yes | NA | Yes | No | Yes | No | Yes | No | **Fair** |
| Shakib 2013 | Cohort studies | Yes | Yes | Yes | Yes | No | Yes | Yes | NA | Yes | No | Yes | No | NA | No | **Fair** |
| Shimabukuro 2021 | Cohort studies | Yes | Yes | No | Yes | No | Yes | Yes | NA | Yes | Yes | Yes | No | NA | No | **Fair** |
| Zauche 2021 | Registry analysis | Yes | Yes | Yes | Yes | No | Yes | Yes | NA | Yes | Yes | Yes | No | NR | No | **Fair** |
| Zheteyeva 2012 | Registry analysis | Yes | Yes | Yes | Yes | No | Yes | Yes | NA | Yes | NA | Yes | No | No | No | **Fair** |
| Warda 2021 | Cohort studies | Yes | Yes | Yes | Yes | No | Yes | Yes | NA | Yes | No | Yes | No | No | No | **Fair** |
| Blakeway 2021 | Cohort studies | Yes | Yes | No | Yes | No | Yes | Yes | NA | Yes | No | Yes | No | No | Yes | **Good** |

**NA**: Not applicable, **NR**: Not reported, **CD**: cannot be determined

**6.2 Risk of bias of clinical trials**

| **Study ID** | **Adequate sequence generation** | **Allocation concealment** | **Blinding of participant & personnel** | **Blinding of outcome assessment** | **Incomplete outcome data** | **Selective reporting** | **Other sources of bias** |
| --- | --- | --- | --- | --- | --- | --- | --- |
| Glenn 2015 | Unclear | Unclear | Low | Low | Low | Unclear | Unclear |
| Heldens 2009 | High | High | Unclear | Unclear | Low | Unclear | High |
| Madhi 2020 | High | High | Unclear | Unclear | Low | Unclear | Unclear |
| Muñoz 2019 | Unclear | Unclear | High | Low | Low | Unclear | Unclear |
| Stedman 2019 | Unclear | Unclear | Unclear | Unclear | Low | Unclear | Unclear |
| Muñoz 2014 | Low | Low | Low | Low | Low | Unclear | Low |
| Hoang 2016 | Unclear | Unclear | Unclear | Unclear | Low | Unclear | Low |
| Swamy 2020 | Low | Low | Low | Low | Unclear | Low | Low |
| Perrett 2020 | Low | Low | Low | Low | Unclear | Low | Low |
| Segal 2011 | Unclear | Low | Low | Low | Low | Low | Low |
| Shan 2019 | Unclear | Unclear | Unclear | Unclear | Low | Low | Low |
| Wise 2008 | Unclear | Unclear | Unclear | Unclear | Low | Low | Low |

# **Table 7. Meta-analysis and certainty of evidence for controlled studies involving non-COVID-19 vaccines in pregnant persons**

| **Outcome (Effect measure)** | **Trimester Ex.** | **Effect** (95% CI) | **# Studies (Ex vs. no-Ex)** | **I²** | **GRADE** |
| --- | --- | --- | --- | --- | --- |
| **Exposure to vaccines containing AS03 vs. no exposure** | | | | | |
| Spontaneous abortion / miscarriage (aHR) | All | 0.77 (0.39 to 1.50) | 2 (16,507 vs 77,941) | 85% | Very low^2,3^ |
| Stillbirth (aHR) | 1st | 0.97 (0.72 to 1.31) | 1 (41,138 vs 234,317) | NA | Very low^2^ |
|  | 2nd | 0.67 (0.47 to 0.96) | 1 (41,138 vs 234,317) | NA | Low |
|  | 3rd | 1.05 (0.57 to 1.94) | 2 (50,583 vs 264,735) | 51% | Very low^1^ |
|  | All | 0.81 (0.57 to 1.14) | 3 (82,441 vs 291,203) | 46% | Very low^2^ |
| Stillbirth (aOR) | All | 1.63 (0.24 to 10.93) | 1 (18,612 vs 136,914) | 74% | Very low^2,4^ |
| Fetal death (aHR) | All | 0.85 (0.67 to 1.07) | 2 (3,027,812 vs 10,462,156) | 0% | Very low^2^ |
| Fetal death (aRR) | All | 0.66 (0.47 to 0.93) | 1 (23,340 vs 32,230) | NA | Low |
| Early neonatal death (aHR) | 1st | 0.98 (0.52 to 1.85) | 1 (41,138 vs 234,317) | NA | Very low^2^ |
|  | 2nd | 0.64 (0.31 to 1.32) | 1 (41,138 vs 234,317) | NA |  |
|  | 3rd | 0.48 (0.19 to 1.21) | 1 (41,138 vs 234,317) | NA |  |
|  | All | 0.77 (0.51 to 1.18) | 2 (75,379 vs 243,680) | 0% |  |
| Late neonatal death (aHR) | 1st | 0.86 (0.51 to 1.45) | 1 (41,138 vs 234,317) | NA | Very low^2^ |
|  | 2nd | 1.10 (0.69 to 1.75) | 1 (41,138 vs 234,317) | NA |  |
|  | 3rd | 0.93 (0.54 to 1.60) | 1 (41,138 vs 234,317) | NA |  |
|  | All | 0.97 (0.69 to 1.36) | 1 (41,138 vs 234,317) | NA |  |
| Congenital malformations (aOR) | 1st | 1.04 (0.95 to 1.14) | 2 (9,099 vs 56,966) | 0% | Very low^2^ |
|  | All | 1.00 (0.95 to 1.05) | 2 (20,649 vs 146,357) | 0% |  |
| Preterm birth (aHR) | All - preterm birth | 1.00 (0.94 to 1.06) | 2 (3,054,991 vs 10,423,996) | 0% | Very low^2^ |
|  | All - very preterm birth | 0.90 (0.55 to 1.47) | 1 (34,241 vs 9,363) | NA |  |
| Preterm birth (aOR) | 1st | 1.08 (0.92 to 1.28) | 2 (20,359 vs 55,313) | 0% | Very low^2^ |
|  | 2nd/3rd | 0.96 (0.87 to 1.06) | 2 (20,359 vs 55,313) | 0% |  |
|  | All - preterm birth | 0.94 (0.87 to 1.01) | 3 (55,249 vs 176,934) | 42% | Low |
|  | All - very preterm birth | 0.89 (0.51 to 1.54) | 2 (23,849 vs 32,429) | 6% | Very low^1,5^ |
| 5-minute Apgar score < 7 (aRR) | 1st | 1.35 (0.98 to 1.86) | 1 (13,297 vs 7790) | NA | Very low^2^ |
|  | 2nd/3rd | 0.92 (0.70 to 1.21) | 1 (13,297 vs 7790) | NA |  |
|  | All | 1.00 (0.87 to 1.14) | 2 (36,637 vs 40,020) | 0% |  |
| Low Apgar score at term (aHR) | All | 1.08 (0.91 to 1.28) | 1 (3,020,750 vs 10,414,633) | NA | Very low^2^ |
| Low birth weight (aHR) | 1st | 1.11 (0.84 to 1.34) | 1 (34,241 vs 9,363) | NA | Very low^2^ |
|  | 2nd | 0.95 (0.74 to 1.22) | 1 (34,241 vs 9,363) | NA |  |
|  | 3rd | 1.15 (0.85 to 1.56) | 1 (34,241 vs 9,363) | NA |  |
|  | All | 0.98 (0.84 to 1.14) | 2 (3,054,991 vs 10,423,996) | 43% |  |
|  | All-very low birth weight | 0.86 (0.62 to 1.19) | 1 (34,241 vs 9,363) | NA |  |
| Low birth weight (aOR) | 1st | 1.00 (0.80 to 1.24) | 2 (20,359 vs 55,313) | 0% | Very low^2^ |
|  | 2nd/3rd | 0.97 (0.71 to 1.32) | 2 (20,359 vs 55,313) | 84% | Very low^3^ |
|  | All | 0.88 (0.81 to 0.96) | 2 (31,909 vs 144,704) | 0% | Low |
| Small for gestational age <10th percentile (aRR) | 1st | 1.01 (0.89 to 1.14) | 2 (20,359 vs 55,313) | 0% | Very low^2^ |
|  | 2nd/3rd | 0.96 (0.90 to 1.04) | 2 (20,359 vs 55,313) | 0% | Low |
|  | All | 0.96 (0.88 to 1.03) | 3 (55,249 vs 176,934) | 64% | Very low^4^ |
| Fetal growth restriction (aHR) | All | 1.17 (0.98 to 1.40) | 1 (34,241 vs 9,363) | NA | Very low^2^ |
| Gestational diabetes (aOR) | All | 0.94 (0.81 to 1.09) | 1 (18,612 vs 136,914) | NA | Very low^2^ |
| Pre-eclampsia (aOR) | All | 0.99 (0.92 to 1.06) | 2 (19,121 vs 137,113) | 0% | Very low^2^ |
| Caesarean section (aOR) | 1st | 0.95 (0.87 to 1.04) | 1 (13,297 vs 7790) | NA | Low |
|  | 2nd/3rd | 0.93 (0.87 to 0.99) | 1 (13,297 vs 7790) | NA |  |
|  | All | 0.94 (0.89 to 1.00) | 2 (13,806 vs 7,989) | 0% |  |
| **Exposure to vaccines containing aluminum vs. no exposure** | | | | | |
| Respiratory Distress Syndrome (aRR) | All | 0.61 (0.20 to 1.86) | 1 (1,019 vs 253) | NA | Very low^3^ |
| Congenital malformations (aOR) | All | 0.80 (0.38 to 1.68) | 1 (650 vs 1,109) | NA | Very low^3^ |
| Preterm birth (aHR) | 3rd | 0.88 (0.80 to 0.97) | 1 (26,229 vs 97,265) | NA | Low |
|  | All - preterm birth | 1.03 (0.97 to 1.09) | 3 (29,954 vs 103,639) | 0% | Very low^2^ |
| Preterm birth (aOR) | All - preterm birth | 0.90 (0.67 to 1.20) | 3 (3,189 vs 1,306,227) | 72% | Very low^2,4^ |
| Low Apgar at 5 minutes (aRR) | All | 0.79 (0.35 to 1.80) | 2 (1,669 vs 1,362) | 0% | Very low^1^ |
| Low birth weight (aOR) | All | 1.03 (0.82 to 1.30) | 3 (3,189 vs 1,306,227) | 49% | Very low^2^ |
| Low birth weight (aHR) | All | 0.90 (0.55 to 1.46) | 2 (3,725 vs 6,374) | 21% | Very low^2^ |
| Small for gestational age <10^th^ percentile (aHR) | All | 1.00 (0.96 to 1.04) | 3 (29,954 vs 103,639) | 0% | Low |
| Small for gestational age (aOR) | 3rd | 1.03 (0.96 to 1.11) | 1 (26,229 vs 97,265) | NA | Very low^2^ |
|  | All | 1.18 (1.00 to 1.39) | 3 (3,189 vs 1,306,227) | 29% |  |
| Gestational diabetes (aOR) | All | 1.00 (0.88 to 1.14) | 2 (2,539 vs 1,305,118) | 18% | Very low^2^ |
| Gestational hypertension (aHR) | All | 1.33 (0.70 to 2.53) | 1 (1,019 vs 253) | NA | Very low^1^ |
| Gestational hypertension (aOR) | All | 1.03 (0.91 to 1.17) | 3 (28,768 vs 1,402,383) | 27% | Very low^2^ |
| Pre-eclampsia/eclampsia (aHR) | All | 0.75 (0.47 to 1.20) | 1 (1,019 vs 253) | NA | Very low^2^ |
| Pre-eclampsia/eclampsia (aOR) | All | 1.01 (0.84 to 1.21) | 2 (2,539 vs 1,305,118) | 0% | Very low^2^ |
| Cesarean delivery (aOR) | All | 0.90 (0.70 to 1.16) | 2 (2,049 vs 653,541) | 78% | Very low^2,3^ |
| Postpartum hemorrhage (aRR) | All | 0.81 (0.52 to 1.26) | 1 (1,019 vs 253) | NA | Very low^2^ |
| Chorioamnionitis/funisitis (aRR) | All | 0.71 (0.27 to 1.87) | 1 (1,019 vs 253) | NA | Very low^1^ |

**aHR**: adjusted Hazard Ratio; **aRR**: adjusted Risk Ratio; **aOR**: adjusted Odds Ratio;

in the last year; **CI**: Confidence interval; **NA**; Not applicable; **Ex**: Exposure; **1^st^**: first; **2^nd^**: second; **3^rd^**: third

**GRADE** certainty of evidence: started “Low” due to the observational designs, and was downgraded due to the limits agreed by our research team:

1. Downgraded two levels due to very serious imprecision (crossing the null effect, and 95%CI < 0.5 and/or > 1.50)
2. Downgraded one level due to serious imprecision (crossing the null effect, and 95%CI < 0.95 and/or >1.05)
3. Downgraded two levels due to very serious inconsistency (I^2^ >75%)
4. Downgraded one level due to serious inconsistency (I^2^ >60%)
5. Downgraded one level due to risk of bias (The sensitivity analysis estimation, excluding Ray 2014 of high RoB is 0.73; 95%CI 0.58 to 0.92; Low certainty of evidence)

# **Table 8. Proportional meta-analysis for uncontrolled studies involving non-COVID-19 vaccines in pregnant persons**

| **Outcomes** | | **Vaccines using AS03 % (95 CI)** | **Vaccines using aluminum % (95 CI)** | **Historical rates** |
| --- | --- | --- | --- | --- |
| Spontaneous abortion/miscarriage | | NA | 11.98 (9.27-14.99)^S13^ | 10% (in clinically recognized pregnancies)[[113](#_ENREF_113)] |
| Fetal death | | 1.83 (1.69-1.98)^S1^ | 0.82 (0.00-3.40)^S2^ | NA |
| Stillbirth | | 0.11 (0.00-0.04)^S4^ | 0.04 (0.03-0.06)^S18^ | Finland 2010: 2.9%[[114](#_ENREF_114)]  Belgium 2010: 3.1%[[115](#_ENREF_115)]  UK 2017: 4.2%[[116](#_ENREF_116)]  Argentina, USA 2019: 3.0%[[115](#_ENREF_115)]  Australia 2010: 2.9%[[115](#_ENREF_115)]  Vietnam 2010: 9.6%[[115](#_ENREF_115)]  New Zealand 2010: 3.3%[[115](#_ENREF_115)] |
| Fetal growth retardation | | 1.36 (0.56-2.50)^S1^ | 0.89 (0.01-2.08)^S2^ | 23.8% newborns per year[[117](#_ENREF_117)] |
| Gestational diabetes | | NA | 2.63 (0.09-8.49)^S7^ | 6-7%[[118](#_ENREF_118)] |
| Hypertensive disorders | | 3.98 (3.74-4.24)^S2^ | 2.48 (1.50-3.71)^S14^ | Hypertensive disorders: 10%  Preeclampsia: 4-5%[[119](#_ENREF_119)] |
| Low birth weight | | 3.73 (2.55-5.11)^S3^ | 5.07 (2.84-7.91)^S8^ | Estimations for 2019[[120](#_ENREF_120)]  Global rate: 6%  Argentina: 7.2%  Sweden: 4.2 %  Denmark: 5.2%  USA: 8.1%  Australia: 6.2 %  UK: 5.3% |
| Small for gestational age | | 9.49 (8.33-10.72)^S3^ | 6.84 (5.58-8.21)^S9^ | Asia: 5.3 to 41.5%  Africa: 1.2 to 3.0% livebirths[[121](#_ENREF_121)] |
| Preterm birth | | 5.38 (4.49-6.40)^S7^ | 5.52 (5.00-6.08)^S20^ | 10%[[122](#_ENREF_122)] |
| Maternal death | | NA | NA | Israel: 2/100,000 livebirths[[123](#_ENREF_123)] |
| Any congenital malformation | | 4.97 (2.74-7.81)^S4^ | 3.90 (2.47-5.64)^S16^ | 2-4%[[124](#_ENREF_124)] |
| Neonatal death | | 0.07 (0.04-0.10)^S1^ | 0.20 (0.05-0.45)^S8^ | Estimations for 2019[[125](#_ENREF_125)] Argentina: 6%  USA: 3.6%  Australia: 2.3%  UK: 2.7%  Finland: 1.4%  New Zealand: 2.6%  Israel: 1.9% |
| Postpartum hemorrhage | |  | 2.71 (1.10-5.02)^S7^ | 2-5%[[126](#_ENREF_126)] |
| Suspected Chorioamnionitis | | NA | NA | USA: 9.7 per 1000 live births |
| Antenatal bleeding | |  | 0.44 (0.10-1.02)^S1^ | 6%[[127](#_ENREF_127)] |
| Neonatal infections | |  | 7.23 (0.21-22.82)^S4^ | NA |
| Neonatal encephalopathy | | NA | 0.23 (0.09-0.42)^S2^ | HICs: 0.25/100 livebirths[[128](#_ENREF_128)] |
| Respiratory distress newborn | | NA | 1.23 (0.38-2.56)^S4^ | LMICs: 1.2% to 7.2% [[129](#_ENREF_129)]  USA: 10%[[130](#_ENREF_130)] |
| Failure to thrive | | NA | 0.42 (0.00-1.75)^S3^ | HICs: 1–10%[[131](#_ENREF_131)] |
| Microcephaly | | NA | 0.78 (0.50-1.13)^S1^ | Europe: 1.53/10,000 births[[132](#_ENREF_132)]  USA: 8.7/10,000 livebirths[[133](#_ENREF_133)] |
| Injection site reactions | LMICs | 37.21 (1.15-97.74)^S3^ | 22.06 (13.83-31.57)^S12^ | Not applicable |
|  | HICs | 68.95 (61.26-76.15)^S2^ |  | Not applicable |
| Fever | | 0.23 (0.20-0.27)^S1^ | 2.68 (0.72-5.84)^S12^ | Not applicable |
| Headache | | 2.20 (0.19-0.25)^S1^ | 14.32 (2.37-33.92)^S9^ | Not applicable |
| Gastrointestinal | | NA | 17.51 (13.75-21.61)^S2^ | Not applicable |
| Malaise | | NA | 3.07 (0.29-8.65)^S4^ | Not applicable |
| Myalgia | | NA | 3.88 (1.37-7.58)^S5^ | Not applicable |
| Fatigue | | NA | 15.32 (4.57-30.84)^S6^ | Not applicable |
| Vomiting | | 0.09 (0.07-0.11)^S1^ | 2.00 (0.48-4.54)^S3^ | Not applicable |
| Nausea | | NA | 5.13 (0.44-14.48)^S3^ | Not applicable |
| Chills | | NA | 2.28 (0.13-7.01)^S2^ | Not applicable |
| Joint pain | | 0.05 (0.04-0.07)^S1^ | 3.12 (2.06-4.38)^S3^ | Not applicable |
| Systemic reactions (non-minor) | | 25.82 (17.38-35.29)^S1^ | 17.45 (4.80-35.75)^S2^ | Not applicable |
| Any systemic reactions | | NA | 41.22 (39.48-42.97)^S1^ | Not applicable |
| Anaphylaxis | | 0.023 (0.001-0.003)^S1^ | NA | Not applicable |
| MAEs | | NA | 33.90 (11.79-99.95)^S2^ | Not applicable |
| SAEs | | NA | 12.31 (1.27-32.24)^S6^ | Not applicable |

**MAEs**: Medically attended adverse events; **SAEs**: Serious adverse events; **NA**: Not available; **HICs**: High Income Countries; **LMIC**s: Low-to-Middle-Income Countries. **Sx** indicates the number of contributive studies (for details see **Appendix B**).

# **References**

[1] Adeel B, Abdul-Badi A-S, Hiam C, Abdullatif Al K, Peter C, Huda S, et al. Effectiveness of the SARS-CoV-2 mRNA Vaccines in Pregnant Women. Research Square. 2021.

[2] Alguacil-Ramos AM, Garrigues-Pelufo TM, Muelas-Tirado J, Portero-Alonso A, Perez-Panades J, Fons-Martinez J. [Safety of influenza vaccines in risk groups: analysis of adverse events following immunization reported in Valencian Community from 2005 to 2011]. Seguridad de las vacunas antigripales en grupos de riesgo: analisis de las sospechas de reacciones adversas notificadas en Comunidad Valenciana entre 2005 y 2011. 2015;28:193-9.

[3] Arriola CS, Vasconez N, Thompson MG, Olsen SJ, Moen AC, Bresee J, et al. Association of influenza vaccination during pregnancy with birth outcomes in Nicaragua. Vaccine. 2017;35:3056-63.

[4] Beau AB, Hurault-Delarue C, Vidal S, Guitard C, Vayssiere C, Petiot D, et al. Pandemic A/H1N1 influenza vaccination during pregnancy: a comparative study using the EFEMERIS database. Vaccine. 2014;32:1254-8.

[5] Beharier O, Mayo RP, Raz T, Sacks KN, Schreiber L, Suissa-Cohen Y, et al. Efficient maternal to neonatal transfer of antibodies against SARS-CoV-2 and BNT162b2 mRNA COVID-19 vaccine. Journal of Clinical Investigation. 2021;131.

[6] Burd I, Kino T, Segars J. The Israeli study of Pfizer BNT162b2 vaccine in pregnancy: Considering maternal and neonatal benefits. Journal of Clinical Investigation. 2021;131.

[7] Carcione D, Blyth CC, Richmond PC, Mak DB, Effler PV. Safety surveillance of influenza vaccine in pregnant women. The Australian & New Zealand journal of obstetrics & gynaecology. 2013;53:98-9.

[8] Chambers CD, Johnson D, Xu RH, Luo YJ, Louik C, Mitchell AA, et al. Risks and safety of pandemic H1N1 influenza vaccine in pregnancy: birth defects, spontaneous abortion, preterm delivery, and small for gestational age infants. Vaccine. 2013;31:5026-32.

[9] Chambers CD, Louik C, Jones KL, Mitchell AA, Schatz M. Safety of seasonal influenza vaccines in pregnancy: VAMPSS update. Pharmacoepidemiology and Drug Safety. 2015;24:12.

[10] Chambers CD, Johnson DL, Xu R, Luo YJ, Louik C, Mitchell AA, et al. Safety of the 2010-11, 2011-12, 2012-13, and 2013-14 seasonal influenza vaccines in pregnancy: birth defects, spontaneous abortion, preterm delivery, and small for gestational age infants, a study from the cohort arm of VAMPSS. Vaccine. 2016;34:4443-9.

[11] Choe YJ, Cho H, Song KM, Kim J-H, Han OP, Kwon YH, et al. Active surveillance of adverse events following immunization against pandemic influenza A (H1N1) in Korea. Japanese journal of infectious diseases. 2011;64:297-303.

[12] Conlin AMS, Bukowinski AT, Sevick CJ, DeScisciolo C, Crum-Cianflone NF. Safety of the pandemic H1N1 influenza vaccine among pregnant U.S. military women and their newborns. Obstetrics & Gynecology (New York). 2013;121:511-8.

[13] Covington D, Kaydo S, Veley K. Hepatitis B virus (HBV) vaccine in pregnancy and impact on pregnancy outcome. Value in Health. 2018;21:S151.

[14] Cross JW, Joy M, McGee C, Akinyemi O, Gatenby P, Lusignan Sd. Adverse events of interest vary by influenza vaccine type and brand: Sentinel network study of eight seasons (2010-2018). Vaccine. 2020;38:3869-80.

[15] Dodds L, McNeil S, Scott J, Allen VM, Spencer A, MacDonald N. Influenza vaccination in pregnancy. American Journal of Epidemiology. 2011;173:S40.

[16] Donahue JG, Kieke BA, King JP, DeStefano F, Mascola MA, Irving SA, et al. Association of spontaneous abortion with receipt of inactivated influenza vaccine containing H1N1pdm09 in 2010-11 and 2011-12. Vaccine. 2017;35:5314-22.

[17] Donahue JG, Kieke BA, King JP, Mascola MA, Shimabukuro TT, Destefano F, et al. Inactivated influenza vaccine and spontaneous abortion in the Vaccine Safety Datalink in 2012-13, 2013-14, and 2014-15. Vaccine. 2019;37:6673-81.

[18] Durrieu G, Caillet C, Faucher A, Jacquet A, Ouaret S, Auriche P, et al. Safety surveillance of influenza A(H1N1)v monovalent vaccines during the 2009-2010 mass vaccination campaign in France. Fundamental and Clinical Pharmacology. 2011;25:72-3.

[19] Eaton A, Lewis N, Fireman B, Hansen J, Baxter R, Gee J, et al. Birth outcomes following immunization of pregnant women with pandemic H1N1 influenza vaccine 2009-2010. Vaccine. 2018;36:2733-9.

[20] Golan Y, Prahl M, Cassidy AG, Gay C, Wu AHB, Jigmeddagva U, et al. COVID-19 mRNA Vaccination in Lactation: Assessment of adverse effects and transfer of anti-SARS-CoV2 antibodies from mother to child. medRxiv : the preprint server for health sciences. 2021.

[21] Goldman GS. Comparison of VAERS fetal-loss reports during three consecutive influenza seasons: was there a synergistic fetal toxicity associated with the two-vaccine 2009/2010 season? Human & Experimental Toxicology. 2013;32:464-75.

[22] Kankawinpong O, Sangsajja C, Cholapand A, Manosuthi W, Thientong V, Nuntapanich N, et al. Immunogenicity and safety of an inactivate pandemic H1N1 vaccine provided by the Thai ministry of public health as a routine public health service. The Southeast Asian journal of tropical medicine and public health. 2012;43:680-6.

[23] Kharbanda EO, Vazquez-Benitez G, Lipkind H, Naleway A, Lee G, Nordin JD. Inactivated influenza vaccine during pregnancy and risks for adverse obstetric events. Obstetrics & Gynecology (New York). 2013;122:659-67.

[24] Kharbanda EO, Vazquez-Benitez G, Romitti PA, Naleway AL, Cheetham TC, Lipkind HS, et al. First trimester influenza vaccination and risks for major structural birth defects in offspring. Journal of Pediatrics. 2017;187:234-e4.

[25] Kozuki N, Katz J, Englund JA, Steinhoff MC, Khatry SK, Shrestha L, et al. Impact of maternal vaccination timing and influenza virus circulation on birth outcomes in rural Nepal. International Journal of Gynecology & Obstetrics. 2018;140:65-72.

[26] Louik C, Ahrens K, Kerr S, Pyo JH, Chambers C, Jones KL, et al. Risks and safety of pandemic H1N1 influenza vaccine in pregnancy: exposure prevalence, preterm delivery, and specific birth defects. Vaccine. 2013;31:5033-40.

[27] Louik C, Kerr S, Bennekom CMv, Chambers C, Jones KL, Schatz M, et al. Safety of the 2011-12, 2012-13, and 2013-14 seasonal influenza vaccines in pregnancy: preterm delivery and specific malformations, a study from the case-control arm of VAMPSS. Vaccine. 2016;34:4450-9.

[28] Lylianou D, Phousavath S, Vongphrachanh P, Phonekeo D, Corwin A, Khanthamaly V, et al. Adverse events following immunization from pandemic influenza A (H1N1)-Laos 2010. International Journal of Infectious Diseases. 2012;16:e308-e9.

[29] McHugh L, Andrews RM, Lambert SB, Viney KA, Wood N, Perrett KP, et al. Birth outcomes for Australian mother-infant pairs who received an influenza vaccine during pregnancy, 2012-2014: the FluMum study. Vaccine. 2017;35:1403-9.

[30] McHugh L, Binks MJ, Gao Y, Andrews RM, Ware RS, Snelling T, et al. Influenza vaccination in pregnancy among a group of remote dwelling Aboriginal and Torres Strait Islander mothers in the Northern Territory: The 1+1 Healthy Start to Life study. Communicable diseases intelligence (2018). 2019;43.

[31] Macias Saint-Gerons D, Sola Arnau I, De Mucio B, Arevalo-Rodriguez I, Aleman A, Castro JL, et al. Adverse events associated with the use of recommended vaccines during pregnancy: An overview of systematic reviews. Vaccine. 2021;39 Suppl 2:B12-B26.

[32] Mohammed H, Roberts CT, Grzeskowiak LE, Giles LC, Dekker GA, Marshall HS. Safety and protective effects of maternal influenza vaccination on pregnancy and birth outcomes: A prospective cohort study. EClinicalMedicine. 2020;26.

[33] Moro PL, Cragan J, Lewis P, Sukumaran L. Major Birth Defects after Vaccination Reported to the Vaccine Adverse Event Reporting System (VAERS), 1990 to 2014. Birth Defects Research. 2017;109:1057-62.

[34] Moro PL, Marquez P. Reports of cell-based influenza vaccine administered during pregnancy in the Vaccine Adverse Event Reporting System (VAERS), 2013–2020. Vaccine. 2020.

[35] Nordin JD, Kharbanda EO, Benitez GV, Nichol K, Lipkind H, Naleway A, et al. Maternal safety of trivalent inactivated influenza vaccine in pregnant women. Obstetrics & Gynecology (New York). 2013;121:519-25.

[36] Ohfuji S, Deguchi M, Tachibana D, Koyama M, Takagi T, Yoshioka T, et al. Safety of influenza vaccination on adverse birth outcomes among pregnant women: A prospective cohort study in Japan. International journal of infectious diseases : IJID : official publication of the International Society for Infectious Diseases. 2020;93:68-76.

[37] Omer SB, Goodman D, Steinhoff MC, Rochat R, Klugman KP, Stoll BJ, et al. Maternal influenza immunization and reduced likelihood of prematurity and small for gestational age births: a retrospective cohort study. PLoS Medicine. 2011;8:e1000441.

[38] Panagiotakopoulos L, McCarthy NL, Tepper NK, Kharbanda EO, Lipkind HS, Vazquez-Benitez G, et al. Evaluating the Association of Stillbirths After Maternal Vaccination in the Vaccine Safety Datalink. Obstetrics and gynecology. 2020;136:1086-94.

[39] Peppa M, Thomas SL, Minassian C, Walker JL, McDonald HI, Andrews NJ, et al. Seasonal influenza vaccination during pregnancy and the risk of major congenital malformations in live-born infants: A 2010-2016 historical cohort study. Clinical infectious diseases : an official publication of the Infectious Diseases Society of America. 2020.

[40] Phengxay M, Mirza SA, Reyburn R, Xeuatvongsa A, Winter C, Lewis H, et al. Introducing seasonal influenza vaccine in low-income countries: an adverse events following immunization survey in the Lao People's Democratic Republic. Influenza and other respiratory viruses. 2015;9:94‐8.

[41] Ran B, Noa D, Noam B, Tal B-S, Maya M-A, Calanit K, et al. Effectiveness of the BNT162b2 mRNA COVID-19 Vaccine in Pregnancy. Nature Portfolio. 2021.

[42] Regan AK, Blyth CC, Mak DB, Richmond PC, Effler PV. Using SMS to monitor adverse events following trivalent influenza vaccination in pregnant women. Australian and New Zealand Journal of Obstetrics and Gynaecology. 2014;54:522-8.

[43] Regan AK, Tracey LE, Blyth CC, Richmond PC, Effler PV. A prospective cohort study assessing the reactogenicity of pertussis and influenza vaccines administered during pregnancy. Vaccine. 2016;34:2299-304.

[44] Regan AK, Moore HC, Sullivan SG. Does influenza vaccination during early pregnancy really increase the risk of miscarriage? Vaccine. 2018;36:2227-8.

[45] Richner JM, Jagger BW, Shan C, Fontes CR, Dowd KA, Cao B, et al. Vaccine mediated protection against Zika virus-induced congenital disease. Cell (Cambridge). 2017;170:273-e12.

[46] Rottenstreich A, Zarbiv G, Oiknine-Djian E, Zigron R, Wolf DG, Porat S. Efficient maternofetal transplacental transfer of anti- SARS-CoV-2 spike antibodies after antenatal SARS-CoV-2 BNT162b2 mRNA vaccination. Clinical infectious diseases : an official publication of the Infectious Diseases Society of America. 2021.

[47] Shatla MM, Khayat ME, Ahmed MM, Alzahrani AA, Khadrawi AA, Almisfer AS, et al. Effect of maternal antenatal influenza vaccination on adverse neonatal outcomes in terms of premature birth, small-for-gestational age and low birth weight: a comparative study. International Journal of Medical Science and Public Health. 2016;5:2378-84.

[48] Vazquez-Benitez G, Kharbanda EO, Naleway AL, Lipkind H, Lakshmi S, McCarthy NL, et al. Risk of preterm or small-for-gestational-age birth after influenza vaccination during pregnancy: caveats when conducting retrospective observational studies. American Journal of Epidemiology. 2016;184:176-86.

[49] Walsh LK, Donelle J, Dodds L, Hawken S, Wilson K, Benchimol EI, et al. Health outcomes of young children born to mothers who received 2009 pandemic H1N1 influenza vaccination during pregnancy: retrospective cohort study. BMJ (Clinical research ed). 2019;366:l4151.

[50] Wijnans L, Dodd CN, Weibel D, Sturkenboom M. Bell's palsy and influenza(H1N1)pdm09 containing vaccines: a self-controlled case series. PLoS ONE. 2017;12:e0175539.

[51] Zdanowski W, Wasniewski T. Evaluation of SARS-CoV-2 Spike Protein Antibody Titers in Cord Blood after COVID-19 Vaccination during Pregnancy in Polish Healthcare Workers: Preliminary Results. Vaccines. 2021;9.

[52] Guo Y, Allen V, Bujold E, Coleman B, Drews S, Gouin K, et al. Efficacy and safety of pandemic influenza vaccine in pregnancy. Canadian Journal of Infectious Diseases and Medical Microbiology. 2010;21:209.

[53] Tavares F, Nazareth I, Monegal JS, Kolte I, Verstraeten T, Bauchau V. Pregnancy and safety outcomes in women vaccinated with an AS03-adjuvanted split virion H1N1 (2009) pandemic influenza vaccine during pregnancy: a prospective cohort study. Vaccine. 2011;29:6358-65.

[54] Baum U, Leino T, Gissler M, Kilpi T, Jokinen J. Perinatal survival and health after maternal influenza A(H1N1)pdm09 vaccination: A cohort study of pregnancies stratified by trimester of vaccination. Vaccine. 2015;33:4850-7.

[55] Chavant F, Ingrand I, Jonville-Bera AP, Plazanet C, Gras-Champel V, Lagarce L, et al. The PREGVAXGRIP study: a cohort study to assess foetal and neonatal consequences of in utero exposure to vaccination against A(H1N1)v2009 influenza. Drug safety. 2013;36:455-65.

[56] Fell DB, Sprague AE, Liu N, Yasseen AS, 3rd, Wen S-W, Smith G, et al. H1N1 influenza vaccination during pregnancy and fetal and neonatal outcomes. American journal of public health. 2012;102:e33-40.

[57] Galindo Santana BM, Pelaez Sanchez OR, Galindo Sardina MA, Leon Villafuerte M, Concepcion Diaz D, Estruch Rancano L, et al. Active surveillance of adverse effects of Pandemrix vaccine to prevent influenza A(H1N1) in Cuba. Vigilancia activa de eventos adversos a la vacuna Pandemrixpara prevenir la influenza AH1N1 en Cuba. 2011;63:231-8.

[58] Källén B, Olausson PO. Vaccination against H1N1 influenza with Pandemrix® during pregnancy and delivery outcome: A Swedish register study. BJOG: An International Journal of Obstetrics and Gynaecology. 2012;119:1583-90.

[59] Lacroix I, Damase-Michel C, Kreft-Jais C, Castot AC, Montastruc JL. H1N1 influenza vaccine in pregnant women: French pharmacovigilance survey. Drug Safety. 2010;33:908-9.

[60] Layton D, Dryburgh M, MacDonald TM, Shakir SA, MacKenzie IS. Pilot swine flu vaccination active surveillance study: Final results. Drug Safety. 2011;34:889-90.

[61] Levi M. Vaccination against influenza A(H1N1) virus is also safe during pregnancy. Nederlands Tijdschrift voor Geneeskunde. 2012;156.

[62] Ludvigsson JF, Zugna D, Cnattingius S, Richiardi L, Ekbom A, Ortqvist A, et al. Influenza H1N1 vaccination and adverse pregnancy outcome. European Journal of Epidemiology. 2013;28:579-88.

[63] Ludvigsson JF, Strom P, Lundholm C, Cnattingius S, Ekbom A, Ortqvist A, et al. Risk for congenital malformation with H1N1 influenza vaccine: a cohort study with sibling analysis. Annals of Internal Medicine. 2016;165:848-55.

[64] Mackenzie IS, MacDonald TM, Shakir S, Dryburgh M, Mantay BJ, McDonnell P, et al. Influenza H1N1 (swine flu) vaccination: a safety surveillance feasibility study using self-reporting of serious adverse events and pregnancy outcomes. British Journal of Clinical Pharmacology. 2012;73:801-11.

[65] Núñez Rojas Y, Orive Rodríguez N, Varona De La Peña F, Bermúdez Velásquez Y, Raad López AF, Muñoz Martínez Y, et al. Vaccination against influenza a H1N1 and the risk of birth defects. VacciMonitor. 2010;19:209.

[66] Oppermann M, Fritzsche J, Weber-Schoendorfer C, Keller-Stanislawski B, Allignol A, Meister R, et al. A(H1N1)v2009: a controlled observational prospective cohort study on vaccine safety in pregnancy. Vaccine. 2012;30:4445-52.

[67] Pasternak B, Svanstrom H, Molgaard-Nielsen D, Krause TG, Emborg HD, Melbye M, et al. Vaccination against pandemic A/H1N1 2009 influenza in pregnancy and risk of fetal death: cohort study in Denmark. BMJ. 2012;344.

[68] Pasternak B, Svanstrom H, Molgaard-Nielsen D, Krause TG, Emborg HD, Melbye M, et al. Risk of adverse fetal outcomes following administration of a pandemic influenza A(H1N1) vaccine during pregnancy. JAMA, Journal of the American Medical Association. 2012;308:165-74.

[69] Ray JG, McGeer AJ, Blake JM, Lebovic G, Smith GN, Yudin MH. Peripartum outcomes: non-adjuvanted v. adjuvanted H1N1 vaccination. CMAJ : Canadian Medical Association journal = journal de l'Association medicale canadienne. 2014;186:137.

[70] Sammon CJ, Snowball J, McGrogan A, de Vries CS. Evaluating the Hazard of Foetal Death following H1N1 Influenza Vaccination; A Population Based Cohort Study in the UK GPRD. PLoS ONE. 2012;7.

[71] Folkenberg M, Callreus T, Svanstrom H, Valentiner-Branth P, Hviid A. Spontaneous reporting of adverse events following immunisation against pandemic influenza in Denmark November 2009-March 2010. Vaccine. 2011;29:1180-4.

[72] Haberg SE, Trogstad L, Gunnes N, Wilcox AJ, Gjessing HK, Samuelsen SO, et al. Risk of fetal death after pandemic influenza virus infection or vaccination. The New England journal of medicine. 2013;368:333-40.

[73] Läkemedelsverket. Läkemedelsverket. Final summary of adverse drug reaction reports in Sweden with Pandemrix through October 2009-mid April 2010. June 2, 2010. Accessed 23 May 2011 from <www.lakemedelsverket.se>. 2010.

[74] Ludvigsson JF, Strom P, Lundholm C, Cnattingius S, Ekbom A, Ortqvist A, et al. Maternal vaccination against H1N1 influenza and offspring mortality: Population based cohort study and sibling design. BMJ 2015;351:h5585.

[75] Sammon CJ, McGrogan A, Snowball J, De Vries CS. Swine flu vaccination in pregnancy and associated miscarriage risk. Pharmacoepidemiology and Drug Safety. 2011;20:S58-S9.

[76] Groom HC, Smith N, Irving SA, Koppolu P, Vazquez-Benitez G, Kharbanda EO, et al. Uptake and safety of hepatitis A vaccination during pregnancy: A Vaccine Safety Datalink study. Vaccine. 2019;37:6648-55.

[77] Hoang HTT, Leuridan E, Maertens K, Nguyen TD, Hens N, Vu NH, et al. Pertussis vaccination during pregnancy in Vietnam: Results of a randomized controlled trial Pertussis vaccination during pregnancy. Vaccine. 2016;34:151-9.

[78] Katz N, Neyro S, Carrega MEP, Del Valle Juarez M, Rancaño C, Pasinovich M, et al. Maternal immunization in argentina: The importance of a safety profile analysis. Open Forum Infectious Diseases. 2016;3.

[79] Madhi SA, Polack FP, Piedra PA, Munoz FM, Trenholme AA, Simoes EA, et al. Vaccination of pregnant women with respiratory syncytial virus vaccine and protection of their infants. New England journal of medicine. 2020;383:426‐39.

[80] Maertens K, Cabore RN, Huygen K, Hens N, Van Damme P, Leuridan E. Pertussis vaccination during pregnancy in Belgium: results of a prospective controlled cohort study. Vaccine. 2016;34:142‐50.

[81] McHugh L, Marshall HS, Perrett KP, Nolan T, Wood N, Lambert SB, et al. The safety of influenza and pertussis vaccination in pregnancy in a cohort of Australian mother-infant pairs, 2012-2015: the FluMum study. Clinical Infectious Diseases. 2019;68:402-8.

[82] Mohammed H, Roberts CT, Grzeskowiak LE, Giles LC, Verburg PE, Dekker G, et al. Safety of maternal pertussis vaccination on pregnancy and birth outcomes: A prospective cohort study. Vaccine. 2021;39:324-31.

[83] Swamy GK, Metz TD, Edwards KM, Soper DE, Beigi RH, Campbell JD, et al. Safety and immunogenicity of an investigational maternal trivalent group B streptococcus vaccine in pregnant women and their infants: results from a randomized placebo-controlled phase II trial. Vaccine. 2020;38:6930‐40.

[84] Zheteyeva YA, Moro PL, Tepper NK, Rasmussen SA, Barash FE, Revzina NV, et al. Adverse event reports after tetanus toxoid, reduced diphtheria toxoid, and acellular pertussis vaccines in pregnant women. Am J Obstet Gynecol. 2012;207:59 e1-7.

[85] Munoz FM, Bond NH, Maccato M, Pinell P, Hammill HA, Swamy GK, et al. Safety and Immunogenicity of Tetanus Diphtheria and Acellular Pertussis (Tdap) Immunization During Pregnancy in Mothers and Infants. JAMA. 2014;311:1760.

[86] Kushner T, Youhanna J, Walker R, Erby K, Janssen RS. Safety and immunogenicity of Heplisav-B in pregnancy. Hepatology. 2020;72:469A-70A.

[87] Rega A, Moore H, De Klerk N, Effler P. Maternal vaccinations in Australia-uptake, safety and impact. Australian and New Zealand Journal of Obstetrics and Gynaecology. 2016;56:13-4.

[88] Munoz FM, Swamy GK, Hickman SP, Agrawal S, Piedra PA, Glenn GM, et al. Safety and Immunogenicity of a Respiratory Syncytial Virus Fusion (F) Protein Nanoparticle Vaccine in Healthy Third-Trimester Pregnant Women and Their Infants. J Infect Dis. 2019;220:1802-15.

[89] Perrett KP, Halperin SA, Nolan T, Martínez Pancorbo C, Tapiero B, Martinón-Torres F, et al. Immunogenicity, transplacental transfer of pertussis antibodies and safety following pertussis immunization during pregnancy: evidence from a randomized, placebo-controlled trial. Vaccine. 2020;38:2095‐104.

[90] Petousis-Harris H, Walls T, Watson D, Paynter J, Graham P, Turner N. Safety of Tdap vaccine in pregnant women: an observational study. BMJ Open. 2016;6:e010911.

[91] Fortner KB, Edwards KM, Broder KR, Jimenez N, Zhu Y, Walter EB, et al. 343: Reactogenicity of tetanus toxoid, reduced diphtheria toxoid, and acellular pertussis vaccine (Tdap) in pregnant women. American Journal of Obstetrics and Gynecology. 2016;214:S193-S4.

[92] Angelo M-G, David M-P, Zima J, Baril L, Dubin G, Arellano F, et al. Pooled analysis of large and long-term safety data from the human papillomavirus-16/18-AS04-adjuvanted vaccine clinical trial programme. Pharmacoepidemiology and drug safety. 2014;23:466-79.

[93] Angelo M-G, Zima J, Tavares Da Silva F, Baril L, Arellano F. Post-licensure safety surveillance for human papillomavirus-16/18-AS04-adjuvanted vaccine: more than 4 years of experience. Pharmacoepidemiology and drug safety. 2014;23:456-65.

[94] Baril L, Rosillon D, Willame C, Angelo MG, Zima J, van den Bosch JH, et al. Risk of spontaneous abortion and other pregnancy outcomes in 15-25 year old women exposed to human papillomavirus-16/18 AS04-adjuvanted vaccine in the United Kingdom. Vaccine. 2015;33:6884-91.

[95] Berenson AB, Hirth JM, Rahman M, Laz TH, Rupp RE, Sarpong KO. Maternal and infant outcomes among women vaccinated against pertussis during pregnancy. Human Vaccines & Immunotherapeutics. 2016;12:1965-71.

[96] Celzo F, Buyse H, Welby S, Ibrahimi A. Safety evaluation of adverse events following vaccination with Havrix, Engerix-B or Twinrix during pregnancy. Vaccine. 2020;38:6215-23.

[97] Dana A, Buchanan KM, Goss MA, Seminack MM, Shields KE, Korn S, et al. Pregnancy outcomes from the pregnancy registry of a human papillomavirus type6/11/16/18 vaccine. Obstetrics and Gynecology. 2009;114:1170-8.

[98] Donegan K, King B, Bryan P. Safety of pertussis vaccination in pregnant women in UK: observational study. BMJ. 2014;349:g4219-g.

[99] Groom HC, Irving SA, Koppolu P, Smith N, Vazquez-Benitez G, Kharbanda EO, et al. Uptake and safety of Hepatitis B vaccination during pregnancy: a Vaccine Safety Datalink study. Vaccine. 2018;36:6111-6.

[100] Kharbanda EO, Vazquez-Benitez G, Lipkind HS, Klein NP, Cheetham TC, Naleway A, et al. Evaluation of the Association of Maternal Pertussis Vaccination With Obstetric Events and Birth Outcomes. JAMA. 2014;312:1897.

[101] Kharbanda EO, Vazquez-Benitez G, Lipkind HS, Klein NP, Cheetham TC, Naleway AL, et al. Maternal Tdap vaccination: Coverage and acute safety outcomes in the vaccine safety datalink, 2007–2013. Vaccine. 2016;34:968-73.

[102] Moro PL, Museru OI, Niu M, Lewis P, Broder K. Reports to the vaccine adverse event reporting system after hepatitis a and hepatitis AB vaccines in pregnant women. American Journal of Obstetrics and Gynecology. 2014;210:561.e1-.e6.

[103] Moro PL, Zheteyeva Y, Barash F, Lewis P, Cano M. Assessing the safety of hepatitis B vaccination during pregnancy in the Vaccine Adverse Event Reporting System (VAERS), 1990-2016. Vaccine. 2018;36:50-4.

[104] Shakib JH, Korgenski K, Sheng X, Varner MW, Pavia AT, Byington CL. Tetanus, Diphtheria, Acellular Pertussis Vaccine during Pregnancy: Pregnancy and Infant Health Outcomes. The Journal of Pediatrics. 2013;163:1422-6.e4.

[105] Bielefeldt-Ohmann H, Prow NA, Wang W, Tan CSE, Coyle M, Douma A, et al. Safety and immunogenicity of a delta inulin-adjuvanted inactivated Japanese encephalitis virus vaccine in pregnant mares and foals. Veterinary research. 2014;45:130.

[106] Warda FF, Ahmed HES, Shafik NG, Mikhael CA, Abd-ElAziz HMG, Mohammed WA, et al. Application of equine herpesvirus-1 vaccine inactivated by both formaldehyde and binary ethylenimine in equine. Veterinary world. 2021;14:1815-21.

[107] Wise LD, Wolf JJ, Kaplanski CV, Pauley CJ, Ledwith BJ. Lack of effects on fertility and developmental toxicity of a quadrivalent HPV vaccine in Sprague-Dawley rats. Birth defects research Part B, Developmental and reproductive toxicology. 2008;83:561-72.

[108] Segal L, Wilby OK, Willoughby CR, Veenstra S, Deschamps M. Evaluation of the intramuscular administration of Cervarix TM vaccine on fertility, pre- and post-natal development in rats. Reproductive toxicology (Elmsford, NY). 2011;31:111-20.

[109] Glenn GM, Fries LF, Smith G, Kpamegan E, Lu H, Guebre-Xabier M, et al. Modeling maternal fetal RSV F vaccine induced antibody transfer in guinea pigs. Special Issue: Advancing maternal immunization programs through research in low and medium income countries. 2015;33:6488-92.

[110] Heldens JGM, Pouwels HGW, Derks CGG, Van de Zande SMA, Hoeijmakers MJH. The first safe inactivated equine influenza vaccine formulation adjuvanted with ISCOM-Matrix that closes the immunity gap. Vaccine. 2009;27:5530-7.

[111] Shan C, Xie X, Luo H, Muruato AE, Liu Y, Wakamiya M, et al. Maternal vaccination and protective immunity against Zika virus vertical transmission. Nature Communications. 2019;10.

[112] Stedman A, Wright D, Schreur PJW, Clark MHA, Hill AVS, Gilbert SC, et al. Safety and efficacy of ChAdOx1 RVF vaccine against Rift Valley fever in pregnant sheep and goats. npj Vaccines. 2019;4.

[113] Magnus MC, Wilcox AJ, Morken N-H, Weinberg CR, Håberg SE. Role of maternal age and pregnancy history in risk of miscarriage: prospective register based study. BMJ. 2019;364:l869-l.

[114] Zeitlin J, Mortensen L, Prunet C, Macfarlane A, Hindori-Mohangoo AD, Gissler M, et al. Socioeconomic inequalities in stillbirth rates in Europe: measuring the gap using routine data from the Euro-Peristat Project. BMC Pregnancy Childbirth. 2016;16:15.

[115] Hug L, You D, Blencowe H, Mishra A, Wang Z, Fix MJ, et al. Global, regional, and national estimates and trends in stillbirths from 2000 to 2019: a systematic assessment. Lancet. 2021;398:772-85.

[116] Kingdon C, Roberts D, Turner MA, Storey C, Crossland N, Finlayson KW, et al. Inequalities and stillbirth in the UK: a meta-narrative review. BMJ Open. 2019;9:e029672-e.

[117] de Onis M, Blossner M, Villar J. Levels and patterns of intrauterine growth retardation in developing countries. Eur J Clin Nutr. 1998;52 Suppl 1:S5-15.

[118] ACOG Practice Bulletin No. 190: Gestational Diabetes Mellitus. Obstetrics and gynecology. 2018;131:e49-e64.

[119] Kuklina EV, Ayala C, Callaghan WM. Hypertensive disorders and severe obstetric morbidity in the United States. Obstetrics and gynecology. 2009;113:1299-306.

[120] Blencowe H, Krasevec J, de Onis M, Black RE, An X, Stevens GA, et al. National, regional, and worldwide estimates of low birthweight in 2015, with trends from 2000: a systematic analysis. The Lancet Global health. 2019;7:e849-e60.

[121] Lee ACC, Katz J, Blencowe H, Cousens S, Kozuki N, Vogel JP, et al. National and regional estimates of term and preterm babies born small for gestational age in 138 low-income and middle-income countries in 2010. The Lancet Global health. 2013;1:e26-e36.

[122] Chawanpaiboon S, Vogel JP, Moller AB, Lumbiganon P, Petzold M, Hogan D, et al. Global, regional, and national estimates of levels of preterm birth in 2014: a systematic review and modelling analysis. The Lancet Global health. 2019;7:e37-e46.

[123] Trends in maternal mortality 2000 to 2017: estimates. Geneva: World Health Organization; 2019.

[124] Mai CT, Isenburg JL, Canfield MA, Meyer RE, Correa A, Alverson CJ, et al. National population-based estimates for major birth defects, 2010-2014. Birth defects research. 2019;111:1420-35.

[125] UNICEF. Neonatal mortality. The neonatal period is the most vulnerable time for a child. 2020.

[126] Vogel JP, Oladapo OT, Dowswell T, Gülmezoglu AM. Updated WHO recommendation on intravenous tranexamic acid for the treatment of post-partum haemorrhage. The Lancet Global health. 2018;6:e18-e9.

[127] Prabhu M, Eckert LO, Belfort M, Babarinsa I, Ananth CV, Silver RM, et al. Antenatal bleeding: Case definition and guidelines for data collection, analysis, and presentation of immunization safety data. Vaccine. 2017;35:6529-37.

[128] Graham EM, Ruis KA, Hartman AL, Northington FJ, Fox HE. A systematic review of the role of intrapartum hypoxia-ischemia in the causation of neonatal encephalopathy. American journal of obstetrics and gynecology. 2008;199:587-95.

[129] Sivanandan S, Agarwal R, Sethi AJSif, medicine n. Respiratory distress in term neonates in low-resource settings. 2017;22 4:260-6.

[130] Sweet LR, Keech C, Klein NP, Marshall HS, Tagbo BN, Quine D, et al. Respiratory distress in the neonate: Case definition & guidelines for data collection, analysis, and presentation of maternal immunization safety data. Vaccine. 2017;35:6506-17.

[131] Ross E, Munoz FM, Edem B, Nan C, Jehan F, Quinn J, et al. Failure to thrive: Case definition & guidelines for data collection, analysis, and presentation of maternal immunisation safety data. Vaccine. 2017;35:6483-91.

[132] Morris JK, Rankin J, Garne E, Loane M, Greenlees R, Addor M-C, et al. Prevalence of microcephaly in Europe: population based study. BMJ. 2016;354:i4721.

[133] Cragan JD, Isenburg JL, Parker SE, Alverson CJ, Meyer RE, Stallings EB, et al. Population-based microcephaly surveillance in the United States, 2009 to 2013: An analysis of potential sources of variation. Birth defects research Part A, Clinical and molecular teratology. 2016;106:972-82.
