# Supplementary material for: Safety of COVID-19 vaccines during pregnancy: A systematic review and meta-analysis
Source: Vaccine. 2023 Jun 7;41(25):3688–700. doi: 10.1016/j.vaccine.2023.03.038 (PMC10040368; doi:10.1016/j.vaccine.2023.03.038)
Supplement: Supplementary data 2 [file mmc2.docx]

**Index Supplementary Material** **Appendix B** (with hyperlinks to specific sections)

[1 UNCONTROLLED STUDIES ASSESSING EXPOSURE VS NO EXPOSURE TO COVID-19 VACCINES IN PREGNANT PERSONS 3](#_Toc108382354)

[1.1 mRNA COVID-19 VACCINES IN HUMANS 3](#_Toc108382355)

[2 UNCONTROLLED STUDIES ASSESSING EXPOSURE VS NO EXPOSURE TO COVID-19 VACCINES IN PREGNANT ANIMALS 44](#_Toc108382356)

[2.1 mRNA VACCINES IN ANIMALS 44](#_Toc108382357)

[2.2 VECTORED VACCINES IN ANIMALS 46](#_Toc108382358)

[3 CONTROLLED STUDIES ASSESSING EXPOSURE VS NO EXPOSURE TO NON-COVID-19 VACCINES IN PREGNANT PERSONS 48](#_Toc108382359)

[3.1 EXPOSURE TO VACCINES CONTAINING AS03 VS. NO EXPOSURE 48](#_Toc108382360)

[Spontaneous abortion / miscarriage (aHR) 49](#_Toc108382361)

[Stillbirth (aHR) 49](#_Toc108382362)

[Stillbirth (aOR) 50](#_Toc108382363)

[Fetal death (aHR, aRR) 50](#_Toc108382364)

[Early neonatal death (aHR) 51](#_Toc108382365)

[Late neonatal death (aHR) 51](#_Toc108382366)

[Congenital malformations (aOR) 52](#_Toc108382367)

[Preterm birth (aHR) 52](#_Toc108382368)

[Preterm birth (aOR) 53](#_Toc108382369)

[Sensitivity analysis outcome Preterm birth (aOR) 54](#_Toc108382370)

[5-minute Apgar score (aRR & aHR) 55](#_Toc108382371)

[Low birth weight (aHR) 56](#_Toc108382372)

[Low birth weight (aOR) 57](#_Toc108382373)

[Small for gestational age <10th percentile (aRR) 58](#_Toc108382374)

[Fetal growth restriction (aHR) 58](#_Toc108382375)

[Gestational diabetes (aOR) 58](#_Toc108382376)

[Pre-eclampsia (aOR) 59](#_Toc108382377)

[3.2 EXPOSURE TO VACCINES CONTAINING ALUMINUM VS. NO EXPOSURE 60](#_Toc108382378)

[Low birth weight (aOR & aHR) 62](#_Toc108382379)

[Small for gestational age <10th percentile (aHR & aOR) 63](#_Toc108382380)

[Gestational diabetes (aHR) 63](#_Toc108382381)

[Gestational hypertension (aHR & aOR) 64](#_Toc108382382)

[Pre-eclampsia/eclampsia (aHR & aOR) 64](#_Toc108382383)

[Cesarean delivery (aOR) 65](#_Toc108382384)

[Postpartum hemorrhage (aRR) 65](#_Toc108382385)

[Chorioamnionitis/funisitis (aRR) 65](#_Toc108382386)

[4 UNCONTROLLED STUDIES ASSESSING EXPOSURE VS NO EXPOSURE TO NON-COVID-19 VACCINES IN PREGNANT PERSONS 66](#_Toc108382387)

[4.1 VACCINES CONTAINING AS03 ADJUVANTS IN HUMANS 66](#_Toc108382388)

[4.2 VACCINES CONTAINING ALUMINUM ADJUVANTS IN HUMANS 94](#_Toc108382389)

[5 VACCINES CONTAINING ALUMINUM IN ANIMALS & HUMANS + ANIMALS 164](#_Toc108382390)

## ***1 UNCONTROLLED STUDIES ASSESSING EXPOSURE VS NO EXPOSURE TO COVID-19 VACCINES IN PREGNANT PERSONS***

## ***1.1 mRNA COVID-19 VACCINES IN HUMANS***

**Proportion meta-analysis Hypertensive disorders of pregnancy**

Method: Stuart-Ord (inverse double arcsine square root)

| Stratum | Responding | Total |  |
| --- | --- | --- | --- |
| 1 | 0 | 13 | Gray 2021 |
| 2 | 1 | 57 | Bookstein 2021 |

| Stratum | Proportion | 95% CI (exact) | |  |
| --- | --- | --- | --- | --- |
| 1 | 0 | 0 | 0.247053 | Gray 2021 [97.5% one-sided CI] |
| 2 | 0.017544 | 0.000444 | 0.093917 | Bookstein 2021 |

| Stratum | Standardized effect | Variance | % Weights (fixed, random) | |  |
| --- | --- | --- | --- | --- | --- |
| 1 | 0 | 0.074074 | 19.014085 | 19.014085 | Gray 2021 [97.5% one-sided CI] |
| 2 | 0.017544 | 0.017391 | 80.985915 | 80.985915 | Bookstein 2021 |

Fixed effects (inverse variance)

Pooled proportion = 0.023735 (95% CI = 0.001472 to 0.07165)

Non-combinability of studies

Cochran Q = 0.025102 (df = 1) P = 0.8741

Moment-based estimate of between studies variance = 0

I_2_ (inconsistency) = 0% (95% CI = *% to *%)

Random effects (DerSimonian-Laird)

Pooled proportion = 0.023735 (95% CI = 0.001472 to 0.07165)

Bias indicators

Begg-Mazumdar: Kendall's <too few strata> P = *

Egger: bias = * (95% CI = * to *) P = *

Harbord: bias = -0.795404 (92.5% CI = * to *) P = *

**Proportion meta-analysis Maternal death**

Method: Stuart-Ord (inverse double arcsine square root)

| Stratum | Responding | Total |  |
| --- | --- | --- | --- |
| 1 | 0 | 7530 | Goldstein 2021 |

| Stratum | Proportion | 95% CI (exact) | |  |
| --- | --- | --- | --- | --- |
| 1 | 0 | 0 | 0.00049 | Goldstein 2021 [97.5% one-sided CI] |

| Stratum | Standardized effect | Variance | % Weights (fixed, random) | |  |
| --- | --- | --- | --- | --- | --- |
| 1 | 0 | 0.000133 | 100 | 100 | Goldstein 2021 [97.5% one-sided CI] |

Fixed effects (inverse variance)

Pooled proportion = 0 (95% CI = 0 to 0.000291)

Non-combinability of studies

Cochran Q = 0 (df = 0) P = *

Moment-based estimate of between studies variance = 0

I_2_ (inconsistency) = *% (95% CI = *% to *%)

Random effects (DerSimonian-Laird)

Pooled proportion = 0.000033 (95% CI = 0.000031 to 0.000291)

Bias indicators

Begg-Mazumdar: Kendall's <too few strata> P = *

Egger: bias = * (95% CI = * to *) P = *

Harbord: bias = * (92.5% CI = * to *) P = *

**Proportion meta-analysis postpartum hemorrhage**

Method: Stuart-Ord (inverse double arcsine square root)

| Stratum | Responding | Total |  |
| --- | --- | --- | --- |
| 1 | 6 | 57 | Bookstein 2021 |
| 2 | 13 | 133 | Blakeway 2021 |

| Stratum | Proportion | 95% CI (exact) | |  |
| --- | --- | --- | --- | --- |
| 1 | 0.105263 | 0.039622 | 0.215164 | Bookstein 2021 |
| 2 | 0.097744 | 0.053078 | 0.161345 | Blakeway 2021 |

| Stratum | Standardized effect | Variance | % Weights (fixed, random) | |  |
| --- | --- | --- | --- | --- | --- |
| 1 | 0.105263 | 0.017391 | 30.104712 | 30.104712 | Bookstein 2021 |
| 2 | 0.097744 | 0.007491 | 69.895288 | 69.895288 | Blakeway 2021 |

Fixed effects (inverse variance)

Pooled proportion = 0.104035 (95% CI = 0.064857 to 0.151163)

Non-combinability of studies

Cochran Q = 0.053172 (df = 1) P = 0.8176

Moment-based estimate of between studies variance = 0

I_2_ (inconsistency) = 0% (95% CI = *% to *%)

Random effects (DerSimonian-Laird)

Pooled proportion = 0.104035 (95% CI = 0.064857 to 0.151163)

Bias indicators

Begg-Mazumdar: Kendall's <too few strata> P = *

Egger: bias = * (95% CI = * to *) P = *

Harbord: bias = 0.538389 (92.5% CI = * to *) P = *

**Proportion meta-analysis: Abortion**

Method: Stuart-Ord (inverse double arcsine square root)

| Stratum | Responding | Total |  |
| --- | --- | --- | --- |
| 1 | 1128 | 20139 | Kharbanda 2021 USA |
| 2 | 165 | 2456 | Zauche 2021 (datos de v-safe) |
| USAEE3 | 0 | 57 | Bookstein 2021 Israel |
| 4 | 128 | 7530 | Goldstein 2021 Israel |
| 5 | 104 | 827 | Shimabukuro 2021 (T2D) USA |

| Stratum | Proportion | 95% CI (exact) | |  |
| --- | --- | --- | --- | --- |
| 1 | 0.056011 | 0.052874 | 0.059276 | Kharbanda 2021 |
| 2 | 0.067182 | 0.057599 | 0.077813 | Zauche 2021 (datos de v-safe) |
| 3 | 0 | 0 | 0.062667 | Bookstein 2021 [97.5% one-sided CI] |
| 4 | 0.016999 | 0.014201 | 0.020179 | Goldstein 2021 |
| 5 | 0.125756 | 0.103919 | 0.150299 | Shimabukuro 2021 (T2D) |

| Stratum | Standardized effect | Variance | % Weights (fixed, random) | |  |
| --- | --- | --- | --- | --- | --- |
| 1 | 0.056011 | 0.00005 | 64.942038 | 22.251253 | Kharbanda 2021 |
| 2 | 0.067182 | 0.000407 | 7.921255 | 21.900758 | Zauche 2021 (datos de v-safe) |
| 3 | 0 | 0.017391 | 0.185415 | 12.525569 | Bookstein 2021 [97.5% one-sided CI] |
| 4 | 0.016999 | 0.000133 | 24.282927 | 22.168729 | Goldstein 2021 |
| 5 | 0.125756 | 0.001208 | 2.668365 | 21.153691 | Shimabukuro 2021 (T2D) |

Fixed effects (inverse variance)

Pooled proportion = 0.046615 (95% CI = 0.044297 to 0.04899)

Non-combinability of studies

Cochran Q = 358.034652 (df = 4) P < 0.0001

Moment-based estimate of between studies variance = 0.022284

I_2_ (inconsistency) = 98.9% (95% CI = 98.6% to 99.1%)

Random effects (DerSimonian-Laird)

Pooled proportion = 0.049685 (95% CI = 0.024049 to 0.083905)

Bias indicators

Begg-Mazumdar: Kendall's 0 P = 0.8167

Egger: bias = 4.717014 (95% CI = -20.127469 to 29.561498) P = 0.5883

Harbord: bias = 2.359806 (92.5% CI = -18.116827 to 22.83644) P = 0.7776

**Proportion meta-analysis Small for Gestational Age**

Method: Stuart-Ord (inverse double arcsine square root)

| Stratum | Responding | Total |  |
| --- | --- | --- | --- |
| 1 | 16 | 133 | Blakeway 2021 |

| Stratum | Proportion | 95% CI (exact) | |  |
| --- | --- | --- | --- | --- |
| 1 | 0.120301 | 0.07035 | 0.188 | Blakeway 2021 |

| Stratum | Standardized effect | Variance | % Weights (fixed, random) | |  |
| --- | --- | --- | --- | --- | --- |
| 1 | 0.120301 | 0.007491 | 100 | 100 | Blakeway 2021 |

Fixed effects (inverse variance)

Pooled proportion = 0.12311 (95% CI = 0.073052 to 0.183987)

Non-combinability of studies

Cochran Q = 0 (df = 0) P = *

Moment-based estimate of between studies variance = 0

I_2_ (inconsistency) = *% (95% CI = *% to *%)

Random effects (DerSimonian-Laird)

Pooled proportion = 0.12311 (95% CI = 0.073052 to 0.183987)

Bias indicators

Begg-Mazumdar: Kendall's <too few strata> P = *

Egger: bias = * (95% CI = * to *) P = *

Harbord: bias = * (92.5% CI = * to *) P = *

**Proportion meta-analysis Suspected Chorioamnionitis**

Method: Stuart-Ord (inverse double arcsine square root)

| Stratum | Responding | Total |  |
| --- | --- | --- | --- |
| 1 | 0 | 133 | Blakeway 2021 |

| Stratum | Proportion | 95% CI (exact) | |  |
| --- | --- | --- | --- | --- |
| 1 | 0 | 0 | 0.027355 | Blakeway 2021 [97.5% one-sided CI] |

| Stratum | Standardized effect | Variance | % Weights (fixed, random) | |  |
| --- | --- | --- | --- | --- | --- |
| 1 | 0 | 0.007491 | 100 | 100 | Blakeway 2021 [97.5% one-sided CI] |

Fixed effects (inverse variance)

Pooled proportion = 0 (95% CI = 0 to 0.016311)

Non-combinability of studies

Cochran Q = 0 (df = 0) P = *

Moment-based estimate of between studies variance = 0

I_2_ (inconsistency) = *% (95% CI = *% to *%)

Random effects (DerSimonian-Laird)

Pooled proportion = 0.001869 (95% CI = 0.001727 to 0.016311)

Bias indicators

Begg-Mazumdar: Kendall's <too few strata> P = *

Egger: bias = * (95% CI = * to *) P = *

Harbord: bias = * (92.5% CI = * to *) P = *

**Proportion meta-analysis antenatal bleeding**

Method: Stuart-Ord (inverse double arcsine square root)

| Stratum | Responding | Total |  |
| --- | --- | --- | --- |
| 1 | 1 | 390 | Bookstein 2021 |

| Stratum | Proportion | 95% CI (exact) | |  |
| --- | --- | --- | --- | --- |
| 1 | 0.002564 | 0.000065 | 0.014203 | Bookstein 2021 |

| Stratum | Standardized effect | Variance | % Weights (fixed, random) | |  |
| --- | --- | --- | --- | --- | --- |
| 1 | 0.002564 | 0.002561 | 100 | 100 | Bookstein 2021 |

Fixed effects (inverse variance)

Pooled proportion = 0.003727 (95% CI = 0.000132 to 0.0122)

Non-combinability of studies

Cochran Q = 0 (df = 0) P = *

Moment-based estimate of between studies variance = 0

I_2_ (inconsistency) = *% (95% CI = *% to *%)

Random effects (DerSimonian-Laird)

Pooled proportion = 0.003727 (95% CI = 0.000132 to 0.0122)

Bias indicators

Begg-Mazumdar: Kendall's <too few strata> P = *

Egger: bias = * (95% CI = * to *) P = *

Harbord: bias = * (92.5% CI = * to *) P = *

**Proportion meta-analysis gestational diabetes**

Method: Stuart-Ord (inverse double arcsine square root)

| Stratum | Responding | Total |  |
| --- | --- | --- | --- |
| 1 | 4 | 57 | Bookstein 2021 |

| Stratum | Proportion | 95% CI (exact) | |  |
| --- | --- | --- | --- | --- |
| 1 | 0.070175 | 0.01945 | 0.17004 | Bookstein 2021 |

| Stratum | Standardized effect | Variance | % Weights (fixed, random) | |  |
| --- | --- | --- | --- | --- | --- |
| 1 | 0.070175 | 0.017391 | 100 | 100 | Bookstein 2021 |

Fixed effects (inverse variance)

Pooled proportion = 0.077366 (95% CI = 0.023115 to 0.159695)

Non-combinability of studies

Cochran Q = 0 (df = 0) P = *

Moment-based estimate of between studies variance = 0

I_2_ (inconsistency) = *% (95% CI = *% to *%)

Random effects (DerSimonian-Laird)

Pooled proportion = 0.077366 (95% CI = 0.023115 to 0.159695)

Bias indicators

Begg-Mazumdar: Kendall's <too few strata> P = *

Egger: bias = * (95% CI = * to *) P = *

Harbord: bias = * (92.5% CI = * to *) P = *

**Proportion meta-analysis fetal growth retardation**

Method: Stuart-Ord (inverse double arcsine square root)

| Stratum | Responding | Total |  |
| --- | --- | --- | --- |
| 1 | 0 | 13 | Gray 2021 |
| 2 | 36 | 7530 | Goldstein 2021 |

| Stratum | Proportion | 95% CI (exact) | |  |
| --- | --- | --- | --- | --- |
| 1 | 0 | 0 | 0.247053 | Gray 2021 [97.5% one-sided CI] |
| 2 | 0.004781 | 0.003351 | 0.006613 | Goldstein 2021 |

| Stratum | Standardized effect | Variance | % Weights (fixed, random) | |  |
| --- | --- | --- | --- | --- | --- |
| 1 | 0 | 0.074074 | 0.17895 | 0.17895 | Gray 2021 [97.5% one-sided CI] |
| 2 | 0.004781 | 0.000133 | 99.82105 | 99.82105 | Goldstein 2021 |

Fixed effects (inverse variance)

Pooled proportion = 0.004863 (95% CI = 0.003419 to 0.006558)

Non-combinability of studies

Cochran Q = 0.231982 (df = 1) P = 0.6301

Moment-based estimate of between studies variance = 0

I_2_ (inconsistency) = 0% (95% CI = *% to *%)

Random effects (DerSimonian-Laird)

Pooled proportion = 0.004863 (95% CI = 0.003419 to 0.006558)

Bias indicators

Begg-Mazumdar: Kendall's <too few strata> P = *

Egger: bias = * (95% CI = * to *) P = *

Harbord: bias = -0.25854 (92.5% CI = * to *) P = *

**Proportion meta-analysis Stillbirth**

Method: Stuart-Ord (inverse double arcsine square root)

| Stratum | Responding | Total |  |
| --- | --- | --- | --- |
| 1 | 1 | 7530 | Goldstein 2021 |
| 2 | 1 | 725 | Shimabukuro 2021 (T2D) |
| 3 | 0 | 133 | Blakeway 2021 |

| Stratum | Proportion | 95% CI (exact) | |  |
| --- | --- | --- | --- | --- |
| 1 | 0.000133 | 0.000003 | 0.00074 | Goldstein 2021 |
| 2 | 0.001379 | 0.000035 | 0.007661 | Shimabukuro 2021 (T2D) |
| 3 | 0 | 0 | 0.027355 | Blakeway 2021 [97.5% one-sided CI] |

| Stratum | Standardized effect | Variance | % Weights (fixed, random) | |  |
| --- | --- | --- | --- | --- | --- |
| 1 | 0.000133 | 0.000133 | 89.761011 | 68.950172 | Goldstein 2021 |
| 2 | 0.001379 | 0.001378 | 8.647714 | 24.995225 | Shimabukuro 2021 (T2D) |
| 3 | 0 | 0.007491 | 1.591275 | 6.054603 | Blakeway 2021 [97.5% one-sided CI] |

Fixed effects (inverse variance)

Pooled proportion = 0.000291 (95% CI = 0.00004 to 0.00077)

Non-combinability of studies

Cochran Q = 2.900771 (df = 2) P = 0.2345

Moment-based estimate of between studies variance = 0.000576

I_2_ (inconsistency) = 31.1% (95% CI = 0% to 80.4%)

Random effects (DerSimonian-Laird)

Pooled proportion = 0.000548 (95% CI = 0.000003 to 0.00203)

Bias indicators

Begg-Mazumdar: Kendall's <too few strata> P = *

Egger: bias = * (95% CI = * to *) P = *

Harbord: bias = 0.975928 (92.5% CI = -11.555018 to 13.506874) P = 0.6295

**Proportion meta-analysis Preterm birth**

Method: Stuart-Ord (inverse double arcsine square root)

| Stratum | Responding | Total |  |
| --- | --- | --- | --- |
| 1 | 1 | 13 | Gray 2021 |
| 2 | 0 | 57 | Bookstein 2021 |
| 3 | 77 | 1387 | Goldstein 2021 |
| 4 | 60 | 636 | Shimabukuro 2021 (T2D) |

| Stratum | Proportion | 95% CI (exact) | |  |
| --- | --- | --- | --- | --- |
| 1 | 0.076923 | 0.001946 | 0.360297 | Gray 2021 |
| 2 | 0 | 0 | 0.062667 | Bookstein 2021 [97.5% one-sided CI] |
| 3 | 0.055516 | 0.044057 | 0.068899 | Goldstein 2021 |
| 4 | 0.09434 | 0.072764 | 0.119765 | Shimabukuro 2021 (T2D) |

| Stratum | Standardized effect | Variance | % Weights (fixed, random) | |  |
| --- | --- | --- | --- | --- | --- |
| 1 | 0.076923 | 0.074074 | 0.644391 | 7.020756 | Gray 2021 |
| 2 | 0 | 0.017391 | 2.74463 | 18.959977 | Bookstein 2021 [97.5% one-sided CI] |
| 3 | 0.055516 | 0.000721 | 66.229117 | 37.930597 | Goldstein 2021 |
| 4 | 0.09434 | 0.001571 | 30.381862 | 36.08867 | Shimabukuro 2021 (T2D) |

Fixed effects (inverse variance)

Pooled proportion = 0.064656 (95% CI = 0.054528 to 0.075582)

Non-combinability of studies

Cochran Q = 18.638043 (df = 3) P = 0.0003

Moment-based estimate of between studies variance = 0.015941

I_2_ (inconsistency) = 83.9% (95% CI = 44.6% to 92%)

Random effects (DerSimonian-Laird)

Pooled proportion = 0.056101 (95% CI = 0.025769 to 0.097188)

Bias indicators

Begg-Mazumdar: Kendall's 0 P = 0.75

Egger: bias = -0.391899 (95% CI = -13.297286 to 12.513488) P = 0.908

Harbord: bias = -0.374222 (92.5% CI = -8.286282 to 7.537837) P = 0.8856

**Proportion meta-analysis Congenital malformation**

Method: Stuart-Ord (inverse double arcsine square root)

| Stratum | Responding | Total |  |
| --- | --- | --- | --- |
| 1 | 16 | 724 | Shimabukuro 2021 (T2D) |
| 2 | 3 | 133 | Blakeway 2021 |

| Stratum | Proportion | 95% CI (exact) | |  |
| --- | --- | --- | --- | --- |
| 1 | 0.022099 | 0.012683 | 0.035641 | Shimabukuro 2021 (T2D) |
| 2 | 0.022556 | 0.004676 | 0.064504 | Blakeway 2021 |

| Stratum | Standardized effect | Variance | % Weights (fixed, random) | |  |
| --- | --- | --- | --- | --- | --- |
| 1 | 0.022099 | 0.00138 | 84.440559 | 84.440559 | Shimabukuro 2021 (T2D) |
| 2 | 0.022556 | 0.007491 | 15.559441 | 15.559441 | Blakeway 2021 |

Fixed effects (inverse variance)

Pooled proportion = 0.023243 (95% CI = 0.014236 to 0.034384)

Non-combinability of studies

Cochran Q = 0.049685 (df = 1) P = 0.8236

Moment-based estimate of between studies variance = 0

I_2_ (inconsistency) = 0% (95% CI = *% to *%)

Random effects (DerSimonian-Laird)

Pooled proportion = 0.023243 (95% CI = 0.014236 to 0.034384)

Bias indicators

Begg-Mazumdar: Kendall's <too few strata> P = *

Egger: bias = * (95% CI = * to *) P = *

Harbord: bias = 0.061208 (92.5% CI = * to *) P = *

**Proportion meta-analysis Neonatal infections**

Method: Stuart-Ord (inverse double arcsine square root)

| Stratum | Responding | Total |  |
| --- | --- | --- | --- |
| 1 | 23 | 724 | Shimabukuro 2021 (T2D) |

| Stratum | Proportion | 95% CI (exact) | |  |
| --- | --- | --- | --- | --- |
| 1 | 0.031768 | 0.020243 | 0.047288 | Shimabukuro 2021 (T2D) |

| Stratum | Standardized effect | Variance | % Weights (fixed, random) | |  |
| --- | --- | --- | --- | --- | --- |
| 1 | 0.031768 | 0.00138 | 100 | 100 | Shimabukuro 2021 (T2D) |

Fixed effects (inverse variance)

Pooled proportion = 0.03241 (95% CI = 0.020766 to 0.046533)

Non-combinability of studies

Cochran Q = 0 (df = 0) P = *

Moment-based estimate of between studies variance = 0

I_2_ (inconsistency) = *% (95% CI = *% to *%)

Random effects (DerSimonian-Laird)

Pooled proportion = 0.03241 (95% CI = 0.020766 to 0.046533)

Bias indicators

Begg-Mazumdar: Kendall's <too few strata> P = *

Egger: bias = * (95% CI = * to *) P = *

Harbord: bias = * (92.5% CI = * to *) P = *

**Proportion meta-analysis Neonatal death**

Method: Stuart-Ord (inverse double arcsine square root)

| Stratum | Responding | Total |  |
| --- | --- | --- | --- |
| 1 | 0 | 57 | Bookstein 2021 |

| Stratum | Proportion | 95% CI (exact) | |  |
| --- | --- | --- | --- | --- |
| 1 | 0 | 0 | 0.062667 | Bookstein 2021 [97.5% one-sided CI] |

| Stratum | Standardized effect | Variance | % Weights (fixed, random) | |  |
| --- | --- | --- | --- | --- | --- |
| 1 | 0 | 0.017391 | 100 | 100 | Bookstein 2021 [97.5% one-sided CI] |

Fixed effects (inverse variance)

Pooled proportion = 0 (95% CI = 0 to 0.037576)

Non-combinability of studies

Cochran Q = 0 (df = 0) P = *

Moment-based estimate of between studies variance = 0

I_2_ (inconsistency) = *% (95% CI = *% to *%)

Random effects (DerSimonian-Laird)

Pooled proportion = 0.004329 (95% CI = 0.004013 to 0.037576)

Bias indicators

Begg-Mazumdar: Kendall's <too few strata> P = *

Egger: bias = * (95% CI = * to *) P = *

Harbord: bias = * (92.5% CI = * to *) P = *

**Proportion meta-analysis Respiratory distress in the newborn**

Method: Stuart-Ord (inverse double arcsine square root)

| Stratum | Responding | Total |  |
| --- | --- | --- | --- |
| 1 | 0 | 13 | Gray 2021 |

| Stratum | Proportion | 95% CI (exact) | |  |
| --- | --- | --- | --- | --- |
| 1 | 0 | 0 | 0.247053 | Gray 2021 [97.5% one-sided CI] |

| Stratum | Standardized effect | Variance | % Weights (fixed, random) | |  |
| --- | --- | --- | --- | --- | --- |
| 1 | 0 | 0.074074 | 100 | 100 | Gray 2021 [97.5% one-sided CI] |

Fixed effects (inverse variance)

Pooled proportion = 0 (95% CI = 0 to 0.153079)

Non-combinability of studies

Cochran Q = 0 (df = 0) P = *

Moment-based estimate of between studies variance = 0

I_2_ (inconsistency) = *% (95% CI = *% to *%)

Random effects (DerSimonian-Laird)

Pooled proportion = 0.018188 (95% CI = 0.017178 to 0.153079)

Bias indicators

Begg-Mazumdar: Kendall's <too few strata> P = *

Egger: bias = * (95% CI = * to *) P = *

Harbord: bias = * (92.5% CI = * to *) P = *

**Proportion meta-analysis injection site reactions**

Method: Stuart-Ord (inverse double arcsine square root)

| Stratum | Responding | Total |  |
| --- | --- | --- | --- |
| 1 | 26 | 77 | Gray 2021 |
| 2 | 358 | 390 | Bookstein 2021 |
| 3 | 4 | 57 | Goldstein 2021 |
| 4 | 11274 | 12273 | Shimabukuro 2021 (T2D) |

| Stratum | Proportion | 95% CI (exact) | |  |
| --- | --- | --- | --- | --- |
| 1 | 0.337662 | 0.233776 | 0.454459 | Gray 2021 |
| 2 | 0.917949 | 0.886142 | 0.943201 | Bookstein 2021 |
| 3 | 0.070175 | 0.01945 | 0.17004 | Goldstein 2021 |
| 4 | 0.918602 | 0.913623 | 0.923381 | Shimabukuro 2021 (T2D) |

| Stratum | Standardized effect | Variance | % Weights (fixed, random) | |  |
| --- | --- | --- | --- | --- | --- |
| 1 | 0.337662 | 0.012903 | 0.605516 | 24.670266 | Gray 2021 |
| 2 | 0.917949 | 0.002561 | 3.05102 | 25.390233 | Bookstein 2021 |
| 3 | 0.070175 | 0.017391 | 0.449254 | 24.370386 | Goldstein 2021 |
| 4 | 0.918602 | 0.000081 | 95.89421 | 25.569116 | Shimabukuro 2021 (T2D) |

Fixed effects (inverse variance)

Pooled proportion = 0.913816 (95% CI = 0.908892 to 0.918615)

Non-combinability of studies

Cochran Q = 360.734825 (df = 3) P < 0.0001

Moment-based estimate of between studies variance = 0.35183

I_2_ (inconsistency) = 99.2% (95% CI = 99% to 99.3%)

Random effects (DerSimonian-Laird)

Pooled proportion = 0.588555 (95% CI = 0.300751 to 0.84662)

Bias indicators

Begg-Mazumdar: Kendall's -0.666667 P = 0.0833

Egger: bias = -12.453053 (95% CI = -41.20085 to 16.294745) P = 0.2034

Harbord: bias = -16.079273 (92.5% CI = -41.065948 to 8.907402) P = 0.1571

**Proportion meta-analysis Fever**

Method: Stuart-Ord (inverse double arcsine square root)

| Stratum | Responding | Total |  |
| --- | --- | --- | --- |
| 1 | 25 | 77 | Gray 2021 |
| 2 | 6 | 390 | Bookstein 2021 |
| 3 | 4242 | 12273 | Shimabukuro 2021 (T2D) |

| Stratum | Proportion | 95% CI (exact) | |  |
| --- | --- | --- | --- | --- |
| 1 | 0.324675 | 0.222317 | 0.440987 | Gray 2021 |
| 2 | 0.015385 | 0.005666 | 0.033184 | Bookstein 2021 |
| 3 | 0.345637 | 0.33722 | 0.354127 | Shimabukuro 2021 (T2D) |

| Stratum | Standardized effect | Variance | % Weights (fixed, random) | |  |
| --- | --- | --- | --- | --- | --- |
| 1 | 0.324675 | 0.012903 | 0.608249 | 32.724967 | Gray 2021 |
| 2 | 0.015385 | 0.002561 | 3.064788 | 33.537152 | Bookstein 2021 |
| 3 | 0.345637 | 0.000081 | 96.326963 | 33.73788 | Shimabukuro 2021 (T2D) |

Fixed effects (inverse variance)

Pooled proportion = 0.331056 (95% CI = 0.32291 to 0.339252)

Non-combinability of studies

Cochran Q = 377.383247 (df = 2) P < 0.0001

Moment-based estimate of between studies variance = 0.414161

I_2_ (inconsistency) = 99.5% (95% CI = 99.3% to 99.6%)

Random effects (DerSimonian-Laird)

Pooled proportion = 0.192769 (95% CI = 0.007757 to 0.535475)

Bias indicators

Begg-Mazumdar: Kendall's <too few strata> P = *

Egger: bias = * (95% CI = * to *) P = *

Harbord: bias = -7.597612 (92.5% CI = -77.455476 to 62.260253) P = 0.5269

**Proportion meta-analysis headache**

Method: Stuart-Ord (inverse double arcsine square root)

| Stratum | Responding | Total |  |
| --- | --- | --- | --- |
| 1 | 25 | 77 | Gray 2021 |
| 2 | 18 | 390 | Bookstein 2021 |
| 3 | 10 | 57 | Goldstein 2021 |
| 4 | 6800 | 12273 | Shimabukuro 2021 (T2D) |

| Stratum | Proportion | 95% CI (exact) | |  |
| --- | --- | --- | --- | --- |
| 1 | 0.324675 | 0.222317 | 0.440987 | Gray 2021 |
| 2 | 0.046154 | 0.02758 | 0.071964 | Bookstein 2021 |
| 3 | 0.175439 | 0.087473 | 0.299058 | Goldstein 2021 |
| 4 | 0.554062 | 0.545215 | 0.562883 | Shimabukuro 2021 (T2D) |

| Stratum | Standardized effect | Variance | % Weights (fixed, random) | |  |
| --- | --- | --- | --- | --- | --- |
| 1 | 0.324675 | 0.012903 | 0.605516 | 24.811149 | Gray 2021 |
| 2 | 0.046154 | 0.002561 | 3.05102 | 25.225994 | Bookstein 2021 |
| 3 | 0.175439 | 0.017391 | 0.449254 | 24.635343 | Goldstein 2021 |
| 4 | 0.554062 | 0.000081 | 95.89421 | 25.327513 | Shimabukuro 2021 (T2D) |

Fixed effects (inverse variance)

Pooled proportion = 0.53202 (95% CI = 0.523371 to 0.540659)

Non-combinability of studies

Cochran Q = 629.338217 (df = 3) P < 0.0001

Moment-based estimate of between studies variance = 0.616

I_2_ (inconsistency) = 99.5% (95% CI = 99.4% to 99.6%)

Random effects (DerSimonian-Laird)

Pooled proportion = 0.253131 (95% CI = 0.019499 to 0.627494)

Bias indicators

Begg-Mazumdar: Kendall's 0 P = 0.75

Egger: bias = -16.146537 (95% CI = -94.689704 to 62.396629) P = 0.4697

Harbord: bias = -10.5442 (92.5% CI = -31.90179 to 10.81339) P = 0.2313

**Proportion meta-analysis gastrointestinal**

Method: Stuart-Ord (inverse double arcsine square root)

| Stratum | Responding | Total |  |
| --- | --- | --- | --- |
| 1 | 5 | 57 | Goldstein 2021 |

| Stratum | Proportion | 95% CI (exact) | |  |
| --- | --- | --- | --- | --- |
| 1 | 0.087719 | 0.029099 | 0.192957 | Goldstein 2021 |

| Stratum | Standardized effect | Variance | % Weights (fixed, random) | |  |
| --- | --- | --- | --- | --- | --- |
| 1 | 0.087719 | 0.017391 | 100 | 100 | Goldstein 2021 |

Fixed effects (inverse variance)

Pooled proportion = 0.094652 (95% CI = 0.033293 to 0.182941)

Non-combinability of studies

Cochran Q = 0 (df = 0) P = *

Moment-based estimate of between studies variance = 0

I_2_ (inconsistency) = *% (95% CI = *% to *%)

Random effects (DerSimonian-Laird)

Pooled proportion = 0.094652 (95% CI = 0.033293 to 0.182941)

Bias indicators

Begg-Mazumdar: Kendall's <too few strata> P = *

Egger: bias = * (95% CI = * to *) P = *

Harbord: bias = * (92.5% CI = * to *) P = *

**Proportion meta-analysis myalgia**

Method: Stuart-Ord (inverse double arcsine square root)

| Stratum | Responding | Total |  |
| --- | --- | --- | --- |
| 1 | 37 | 77 | Gray 2021 |
| 2 | 23 | 390 | Bookstein 2021 |
| 3 | 6638 | 12273 | Shimabukuro 2021 (T2D) |

| Stratum | Proportion | 95% CI (exact) | |  |
| --- | --- | --- | --- | --- |
| 1 | 0.480519 | 0.365177 | 0.597405 | Gray 2021 |
| 2 | 0.058974 | 0.037748 | 0.087179 | Bookstein 2021 |
| 3 | 0.540862 | 0.531996 | 0.549709 | Shimabukuro 2021 (T2D) |

| Stratum | Standardized effect | Variance | % Weights (fixed, random) | |  |
| --- | --- | --- | --- | --- | --- |
| 1 | 0.480519 | 0.012903 | 0.608249 | 32.879021 | Gray 2021 |
| 2 | 0.058974 | 0.002561 | 3.064788 | 33.486056 | Bookstein 2021 |
| 3 | 0.540862 | 0.000081 | 96.326963 | 33.634923 | Shimabukuro 2021 (T2D) |

Fixed effects (inverse variance)

Pooled proportion = 0.522797 (95% CI = 0.514121 to 0.531466)

Non-combinability of studies

Cochran Q = 507.408638 (df = 2) P < 0.0001

Moment-based estimate of between studies variance = 0.557618

I_2_ (inconsistency) = 99.6% (95% CI = 99.5% to 99.7%)

Random effects (DerSimonian-Laird)

Pooled proportion = 0.330778 (95% CI = 0.035051 to 0.741296)

Bias indicators

Begg-Mazumdar: Kendall's <too few strata> P = *

Egger: bias = * (95% CI = * to *) P = *

Harbord: bias = -10.764947 (92.5% CI = -104.390558 to 82.860664) P = 0.5092

**Proportion meta-analysis fatigue**

Method: Stuart-Ord (inverse double arcsine square root)

| Stratum | Responding | Total |  |
| --- | --- | --- | --- |
| 1 | 41 | 77 | Gray 2021 |
| 2 | 100 | 390 | Bookstein 2021 |
| 3 | 8772 | 12273 | Shimabukuro 2021 (T2D) |

| Stratum | Proportion | 95% CI (exact) | |  |
| --- | --- | --- | --- | --- |
| 1 | 0.532468 | 0.415213 | 0.647149 | Gray 2021 |
| 2 | 0.25641 | 0.213784 | 0.302778 | Bookstein 2021 |
| 3 | 0.71474 | 0.70666 | 0.722717 | Shimabukuro 2021 (T2D) |

| Stratum | Standardized effect | Variance | % Weights (fixed, random) | |  |
| --- | --- | --- | --- | --- | --- |
| 1 | 0.532468 | 0.012903 | 0.608249 | 32.681767 | Gray 2021 |
| 2 | 0.25641 | 0.002561 | 3.064788 | 33.551417 | Bookstein 2021 |
| 3 | 0.71474 | 0.000081 | 96.326963 | 33.766816 | Shimabukuro 2021 (T2D) |

Fixed effects (inverse variance)

Pooled proportion = 0.70041 (95% CI = 0.692427 to 0.708333)

Non-combinability of studies

Cochran Q = 351.959322 (df = 2) P < 0.0001

Moment-based estimate of between studies variance = 0.386111

I_2_ (inconsistency) = 99.4% (95% CI = 99.3% to 99.5%)

Random effects (DerSimonian-Laird)

Pooled proportion = 0.500299 (95% CI = 0.175155 to 0.825299)

Bias indicators

Begg-Mazumdar: Kendall's <too few strata> P = *

Egger: bias = * (95% CI = * to *) P = *

Harbord: bias = -12.628553 (92.5% CI = -98.686537 to 73.429432) P = 0.4321

**Proportion meta-analysis Vomiting**

Method: Stuart-Ord (inverse double arcsine square root)

| Stratum | Responding | Total |  |
| --- | --- | --- | --- |
| 1 | 558 | 12273 | Shimabukuro 2021 (T2D) |

| Stratum | Proportion | 95% CI (exact) | |  |
| --- | --- | --- | --- | --- |
| 1 | 0.045466 | 0.041847 | 0.049301 | Shimabukuro 2021 (T2D) |

| Stratum | Standardized effect | Variance | % Weights (fixed, random) | |  |
| --- | --- | --- | --- | --- | --- |
| 1 | 0.045466 | 0.000081 | 100 | 100 | Shimabukuro 2021 (T2D) |

Fixed effects (inverse variance)

Pooled proportion = 0.045503 (95% CI = 0.041887 to 0.049261)

Non-combinability of studies

Cochran Q = 0 (df = 0) P = *

Moment-based estimate of between studies variance = 0

I_2_ (inconsistency) = *% (95% CI = *% to *%)

Random effects (DerSimonian-Laird)

Pooled proportion = 0.045503 (95% CI = 0.041887 to 0.049261)

Bias indicators

Begg-Mazumdar: Kendall's <too few strata> P = *

Egger: bias = * (95% CI = * to *) P = *

Harbord: bias = * (92.5% CI = * to *) P = *

**Proportion meta-analysis Nausea**

Method: Stuart-Ord (inverse double arcsine square root)

| Stratum | Responding | Total |  |
| --- | --- | --- | --- |
| 1 | 3265 | 12273 | Shimabukuro 2021 (T2D) |

| Stratum | Proportion | 95% CI (exact) | |  |
| --- | --- | --- | --- | --- |
| 1 | 0.266031 | 0.258229 | 0.273944 | Shimabukuro 2021 (T2D) |

| Stratum | Standardized effect | Variance | % Weights (fixed, random) | |  |
| --- | --- | --- | --- | --- | --- |
| 1 | 0.266031 | 0.000081 | 100 | 100 | Shimabukuro 2021 (T2D) |

Fixed effects (inverse variance)

Pooled proportion = 0.26605 (95% CI = 0.25827 to 0.273904)

Non-combinability of studies

Cochran Q = 0 (df = 0) P = *

Moment-based estimate of between studies variance = 0

I_2_ (inconsistency) = *% (95% CI = *% to *%)

Random effects (DerSimonian-Laird)

Pooled proportion = 0.26605 (95% CI = 0.25827 to 0.273904)

Bias indicators

Begg-Mazumdar: Kendall's <too few strata> P = *

Egger: bias = * (95% CI = * to *) P = *

Harbord: bias = * (92.5% CI = * to *) P = *

**Proportion meta-analysis Chills**

Method: Stuart-Ord (inverse double arcsine square root)

| Stratum | Responding | Total |  |
| --- | --- | --- | --- |
| 1 | 25 | 77 | Gray 2021 |
| 2 | 4502 | 12273 | Shimabukuro 2021 (T2D) |

| Stratum | Proportion | 95% CI (exact) | |  |
| --- | --- | --- | --- | --- |
| 1 | 0.324675 | 0.222317 | 0.440987 | Gray 2021 |
| 2 | 0.366821 | 0.358287 | 0.375419 | Shimabukuro 2021 (T2D) |

| Stratum | Standardized effect | Variance | % Weights (fixed, random) | |  |
| --- | --- | --- | --- | --- | --- |
| 1 | 0.324675 | 0.012903 | 0.62748 | 0.62748 | Gray 2021 |
| 2 | 0.366821 | 0.000081 | 99.37252 | 99.37252 | Shimabukuro 2021 (T2D) |

Fixed effects (inverse variance)

Pooled proportion = 0.366579 (95% CI = 0.358102 to 0.375097)

Non-combinability of studies

Cochran Q = 0.542294 (df = 1) P = 0.4615

Moment-based estimate of between studies variance = 0

I_2_ (inconsistency) = 0% (95% CI = *% to *%)

Random effects (DerSimonian-Laird)

Pooled proportion = 0.366579 (95% CI = 0.358102 to 0.375097)

Bias indicators

Begg-Mazumdar: Kendall's <too few strata> P = *

Egger: bias = * (95% CI = * to *) P = *

Harbord: bias = -0.833512 (92.5% CI = * to *) P = *

**Proportion meta-analysis Joint Pain**

Method: Stuart-Ord (inverse double arcsine square root)

| Stratum | Responding | Total |  |
| --- | --- | --- | --- |
| 1 | 3138 | 12273 | Shimabukuro 2021 (T2D) |

| Stratum | Proportion | 95% CI (exact) | |  |
| --- | --- | --- | --- | --- |
| 1 | 0.255683 | 0.247983 | 0.263499 | Shimabukuro 2021 (T2D) |

| Stratum | Standardized effect | Variance | % Weights (fixed, random) | |  |
| --- | --- | --- | --- | --- | --- |
| 1 | 0.255683 | 0.000081 | 100 | 100 | Shimabukuro 2021 (T2D) |

Fixed effects (inverse variance)

Pooled proportion = 0.255703 (95% CI = 0.248024 to 0.263459)

Non-combinability of studies

Cochran Q = 0 (df = 0) P = *

Moment-based estimate of between studies variance = 0

I_2_ (inconsistency) = *% (95% CI = *% to *%)

Random effects (DerSimonian-Laird)

Pooled proportion = 0.255703 (95% CI = 0.248024 to 0.263459)

Bias indicators

Begg-Mazumdar: Kendall's <too few strata> P = *

Egger: bias = * (95% CI = * to *) P = *

Harbord: bias = * (92.5% CI = * to *) P = *

## **2 *UNCONTROLLED STUDIES ASSESSING EXPOSURE VS NO EXPOSURE TO COVID-19 VACCINES IN PREGNANT ANIMALS***

## ***2.1 mRNA VACCINES IN ANIMALS***

**Proportion meta-analysis any congenital malformation**

Method: Stuart-Ord (inverse double arcsine square root)

| Stratum | Responding | Total |  |
| --- | --- | --- | --- |
| 1 | 21 | 276 | Bowman 2021 |

| Stratum | Proportion | 95% CI (exact) | |  |
| --- | --- | --- | --- | --- |
| 1 | 0.076087 | 0.047714 | 0.113957 | Bowman 2021 |

| Stratum | Standardized effect | Variance | % Weights (fixed, random) | |  |
| --- | --- | --- | --- | --- | --- |
| 1 | 0.076087 | 0.003617 | 100 | 100 | Bowman 2021 |

Fixed effects (inverse variance)

Pooled proportion = 0.077608 (95% CI = 0.049075 to 0.112002)

Non-combinability of studies

Cochran Q = 0 (df = 0) P = *

Moment-based estimate of between studies variance = 0

I_2_ (inconsistency) = *% (95% CI = *% to *%)

Random effects (DerSimonian-Laird)

Pooled proportion = 0.077608 (95% CI = 0.049075 to 0.112002)

Bias indicators

Begg-Mazumdar: Kendall's <too few strata> P = *

Egger: bias = * (95% CI = * to *) P = *

Harbord: bias = * (92.5% CI = * to *) P = *

## ***2.2* *VECTORED VACCINES IN ANIMALS***

**Proportion meta-analysis abortion in animals**

Method: Stuart-Ord (inverse double arcsine square root)

| Stratum | Responding | Total |  |
| --- | --- | --- | --- |
| 1 | 0 | 16 | Stedman 2019 |
| 2 | 1 | 25 | Stebbings 2021 |

| Stratum | Proportion | 95% CI (exact) | |  |
| --- | --- | --- | --- | --- |
| 1 | 0 | 0 | 0.205907 | Stedman 2019 [97.5% one-sided CI] |
| 2 | 0.04 | 0.001012 | 0.203517 | Stebbings 2021 |

| Stratum | Standardized effect | Variance | % Weights (fixed, random) | |  |
| --- | --- | --- | --- | --- | --- |
| 1 | 0 | 0.060606 | 39.285714 | 39.285714 | Stedman 2019 [97.5% one-sided CI] |
| 2 | 0.04 | 0.039216 | 60.714286 | 60.714286 | Stebbings 2021 |

Fixed effects (inverse variance)

Pooled proportion = 0.036924 (95% CI = 0.001775 to 0.114105)

Non-combinability of studies

Cochran Q = 0.545971 (df = 1) P = 0.46

Moment-based estimate of between studies variance = 0

I_2_ (inconsistency) = 0% (95% CI = *% to *%)

Random effects (DerSimonian-Laird)

Pooled proportion = 0.036924 (95% CI = 0.001775 to 0.114105)

Bias indicators

Begg-Mazumdar: Kendall's <too few strata> P = *

Egger: bias = * (95% CI = * to *) P = *

Harbord: bias = -4.242342 (92.5% CI = * to *) P = *

**Proportion meta-analysis any congenital malformation in animals**

Method: Stuart-Ord (inverse double arcsine square root)

| Stratum | Responding | Total |  |
| --- | --- | --- | --- |
| 1 | 2 | 340 | Stebbings 2021 |

| Stratum | Proportion | 95% CI (exact) | |  |
| --- | --- | --- | --- | --- |
| 1 | 0.005882 | 0.000713 | 0.021086 | Stebbings 2021 |

| Stratum | Standardized effect | Variance | % Weights (fixed, random) | |  |
| --- | --- | --- | --- | --- | --- |
| 1 | 0.005882 | 0.002937 | 100 | 100 | Stebbings 2021 |

Fixed effects (inverse variance)

Pooled proportion = 0.007258 (95% CI = 0.001036 to 0.019034)

Non-combinability of studies

Cochran Q = 0 (df = 0) P = *

Moment-based estimate of between studies variance = 0

I_2_ (inconsistency) = *% (95% CI = *% to *%)

Random effects (DerSimonian-Laird)

Pooled proportion = 0.007258 (95% CI = 0.001036 to 0.019034)

Bias indicators

Begg-Mazumdar: Kendall's <too few strata> P = *

Egger: bias = * (95% CI = * to *) P = *

Harbord: bias = * (92.5% CI = * to *) P = *

## ***3 CONTROLLED STUDIES ASSESSING EXPOSURE VS NO EXPOSURE TO NON-COVID-19 VACCINES IN PREGNANT PERSONS***

## ***3.1 EXPOSURE TO VACCINES CONTAINING AS03 VS. NO EXPOSURE***

## **Spontaneous abortion / miscarriage (aHR)**

**
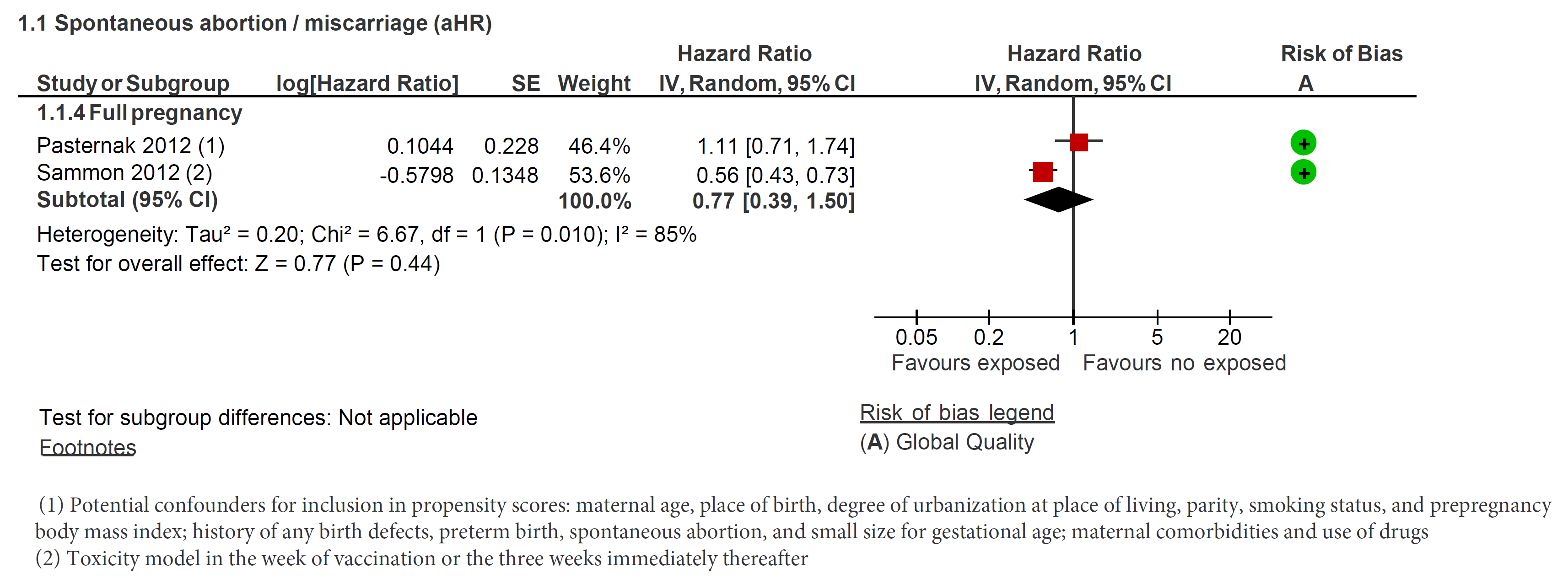
**

## **Stillbirth (aHR)**

**
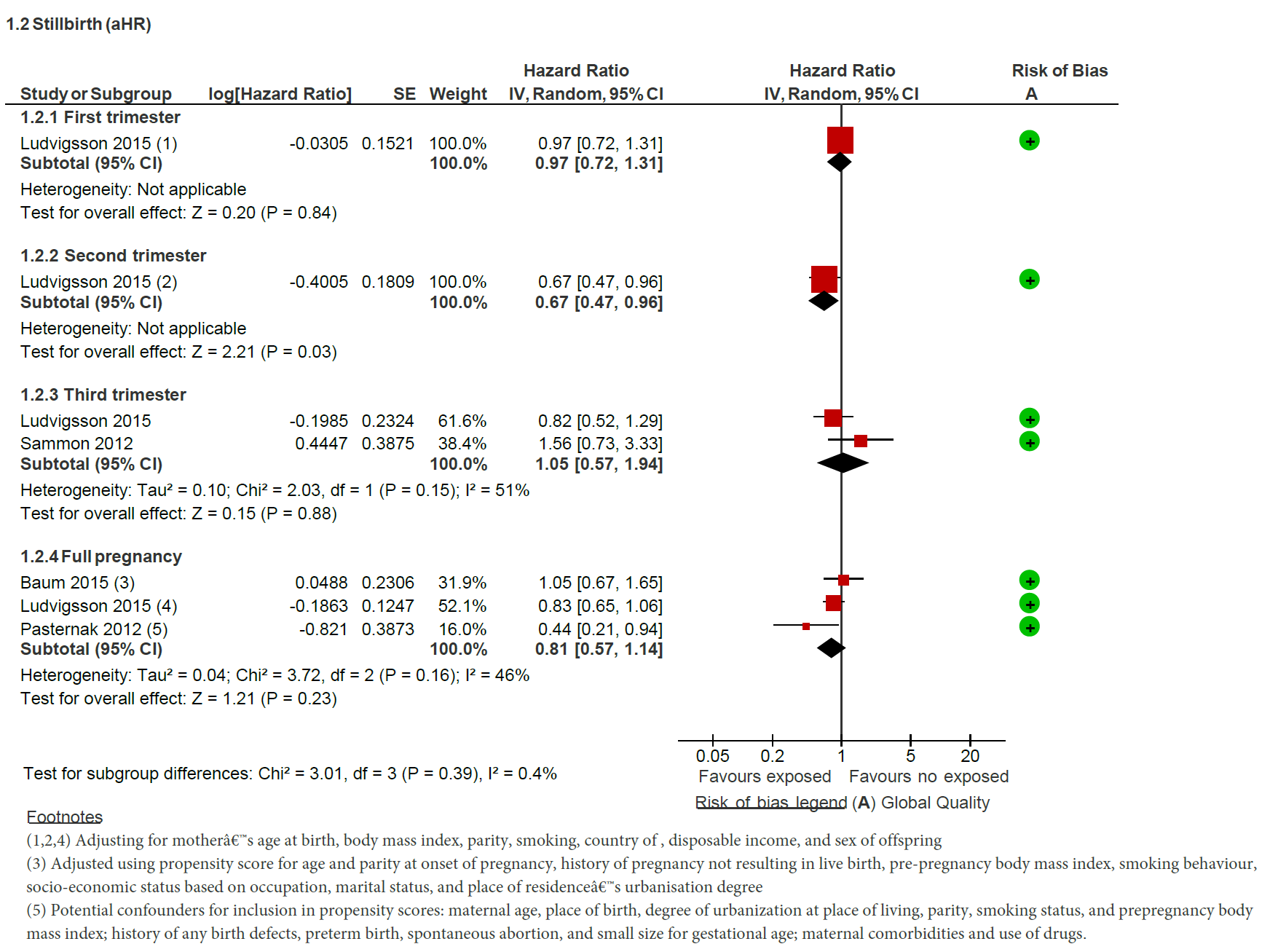
**

## **Stillbirth (aOR)**

**
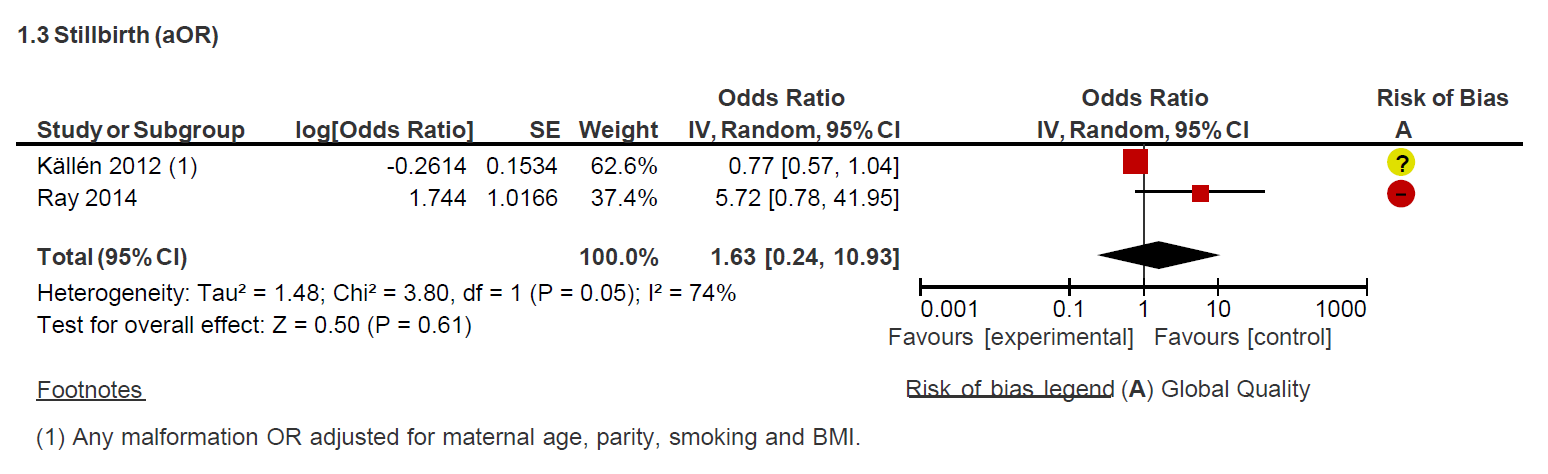
**

**
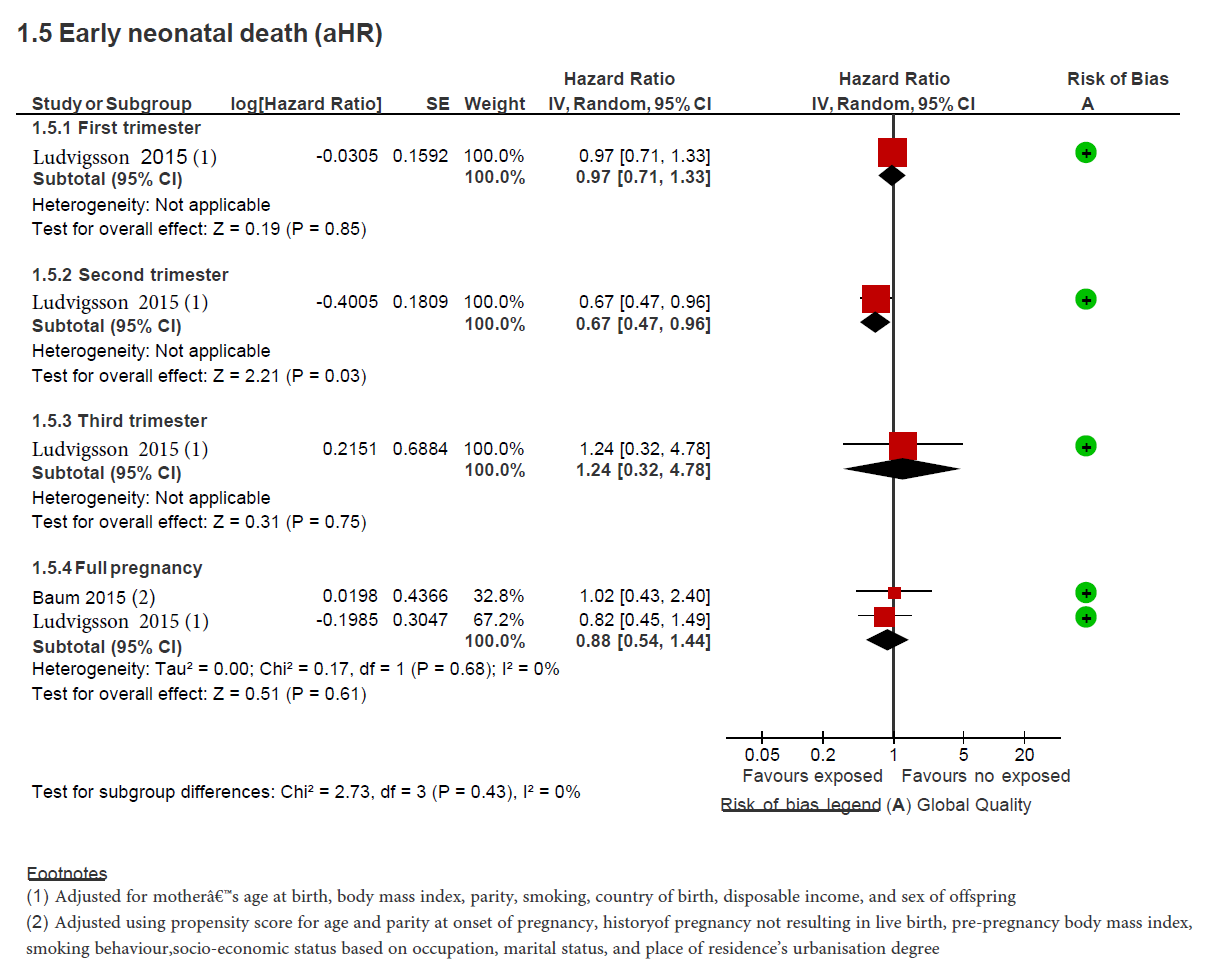
**

## **Fetal death (aHR, aRR)**

**
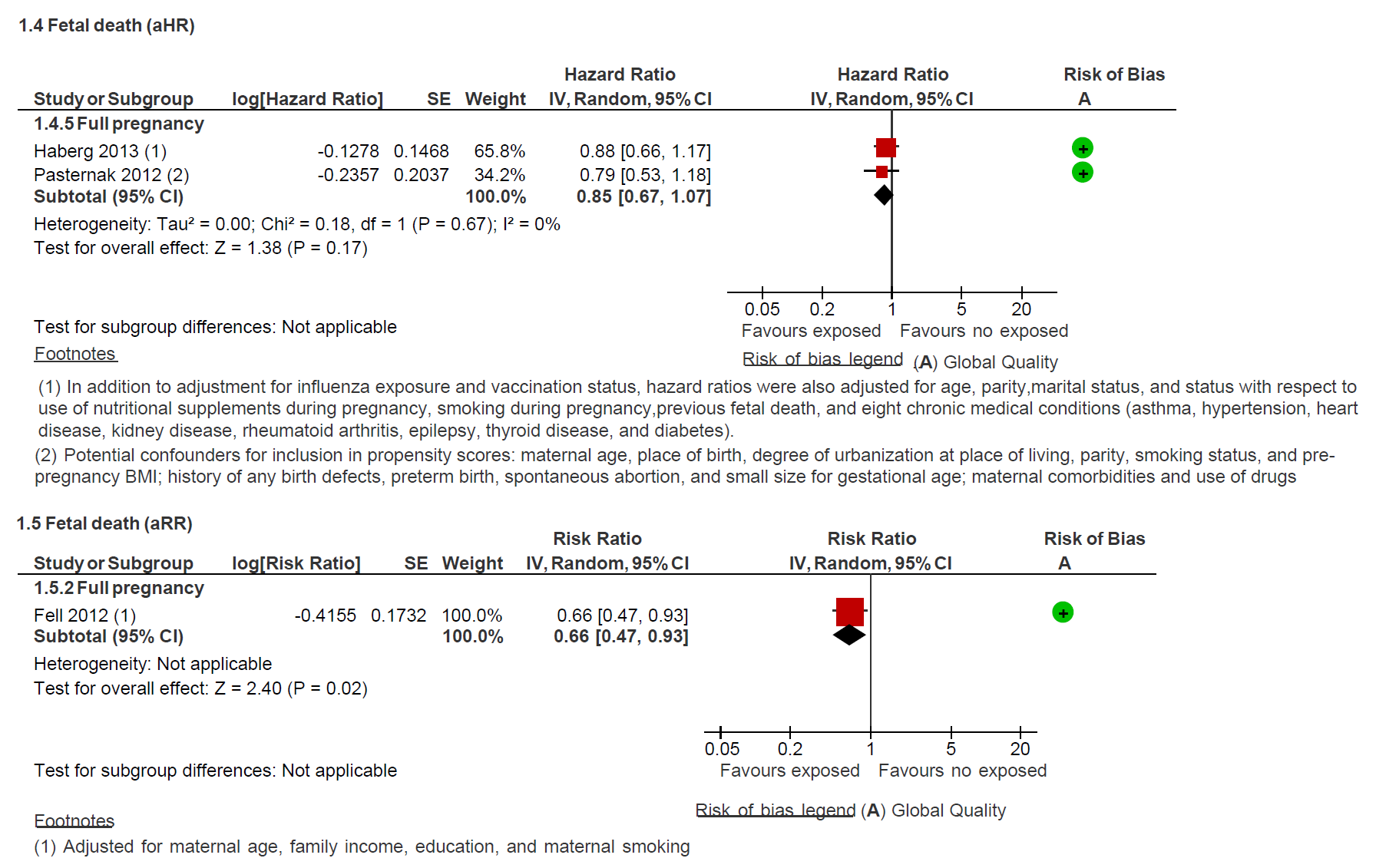
**

## **Early neonatal death (aHR)**

**
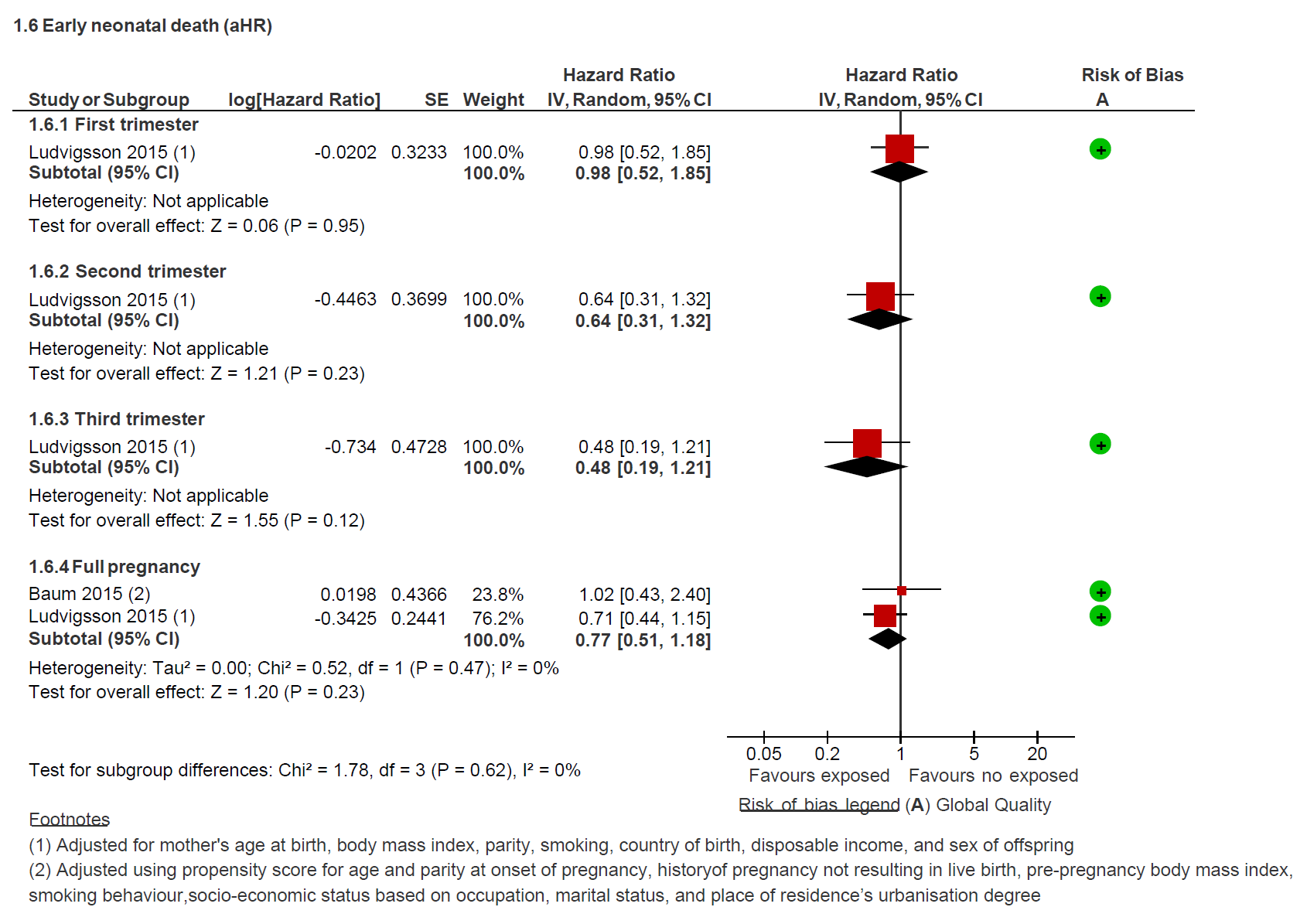
**

## **Late neonatal death (aHR)**

**
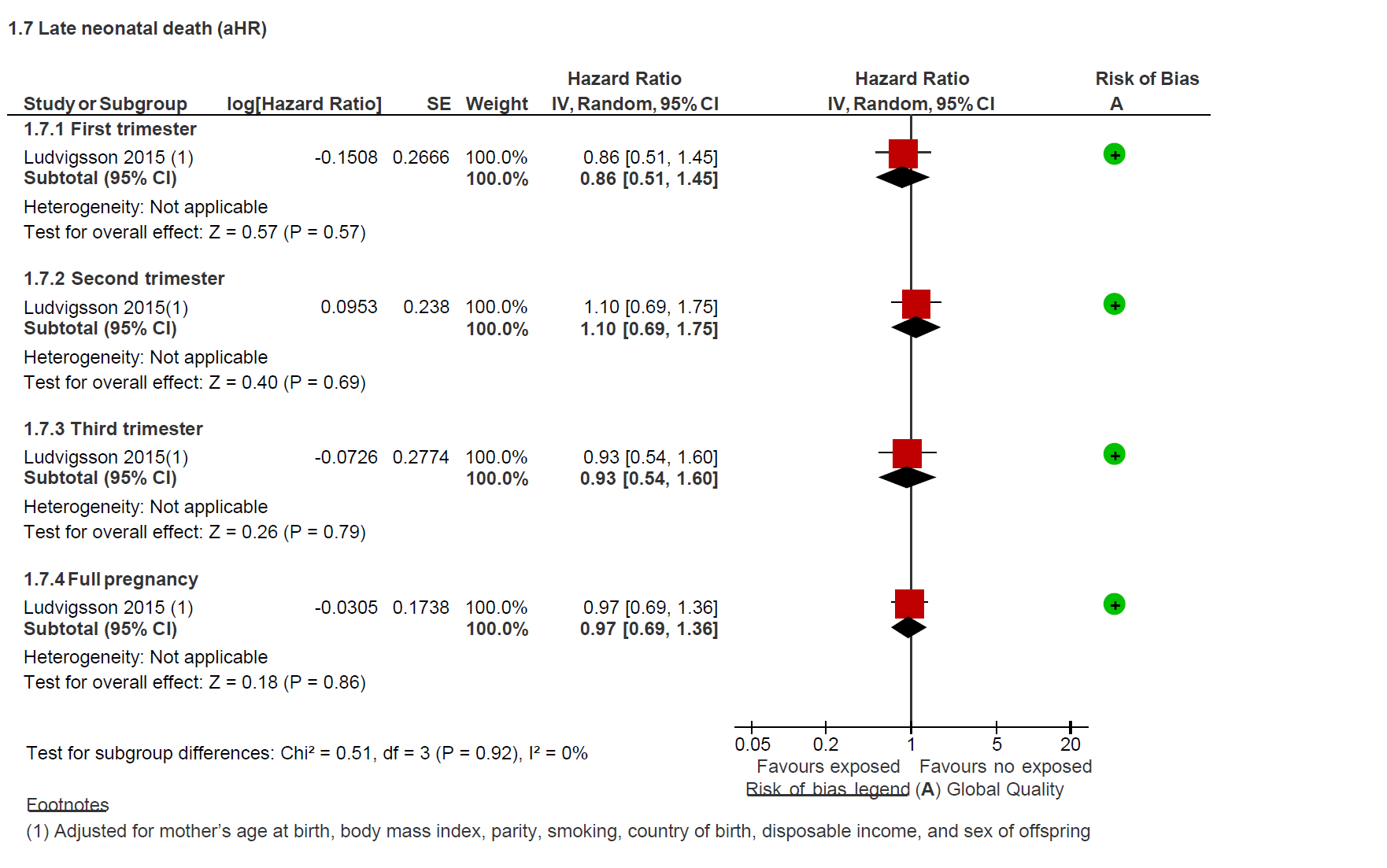
**

## **Congenital malformations (aOR)**

**
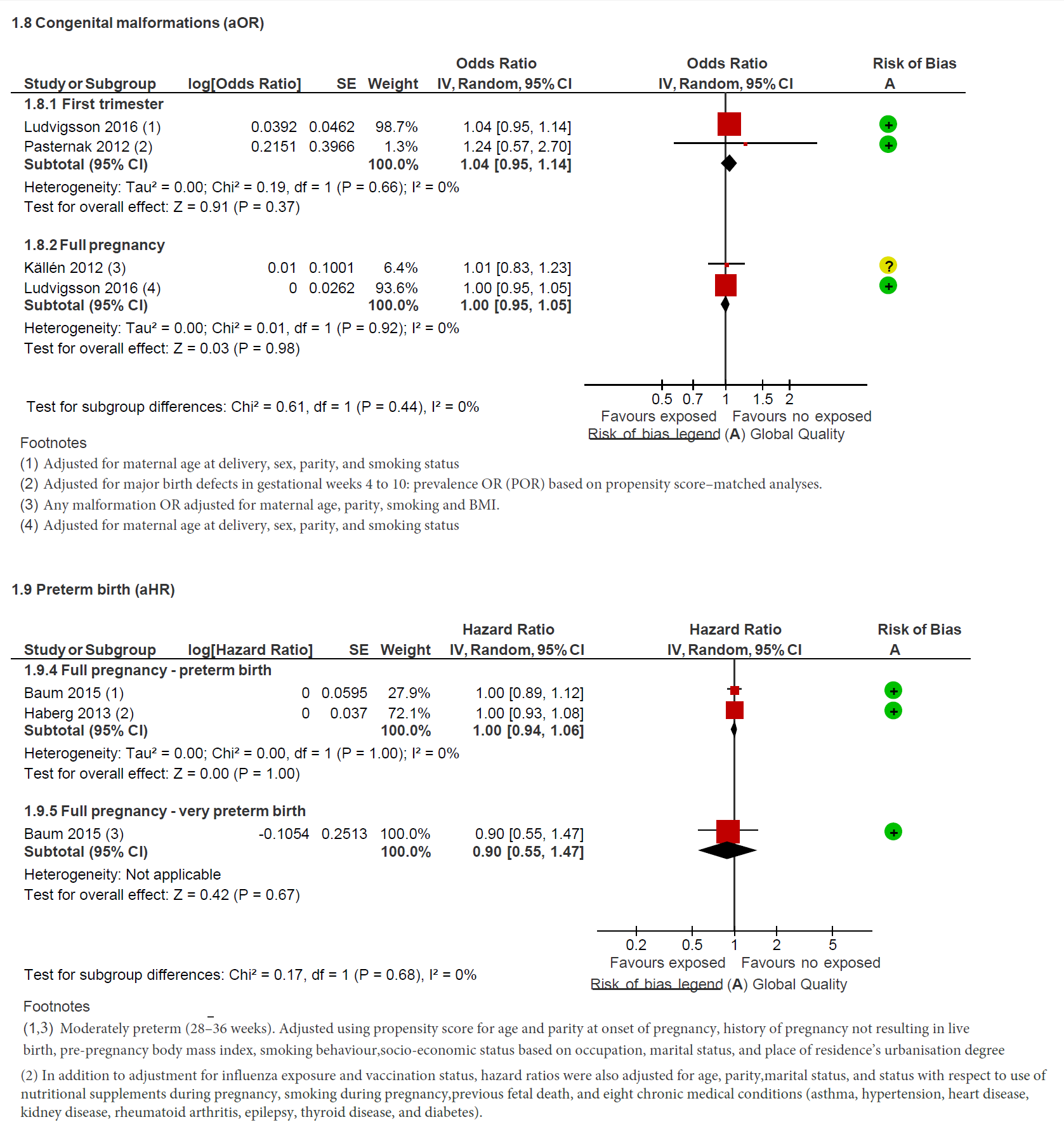
**

## **Preterm birth (aHR)**

**
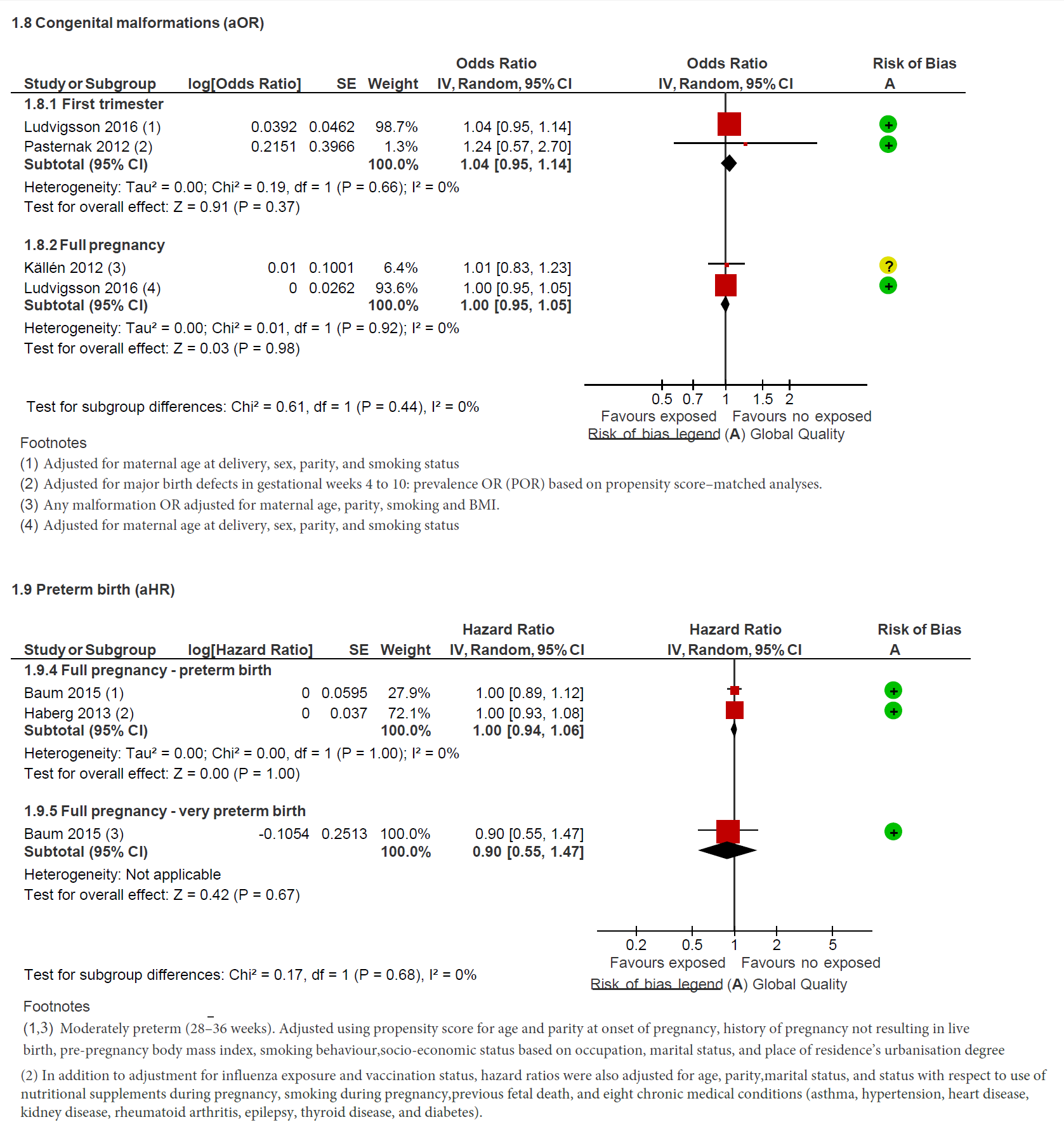
**

## **Preterm birth (aOR)**

**
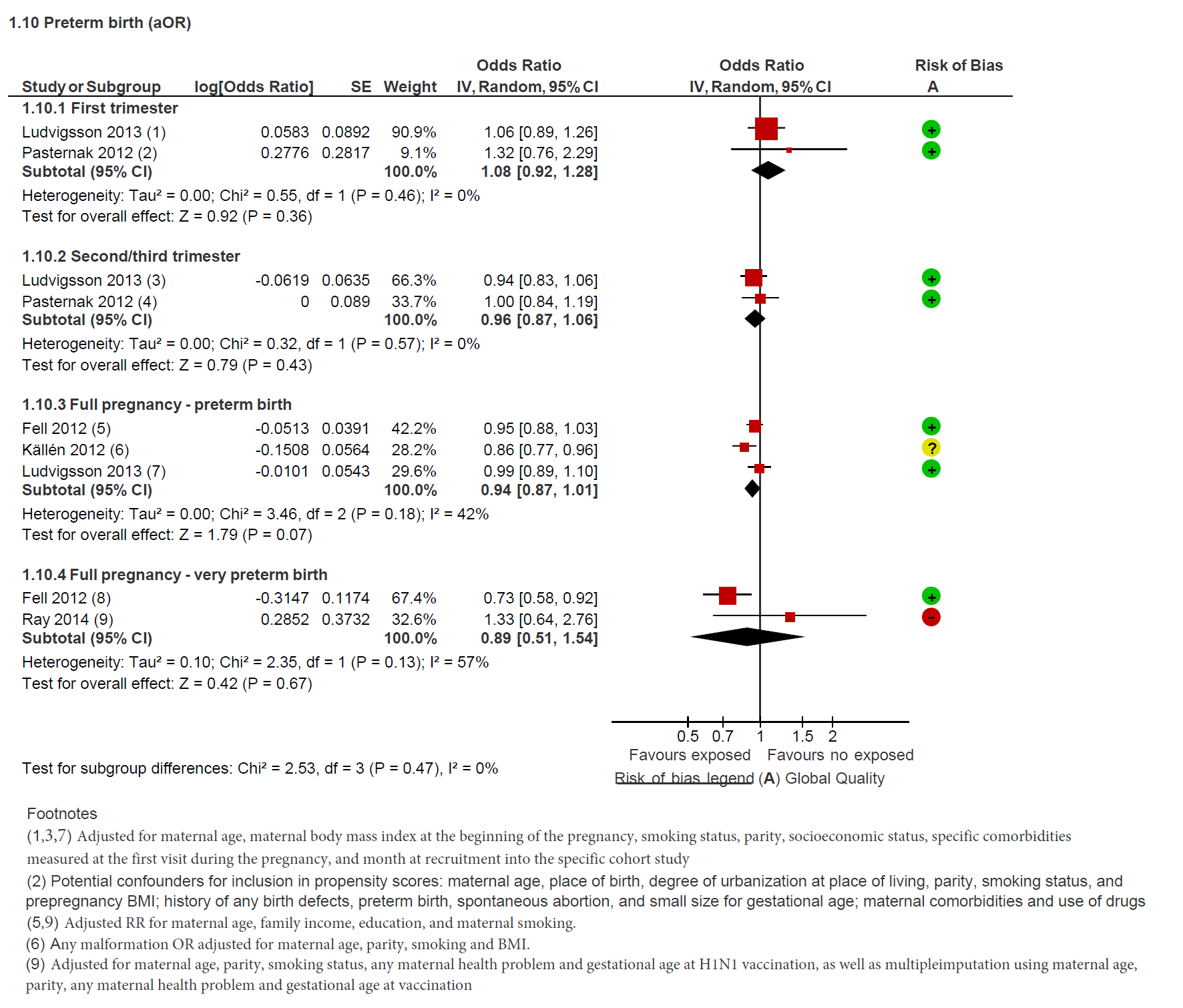
**

## **Sensitivity analysis outcome Preterm birth (aOR)**

**
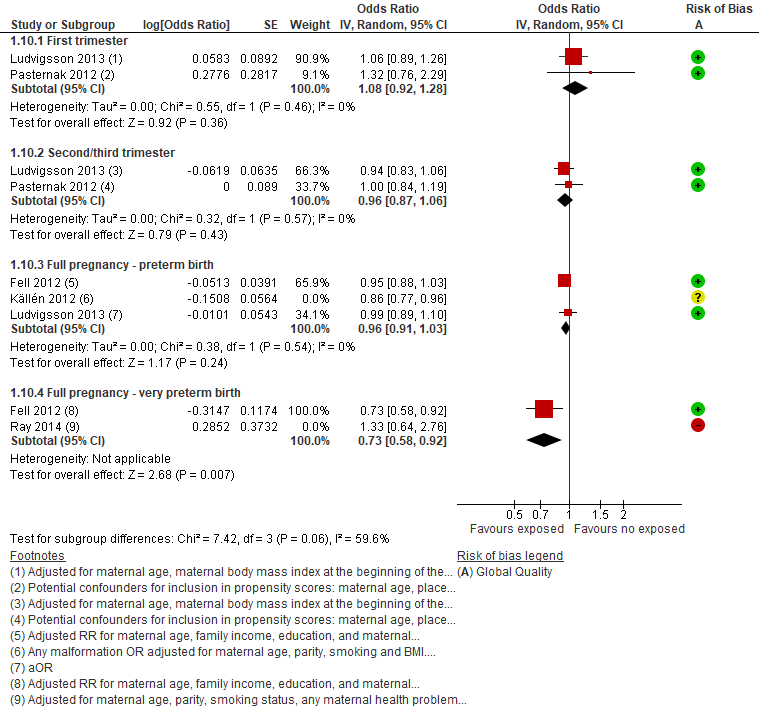
**

## **5-minute Apgar score (aRR & aHR)**

**
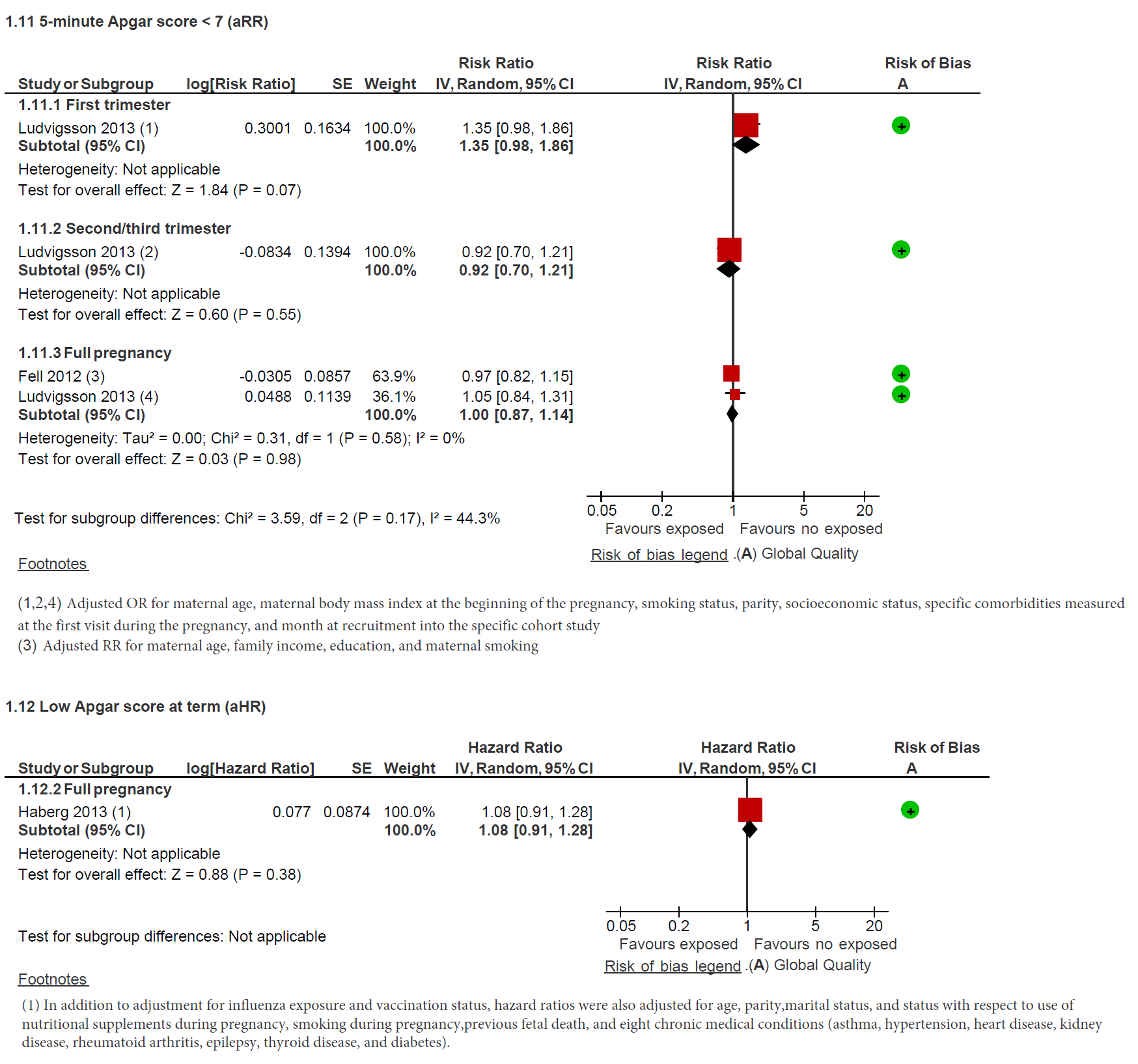
**

## **Low birth weight (aHR)**

**
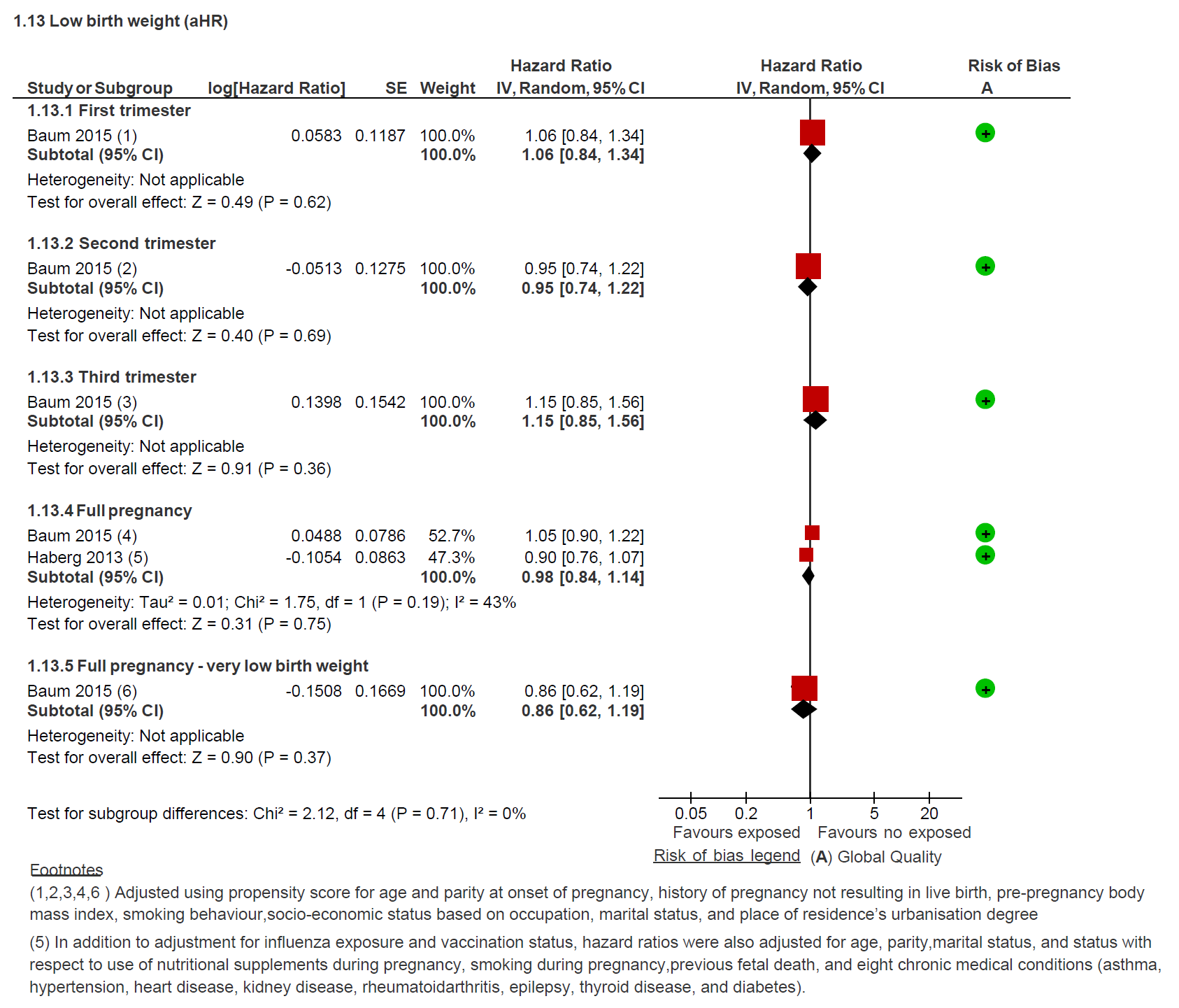
**

## **Low birth weight (aOR)**

**
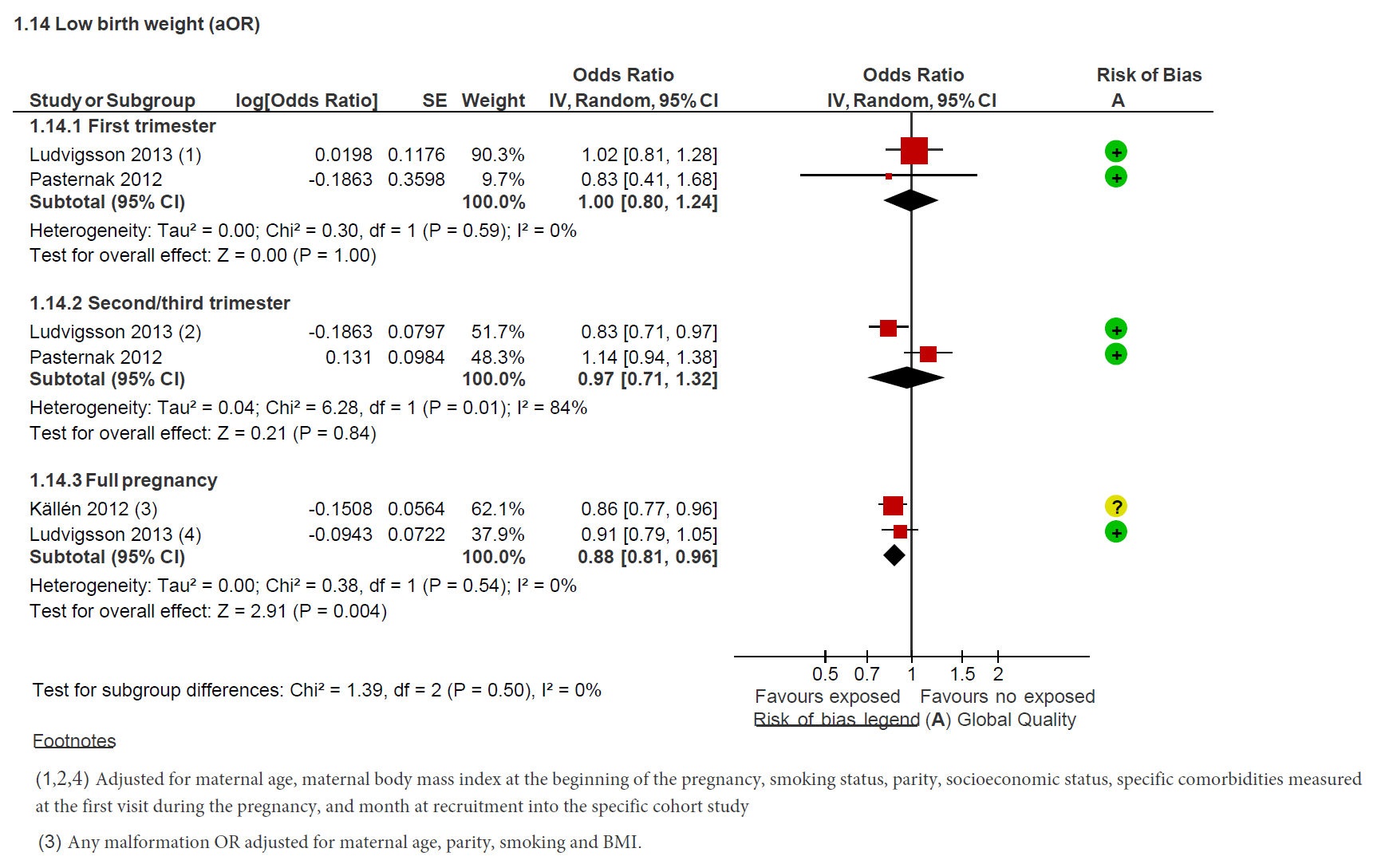
**

## **Small for gestational age <10th percentile (aRR)**

**
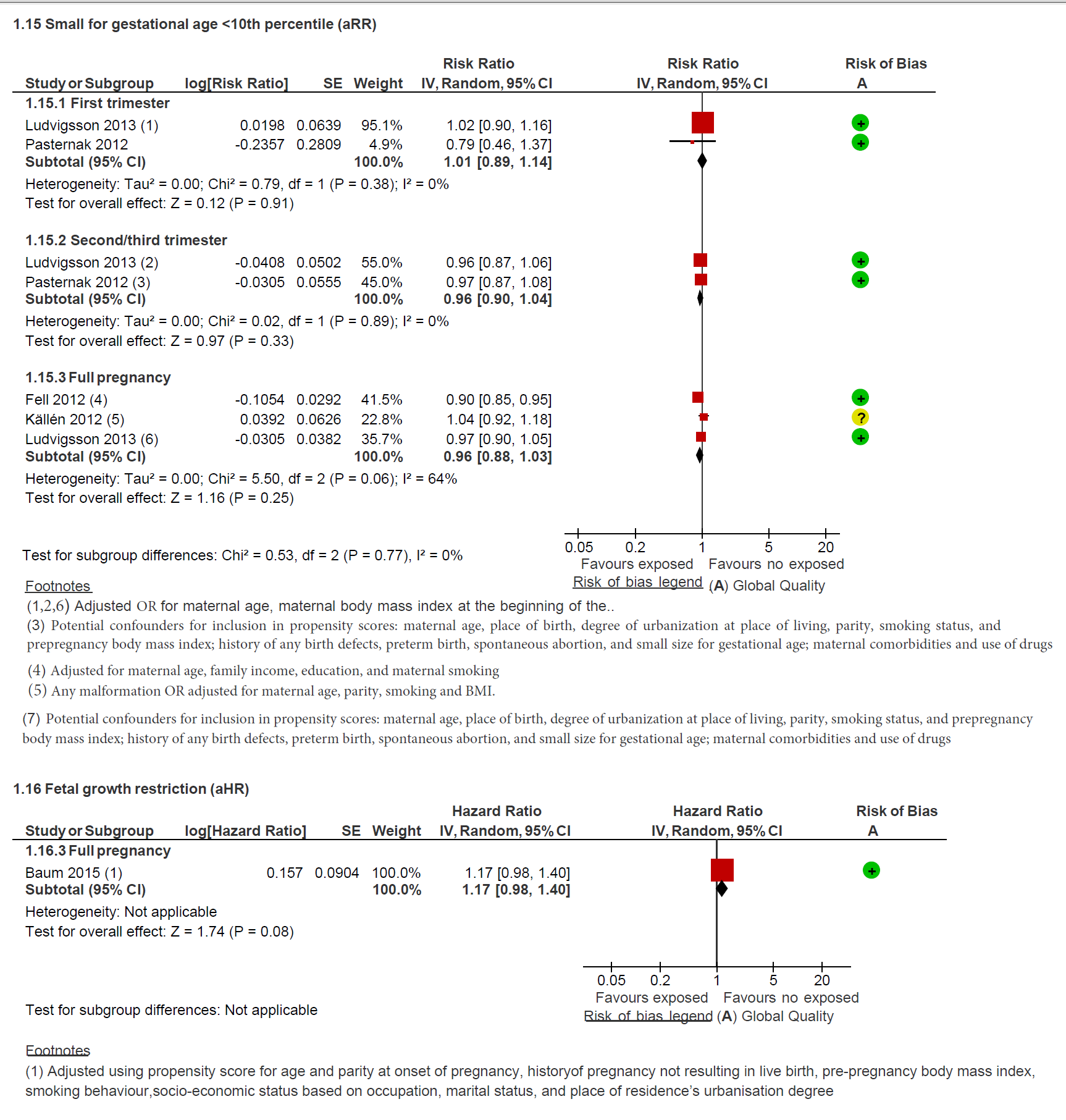
**

## **Fetal growth restriction (aHR)**

**
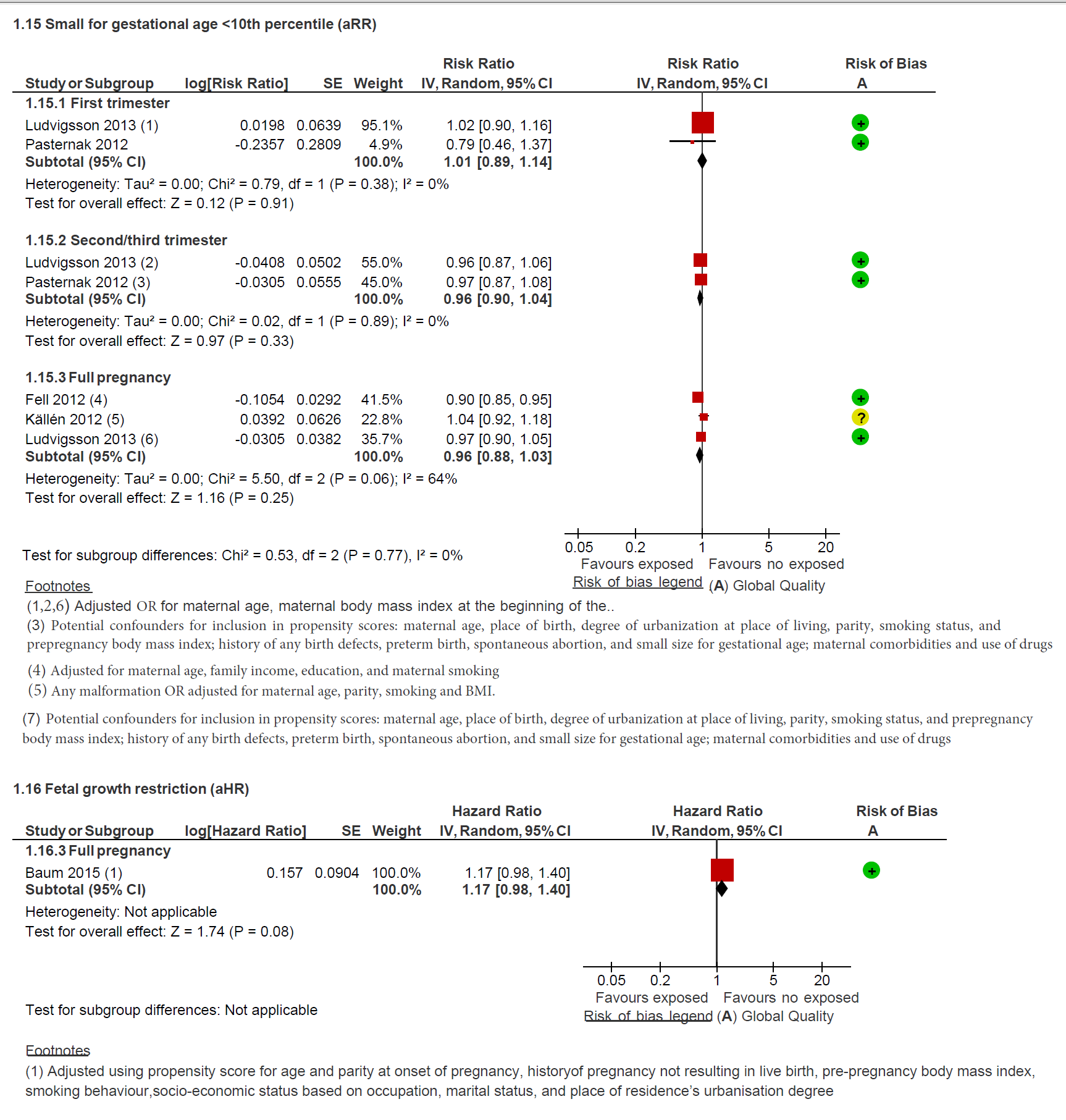
**

## **Gestational diabetes (aOR)**

**
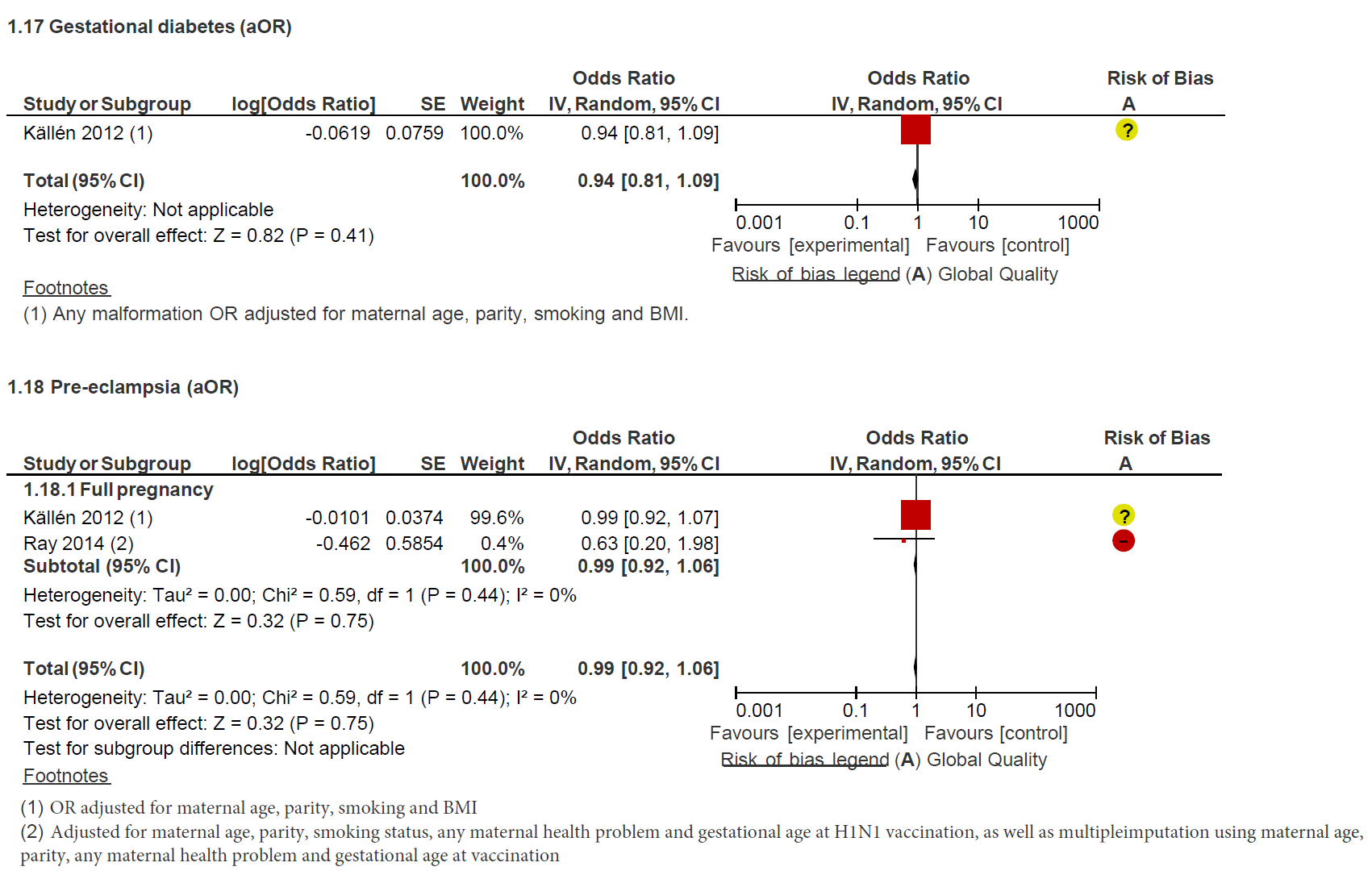
**

## **Pre-eclampsia (aOR)**

**
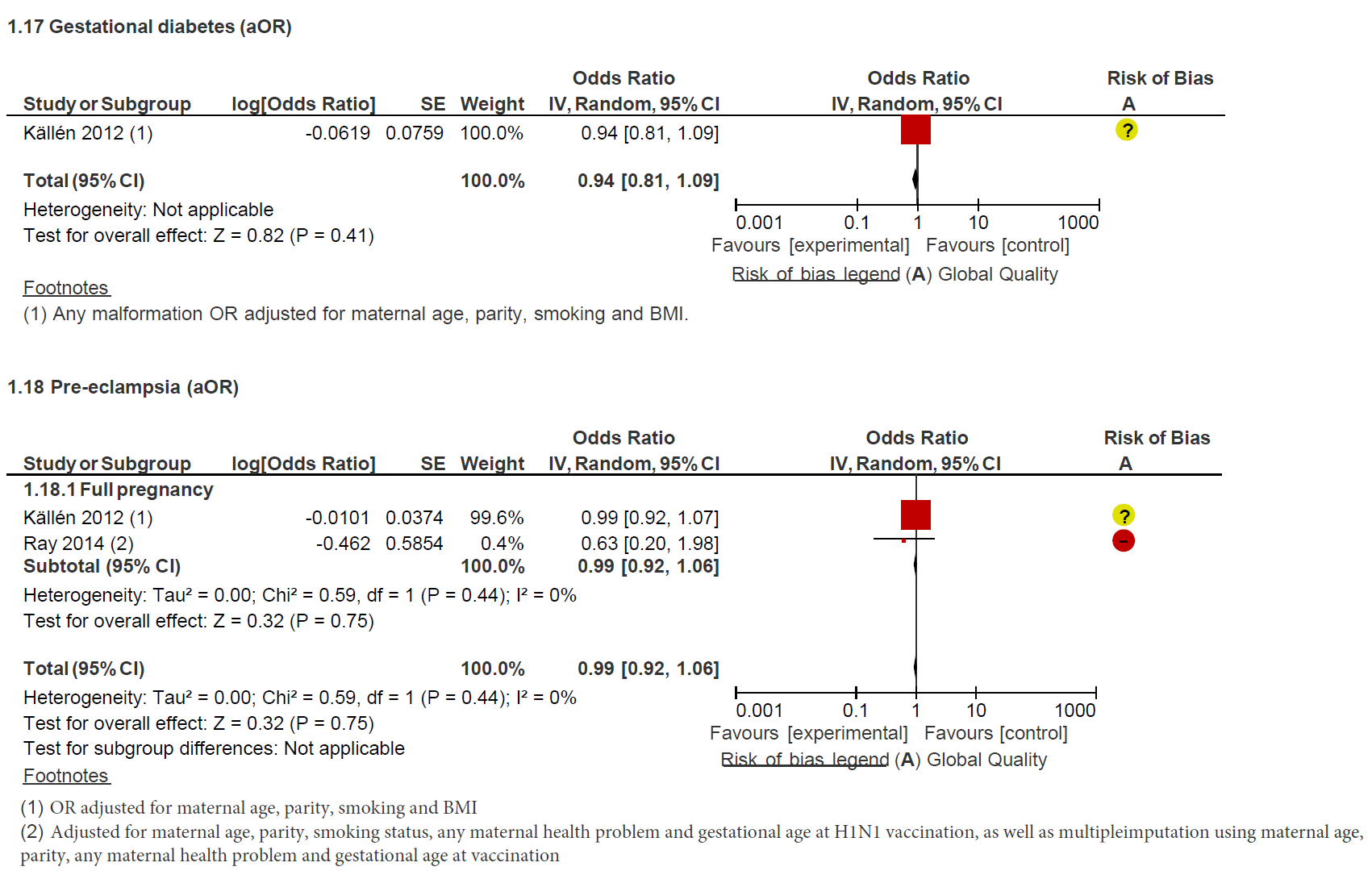
**

**Caesarean section (aOR)**

**
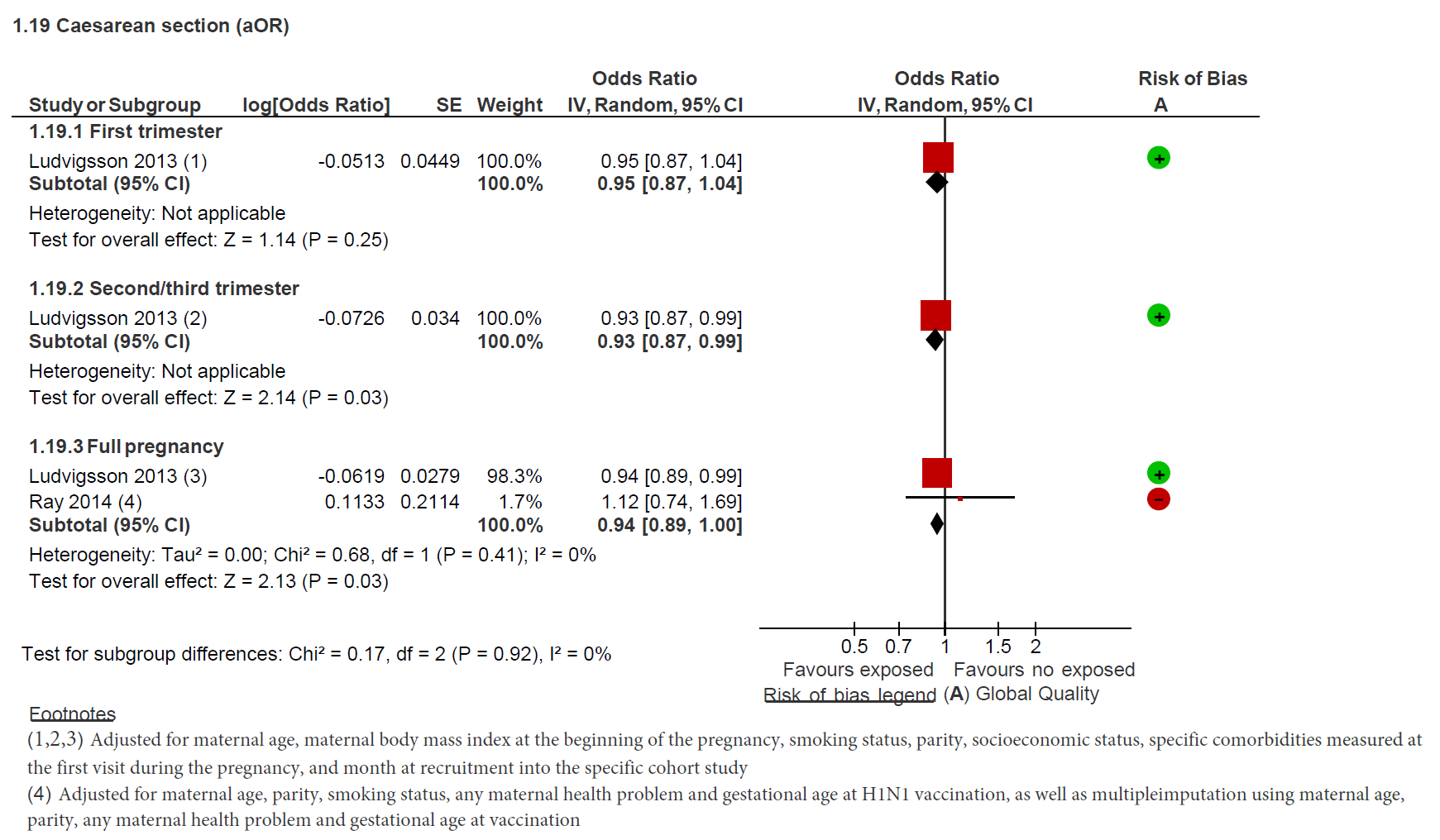
**

## ***3.2 EXPOSURE TO VACCINES CONTAINING ALUMINUM VS. NO EXPOSURE***

**Respiratory Distress Syndrome (aRR)**

**
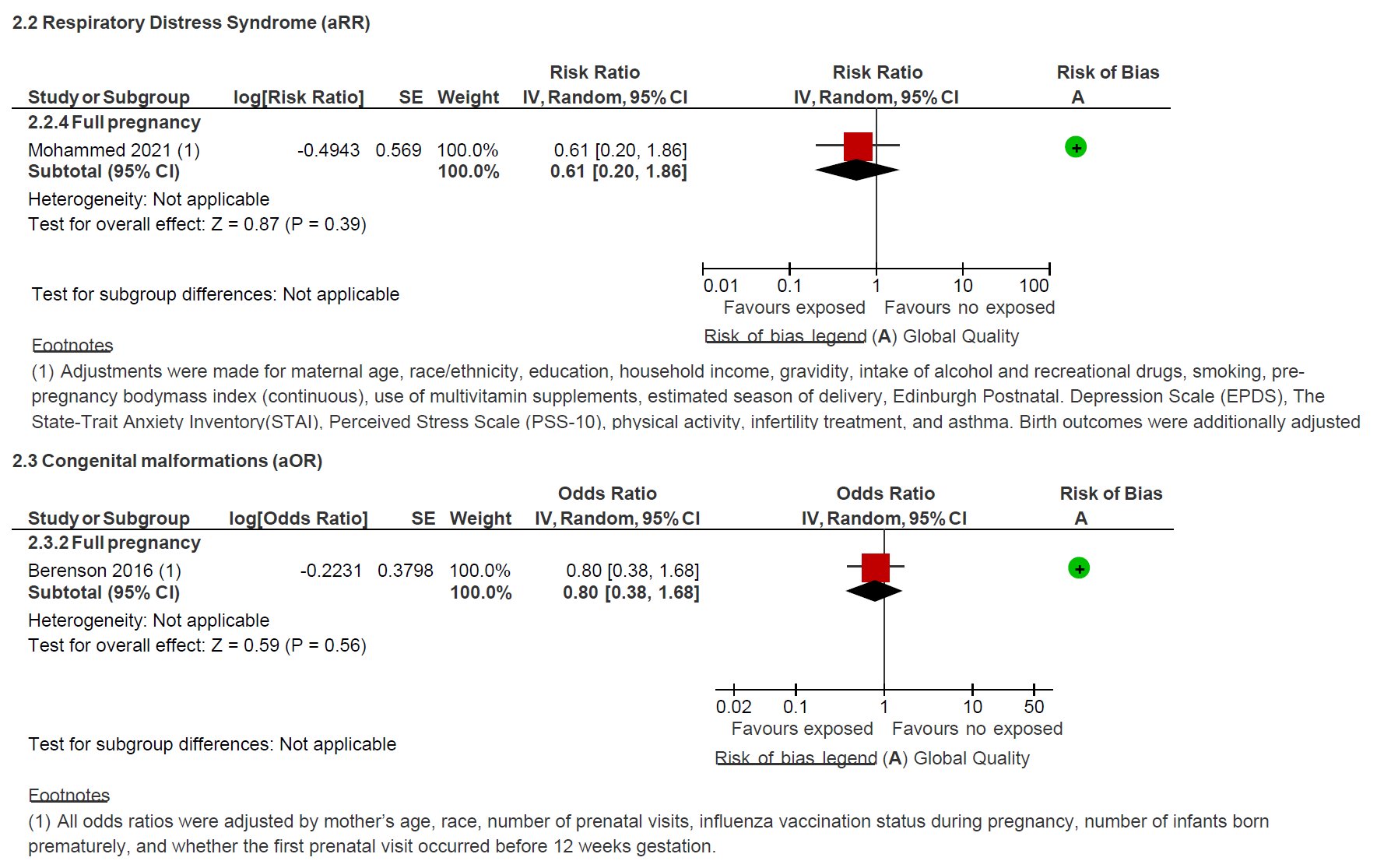
**

**Congenital malformations (aOR)**

**
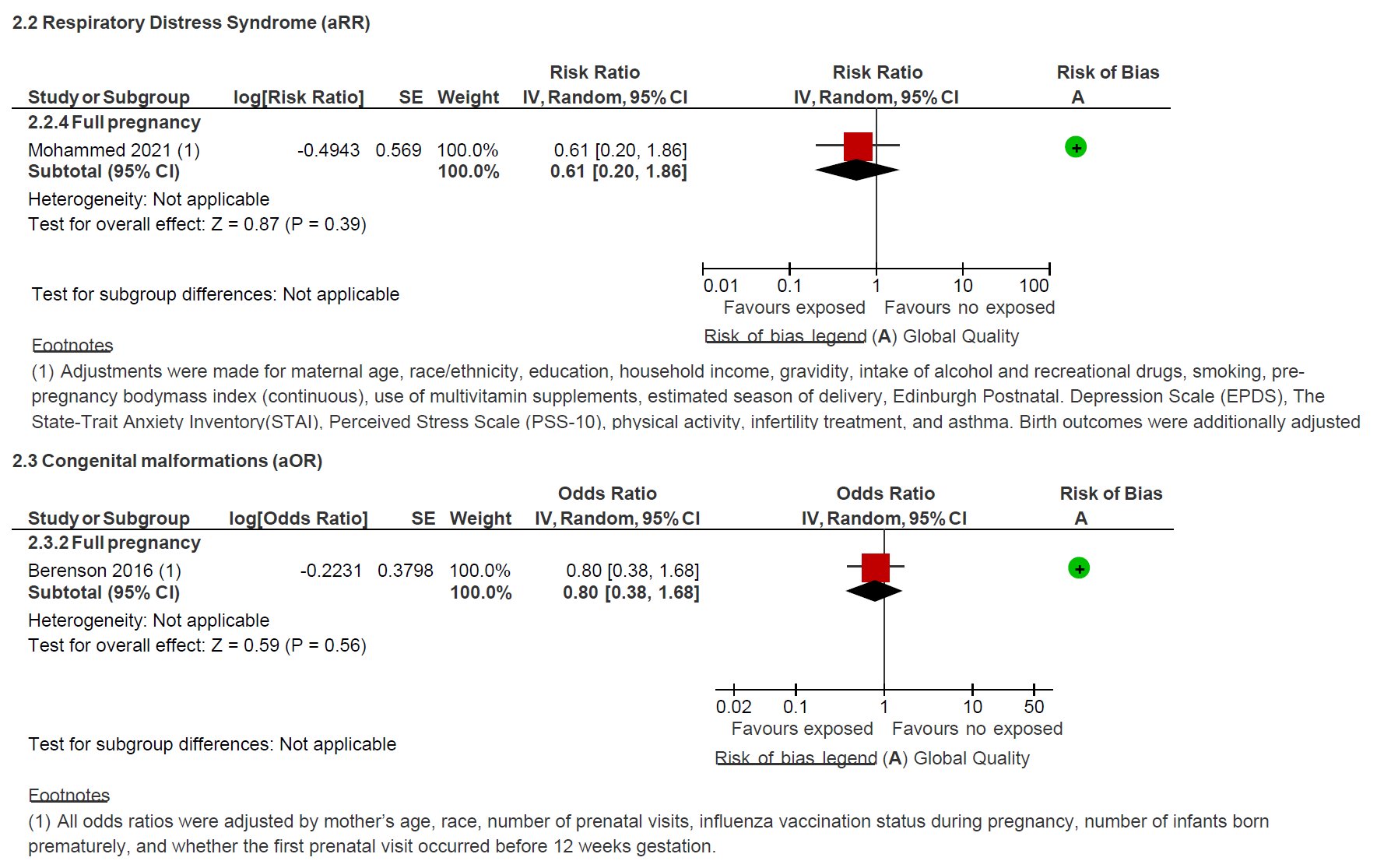
**

**Preterm birth (aHR & aOR)**

**
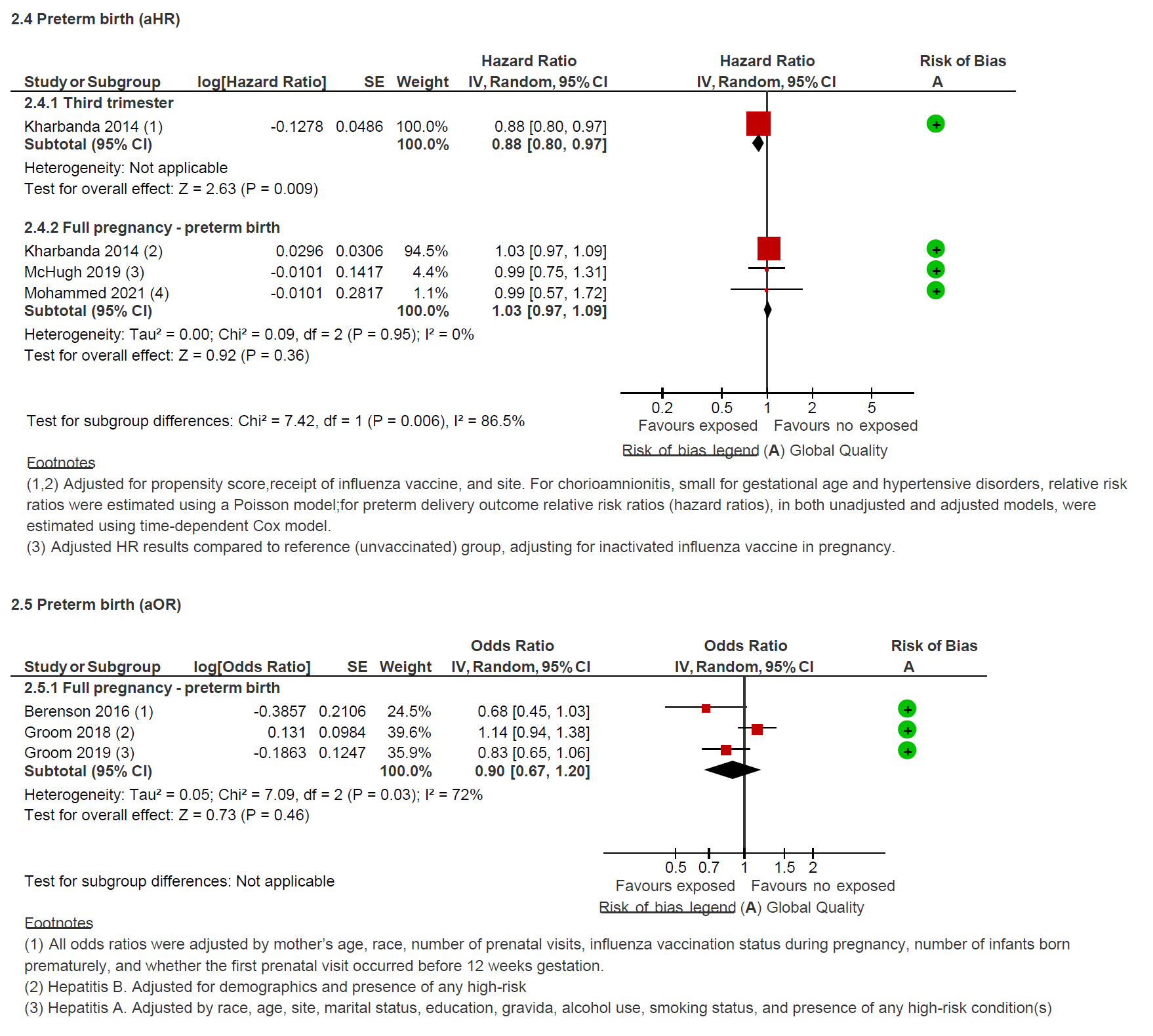
**

**Low Apgar at 5 minutes (aRR)**

**
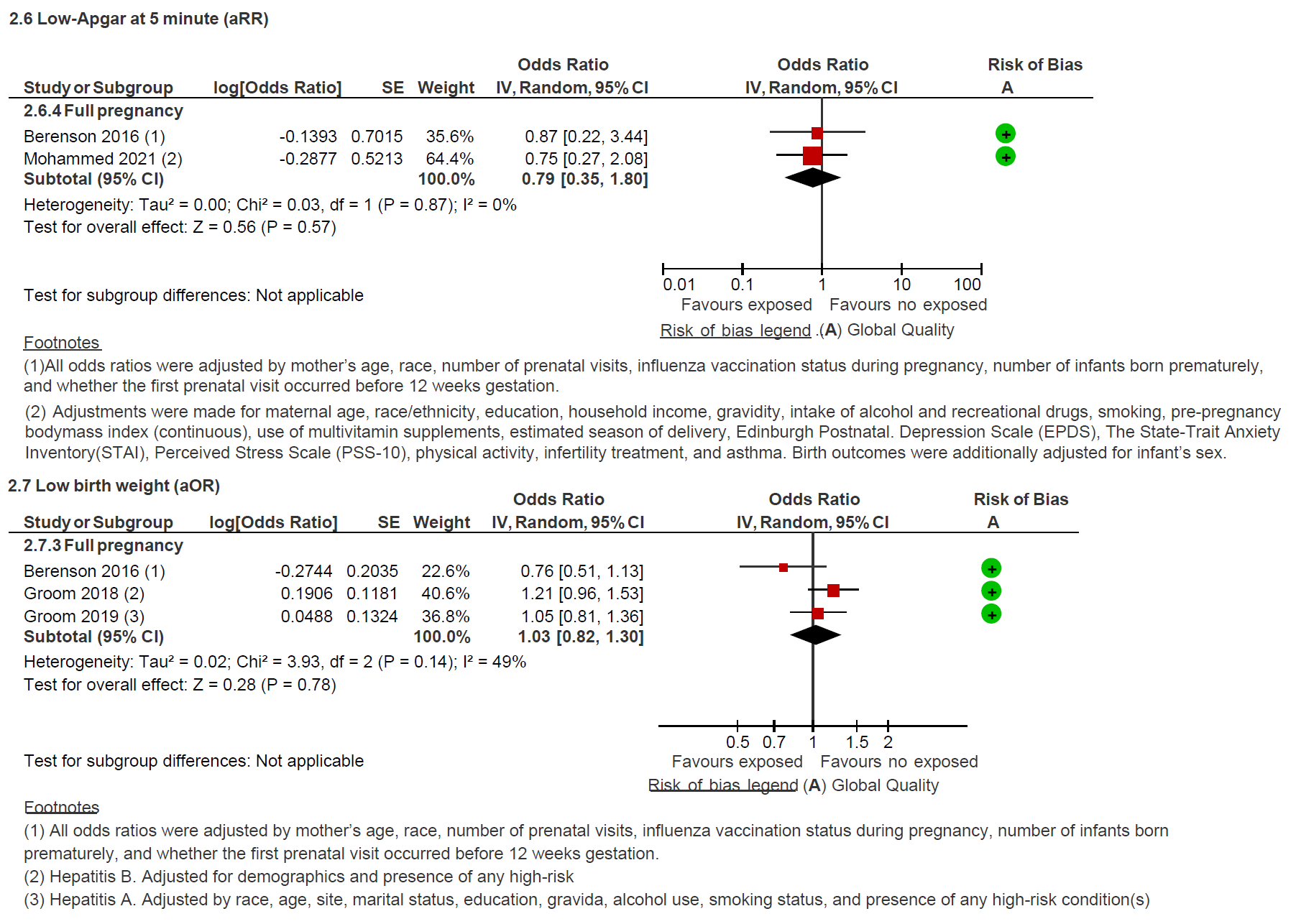
**

## **Low birth weight (aOR & aHR)**

**
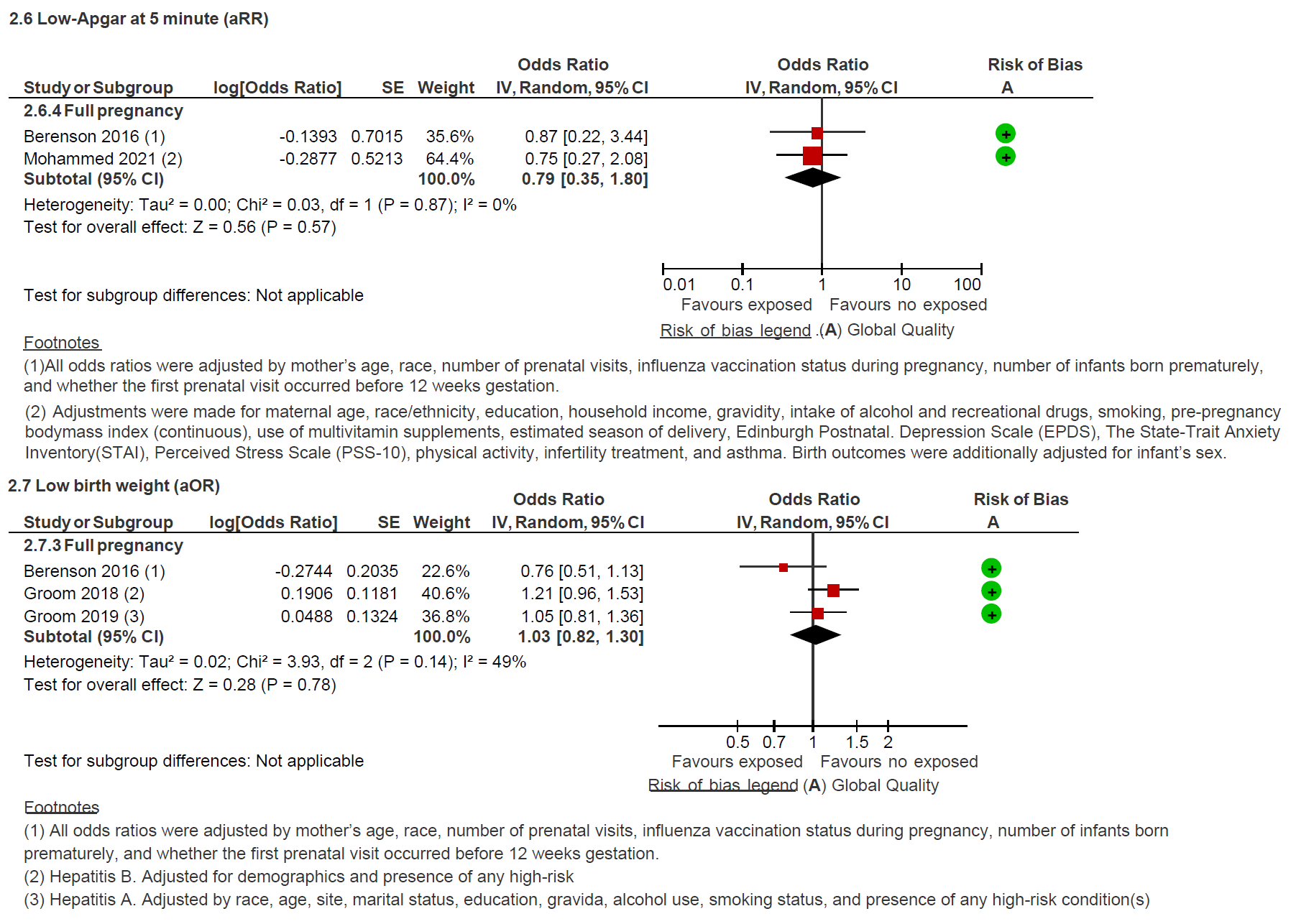
**

**
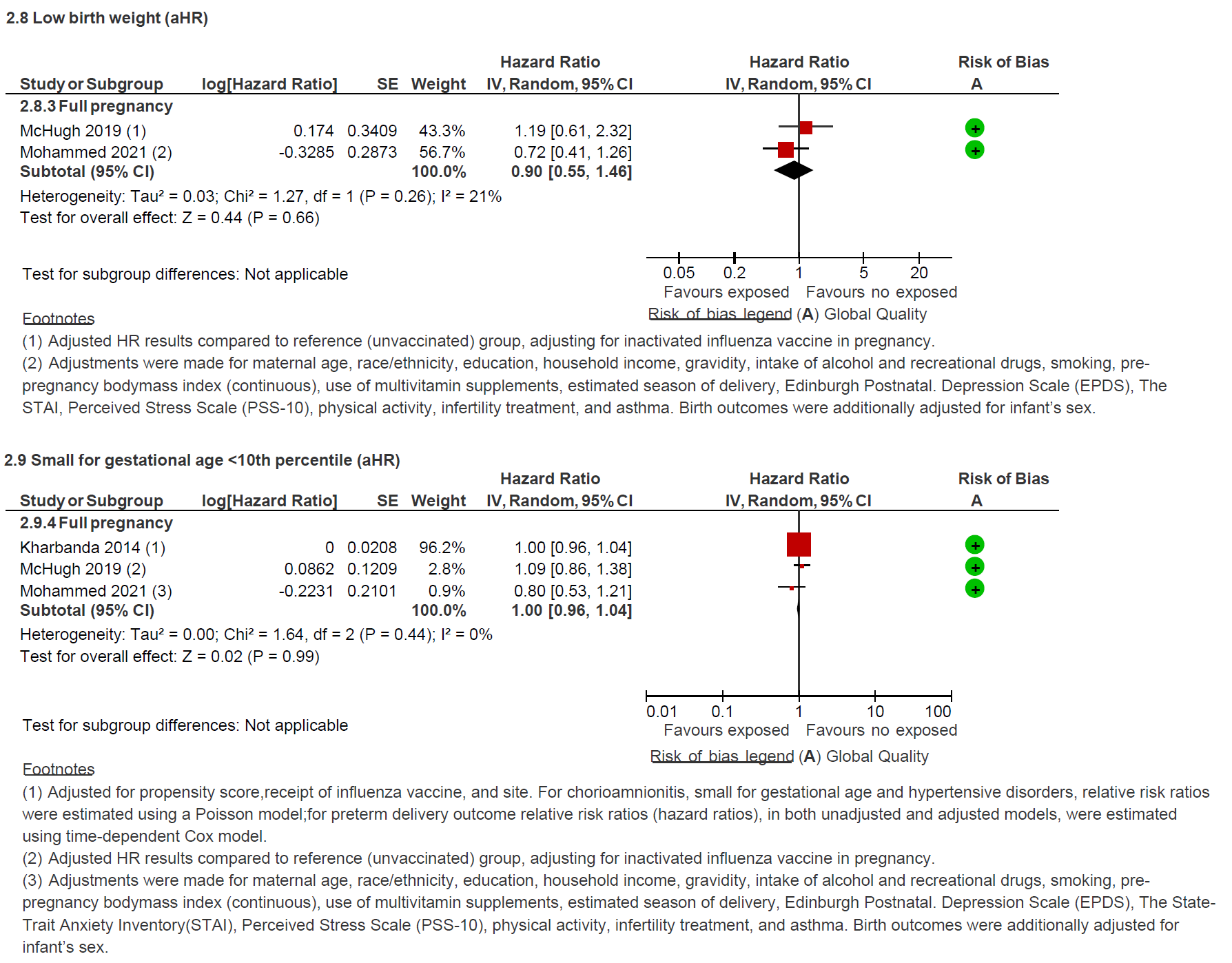
**

## **Small for gestational age <10th percentile (aHR & aOR)**

**
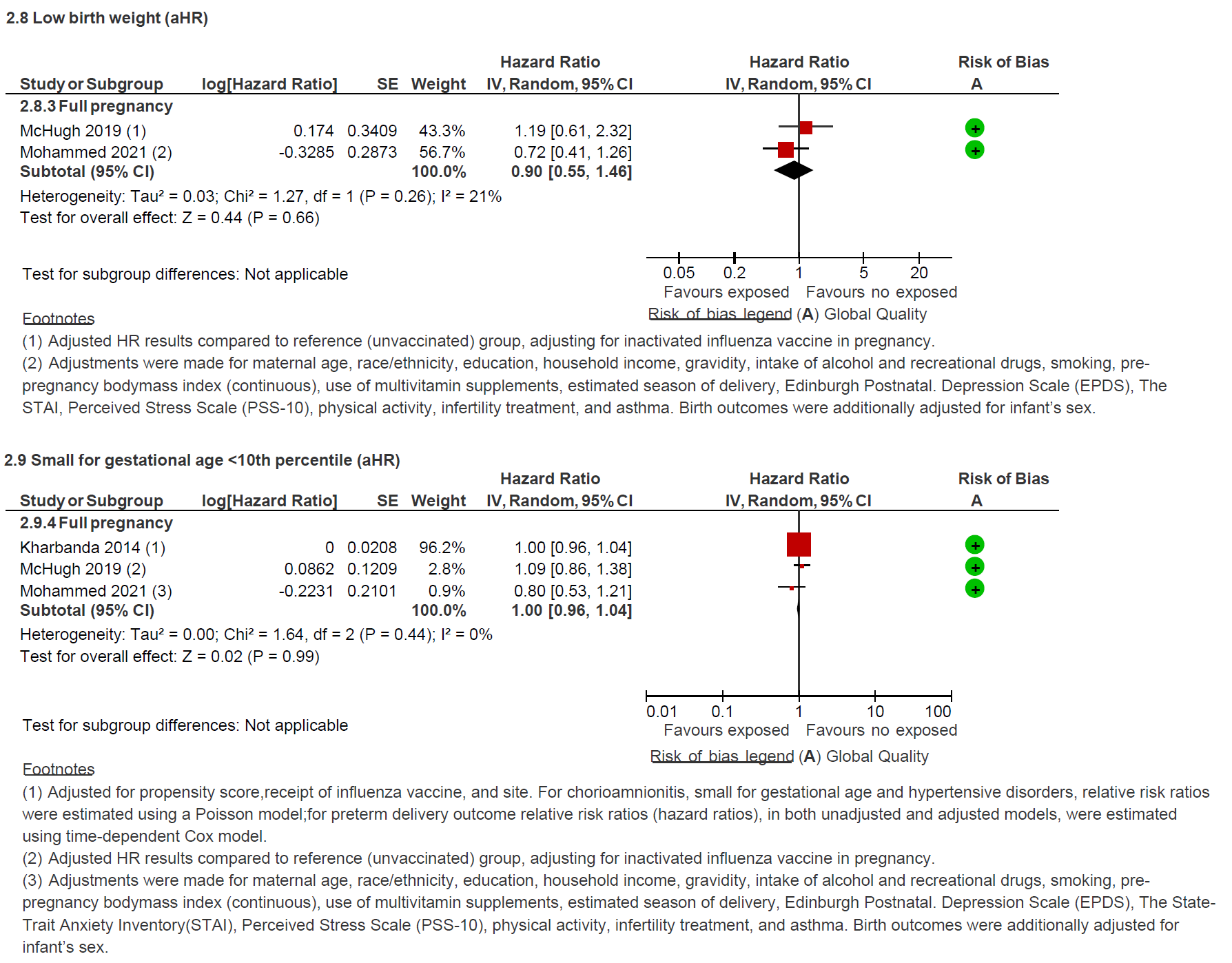
**

**
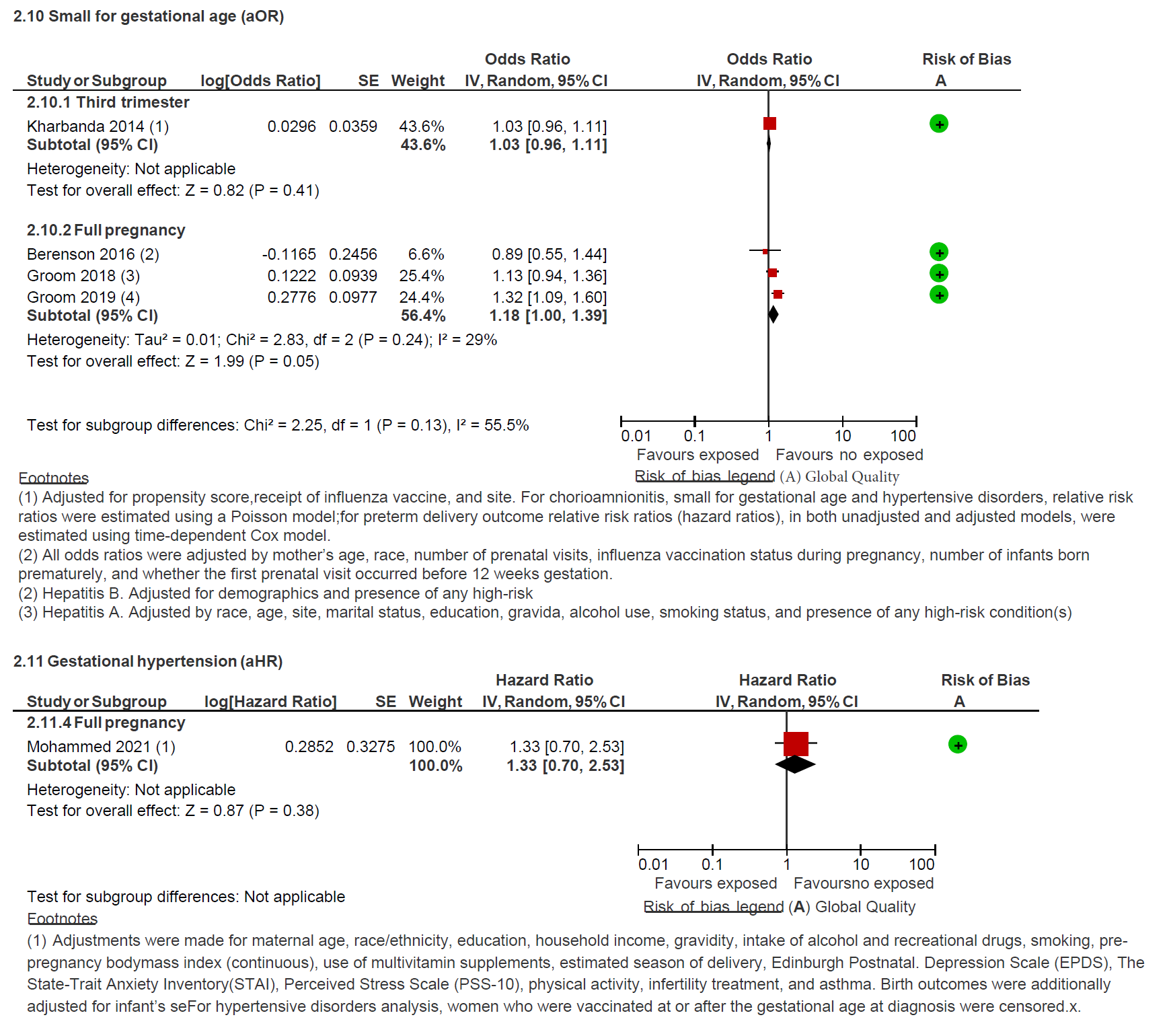
**

## **Gestational diabetes (aHR)**

**
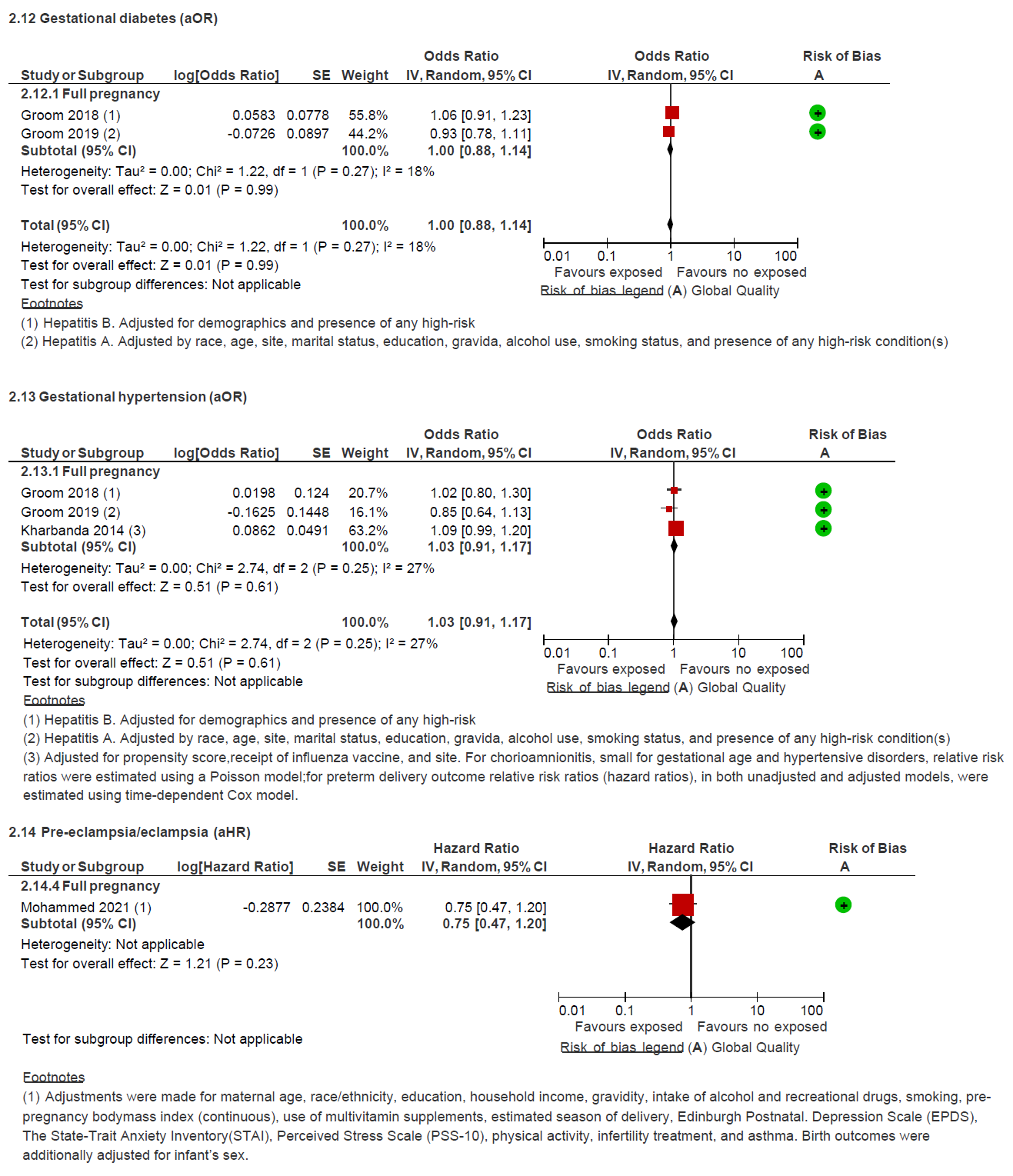
**

## **Gestational hypertension (aHR & aOR)**

**
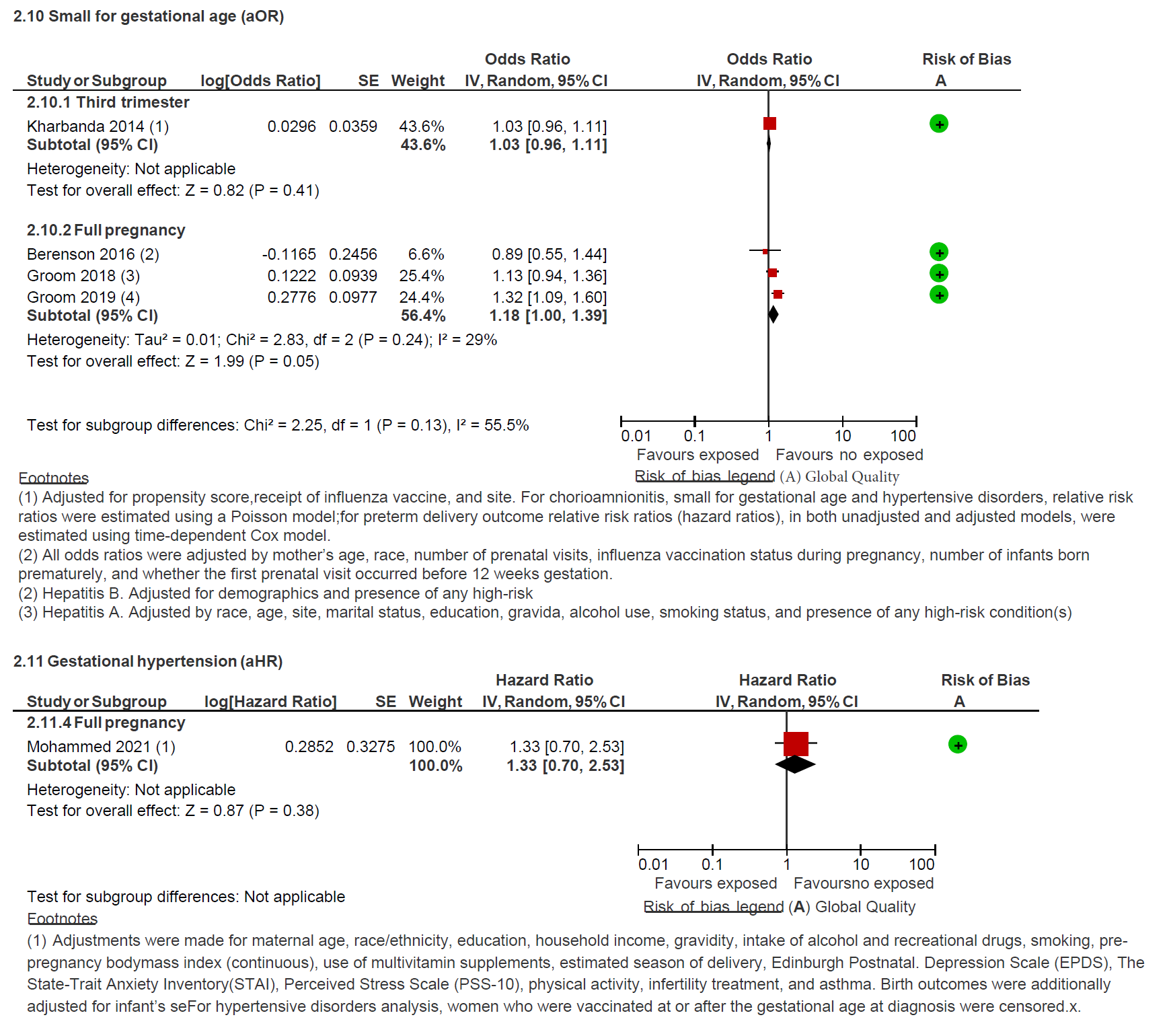
**

**
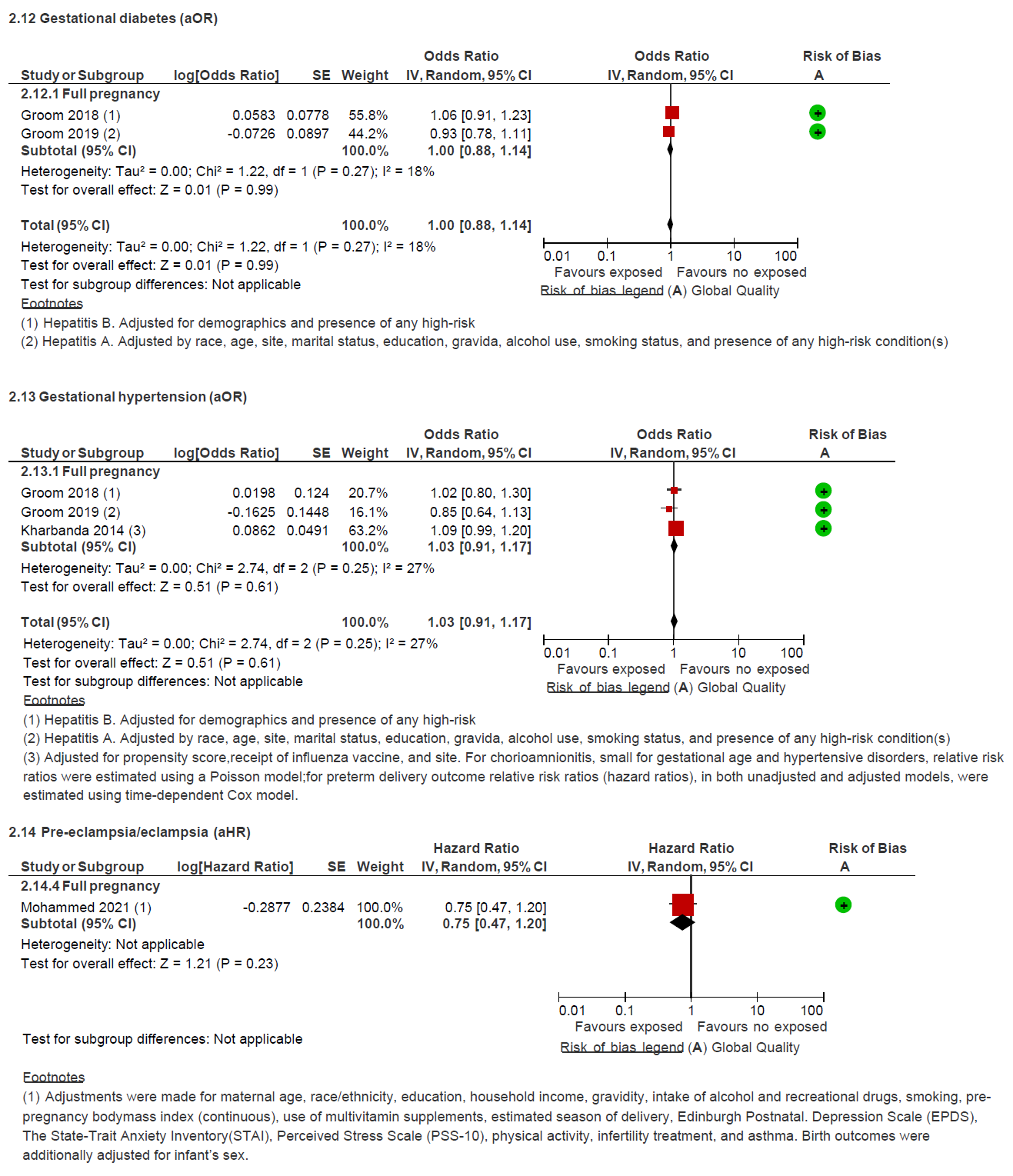
**

## **Pre-eclampsia/eclampsia (aHR & aOR)**

**
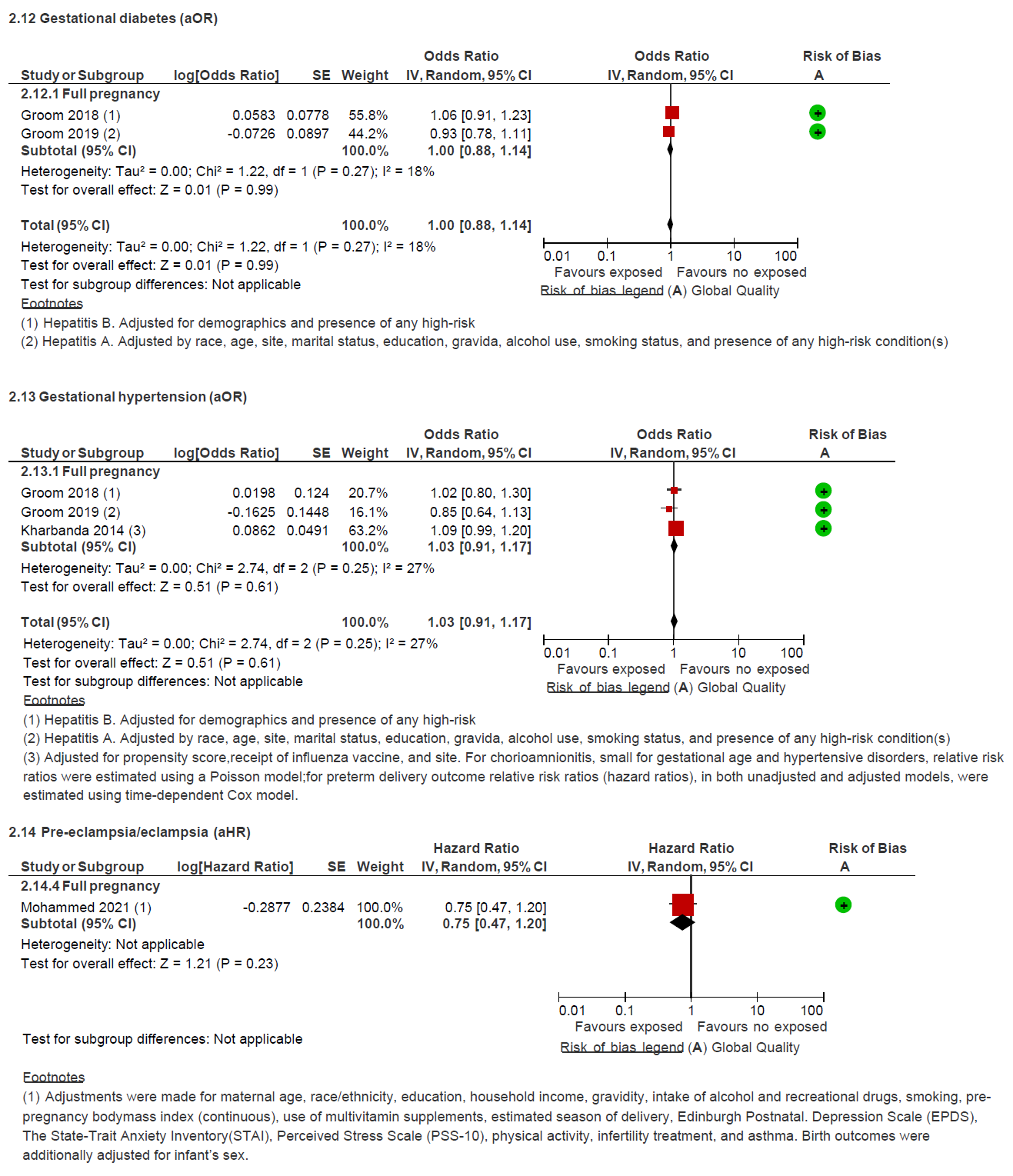

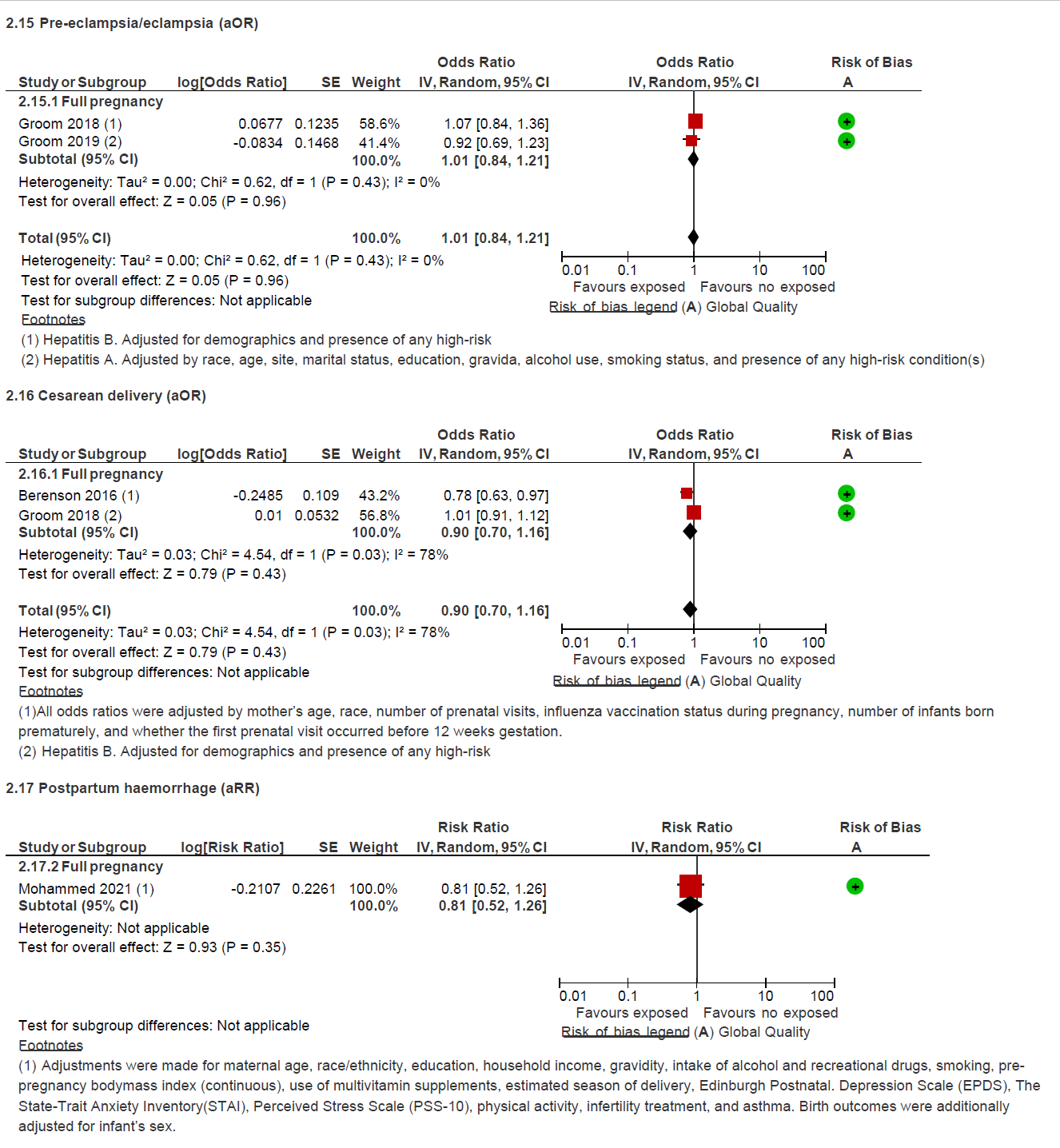
**

## **Cesarean delivery (aOR)**

**
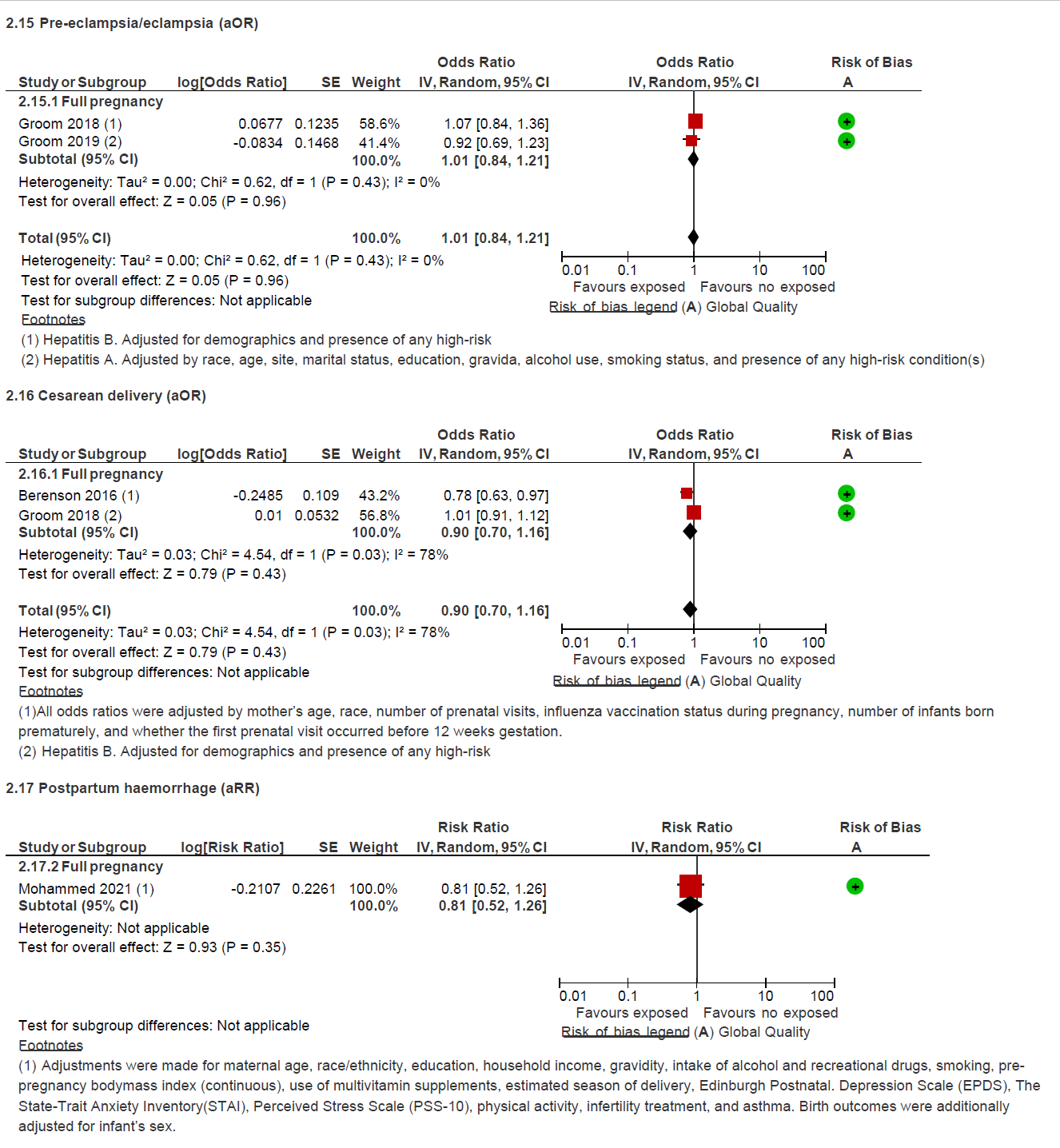
**

## **Postpartum hemorrhage (aRR)**

**
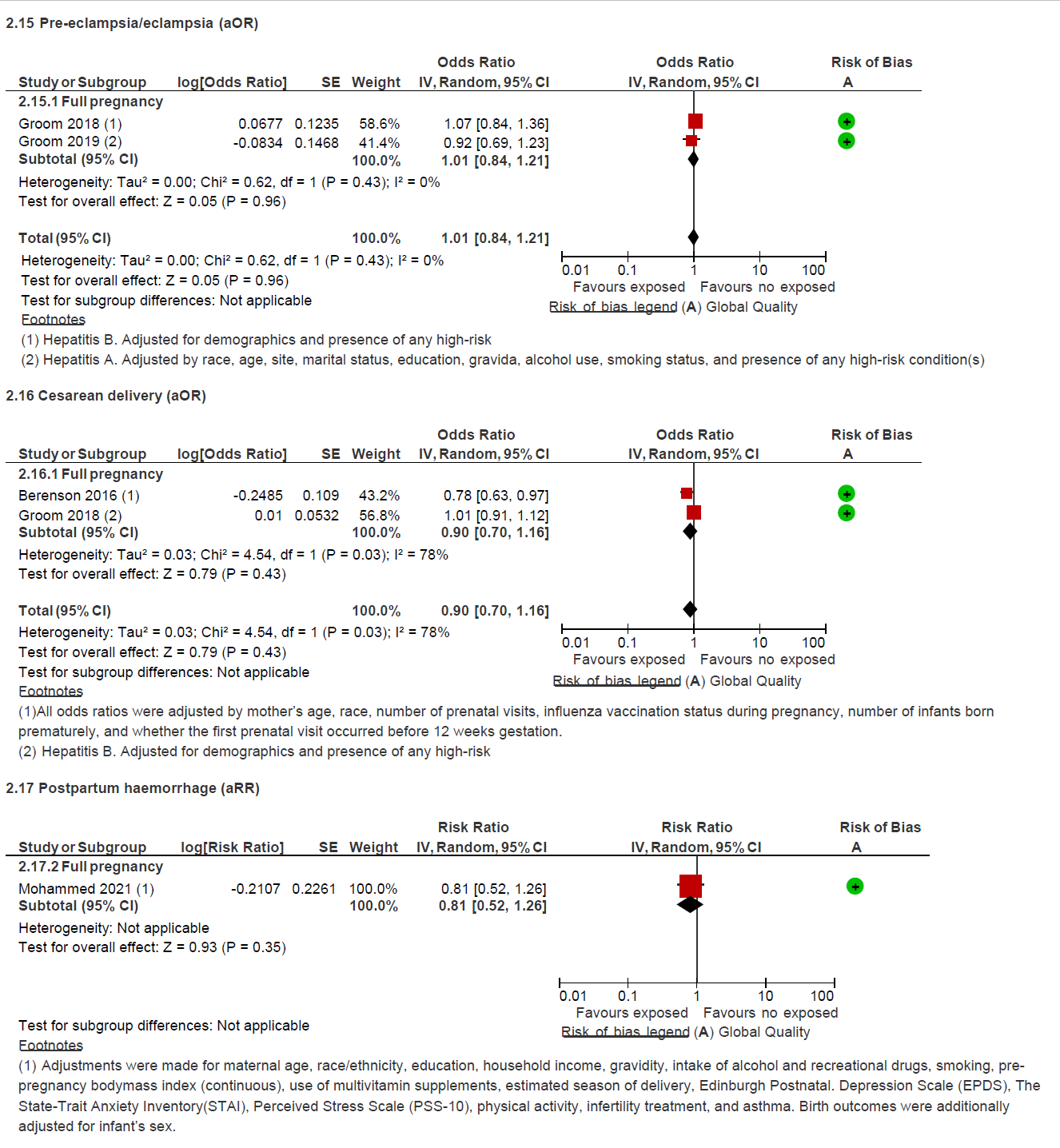
**

## **Chorioamnionitis/funisitis (aRR)**

**
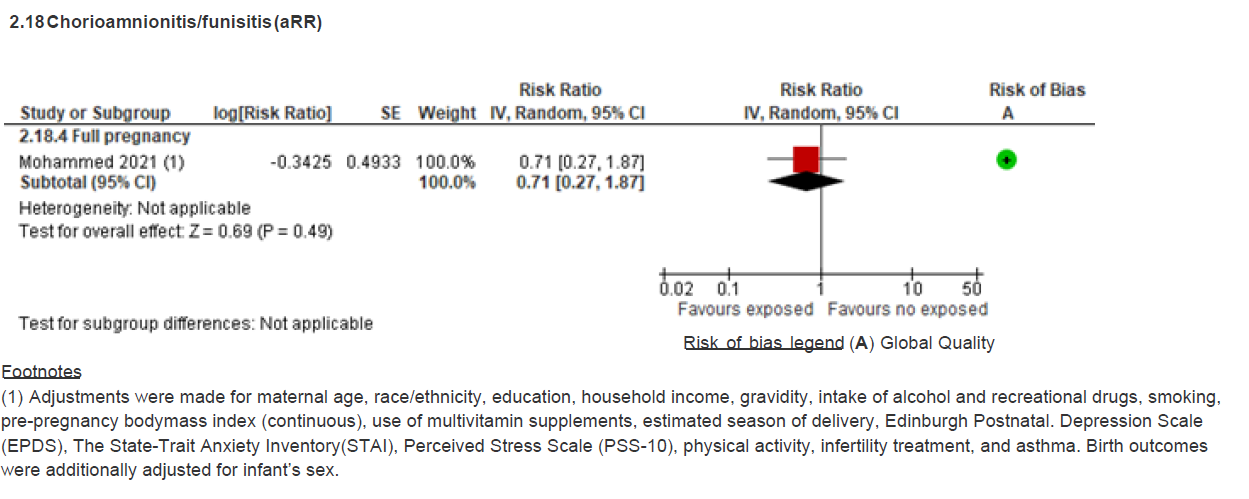
**

## **4 UN*CONTROLLED STUDIES ASSESSING EXPOSURE VS NO EXPOSURE TO NON-COVID-19 VACCINES IN PREGNANT PERSONS***

## ***3.1 VACCINES CONTAINING AS03 ADJUVANTS IN HUMANS***

**Proportion meta-analysis fetal death**

Method: Stuart-Ord (inverse double arcsine square root)

| Stratum | Responding | Total |  |
| --- | --- | --- | --- |
| 1 | 7 | 550 | Guo 2010 |

| Stratum | Proportion | 95% CI (exact) | |  |
| --- | --- | --- | --- | --- |
| 1 | 0.012727 | 0.005132 | 0.026046 | Guo 2010 |

| Stratum | Standardized effect | Variance | % Weights (fixed, random) | |  |
| --- | --- | --- | --- | --- | --- |
| 1 | 0.012727 | 0.001817 | 100 | 100 | Guo 2010 |

Fixed effects (inverse variance)

Pooled proportion = 0.013597 (95% CI = 0.00563 to 0.024956)

Non-combinability of studies

Cochran Q = 0 (df = 0) P = *

Moment-based estimate of between studies variance = 0

I_2_ (inconsistency) = *% (95% CI = *% to *%)

Random effects (DerSimonian-Laird)

Pooled proportion = 0.013597 (95% CI = 0.00563 to 0.024956)

Bias indicators

Begg-Mazumdar: Kendall's <too few strata> P = *

Egger: bias = * (95% CI = * to *) P = *

Harbord: bias = * (92.5% CI = * to *) P = *

**Proportion meta-analysis Stillbirth**

Method: Stuart-Ord (inverse double arcsine square root)

| Stratum | Responding | Total |  |
| --- | --- | --- | --- |
| 1 | 78 | 34241 | Baum 2015 |
| 2 | 1 | 5772 | Folkenberg 2011 |
| 3 | 0 | 261 | Tavares 2011 |
| 4 | 0 | 117 | Mackenzie 2012 |

| Stratum | Proportion | 95% CI (exact) | |  |
| --- | --- | --- | --- | --- |
| 1 | 0.002278 | 0.001801 | 0.002842 | Baum 2015 Finland |
| 2 | 0.000173 | 0.000004 | 0.000965 | Folkenberg 2011 Denmark |
| 3 | 0 | 0 | 0.014034 | Tavares 2011 [97.5% one-sided CI] UK , Belgium |
| 4 | 0 | 0 | 0.031037 | Mackenzie 2012 [97.5% one-sided CI] UK |

| Stratum | Standardized effect | Variance | % Weights (fixed, random) | |  |
| --- | --- | --- | --- | --- | --- |
| 1 | 0.002278 | 0.000029 | 84.770876 | 41.779977 | Baum 2015 |
| 2 | 0.000173 | 0.000173 | 14.290842 | 38.482825 | Folkenberg 2011 |
| 3 | 0 | 0.003824 | 0.647389 | 12.826032 | Tavares 2011 [97.5% one-sided CI] |
| 4 | 0 | 0.008511 | 0.290892 | 6.911166 | Mackenzie 2012 [97.5% one-sided CI] |

Fixed effects (inverse variance)

Pooled proportion = 0.001866 (95% CI = 0.001469 to 0.00231)

Non-combinability of studies

Cochran Q = 20.40926 (df = 3) P = 0.0001

Moment-based estimate of between studies variance = 0.001652

I_2_ (inconsistency) = 85.3% (95% CI = 52.9% to 92.5%)

Random effects (DerSimonian-Laird)

Pooled proportion = 0.001107 (95% CI = 0.000053 to 0.003506)

Bias indicators

Begg-Mazumdar: Kendall's 0.333333 P = 0.75

Egger: bias = -0.057063 (95% CI = -13.29404 to 13.179915) P = 0.9869

Harbord: bias = -1.530685 (92.5% CI = -6.457168 to 3.395799) P = 0.3967

**Proportion meta-analysis fetal growth retardation**

Method: Stuart-Ord (inverse double arcsine square root)

| Stratum | Responding | Total |  |
| --- | --- | --- | --- |
| 1 | 627 | 34241 | Baum 2015 |

| Stratum | Proportion | 95% CI (exact) | |  |
| --- | --- | --- | --- | --- |
| 1 | 0.018311 | 0.016918 | 0.019788 | Baum 2015 |

| Stratum | Standardized effect | Variance | % Weights (fixed, random) | |  |
| --- | --- | --- | --- | --- | --- |
| 1 | 0.018311 | 0.000029 | 100 | 100 | Baum 2015 |

Fixed effects (inverse variance)

Pooled proportion = 0.018325 (95% CI = 0.016932 to 0.019773)

Non-combinability of studies

Cochran Q = 0 (df = 0) P = *

Moment-based estimate of between studies variance = 0

I_2_ (inconsistency) = *% (95% CI = *% to *%)

Random effects (DerSimonian-Laird)

Pooled proportion = 0.018325 (95% CI = 0.016932 to 0.019773)

Bias indicators

Begg-Mazumdar: Kendall's <too few strata> P = *

Egger: bias = * (95% CI = * to *) P = *

Harbord: bias = * (92.5% CI = * to *) P = *

**Proportion meta-analysis Hypertensive disorders of pregnancy**

Method: Stuart-Ord (inverse double arcsine square root)

| Stratum | Responding | Total |  |
| --- | --- | --- | --- |
| 1 | 913 | 22915 | Fell 2012 |
| 2 | 19 | 509 | Ray 2014 |

| Stratum | Proportion | 95% CI (exact) | |  |
| --- | --- | --- | --- | --- |
| 1 | 0.039843 | 0.037347 | 0.042456 | Fell 2012 |
| 2 | 0.037328 | 0.022621 | 0.05768 | Ray 2014 |

| Stratum | Standardized effect | Variance | % Weights (fixed, random) | |  |
| --- | --- | --- | --- | --- | --- |
| 1 | 0.039843 | 0.000044 | 97.824973 | 97.824973 | Fell 2012 |
| 2 | 0.037328 | 0.001963 | 2.175027 | 2.175027 | Ray 2014 |

Fixed effects (inverse variance)

Pooled proportion = 0.039827 (95% CI = 0.037361 to 0.042369)

Non-combinability of studies

Cochran Q = 0.035458 (df = 1) P = 0.8506

Moment-based estimate of between studies variance = 0

I_2_ (inconsistency) = 0% (95% CI = *% to *%)

Random effects (DerSimonian-Laird)

Pooled proportion = 0.039827 (95% CI = 0.037361 to 0.042369)

Bias indicators

Begg-Mazumdar: Kendall's <too few strata> P = *

Egger: bias = * (95% CI = * to *) P = *

Harbord: bias = -0.340949 (92.5% CI = * to *) P = *

**Proportion meta-analysis Low birth weight**

Method: Stuart-Ord (inverse double arcsine square root)

| Stratum | Responding | Total |  |
| --- | --- | --- | --- |
| 1 | 228 | 6642 | Pasternak 2012 |
| 2 | 21 | 260 | Tavares 2011 |
| 3 | 337 | 13297 | Ludvigsson 2013 |

| Stratum | Proportion | 95% CI (exact) | |  |
| --- | --- | --- | --- | --- |
| 1 | 0.034327 | 0.030079 | 0.03899 | Pasternak 2012 |
| 2 | 0.080769 | 0.050692 | 0.120815 | Tavares 2011 |
| 3 | 0.025344 | 0.022739 | 0.028159 | Ludvigsson 2013 |

| Stratum | Standardized effect | Variance | % Weights (fixed, random) | |  |
| --- | --- | --- | --- | --- | --- |
| 1 | 0.034327 | 0.000151 | 32.882849 | 40.483446 | Pasternak 2012 |
| 2 | 0.080769 | 0.003839 | 1.289572 | 17.969773 | Tavares 2011 |
| 3 | 0.025344 | 0.000075 | 65.827579 | 41.546781 | Ludvigsson 2013 |

Fixed effects (inverse variance)

Pooled proportion = 0.028761 (95% CI = 0.026501 to 0.03111)

Non-combinability of studies

Cochran Q = 27.864411 (df = 2) P < 0.0001

Moment-based estimate of between studies variance = 0.002793

I_2_ (inconsistency) = 92.8% (95% CI = 81.1% to 96.1%)

Random effects (DerSimonian-Laird)

Pooled proportion = 0.037279 (95% CI = 0.02553 to 0.051147)

Bias indicators

Begg-Mazumdar: Kendall's <too few strata> P = *

Egger: bias = * (95% CI = * to *) P = *

Harbord: bias = 6.693462 (92.5% CI = -13.272898 to 26.659822) P = 0.2161

**Proportion meta-analysis Small for gestational age**

Method: Stuart-Ord (inverse double arcsine square root)

| Stratum | Responding | Total |  |
| --- | --- | --- | --- |
| 1 | 2403 | 23265 | Fell 2012 |
| 2 | 641 | 6642 | Pasternak 2012 |
| 3 | 1131 | 13297 | Ludvigsson 2013 |

| Stratum | Proportion | 95% CI (exact) | |  |
| --- | --- | --- | --- | --- |
| 1 | 0.103288 | 0.099406 | 0.10727 | Fell 2012 |
| 2 | 0.096507 | 0.089509 | 0.103862 | Pasternak 2012 |
| 3 | 0.085057 | 0.080369 | 0.089927 | Ludvigsson 2013 |

| Stratum | Standardized effect | Variance | % Weights (fixed, random) | |  |
| --- | --- | --- | --- | --- | --- |
| 1 | 0.103288 | 0.000043 | 53.848468 | 34.530048 | Fell 2012 |
| 2 | 0.096507 | 0.000151 | 15.3742 | 31.804205 | Pasternak 2012 |
| 3 | 0.085057 | 0.000075 | 30.777332 | 33.665747 | Ludvigsson 2013 |

Fixed effects (inverse variance)

Pooled proportion = 0.096508 (95% CI = 0.093742 to 0.09931)

Non-combinability of studies

Cochran Q = 32.983861 (df = 2) P < 0.0001

Moment-based estimate of between studies variance = 0.001212

I_2_ (inconsistency) = 93.9% (95% CI = 85.5% to 96.5%)

Random effects (DerSimonian-Laird)

Pooled proportion = 0.094892 (95% CI = 0.083275 to 0.107183)

Bias indicators

Begg-Mazumdar: Kendall's <too few strata> P = *

Egger: bias = * (95% CI = * to *) P = *

Harbord: bias = -6.386396 (92.5% CI = -107.496273 to 94.723481) P = 0.6879

**Proportion meta-analysis Preterm birth**

Method: Stuart-Ord (inverse double arcsine square root)

| Stratum | Responding | Total |  |
| --- | --- | --- | --- |
| 1 | 31 | 359 | Guo 2010 |
| 2 | 1324 | 34241 | Baum 2015 |
| 3 | 1376 | 23280 | Fell 2012 |
| 4 | 302 | 6642 | Pasternak 2012 |
| 5 | 40 | 509 | Ray 2014 |
| 6 | 12 | 261 | Tavares 2011 |
| 7 | 635 | 13297 | Ludvigsson 2013 |

| Stratum | Proportion | 95% CI (exact) | |  |
| --- | --- | --- | --- | --- |
| 1 | 0.086351 | 0.059425 | 0.120329 | Guo 2010 |
| 2 | 0.038667 | 0.03665 | 0.040763 | Baum 2015 |
| 3 | 0.059107 | 0.056111 | 0.062213 | Fell 2012 |
| 4 | 0.045468 | 0.040583 | 0.050757 | Pasternak 2012 |
| 5 | 0.078585 | 0.056734 | 0.105475 | Ray 2014 |
| 6 | 0.045977 | 0.023979 | 0.078935 | Tavares 2011 |
| 7 | 0.047755 | 0.044192 | 0.051518 | Ludvigsson 2013 |

| Stratum | Standardized effect | Variance | % Weights (fixed, random) | |  |
| --- | --- | --- | --- | --- | --- |
| 1 | 0.086351 | 0.002782 | 0.457423 | 9.017689 | Guo 2010 |
| 2 | 0.038667 | 0.000029 | 43.568407 | 18.506765 | Baum 2015 |
| 3 | 0.059107 | 0.000043 | 29.621783 | 18.409988 | Fell 2012 |
| 4 | 0.045468 | 0.000151 | 8.451824 | 17.686305 | Pasternak 2012 |
| 5 | 0.078585 | 0.001963 | 0.648281 | 10.641021 | Ray 2014 |
| 6 | 0.045977 | 0.003824 | 0.332729 | 7.551288 | Tavares 2011 |
| 7 | 0.047755 | 0.000075 | 16.919553 | 18.186943 | Ludvigsson 2013 |

Fixed effects (inverse variance)

Pooled proportion = 0.046957 (95% CI = 0.045489 to 0.048447)

Non-combinability of studies

Cochran Q = 145.569657 (df = 6) P < 0.0001

Moment-based estimate of between studies variance = 0.002587

I_2_ (inconsistency) = 95.9% (95% CI = 94.1% to 96.9%)

Random effects (DerSimonian-Laird)

Pooled proportion = 0.053797 (95% CI = 0.044485 to 0.063938)

Bias indicators

Begg-Mazumdar: Kendall's 0.142857 P = 0.7726

Egger: bias = 2.897313 (95% CI = -4.350205 to 10.144831) P = 0.3512

Harbord: bias = 2.812817 (92.5% CI = -4.455078 to 10.080712) P = 0.4252

**Proportion meta-analysis any congenital malformation**

Method: Stuart-Ord (inverse double arcsine square root)

| Stratum | Responding | Total |  |
| --- | --- | --- | --- |
| 1 | 1 | 56 | Chavant 2013 |
| 2 | 6 | 92 | Layton 2011 |
| 3 | 18 | 330 | Pasternak 2012 |
| 4 | 6 | 261 | Tavares 2011 |
| 5 | 6 | 59 | Mackenzie 2012 |

| Stratum | Proportion | 95% CI (exact) | |  |
| --- | --- | --- | --- | --- |
| 1 | 0.017857 | 0.000452 | 0.095526 | Chavant 2013 |
| 2 | 0.065217 | 0.024308 | 0.136562 | Layton 2011 |
| 3 | 0.054545 | 0.032644 | 0.084837 | Pasternak 2012 |
| 4 | 0.022989 | 0.008482 | 0.049362 | Tavares 2011 |
| 5 | 0.101695 | 0.038244 | 0.20832 | Mackenzie 2012 |

| Stratum | Standardized effect | Variance | % Weights (fixed, random) | |  |
| --- | --- | --- | --- | --- | --- |
| 1 | 0.017857 | 0.017699 | 7.058089 | 13.133938 | Chavant 2013 |
| 2 | 0.065217 | 0.010811 | 11.555278 | 17.620481 | Layton 2011 |
| 3 | 0.054545 | 0.003026 | 41.286696 | 28.701214 | Pasternak 2012 |
| 4 | 0.022989 | 0.003824 | 32.667083 | 26.962406 | Tavares 2011 |
| 5 | 0.101695 | 0.016807 | 7.432854 | 13.581961 | Mackenzie 2012 |

Fixed effects (inverse variance)

Pooled proportion = 0.046484 (95% CI = 0.032999 to 0.062144)

Non-combinability of studies

Cochran Q = 9.233824 (df = 4) P = 0.0555

Moment-based estimate of between studies variance = 0.009354

I_2_ (inconsistency) = 56.7% (95% CI = 0% to 81.9%)

Random effects (DerSimonian-Laird)

Pooled proportion = 0.049708 (95% CI = 0.027443 to 0.078111)

Bias indicators

Begg-Mazumdar: Kendall's 0.4 P = 0.4833

Egger: bias = 1.555352 (95% CI = -2.504222 to 5.614926) P = 0.3099

Harbord: bias = 1.238953 (92.5% CI = -4.494095 to 6.972002) P = 0.603

**Proportion meta-analysis Neonatal death**

Method: Stuart-Ord (inverse double arcsine square root)

| Stratum | Responding | Total |  |
| --- | --- | --- | --- |
| 1 | 23 | 34241 | Baum 2015 |

| Stratum | Proportion | 95% CI (exact) | |  |
| --- | --- | --- | --- | --- |
| 1 | 0.000672 | 0.000426 | 0.001008 | Baum 2015 |

| Stratum | Standardized effect | Variance | % Weights (fixed, random) | |  |
| --- | --- | --- | --- | --- | --- |
| 1 | 0.000672 | 0.000029 | 100 | 100 | Baum 2015 |

Fixed effects (inverse variance)

Pooled proportion = 0.000686 (95% CI = 0.000437 to 0.000992)

Non-combinability of studies

Cochran Q = 0 (df = 0) P = *

Moment-based estimate of between studies variance = 0

I_2_ (inconsistency) = *% (95% CI = *% to *%)

Random effects (DerSimonian-Laird)

Pooled proportion = 0.000686 (95% CI = 0.000437 to 0.000992)

Bias indicators

Begg-Mazumdar: Kendall's <too few strata> P = *

Egger: bias = * (95% CI = * to *) P = *

Harbord: bias = * (92.5% CI = * to *) P = *

**Proportion meta-analysis injection site reactions LMICs**

Method: Stuart-Ord (inverse double arcsine square root)

| Stratum | Responding | Total |  |
| --- | --- | --- | --- |
| 1 | 64 | 90 | Oppermann 2012 (Germany) |
| 2 | 37 | 56 | Chavant 2013 (France) |
| 3 | 55 | 80317 | GalindoSantana 2011 (Cuba) |

| Stratum | Proportion | 95% CI (exact) | |  |
| --- | --- | --- | --- | --- |
| 1 | 0.711111 | 0.606012 | 0.801841 | Oppermann 2012 |
| 2 | 0.660714 | 0.521886 | 0.781891 | Chavant 2013 |
| 3 | 0.000685 | 0.000516 | 0.000891 | GalindoSantana 2011 |

| Stratum | Standardized effect | Variance | % Weights (fixed, random) | |  |
| --- | --- | --- | --- | --- | --- |
| 1 | 0.711111 | 0.01105 | 0.112472 | 33.306056 | Oppermann 2012 |
| 2 | 0.660714 | 0.017699 | 0.070217 | 33.185022 | Chavant 2013 |
| 3 | 0.000685 | 0.000012 | 99.817311 | 33.508922 | GalindoSantana 2011 |

Fixed effects (inverse variance)

Pooled proportion = 0.000786 (95% CI = 0.000604 to 0.000991)

Non-combinability of studies

Cochran Q = 534.004068 (df = 2) P < 0.0001

Moment-based estimate of between studies variance = 1.812065

I_2_ (inconsistency) = 99.6% (95% CI = 99.6% to 99.7%)

Random effects (DerSimonian-Laird)

Pooled proportion = 0.372131 (95% CI = 0.011521 to 0.977352)

Bias indicators

Begg-Mazumdar: Kendall's <too few strata> P = *

Egger: bias = * (95% CI = * to *) P = *

Harbord: bias = 214.727811 (92.5% CI = -71.997976 to 501.453598) P = 0.0998

**Proportion meta-analysis injection site reactions HIC**

Method: Stuart-Ord (inverse double arcsine square root)

| Stratum | Responding | Total |  |
| --- | --- | --- | --- |
| 1 | 64 | 90 | Oppermann 2012 |
| 2 | 37 | 56 | Chavant 2013 |

| Stratum | Proportion | 95% CI (exact) | |  |
| --- | --- | --- | --- | --- |
| 1 | 0.711111 | 0.606012 | 0.801841 | Oppermann 2012 |
| 2 | 0.660714 | 0.521886 | 0.781891 | Chavant 2013 |

| Stratum | Standardized effect | Variance | % Weights (fixed, random) | |  |
| --- | --- | --- | --- | --- | --- |
| 1 | 0.711111 | 0.01105 | 61.564626 | 61.564626 | Oppermann 2012 |
| 2 | 0.660714 | 0.017699 | 38.435374 | 38.435374 | Chavant 2013 |

Fixed effects (inverse variance)

Pooled proportion = 0.689512 (95% CI = 0.61257 to 0.761512)

Non-combinability of studies

Cochran Q = 0.416849 (df = 1) P = 0.5185

Moment-based estimate of between studies variance = 0

I_2_ (inconsistency) = 0% (95% CI = *% to *%)

Random effects (DerSimonian-Laird)

Pooled proportion = 0.689512 (95% CI = 0.61257 to 0.761512)

Bias indicators

Begg-Mazumdar: Kendall's <too few strata> P = *

Egger: bias = * (95% CI = * to *) P = *

Harbord: bias = -3.859506 (92.5% CI = * to *) P = *

**Proportion meta-analysis Systemic reactions**

Method: Stuart-Ord (inverse double arcsine square root)

| Stratum | Responding | Total |  |
| --- | --- | --- | --- |
| 1 | 23 | 90 | Oppermann 2012 |

| Stratum | Proportion | 95% CI (exact) | |  |
| --- | --- | --- | --- | --- |
| 1 | 0.255556 | 0.169406 | 0.358386 | Oppermann 2012 |

| Stratum | Standardized effect | Variance | % Weights (fixed, random) | |  |
| --- | --- | --- | --- | --- | --- |
| 1 | 0.255556 | 0.01105 | 100 | 100 | Oppermann 2012 |

Fixed effects (inverse variance)

Pooled proportion = 0.258223 (95% CI = 0.173803 to 0.352869)

Non-combinability of studies

Cochran Q = 0 (df = 0) P = *

Moment-based estimate of between studies variance = 0

I_2_ (inconsistency) = *% (95% CI = *% to *%)

Random effects (DerSimonian-Laird)

Pooled proportion = 0.258223 (95% CI = 0.173803 to 0.352869)

Bias indicators

Begg-Mazumdar: Kendall's <too few strata> P = *

Egger: bias = * (95% CI = * to *) P = *

Harbord: bias = * (92.5% CI = * to *) P = *

**Proportion meta-analysis Anaphylaxis**

Method: Stuart-Ord (inverse double arcsine square root)

| Stratum | Responding | Total |  |
| --- | --- | --- | --- |
| 1 | 18 | 80317 | GalindoSantana 2011 |

| Stratum | Proportion | 95% CI (exact) | |  |
| --- | --- | --- | --- | --- |
| 1 | 0.000224 | 0.000133 | 0.000354 | GalindoSantana 2011 |

| Stratum | Standardized effect | Variance | % Weights (fixed, random) | |  |
| --- | --- | --- | --- | --- | --- |
| 1 | 0.000224 | 0.000012 | 100 | 100 | GalindoSantana 2011 |

Fixed effects (inverse variance)

Pooled proportion = 0.00023 (95% CI = 0.000137 to 0.000347)

Non-combinability of studies

Cochran Q = 0 (df = 0) P = *

Moment-based estimate of between studies variance = 0

I_2_ (inconsistency) = *% (95% CI = *% to *%)

Random effects (DerSimonian-Laird)

Pooled proportion = 0.00MAEs023 (95% CI = 0.000137 to 0.000347)

Bias indicators

Begg-Mazumdar: Kendall's <too few strata> P = *

Egger: bias = * (95% CI = * to *) P = *

Harbord: bias = * (92.5% CI = * to *) P = *

**Proportion meta-analysis headache**

Method: Stuart-Ord (inverse double arcsine square root)

| Stratum | Responding | Total |  |
| --- | --- | --- | --- |
| 1 | 176 | 80317 | GalindoSantana 2011 |

| Stratum | Proportion | 95% CI (exact) | |  |
| --- | --- | --- | --- | --- |
| 1 | 0.002191 | 0.00188 | 0.00254 | GalindoSantana 2011 |

| Stratum | Standardized effect | Variance | % Weights (fixed, random) | |  |
| --- | --- | --- | --- | --- | --- |
| 1 | 0.002191 | 0.000012 | 100 | 100 | GalindoSantana 2011 |

Fixed effects (inverse variance)

Pooled proportion = 0.002198 (95% CI = 0.001886 to 0.002533)

Non-combinability of studies

Cochran Q = 0 (df = 0) P = *

Moment-based estimate of between studies variance = 0

I_2_ (inconsistency) = *% (95% CI = *% to *%)

Random effects (DerSimonian-Laird)

Pooled proportion = 0.002198 (95% CI = 0.001886 to 0.002533)

Bias indicators

Begg-Mazumdar: Kendall's <too few strata> P = *

Egger: bias = * (95% CI = * to *) P = *

Harbord: bias = * (92.5% CI = * to *) P = *

**Proportion meta-analysis Fever**

Method: Stuart-Ord (inverse double arcsine square root)

| Stratum | Responding | Total |  |
| --- | --- | --- | --- |
| 1 | 188 | 80317 | GalindoSantana 2011 |

| Stratum | Proportion | 95% CI (exact) | |  |
| --- | --- | --- | --- | --- |
| 1 | 0.002341 | 0.002018 | 0.0027 | GalindoSantana 2011 |

| Stratum | Standardized effect | Variance | % Weights (fixed, random) | |  |
| --- | --- | --- | --- | --- | --- |
| 1 | 0.002341 | 0.000012 | 100 | 100 | GalindoSantana 2011 |

Fixed effects (inverse variance)

Pooled proportion = 0.002347 (95% CI = 0.002024 to 0.002693)

Non-combinability of studies

Cochran Q = 0 (df = 0) P = *

Moment-based estimate of between studies variance = 0

I_2_ (inconsistency) = *% (95% CI = *% to *%)

Random effects (DerSimonian-Laird)

Pooled proportion = 0.002347 (95% CI = 0.002024 to 0.002693)

Bias indicators

Begg-Mazumdar: Kendall's <too few strata> P = *

Egger: bias = * (95% CI = * to *) P = *

Harbord: bias = * (92.5% CI = * to *) P = *

**Proportion meta-analysis vomiting**

Method: Stuart-Ord (inverse double arcsine square root)

| Stratum | Responding | Total |  |
| --- | --- | --- | --- |
| 1 | 70 | 80317 | GalindoSantana 2011 |

| Stratum | Proportion | 95% CI (exact) | |  |
| --- | --- | --- | --- | --- |
| 1 | 0.000872 | 0.000679 | 0.001101 | GalindoSantana 2011 |

| Stratum | Standardized effect | Variance | % Weights (fixed, random) | |  |
| --- | --- | --- | --- | --- | --- |
| 1 | 0.000872 | 0.000012 | 100 | 100 | GalindoSantana 2011 |

Fixed effects (inverse variance)

Pooled proportion = 0.000878 (95% CI = 0.000685 to 0.001094)

Non-combinability of studies

Cochran Q = 0 (df = 0) P = *

Moment-based estimate of between studies variance = 0

I_2_ (inconsistency) = *% (95% CI = *% to *%)

Random effects (DerSimonian-Laird)

Pooled proportion = 0.000878 (95% CI = 0.000685 to 0.001094)

Bias indicators

Begg-Mazumdar: Kendall's <too few strata> P = *

Egger: bias = * (95% CI = * to *) P = *

Harbord: bias = * (92.5% CI = * to *) P = *

**Proportion meta-analysis Joint Pain**

Method: Stuart-Ord (inverse double arcsine square root)

| Stratum | Responding | Total |  |
| --- | --- | --- | --- |
| 1 | 40 | 80317 | GalindoSantana 2011 |

| Stratum | Proportion | 95% CI (exact) | |  |
| --- | --- | --- | --- | --- |
| 1 | 0.000498 | 0.000356 | 0.000678 | GalindoSantana 2011 |

| Stratum | Standardized effect | Variance | % Weights (fixed, random) | |  |
| --- | --- | --- | --- | --- | --- |
| 1 | 0.000498 | 0.000012 | 100 | 100 | GalindoSantana 2011 |

Fixed effects (inverse variance)

Pooled proportion = 0.000504 (95% CI = 0.000361 to 0.000671)

Non-combinability of studies

Cochran Q = 0 (df = 0) P = *

Moment-based estimate of between studies variance = 0

I_2_ (inconsistency) = *% (95% CI = *% to *%)

Random effects (DerSimonian-Laird)

Pooled proportion = 0.000504 (95% CI = 0.000361 to 0.000671)

Bias indicators

Begg-Mazumdar: Kendall's <too few strata> P = *

Egger: bias = * (95% CI = * to *) P = *

Harbord: bias = * (92.5% CI = * to *) P = *

## ***3.2* *VACCINES CONTAINING ALUMINUM ADJUVANTS IN HUMANS***

**Proportion meta-analysis Abortion Humans**

Method: Stuart-Ord (inverse double arcsine square root)

| Stratum | Responding | Total |  |
| --- | --- | --- | --- |
| 1 | 3 | 40 | Kushner 2020 |
| 2 | 43 | 378 | Celzo 2020 (H) |
| 3 | 57 | 339 | Celzo 2020 (E) |
| 4 | 26 | 194 | Celzo 2020 (T) |
| 5 | 23 | 110 | Moro 2018 |
| 6 | 15 | 139 | Moro 2014 |
| 7 | 24 | 207 | Baril 2015 |
| 8 | 53 | 593 | Dana 2009 |
| 9 | 16 | 137 | Angelo 2014 |
| 10 | 5 | 9 | Angelo 2014 (Pass. S) |
| 11 | 10 | 160 | Angelo 2014 (Act. S, all) |
| 12 | 4 | 138 | Shakib 2013 |
| 13 | 22 | 132 | Zheteyeva 2012 |

| Stratum | Proportion | 95% CI (exact) | |  |
| --- | --- | --- | --- | --- |
| 1 | 0.075 | 0.015742 | 0.203865 | Kushner 2020 |
| 2 | 0.113757 | 0.08356 | 0.15016 | Celzo 2020 (H) |
| 3 | 0.168142 | 0.129907 | 0.21229 | Celzo 2020 (E) |
| 4 | 0.134021 | 0.08945 | 0.190175 | Celzo 2020 (T) |
| 5 | 0.209091 | 0.137403 | 0.297035 | Moro 2018 |
| 6 | 0.107914 | 0.06167 | 0.171736 | Moro 2014 |
| 7 | 0.115942 | 0.075716 | 0.167587 | Baril 2015 |
| 8 | 0.089376 | 0.067665 | 0.115276 | Dana 2009 |
| 9 | 0.116788 | 0.068249 | 0.182721 | Angelo 2014 |
| 10 | 0.555556 | 0.212009 | 0.863004 | Angelo 2014 (Pass. S) |
| 11 | 0.0625 | 0.030375 | 0.111935 | Angelo 2014 (Act. S, all) |
| 12 | 0.028986 | 0.007953 | 0.07255 | Shakib 2013 |
| 13 | 0.166667 | 0.107496 | 0.241391 | Zheteyeva 2012 |

| Stratum | Standardized effect | Variance | % Weights (fixed, random) | |  |
| --- | --- | --- | --- | --- | --- |
| 1 | 0.075 | 0.024691 | 1.568248 | 4.696135 | Kushner 2020 |
| 2 | 0.113757 | 0.002642 | 14.656341 | 9.630363 | Celzo 2020 (H) |
| 3 | 0.168142 | 0.002946 | 13.146176 | 9.49307 | Celzo 2020 (E) |
| 4 | 0.134021 | 0.005141 | 7.531462 | 8.605447 | Celzo 2020 (T) |
| 5 | 0.209091 | 0.00905 | 4.2788 | 7.377643 | Moro 2018 |
| 6 | 0.107914 | 0.007168 | 5.401742 | 7.921692 | Moro 2014 |
| 7 | 0.115942 | 0.004819 | 8.03485 | 8.725118 | Baril 2015 |
| 8 | 0.089376 | 0.001685 | 22.981607 | 10.090567 | Dana 2009 |
| 9 | 0.116788 | 0.007273 | 5.324298 | 7.889447 | Angelo 2014 |
| 10 | 0.555556 | 0.105263 | 0.367861 | 1.635002 | Angelo 2014 (Pass. S) |
| 11 | 0.0625 | 0.006231 | 6.214908 | 8.224044 | Angelo 2014 (Act. S, all) |
| 12 | 0.028986 | 0.00722 | 5.36302 | 7.905653 | Shakib 2013 |
| 13 | 0.166667 | 0.007547 | 5.130687 | 7.805818 | Zheteyeva 2012 |

Fixed effects (inverse variance)

Pooled proportion = 0.114866 (95% CI = 0.102858 to 0.127447)

Non-combinability of studies

Cochran Q = 53.643558 (df = 12) P < 0.0001

Moment-based estimate of between studies variance = 0.018343

I_2_ (inconsistency) = 77.6% (95% CI = 59.2% to 85.6%)

Random effects (DerSimonian-Laird)

Pooled proportion = 0.119826 (95% CI = 0.092723 to 0.149879)

Bias indicators

Begg-Mazumdar: Kendall's 0.358974 P = 0.1

Egger: bias = 2.77192 (95% CI = 0.089216 to 5.454625) P = 0.044

Harbord: bias = 2.081583 (92.5% CI = -1.066631 to 5.229798) P = 0.2201

**Proportion meta-analysis Fetal death**

Method: Stuart-Ord (inverse double arcsine square root)

| Stratum | Responding | Total |  |
| --- | --- | --- | --- |
| 1 | 11 | 593 | Dana 2009 |
| 2 | 1 | 793 | Petousis-Harris 2016 |

| Stratum | Proportion | 95% CI (exact) | |  |
| --- | --- | --- | --- | --- |
| 1 | 0.01855 | 0.009295 | 0.032948 | Dana 2009 |
| 2 | 0.001261 | 0.000032 | 0.007006 | Petousis-Harris 2016 |

| Stratum | Standardized effect | Variance | % Weights (fixed, random) | |  |
| --- | --- | --- | --- | --- | --- |
| 1 | 0.01855 | 0.001685 | 42.790195 | 49.432401 | Dana 2009 |
| 2 | 0.001261 | 0.00126 | 57.209805 | 50.567599 | Petousis-Harris 2016 |

Fixed effects (inverse variance)

Pooled proportion = 0.007079 (95% CI = 0.003351 to 0.012171)

Non-combinability of studies

Cochran Q = 12.702286 (df = 1) P = 0.0004

Moment-based estimate of between studies variance = 0.017233

I_2_ (inconsistency) = 92.1% (95% CI = *% to *%)

Random effects (DerSimonian-Laird)

Pooled proportion = 0.008196 (95% CI = 0.000017 to 0.03399)

Bias indicators

Begg-Mazumdar: Kendall's <too few strata> P = *

Egger: bias = * (95% CI = * to *) P = *

Harbord: bias = 37.129501 (92.5% CI = * to *) P = *

**Proportion meta-analysis Stillbirth Humans**

Method: Stuart-Ord (inverse double arcsine square root)

| Stratum | Responding | Total |  |
| --- | --- | --- | --- |
| 1 | 15 | 3045 | Madhi 2020 |
| 2 | 1 | 40 | Kushner 2020 |
| 3 | 2 | 378 | Celzo 2020 (H) |
| 4 | 3 | 339 | Celzo 2020 (E) |
| 5 | 2 | 194 | Celzo 2020 (T) |
| 6 | 2 | 110 | Moro 2018 |
| 7 | 1 | 139 | Moro 2014 |
| 8 | 0 | 49 | Swamy |
| 9 | 3 | 330 | Baril 2015 |
| 10 | 0 | 341 | Perrett |
| 11 | 0 | 160 | Angelo 2014 (Act. S, all) |
| 12 | 0 | 52 | Hoang 2016 Vietnam |
| 13 | 12 | 6185 | Donegan 2014 |
| 14 | 0 | 134 | Shakib 2013 |
| 15 | 0 | 650 | Berenson 2016 |
| 16 | 25 | 7152 | Morgan 2015 |
| 17 | 1 | 793 | Petousis-Harris 2016 |
| 18 | 2 | 132 | Zheteyeva 2012 |

| Stratum | Proportion | 95% CI (exact) | |  |
| --- | --- | --- | --- | --- |
| 1 | 0.004926 | 0.00276 | 0.008112 | Madhi 2020 |
| 2 | 0.025 | 0.000633 | 0.131586 | Kushner 2020 |
| 3 | 0.005291 | 0.000641 | 0.018981 | Celzo 2020 (H) |
| 4 | 0.00885 | 0.001829 | 0.025643 | Celzo 2020 (E) |
| 5 | 0.010309 | 0.001251 | 0.036743 | Celzo 2020 (T) |
| 6 | 0.018182 | 0.00221 | 0.064138 | Moro 2018 |
| 7 | 0.007194 | 0.000182 | 0.039431 | Moro 2014 |
| 8 | 0 | 0 | 0.072519 | Swamy [97.5% one-sided CI] |
| 9 | 0.009091 | 0.001879 | 0.026336 | Baril 2015 |
| 10 | 0 | 0 | 0.01076 | Perrett [97.5% one-sided CI] |
| 11 | 0 | 0 | 0.022792 | Angelo 2014 (Act. S, all) [97.5% one-sided CI] |
| 12 | 0 | 0 | 0.068482 | Hoang 2016 [97.5% one-sided CI] |
| 13 | 0.00194 | 0.001003 | 0.003387 | Donegan 2014 |
| 14 | 0 | 0 | 0.027153 | Shakib 2013 [97.5% one-sided CI] |
| 15 | 0 | 0 | 0.005659 | Berenson 2016 [97.5% one-sided CI] |
| 16 | 0.003496 | 0.002263 | 0.005156 | Morgan 2015 |
| 17 | 0.001261 | 0.000032 | 0.007006 | Petousis-Harris 2016 |
| 18 | 0.015152 | 0.00184 | 0.05366 | Zheteyeva 2012 |

| Stratum | Standardized effect | Variance | % Weights (fixed, random) | |  |
| --- | --- | --- | --- | --- | --- |
| 1 | 0.004926 | 0.000328 | 15.052887 | 14.382809 | Madhi 2020 |
| 2 | 0.025 | 0.024691 | 0.200178 | 0.734933 | Kushner 2020 |
| 3 | 0.005291 | 0.002642 | 1.870799 | 5.204488 | Celzo 2020 (H) |
| 4 | 0.00885 | 0.002946 | 1.678035 | 4.802471 | Celzo 2020 (E) |
| 5 | 0.010309 | 0.005141 | 0.961348 | 3.080724 | Celzo 2020 (T) |
| 6 | 0.018182 | 0.00905 | 0.546164 | 1.880662 | Moro 2018 |
| 7 | 0.007194 | 0.007168 | 0.689502 | 2.314678 | Moro 2014 |
| 8 | 0 | 0.020202 | 0.244662 | 0.890669 | Swamy [97.5% one-sided CI] |
| 9 | 0.009091 | 0.003026 | 1.633551 | 4.706392 | Baril 2015 |
| 10 | 0 | 0.002928 | 1.68792 | 4.823649 | Perrett [97.5% one-sided CI] |
| 11 | 0 | 0.006231 | 0.793298 | 2.615616 | Angelo 2014 (Act. S, all) [97.5% one-sided CI] |
| 12 | 0 | 0.019048 | 0.25949 | 0.941998 | Hoang 2016 [97.5% one-sided CI] |
| 13 | 0.00194 | 0.000162 | 30.572855 | 16.476142 | Donegan 2014 |
| 14 | 0 | 0.007435 | 0.664788 | 2.241407 | Shakib 2013 [97.5% one-sided CI] |
| 15 | 0 | 0.001537 | 3.215204 | 7.485273 | Berenson 2016 [97.5% one-sided CI] |
| 16 | 0.003496 | 0.00014 | 35.352412 | 16.796707 | Morgan 2015 |
| 17 | 0.001261 | 0.00126 | 3.922005 | 8.409462 | Petousis-Harris 2016 |
| 18 | 0.015152 | 0.007547 | 0.654903 | 2.211921 | Zheteyeva 2012 |

Fixed effects (inverse variance)

Pooled proportion = 0.003373 (95% CI = 0.002621 to 0.004219)

Non-combinability of studies

Cochran Q = 32.020434 (df = 17) P = 0.015

Moment-based estimate of between studies variance = 0.000984

I_2_ (inconsistency) = 46.9% (95% CI = 0% to 68.2%)

Random effects (DerSimonian-Laird)

Pooled proportion = 0.004137 (95% CI = 0.002589 to 0.006044)

Bias indicators

Begg-Mazumdar: Kendall's 0.150327 P = 0.4101

Egger: bias = 0.381637 (95% CI = -0.239754 to 1.003028) P = 0.2114

Harbord: bias = 0.895885 (92.5% CI = -0.045979 to 1.837748) P = 0.0889

**Proportion meta-analysis fetal growth retardation**

Method: Stuart-Ord (inverse double arcsine square root)

| Stratum | Responding | Total |  |
| --- | --- | --- | --- |
| 1 | 16 | 3008 | Madhi 2020 |
| 2 | 5 | 341 | Perrett |

| Stratum | Proportion | 95% CI (exact) | |  |
| --- | --- | --- | --- | --- |
| 1 | 0.005319 | 0.003043 | 0.008624 | Madhi 2020 |
| 2 | 0.014663 | 0.004778 | 0.033884 | Perrett |

| Stratum | Standardized effect | Variance | % Weights (fixed, random) | |  |
| --- | --- | --- | --- | --- | --- |
| 1 | 0.005319 | 0.000332 | 89.80597 | 61.588221 | Madhi 2020 |
| 2 | 0.014663 | 0.002928 | 10.19403 | 38.411779 | Perrett |

Fixed effects (inverse variance)

Pooled proportion = 0.006308 (95% CI = 0.00391 to 0.009271)

Non-combinability of studies

Cochran Q = 3.435037 (df = 1) P = 0.0638

Moment-based estimate of between studies variance = 0.00397

I_2_ (inconsistency) = 70.9% (95% CI = *% to *%)

Random effects (DerSimonian-Laird)

Pooled proportion = 0.008892 (95% CI = 0.001934 to 0.020844)

Bias indicators

Begg-Mazumdar: Kendall's <too few strata> P = *

Egger: bias = * (95% CI = * to *) P = *

Harbord: bias = 3.285629 (92.5% CI = * to *) P = *

**Proportion meta-analysis gestational diabetes**

Method: Stuart-Ord (inverse double arcsine square root)

| Stratum | Responding | Total |  |
| --- | --- | --- | --- |
| 1 | 2 | 1581 | Madhi 2020 |
| 2 | 179 | 1399 | Groom 2018 |
| 3 | 0 | 49 | Swamy |
| 4 | 0 | 341 | Perrett |
| 5 | 128 | 1140 | Groom 2019 |
| 6 | 1 | 793 | Petousis-Harris 2016 |
| 7 | 7 | 132 | Zheteyeva 2012 |

| Stratum | Proportion | 95% CI (exact) | |  |
| --- | --- | --- | --- | --- |
| 1 | 0.001265 | 0.000153 | 0.004562 | Madhi 2020 |
| 2 | 0.127949 | 0.110883 | 0.146593 | Groom 2018 |
| 3 | 0 | 0 | 0.072519 | Swamy [97.5% one-sided CI] |
| 4 | 0 | 0 | 0.01076 | Perrett [97.5% one-sided CI] |
| 5 | 0.112281 | 0.094534 | 0.132055 | Groom 2019 |
| 6 | 0.001261 | 0.000032 | 0.007006 | Petousis-Harris 2016 |
| 7 | 0.05303 | 0.021584 | 0.106211 | Zheteyeva 2012 |

| Stratum | Standardized effect | Variance | % Weights (fixed, random) | |  |
| --- | --- | --- | --- | --- | --- |
| 1 | 0.001265 | 0.000632 | 29.079709 | 14.736092 | Madhi 2020 |
| 2 | 0.127949 | 0.000715 | 25.733198 | 14.726379 | Groom 2018 |
| 3 | 0 | 0.020202 | 0.910177 | 12.73674 | Swamy [97.5% one-sided CI] |
| 4 | 0 | 0.002928 | 6.279305 | 14.469612 | Perrett [97.5% one-sided CI] |
| 5 | 0.112281 | 0.000877 | 20.970856 | 14.707248 | Groom 2019 |
| 6 | 0.001261 | 0.00126 | 14.59042 | 14.662241 | Petousis-Harris 2016 |
| 7 | 0.05303 | 0.007547 | 2.436334 | 13.961688 | Zheteyeva 2012 |

Fixed effects (inverse variance)

Pooled proportion = 0.036392 (95% CI = 0.031579 to 0.041532)

Non-combinability of studies

Cochran Q = 531.710722 (df = 6) P < 0.0001

Moment-based estimate of between studies variance = 0.124035

I_2_ (inconsistency) = 98.9% (95% CI = 98.7% to 99%)

Random effects (DerSimonian-Laird)

Pooled proportion = 0.026264 (95% CI = 0.000897 to 0.084868)

Bias indicators

Begg-Mazumdar: Kendall's 0.52381 P = 0.1361

Egger: bias = 6.688925 (95% CI = -1.540538 to 14.918388) P = 0.091

Harbord: bias = -4.098814 (92.5% CI = -24.259915 to 16.062287) P = 0.6676

**Proportion meta-analysis Hypertensive disorders of pregnancy**

Method: Stuart-Ord (inverse double arcsine square root)

| Stratum | Responding | Total |  |
| --- | --- | --- | --- |
| 1 | 91 | 3045 | Madhi 2020 Argentina LMIC (Hoang, Katz, Nuñez) |
| 2 | 1 | 22 | Munoz 2019 |
| 3 | 68 | 1399 | Groom 2018 |
| 4 | 6 | 378 | Celzo 2020 (H) |
| 5 | 4 | 339 | Celzo 2020 (E) |
| 6 | 0 | 194 | Celzo 2020 (T) |
| 7 | 3 | 110 | Moro 2018 |
| 8 | 5 | 49 | Swamy |
| 9 | 76 | 1018 | Mohamed |
| 10 | 44 | 1140 | Groom 2019 |
| 11 | 497 | 26229 | Kharbanda 2014 (pregnant women Vaccinated at <20 |
| 12 | 2 | 57 | Maertens 2016 |
| 13 | 22 | 6185 | Donegan 2014 |
| 14 | 2 | 793 | Petousis-Harris 2016 |

| Stratum | Proportion | 95% CI (exact) | |  |
| --- | --- | --- | --- | --- |
| 1 | 0.029885 | 0.024128 | 0.036567 | Madhi 2020 |
| 2 | 0.045455 | 0.00115 | 0.228444 | Munoz 2019 |
| 3 | 0.048606 | 0.037939 | 0.061216 | Groom 2018 |
| 4 | 0.015873 | 0.005847 | 0.034227 | Celzo 2020 (H) |
| 5 | 0.011799 | 0.003224 | 0.029934 | Celzo 2020 (E) |
| 6 | 0 | 0 | 0.018835 | Celzo 2020 (T) [97.5% one-sided CI] |
| 7 | 0.027273 | 0.00566 | 0.077637 | Moro 2018 |
| 8 | 0.102041 | 0.033972 | 0.222277 | Swamy |
| 9 | 0.074656 | 0.059268 | 0.092555 | Mohamed |
| 10 | 0.038596 | 0.028182 | 0.05147 | Groom 2019 |
| 11 | 0.018948 | 0.017333 | 0.020672 | Kharbanda 2014 (pregnant women Vaccinated at <20 |
| 12 | 0.035088 | 0.004278 | 0.121071 | Maertens 2016 |
| 13 | 0.003557 | 0.00223 | 0.00538 | Donegan 2014 |
| 14 | 0.002522 | 0.000306 | 0.009081 | Petousis-Harris 2016 |

| Stratum | Standardized effect | Variance | % Weights (fixed, random) | |  |
| --- | --- | --- | --- | --- | --- |
| 1 | 0.029885 | 0.000328 | 7.434395 | 9.008955 | Madhi 2020 |
| 2 | 0.045455 | 0.044444 | 0.054925 | 2.252119 | Munoz 2019 |
| 3 | 0.048606 | 0.000715 | 3.416331 | 8.778403 | Groom 2018 |
| 4 | 0.015873 | 0.002642 | 0.923959 | 7.784155 | Celzo 2020 (H) |
| 5 | 0.011799 | 0.002946 | 0.828756 | 7.647764 | Celzo 2020 (E) |
| 6 | 0 | 0.005141 | 0.474796 | 6.787321 | Celzo 2020 (T) [97.5% one-sided CI] |
| 7 | 0.027273 | 0.00905 | 0.269742 | 5.654914 | Moro 2018 |
| 8 | 0.102041 | 0.020202 | 0.120835 | 3.831066 | Swamy |
| 9 | 0.074656 | 0.000982 | 2.486269 | 8.625619 | Mohamed |
| 10 | 0.038596 | 0.000877 | 2.784084 | 8.685013 | Groom 2019 |
| 11 | 0.018948 | 0.000038 | 64.029049 | 9.190351 | Kharbanda 2014 (pregnant women Vaccinated at <20 |
| 12 | 0.035088 | 0.017391 | 0.140364 | 4.170032 | Maertens 2016 |
| 13 | 0.003557 | 0.000162 | 15.099475 | 9.112249 | Donegan 2014 |
| 14 | 0.002522 | 0.00126 | 1.937019 | 8.472039 | Petousis-Harris 2016 |

Fixed effects (inverse variance)

Pooled proportion = 0.018199 (95% CI = 0.016928 to 0.019516)

Non-combinability of studies

Cochran Q = 341.953194 (df = 13) P < 0.0001

Moment-based estimate of between studies variance = 0.014376

I_2_ (inconsistency) = 96.2% (95% CI = 95.2% to 96.9%)

Random effects (DerSimonian-Laird)

Pooled proportion = 0.024845 (95% CI = 0.014959 to 0.037147)

Bias indicators

Begg-Mazumdar: Kendall's 0.076923 P = 0.7472

Egger: bias = 2.397813 (95% CI = -1.308672 to 6.104299) P = 0.1841

Harbord: bias = 2.319136 (92.5% CI = -1.567839 to 6.206111) P = 0.2674

**Proportion meta-analysis Low birth weight**

Method: Stuart-Ord (inverse double arcsine square root)

| Stratum | Responding | Total |  |
| --- | --- | --- | --- |
| 1 | 150 | 3008 | Madhi 2020 |
| 2 | 78 | 1399 | Groom 2018 |
| 3 | 1 | 49 | Swamy |
| 4 | 50 | 1018 | Mohamed |
| 5 | 59 | 1140 | Groom 2019 |
| 6 | 126 | 6185 | Donegan 2014 |
| 7 | 61 | 1109 | Berenson 2016 |
| 8 | 714 | 7152 | Morgan 2015 |

| Stratum | Proportion | 95% CI (exact) | |  |
| --- | --- | --- | --- | --- |
| 1 | 0.049867 | 0.042363 | 0.058261 | Madhi 2020 |
| 2 | 0.055754 | 0.044317 | 0.069098 | Groom 2018 |
| 3 | 0.020408 | 0.000517 | 0.108542 | Swamy |
| 4 | 0.049116 | 0.036671 | 0.064244 | Mohamed |
| 5 | 0.051754 | 0.039628 | 0.066255 | Groom 2019 |
| 6 | 0.020372 | 0.016998 | 0.024208 | Donegan 2014 |
| 7 | 0.055005 | 0.042332 | 0.070098 | Berenson 2016 |
| 8 | 0.099832 | 0.092979 | 0.107014 | Morgan 2015 |

| Stratum | Standardized effect | Variance | % Weights (fixed, random) | |  |
| --- | --- | --- | --- | --- | --- |
| 1 | 0.049867 | 0.000332 | 14.282662 | 13.329025 | Madhi 2020 |
| 2 | 0.055754 | 0.000715 | 6.644037 | 13.137362 | Groom 2018 |
| 3 | 0.020408 | 0.020202 | 0.234998 | 7.579534 | Swamy |
| 4 | 0.049116 | 0.000982 | 4.835264 | 13.006546 | Mohamed |
| 5 | 0.051754 | 0.000877 | 5.414451 | 13.057635 | Groom 2019 |
| 6 | 0.020372 | 0.000162 | 29.365268 | 13.416468 | Donegan 2014 |
| 7 | 0.055005 | 0.000901 | 5.267281 | 13.045683 | Berenson 2016 |
| 8 | 0.099832 | 0.00014 | 33.956039 | 13.427747 | Morgan 2015 |

Fixed effects (inverse variance)

Pooled proportion = 0.054521 (95% CI = 0.051496 to 0.057628)

Non-combinability of studies

Cochran Q = 424.062727 (df = 7) P < 0.0001

Moment-based estimate of between studies variance = 0.025862

I_2_ (inconsistency) = 98.3% (95% CI = 98% to 98.6%)

Random effects (DerSimonian-Laird)

Pooled proportion = 0.050733 (95% CI = 0.028385 to 0.079101)

Bias indicators

Begg-Mazumdar: Kendall's -0.285714 P = 0.2751

Egger: bias = 5.407514 (95% CI = -6.249922 to 17.06495) P = 0.2997

Harbord: bias = -2.719341 (92.5% CI = -15.935939 to 10.497257) P = 0.6736

**Proportion meta-analysis Small for gestational age**

Method: Stuart-Ord (inverse double arcsine square root)

| Stratum | Responding | Total |  |
| --- | --- | --- | --- |
| 1 | 151 | 3008 | Madhi 2020 |
| 2 | 120 | 1399 | Groom 2018 |
| 3 | 14 | 330 | Baril 2015 |
| 4 | 2 | 341 | Perrett |
| 5 | 114 | 1017 | Mohamed |
| 6 | 124 | 1140 | Groom 2019 |
| 7 | 978 | 11351 | Kharbanda 2014 (Vaccinated at 27-=36 wk)gestation |
| 8 | 2214 | 26229 | Kharbanda 2014 |
| 9 | 31 | 650 | Berenson 2016 |

| Stratum | Proportion | 95% CI (exact) | |  |
| --- | --- | --- | --- | --- |
| 1 | 0.050199 | 0.042671 | 0.058617 | Madhi 2020 |
| 2 | 0.085776 | 0.071626 | 0.101692 | Groom 2018 |
| 3 | 0.042424 | 0.023385 | 0.070157 | Baril 2015 |
| 4 | 0.005865 | 0.000711 | 0.021025 | Perrett |
| 5 | 0.112094 | 0.093358 | 0.133108 | Mohamed |
| 6 | 0.108772 | 0.091287 | 0.128304 | Groom 2019 |
| 7 | 0.08616 | 0.08106 | 0.091473 | Kharbanda 2014 (Vaccinated at 27-=36 wk)gestation |
| 8 | 0.08441 | 0.081073 | 0.08784 | Kharbanda 2014 |
| 9 | 0.047692 | 0.032631 | 0.067015 | Berenson 2016 |

| Stratum | Standardized effect | Variance | % Weights (fixed, random) | |  |
| --- | --- | --- | --- | --- | --- |
| 1 | 0.050199 | 0.000332 | 6.616523 | 12.446304 | Madhi 2020 |
| 2 | 0.085776 | 0.000715 | 3.077887 | 11.660147 | Groom 2018 |
| 3 | 0.042424 | 0.003026 | 0.726861 | 8.437124 | Baril 2015 |
| 4 | 0.005865 | 0.002928 | 0.751053 | 8.536629 | Perrett |
| 5 | 0.112094 | 0.000983 | 2.237764 | 11.165092 | Mohamed |
| 6 | 0.108772 | 0.000877 | 2.508275 | 11.355584 | Groom 2019 |
| 7 | 0.08616 | 0.000088 | 24.965086 | 13.00692 | Kharbanda 2014 (Vaccinated at 27-=36 wk)gestation |
| 8 | 0.08441 | 0.000038 | 57.685921 | 13.127869 | Kharbanda 2014 |
| 9 | 0.047692 | 0.001537 | 1.430629 | 10.264332 | Berenson 2016 |

Fixed effects (inverse variance)

Pooled proportion = 0.08179 (95% CI = 0.079289 to 0.084326)

Non-combinability of studies

Cochran Q = 153.112304 (df = 8) P < 0.0001

Moment-based estimate of between studies variance = 0.005336

I_2_ (inconsistency) = 94.8% (95% CI = 92.6% to 96.1%)

Random effects (DerSimonian-Laird)

Pooled proportion = 0.068386 (95% CI = 0.055837 to 0.082104)

Bias indicators

Begg-Mazumdar: Kendall's -0.111111 P = 0.6122

Egger: bias = -3.209372 (95% CI = -11.568655 to 5.149911) P = 0.3941

Harbord: bias = -2.061254 (92.5% CI = -6.055019 to 1.93251) P = 0.3165

**Proportion meta-analysis Preterm birth**

Method: Stuart-Ord (inverse double arcsine square root)

| Stratum | Responding | Total |  |
| --- | --- | --- | --- |
| 1 | 174 | 3045 | Madhi 2020 |
| 2 | 1 | 22 | Munoz 2019 |
| 3 | 107 | 1399 | Groom 2018 |
| 4 | 2 | 40 | Kushner 2020 |
| 5 | 13 | 378 | Celzo 2020 (H) |
| 6 | 7 | 339 | Celzo 2020 (E) |
| 7 | 3 | 194 | Celzo 2020 (T) |
| 8 | 7 | 110 | Moro 2018 |
| 9 | 7 | 139 | Moro 2014 |
| 10 | 3 | 49 | Swamy |
| 11 | 16 | 330 | Baril 2015 |
| 12 | 11 | 341 | Perrett |
| 13 | 61 | 1019 | Mohamed |
| 14 | 67 | 1140 | Groom 2019 |
| 15 | 602 | 11351 | Kharbanda 2014 (Vaccinated at 27-36 weeks of gestation |
| 16 | 1527 | 26229 | Kharbanda 2014 |
| 17 | 8 | 134 | Shakib 2013 |
| 18 | 59 | 650 | Berenson 2016 |
| 19 | 427 | 7152 | Morgan 2015 |
| 20 | 2 | 132 | Zheteyeva 2012 |

| Stratum | Proportion | 95% CI (exact) | |  |
| --- | --- | --- | --- | --- |
| 1 | 0.057143 | 0.049163 | 0.065986 | Madhi 2020 |
| 2 | 0.045455 | 0.00115 | 0.228444 | Munoz 2019 |
| 3 | 0.076483 | 0.0631 | 0.091675 | Groom 2018 |
| 4 | 0.05 | 0.006114 | 0.169197 | Kushner 2020 |
| 5 | 0.034392 | 0.018437 | 0.058093 | Celzo 2020 (H) |
| 6 | 0.020649 | 0.008341 | 0.04208 | Celzo 2020 (E) |
| 7 | 0.015464 | 0.0032 | 0.044525 | Celzo 2020 (T) |
| 8 | 0.063636 | 0.025966 | 0.126726 | Moro 2018 |
| 9 | 0.05036 | 0.020484 | 0.101007 | Moro 2014 |
| 10 | 0.061224 | 0.012808 | 0.168659 | Swamy |
| 11 | 0.048485 | 0.027964 | 0.077544 | Baril 2015 |
| 12 | 0.032258 | 0.016211 | 0.056985 | Perrett |
| 13 | 0.059863 | 0.046096 | 0.076235 | Mohamed |
| 14 | 0.058772 | 0.045834 | 0.074041 | Groom 2019 |
| 15 | 0.053035 | 0.048983 | 0.057318 | Kharbanda 2014 (Vaccinated at 27-=36 wk)gestation |
| 16 | 0.058218 | 0.055414 | 0.06112 | Kharbanda 2014 |
| 17 | 0.059701 | 0.026124 | 0.114247 | Shakib 2013 |
| 18 | 0.090769 | 0.069815 | 0.115524 | Berenson 2016 |
| 19 | 0.059704 | 0.054323 | 0.065447 | Morgan 2015 |
| 20 | 0.015152 | 0.00184 | 0.05366 | Zheteyeva 2012 |

| Stratum | Standardized effect | Variance | % Weights (fixed, random) | |  |
| --- | --- | --- | --- | --- | --- |
| 1 | 0.057143 | 0.000328 | 5.618693 | 10.185233 | Madhi 2020 |
| 2 | 0.045455 | 0.044444 | 0.041511 | 0.320559 | Munoz 2019 |
| 3 | 0.076483 | 0.000715 | 2.58196 | 8.023741 | Groom 2018 |
| 4 | 0.05 | 0.024691 | 0.074719 | 0.566018 | Kushner 2020 |
| 5 | 0.034392 | 0.002642 | 0.698301 | 3.896564 | Celzo 2020 (H) |
| 6 | 0.020649 | 0.002946 | 0.626349 | 3.604615 | Celzo 2020 (E) |
| 7 | 0.015464 | 0.005141 | 0.358836 | 2.33748 | Celzo 2020 (T) |
| 8 | 0.063636 | 0.00905 | 0.203863 | 1.437847 | Moro 2018 |
| 9 | 0.05036 | 0.007168 | 0.257366 | 1.764793 | Moro 2014 |
| 10 | 0.061224 | 0.020202 | 0.091323 | 0.685276 | Swamy |
| 11 | 0.048485 | 0.003026 | 0.609745 | 3.534625 | Baril 2015 |
| 12 | 0.032258 | 0.002928 | 0.630039 | 3.620032 | Perrett |
| 13 | 0.059863 | 0.000981 | 1.880892 | 6.999351 | Mohamed |
| 14 | 0.058772 | 0.000877 | 2.104127 | 7.366844 | Groom 2019 |
| 15 | 0.053035 | 0.000088 | 20.942568 | 12.235895 | Kharbanda 2014 (Vaccinated at 27-=36 wk)gestation |
| 16 | 0.058218 | 0.000038 | 48.391233 | 12.770653 | Kharbanda 2014 |
| 17 | 0.059701 | 0.007435 | 0.248141 | 1.709725 | Shakib 2013 |
| 18 | 0.090769 | 0.001537 | 1.200118 | 5.525567 | Berenson 2016 |
| 19 | 0.059704 | 0.00014 | 13.195764 | 11.727633 | Morgan 2015 |
| 20 | 0.015152 | 0.007547 | 0.244451 | 1.687549 | Zheteyeva 2012 |

Fixed effects (inverse variance)

Pooled proportion = 0.057186 (95% CI = 0.055247 to 0.059157)

Non-combinability of studies

Cochran Q = 60.910062 (df = 19) P < 0.0001

Moment-based estimate of between studies variance = 0.001105

I_2_ (inconsistency) = 68.8% (95% CI = 46.6% to 79.3%)

Random effects (DerSimonian-Laird)

Pooled proportion = 0.055236 (95% CI = 0.049951 to 0.06077)

Bias indicators

Begg-Mazumdar: Kendall's -0.136842 P = 0.3859

Egger: bias = -0.732418 (95% CI = -1.972354 to 0.507518) P = 0.2305

Harbord: bias = -0.484008 (92.5% CI = -1.452083 to 0.484066) P = 0.3572

**Proportion meta-analysis any congenital malformation Humans**

Method: Stuart-Ord (inverse double arcsine square root)

| Stratum | Responding | Total |  |
| --- | --- | --- | --- |
| 1 | 6 | 3008 | Madhi 2020 |
| 2 | 1 | 22 | Munoz 2019 |
| 3 | 1 | 40 | Kushner 2020 |
| 4 | 19 | 378 | Celzo 2020 (H) |
| 5 | 29 | 339 | Celzo 2020 (E) |
| 6 | 10 | 194 | Celzo 2020 (T) |
| 7 | 5 | 110 | Moro 2018 |
| 8 | 2 | 139 | Moro 2014 |
| 9 | 7 | 48 | Swamy |
| 10 | 9 | 210 | Baril 2015 |
| 11 | 9 | 341 | Perrett |
| 12 | 20 | 593 | Dana 2009 |
| 13 | 13 | 160 | Angelo 2014 (Act. S, all) |
| 14 | 5 | 134 | Shakib 2013 |
| 15 | 15 | 650 | Berenson 2016 |
| 16 | 84 | 7152 | Morgan 2015 |

| Stratum | Proportion | 95% CI (exact) | |  |
| --- | --- | --- | --- | --- |
| 1 | 0.001995 | 0.000732 | 0.004336 | Madhi 2020 |
| 2 | 0.045455 | 0.00115 | 0.228444 | Munoz 2019 |
| 3 | 0.025 | 0.000633 | 0.131586 | Kushner 2020 |
| 4 | 0.050265 | 0.03053 | 0.077384 | Celzo 2020 (H) |
| 5 | 0.085546 | 0.058039 | 0.120547 | Celzo 2020 (E) |
| 6 | 0.051546 | 0.024992 | 0.092751 | Celzo 2020 (T) |
| 7 | 0.045455 | 0.014921 | 0.102892 | Moro 2018 |
| 8 | 0.014388 | 0.001747 | 0.051008 | Moro 2014 |
| 9 | 0.145833 | 0.060704 | 0.277638 | Swamy |
| 10 | 0.042857 | 0.019781 | 0.079795 | Baril 2015 |
| 11 | 0.026393 | 0.012138 | 0.049509 | Perrett |
| 12 | 0.033727 | 0.020721 | 0.051609 | Dana 2009 |
| 13 | 0.08125 | 0.043972 | 0.134932 | Angelo 2014 (Act. S, all) |
| 14 | 0.037313 | 0.012225 | 0.084928 | Shakib 2013 |
| 15 | 0.023077 | 0.012972 | 0.037777 | Berenson 2016 |
| 16 | 0.011745 | 0.009379 | 0.014521 | Morgan 2015 |

| Stratum | Standardized effect | Variance | % Weights (fixed, random) | |  |
| --- | --- | --- | --- | --- | --- |
| 1 | 0.001995 | 0.000332 | 22.242348 | 7.933835 | Madhi 2020 |
| 2 | 0.045455 | 0.044444 | 0.166346 | 2.64625 | Munoz 2019 |
| 3 | 0.025 | 0.024691 | 0.299423 | 3.771932 | Kushner 2020 |
| 4 | 0.050265 | 0.002642 | 2.798314 | 7.18242 | Celzo 2020 (H) |
| 5 | 0.085546 | 0.002946 | 2.509981 | 7.094129 | Celzo 2020 (E) |
| 6 | 0.051546 | 0.005141 | 1.437971 | 6.514715 | Celzo 2020 (T) |
| 7 | 0.045455 | 0.00905 | 0.816945 | 5.687862 | Moro 2018 |
| 8 | 0.014388 | 0.007168 | 1.031347 | 6.057966 | Moro 2014 |
| 9 | 0.145833 | 0.020619 | 0.358569 | 4.13457 | Swamy |
| 10 | 0.042857 | 0.004751 | 1.556262 | 6.610806 | Baril 2015 |
| 11 | 0.026393 | 0.002928 | 2.524767 | 7.099089 | Perrett |
| 12 | 0.033727 | 0.001685 | 4.387846 | 7.475825 | Dana 2009 |
| 13 | 0.08125 | 0.006231 | 1.186604 | 6.261076 | Angelo 2014 (Act. S, all) |
| 14 | 0.037313 | 0.007435 | 0.994381 | 6.00264 | Shakib 2013 |
| 15 | 0.023077 | 0.001537 | 4.809256 | 7.523234 | Berenson 2016 |
| 16 | 0.011745 | 0.00014 | 52.879639 | 8.003653 | Morgan 2015 |

Fixed effects (inverse variance)

Pooled proportion = 0.014222 (95% CI = 0.012296 to 0.016286)

Non-combinability of studies

Cochran Q = 210.209525 (df = 15) P < 0.0001

Moment-based estimate of between studies variance = 0.021744

I_2_ (inconsistency) = 92.9% (95% CI = 90.5% to 94.4%)

Random effects (DerSimonian-Laird)

Pooled proportion = 0.039025 (95% CI = 0.024708 to 0.056442)

Bias indicators

Begg-Mazumdar: Kendall's -0.183333 P = 0.3057

Egger: bias = 2.686817 (95% CI = 1.479818 to 3.893817) P = 0.0003

Harbord: bias = 5.044128 (92.5% CI = 2.585324 to 7.502931) P = 0.0015

**Proportion meta-analysis Neonatal death Humans**

Method: Stuart-Ord (inverse double arcsine square root)

| Stratum | Responding | Total |  |
| --- | --- | --- | --- |
| 1 | 17 | 3008 | Madhi 2020 |
| 2 | 0 | 22 | Munoz 2019 |
| 3 | 1 | 110 | Moro 2018 |
| 4 | 0 | 341 | Perrett |
| 5 | 1 | 593 | Dana 2009 |
| 6 | 2 | 6185 | Donegan 2014 |
| 7 | 2 | 7152 | Morgan 2015 |
| 8 | 2 | 793 | Petousis-Harris 2016 |

| Stratum | Proportion | 95% CI (exact) | |  |
| --- | --- | --- | --- | --- |
| 1 | 0.005652 | 0.003296 | 0.009033 | Madhi 2020 |
| 2 | 0 | 0 | 0.154373 | Munoz 2019 [97.5% one-sided CI] |
| 3 | 0.009091 | 0.00023 | 0.049611 | Moro 2018 |
| 4 | 0 | 0 | 0.01076 | Perrett [97.5% one-sided CI] |
| 5 | 0.001686 | 0.000043 | 0.00936 | Dana 2009 |
| 6 | 0.000323 | 0.000039 | 0.001168 | Donegan 2014 |
| 7 | 0.00028 | 0.000034 | 0.00101 | Morgan 2015 |
| 8 | 0.002522 | 0.000306 | 0.009081 | Petousis-Harris 2016 |

| Stratum | Standardized effect | Variance | % Weights (fixed, random) | |  |
| --- | --- | --- | --- | --- | --- |
| 1 | 0.005652 | 0.000332 | 16.522957 | 18.567458 | Madhi 2020 |
| 2 | 0 | 0.044444 | 0.123572 | 1.108042 | Munoz 2019 [97.5% one-sided CI] |
| 3 | 0.009091 | 0.00905 | 0.606876 | 4.513367 | Moro 2018 |
| 4 | 0 | 0.002928 | 1.875549 | 9.634156 | Perrett [97.5% one-sided CI] |
| 5 | 0.001686 | 0.001685 | 3.259556 | 12.519118 | Dana 2009 |
| 6 | 0.000323 | 0.000162 | 33.971331 | 19.773292 | Donegan 2014 |
| 7 | 0.00028 | 0.00014 | 39.282184 | 19.939075 | Morgan 2015 |
| 8 | 0.002522 | 0.00126 | 4.357975 | 13.945492 | Petousis-Harris 2016 |

Fixed effects (inverse variance)

Pooled proportion = 0.00103 (95% CI = 0.000617 to 0.001548)

Non-combinability of studies

Cochran Q = 38.428586 (df = 7) P < 0.0001

Moment-based estimate of between studies variance = 0.002467

I_2_ (inconsistency) = 81.8% (95% CI = 61.5% to 89.1%)

Random effects (DerSimonian-Laird)

Pooled proportion = 0.002004 (95% CI = 0.000503 to 0.004499)

Bias indicators

Begg-Mazumdar: Kendall's 0.142857 P = 0.7195

Egger: bias = 0.976155 (95% CI = -0.464537 to 2.416847) P = 0.1484

Harbord: bias = 1.589663 (92.5% CI = -2.687992 to 5.867317) P = 0.4546

**Proportion meta-analysis postpartum hemorrhage (PPH)**

Method: Stuart-Ord (inverse double arcsine square root)

| Stratum | Responding | Total |  |
| --- | --- | --- | --- |
| 1 | 67 | 3045 | Madhi 2020 |
| 2 | 14 | 378 | Celzo 2020 (H) |
| 3 | 6 | 339 | Celzo 2020 (E) |
| 4 | 0 | 194 | Celzo 2020 (T) |
| 5 | 3 | 49 | Swamy |
| 6 | 89 | 1018 | Mohamed |
| 7 | 59 | 6185 | Donegan 2014 |

| Stratum | Proportion | 95% CI (exact) | |  |
| --- | --- | --- | --- | --- |
| 1 | 0.022003 | 0.017092 | 0.02786 | Madhi 2020 |
| 2 | 0.037037 | 0.020394 | 0.061362 | Celzo 2020 (H) |
| 3 | 0.017699 | 0.006522 | 0.038124 | Celzo 2020 (E) |
| 4 | 0 | 0 | 0.018835 | Celzo 2020 (T) [97.5% one-sided CI] |
| 5 | 0.061224 | 0.012808 | 0.168659 | Swamy |
| 6 | 0.087426 | 0.070797 | 0.106485 | Mohamed |
| 7 | 0.009539 | 0.007269 | 0.012288 | Donegan 2014 |

| Stratum | Standardized effect | Variance | % Weights (fixed, random) | |  |
| --- | --- | --- | --- | --- | --- |
| 1 | 0.022003 | 0.000328 | 27.164073 | 16.226309 | Madhi 2020 |
| 2 | 0.037037 | 0.002642 | 3.375998 | 14.773717 | Celzo 2020 (H) |
| 3 | 0.017699 | 0.002946 | 3.028141 | 14.602241 | Celzo 2020 (E) |
| 4 | 0 | 0.005141 | 1.734826 | 13.470979 | Celzo 2020 (T) [97.5% one-sided CI] |
| 5 | 0.061224 | 0.020202 | 0.441511 | 8.796819 | Swamy |
| 6 | 0.087426 | 0.000982 | 9.084422 | 15.787866 | Mohamed |
| 7 | 0.009539 | 0.000162 | 55.17103 | 16.342069 | Donegan 2014 |

Fixed effects (inverse variance)

Pooled proportion = 0.018051 (95% CI = 0.015669 to 0.020597)

Non-combinability of studies

Cochran Q = 164.996272 (df = 6) P < 0.0001

Moment-based estimate of between studies variance = 0.023203

I_2_ (inconsistency) = 96.4% (95% CI = 94.9% to 97.2%)

Random effects (DerSimonian-Laird)

Pooled proportion = 0.027124 (95% CI = 0.010962 to 0.050213)

Bias indicators

Begg-Mazumdar: Kendall's 0.333333 P = 0.3813

Egger: bias = 3.090097 (95% CI = -1.941626 to 8.121821) P = 0.1752

Harbord: bias = 4.384438 (92.5% CI = -5.959363 to 14.72824) P = 0.3855

**Proportion meta-analysis antenatal bleeding**

Method: Stuart-Ord (inverse double arcsine square root)

| Stratum | Responding | Total |  |
| --- | --- | --- | --- |
| 1 | 3 | 793 | Petousis-Harris 2016 |

| Stratum | Proportion | 95% CI (exact) | |  |
| --- | --- | --- | --- | --- |
| 1 | 0.003783 | 0.000781 | 0.011016 | Petousis-Harris 2016 |

| Stratum | Standardized effect | Variance | % Weights (fixed, random) | |  |
| --- | --- | --- | --- | --- | --- |
| 1 | 0.003783 | 0.00126 | 100 | 100 | Petousis-Harris 2016 |

Fixed effects (inverse variance)

Pooled proportion = 0.004386 (95% CI = 0.000991 to 0.010179)

Non-combinability of studies

Cochran Q = 0 (df = 0) P = *

Moment-based estimate of between studies variance = 0

I_2_ (inconsistency) = *% (95% CI = *% to *%)

Random effects (DerSimonian-Laird)

Pooled proportion = 0.004386 (95% CI = 0.000991 to 0.010179)

Bias indicators

Begg-Mazumdar: Kendall's <too few strata> P = *

Egger: bias = * (95% CI = * to *) P = *

Harbord: bias = * (92.5% CI = * to *) P = *

**Proportion meta-analysis Neonatal infections**

Method: Stuart-Ord (inverse double arcsine square root)

| Stratum | Responding | Total |  |
| --- | --- | --- | --- |
| 1 | 342 | 3008 | Madhi 2020 |
| 2 | 8 | 22 | Munoz 2019 |
| 3 | 2 | 139 | Moro 2014 |
| 4 | 3 | 7152 | Morgan 2015 |

| Stratum | Proportion | 95% CI (exact) | |  |
| --- | --- | --- | --- | --- |
| 1 | 0.113697 | 0.102566 | 0.125585 | Madhi 2020 |
| 2 | 0.363636 | 0.171979 | 0.593423 | Munoz 2019 |
| 3 | 0.014388 | 0.001747 | 0.051008 | Moro 2014 |
| 4 | 0.000419 | 0.000087 | 0.001225 | Morgan 2015 |

| Stratum | Standardized effect | Variance | % Weights (fixed, random) | |  |
| --- | --- | --- | --- | --- | --- |
| 1 | 0.113697 | 0.000332 | 29.14366 | 26.397255 | Madhi 2020 |
| 2 | 0.363636 | 0.044444 | 0.21796 | 21.650382 | Munoz 2019 |
| 3 | 0.014388 | 0.007168 | 1.351351 | 25.529816 | Moro 2014 |
| 4 | 0.000419 | 0.00014 | 69.287029 | 26.422546 | Morgan 2015 |

Fixed effects (inverse variance)

Pooled proportion = 0.014049 (95% CI = 0.011869 to 0.016409)

Non-combinability of studies

Cochran Q = 904.572678 (df = 3) P < 0.0001

Moment-based estimate of between studies variance = 0.200862

I_2_ (inconsistency) = 99.7% (95% CI = 99.6% to 99.7%)

Random effects (DerSimonian-Laird)

Pooled proportion = 0.07226 (95% CI = 0.002144 to 0.22817)

Bias indicators

Begg-Mazumdar: Kendall's 0.333333 P = 0.75

Egger: bias = 7.979732 (95% CI = -17.678092 to 33.637556) P = 0.3127

Harbord: bias = 14.379124 (92.5% CI = -66.091657 to 94.849905) P = 0.6011

**Proportion meta-analysis Neonatal encephalopathy**

Method: Stuart-Ord (inverse double arcsine square root)

| Stratum | Responding | Total |  |
| --- | --- | --- | --- |
| 1 | 7 | 3008 | Madhi 2020 |
| 2 | 0 | 341 | Perrett |

| Stratum | Proportion | 95% CI (exact) | |  |
| --- | --- | --- | --- | --- |
| 1 | 0.002327 | 0.000936 | 0.004789 | Madhi 2020 |
| 2 | 0 | 0 | 0.01076 | Perrett [97.5% one-sided CI] |

| Stratum | Standardized effect | Variance | % Weights (fixed, random) | |  |
| --- | --- | --- | --- | --- | --- |
| 1 | 0.002327 | 0.000332 | 89.80597 | 89.80597 | Madhi 2020 |
| 2 | 0 | 0.002928 | 10.19403 | 10.19403 | Perrett [97.5% one-sided CI] |

Fixed effects (inverse variance)

Pooled proportion = 0.002263 (95% CI = 0.000939 to 0.004157)

Non-combinability of studies

Cochran Q = 0.641528 (df = 1) P = 0.4232

Moment-based estimate of between studies variance = 0

I_2_ (inconsistency) = 0% (95% CI = *% to *%)

Random effects (DerSimonian-Laird)

Pooled proportion = 0.002263 (95% CI = 0.000939 to 0.004157)

Bias indicators

Begg-Mazumdar: Kendall's <too few strata> P = *

Egger: bias = * (95% CI = * to *) P = *

Harbord: bias = -1.363491 (92.5% CI = * to *) P = *

**Proportion meta-analysis Respiratory distress in the newborn**

Method: Stuart-Ord (inverse double arcsine square root)

| Stratum | Responding | Total |  |
| --- | --- | --- | --- |
| 1 | 75 | 3008 | Madhi 2020 |
| 2 | 1 | 139 | Moro 2014 |
| 3 | 2 | 341 | Perrett |
| 4 | 7 | 1019 | Mohamed |

| Stratum | Proportion | 95% CI (exact) | |  |
| --- | --- | --- | --- | --- |
| 1 | 0.024934 | 0.019661 | 0.031155 | Madhi 2020 |
| 2 | 0.007194 | 0.000182 | 0.039431 | Moro 2014 |
| 3 | 0.005865 | 0.000711 | 0.021025 | Perrett |
| 4 | 0.006869 | 0.002766 | 0.014102 | Mohamed |

| Stratum | Standardized effect | Variance | % Weights (fixed, random) | |  |
| --- | --- | --- | --- | --- | --- |
| 1 | 0.024934 | 0.000332 | 66.722111 | 30.892363 | Madhi 2020 |
| 2 | 0.007194 | 0.007168 | 3.093812 | 16.930837 | Moro 2014 |
| 3 | 0.005865 | 0.002928 | 7.573741 | 23.525672 | Perrett |
| 4 | 0.006869 | 0.000981 | 22.610335 | 28.651128 | Mohamed |

Fixed effects (inverse variance)

Pooled proportion = 0.018155 (95% CI = 0.014464 to 0.022256)

Non-combinability of studies

Cochran Q = 20.83267 (df = 3) P = 0.0001

Moment-based estimate of between studies variance = 0.007958

I_2_ (inconsistency) = 85.6% (95% CI = 54.5% to 92.6%)

Random effects (DerSimonian-Laird)

Pooled proportion = 0.012286 (95% CI = 0.003774 to 0.025591)

Bias indicators

Begg-Mazumdar: Kendall's 0.333333 P = 0.75

Egger: bias = -1.891565 (95% CI = -19.627181 to 15.844051) P = 0.6914

Harbord: bias = -3.327042 (92.5% CI = -10.389357 to 3.735273) P = 0.2463

**Proportion meta-analysis Failure to thrive**

Method: Stuart-Ord (inverse double arcsine square root)

| Stratum | Responding | Total |  |
| --- | --- | --- | --- |
| 1 | 6 | 3008 | Madhi 2020 |
| 2 | 2 | 22 | Munoz 2019 |
| 3 | 0 | 341 | Perrett |

| Stratum | Proportion | 95% CI (exact) | |  |
| --- | --- | --- | --- | --- |
| 1 | 0.001995 | 0.000732 | 0.004336 | Madhi 2020 |
| 2 | 0.090909 | 0.011206 | 0.291613 | Munoz 2019 |
| 3 | 0 | 0 | 0.01076 | Perrett [97.5% one-sided CI] |

| Stratum | Standardized effect | Variance | % Weights (fixed, random) | |  |
| --- | --- | --- | --- | --- | --- |
| 1 | 0.001995 | 0.000332 | 89.20682 | 50.976583 | Madhi 2020 |
| 2 | 0.090909 | 0.044444 | 0.667161 | 9.011592 | Munoz 2019 |
| 3 | 0 | 0.002928 | 10.126019 | 40.011826 | Perrett [97.5% one-sided CI] |

Fixed effects (inverse variance)

Pooled proportion = 0.002153 (95% CI = 0.000872 to 0.004)

Non-combinability of studies

Cochran Q = 7.97757 (df = 2) P = 0.0185

Moment-based estimate of between studies variance = 0.00914

I_2_ (inconsistency) = 74.9% (95% CI = 0% to 90.4%)

Random effects (DerSimonian-Laird)

Pooled proportion = 0.004173 (95% CI = 0.000012 to 0.017517)

Bias indicators

Begg-Mazumdar: Kendall's <too few strata> P = *

Egger: bias = * (95% CI = * to *) P = *

Harbord: bias = 6.330972 (92.5% CI = -38.379699 to 51.041643) P = 0.4432

**Proportion meta-analysis Microcephaly**

Method: Stuart-Ord (inverse double arcsine square root)

| Stratum | Responding | Total |  |
| --- | --- | --- | --- |
| 1 | 23 | 3008 | Madhi 2020 |

| Stratum | Proportion | 95% CI (exact) | |  |
| --- | --- | --- | --- | --- |
| 1 | 0.007646 | 0.004853 | 0.011451 | Madhi 2020 |

| Stratum | Standardized effect | Variance | % Weights (fixed, random) | |  |
| --- | --- | --- | --- | --- | --- |
| 1 | 0.007646 | 0.000332 | 100 | 100 | Madhi 2020 |

Fixed effects (inverse variance)

Pooled proportion = 0.007809 (95% CI = 0.004979 to 0.011268)

Non-combinability of studies

Cochran Q = 2.317664E-030 (df = 0) P = *

Moment-based estimate of between studies variance = 0

I_2_ (inconsistency) = *% (95% CI = *% to *%)

Random effects (DerSimonian-Laird)

Pooled proportion = 0.007809 (95% CI = 0.004979 to 0.011268)

Bias indicators

Begg-Mazumdar: Kendall's <too few strata> P = *

Egger: bias = * (95% CI = * to *) P = *

Harbord: bias = * (92.5% CI = * to *) P = *

**Proportion meta-analysis injection site reactions**

Method: Stuart-Ord (inverse double arcsine square root)

| Stratum | Responding | Total |  |
| --- | --- | --- | --- |
| 1 | 1240 | 3045 | Madhi 2020 |
| 2 | 1 | 22 | Munoz 2019 |
| 3 | 7 | 110 | Moro 2018 |
| 4 | 24 | 48 | Swamy |
| 5 | 289 | 335 | Perrett |
| 6 | 918 | 100000 | Angelo 2014 (Act. S, all) |
| 7 | 18 | 53885 | Kharbanda 2016 |
| 8 | 8 | 33 | Munoz 2014 |
| 9 | 64 | 361 | Fortner 2016 |
| 10 | 626 | 793 | Petousis-Harris 2016 |
| 11 | 89 | 1257 | Regan 2016 |
| 12 | 6 | 132 | Zheteyeva 2012 |

| Stratum | Proportion | 95% CI (exact) | |  |
| --- | --- | --- | --- | --- |
| 1 | 0.407225 | 0.389706 | 0.424922 | Madhi 2020 |
| 2 | 0.045455 | 0.00115 | 0.228444 | Munoz 2019 |
| 3 | 0.063636 | 0.025966 | 0.126726 | Moro 2018 |
| 4 | 0.5 | 0.352252 | 0.647748 | Swamy |
| 5 | 0.862687 | 0.821122 | 0.897685 | Perrett |
| 6 | 0.00918 | 0.008598 | 0.009791 | Angelo 2014 (Act. S, all) |
| 7 | 0.000334 | 0.000198 | 0.000528 | Kharbanda 2016 |
| 8 | 0.242424 | 0.110923 | 0.422589 | Munoz 2014 |
| 9 | 0.177285 | 0.139291 | 0.220669 | Fortner 2016 |
| 10 | 0.789407 | 0.759348 | 0.817297 | Petousis-Harris 2016 |
| 11 | 0.070804 | 0.057244 | 0.08641 | Regan 2016 |
| 12 | 0.045455 | 0.016861 | 0.096309 | Zheteyeva 2012 |

| Stratum | Standardized effect | Variance | % Weights (fixed, random) | |  |
| --- | --- | --- | --- | --- | --- |
| 1 | 0.407225 | 0.000328 | 1.903116 | 8.851873 | Madhi 2020 |
| 2 | 0.045455 | 0.044444 | 0.01406 | 6.692541 | Munoz 2019 |
| 3 | 0.063636 | 0.00905 | 0.069051 | 8.321111 | Moro 2018 |
| 4 | 0.5 | 0.020619 | 0.030307 | 7.708042 | Swamy |
| 5 | 0.862687 | 0.002981 | 0.209652 | 8.683435 | Perrett |
| 6 | 0.00918 | 0.00001 | 62.489767 | 8.872531 | Angelo 2014 (Act. S, all) |
| 7 | 0.000334 | 0.000019 | 33.672755 | 8.871974 | Kharbanda 2016 |
| 8 | 0.242424 | 0.029851 | 0.020934 | 7.280009 | Munoz 2014 |
| 9 | 0.177285 | 0.002766 | 0.225899 | 8.696811 | Fortner 2016 |
| 10 | 0.789407 | 0.00126 | 0.495854 | 8.791952 | Petousis-Harris 2016 |
| 11 | 0.070804 | 0.000795 | 0.785805 | 8.821751 | Regan 2016 |
| 12 | 0.045455 | 0.007547 | 0.082799 | 8.40797 | Zheteyeva 2012 |

Fixed effects (inverse variance)

Pooled proportion = 0.008288 (95% CI = 0.00785 to 0.008738)

Non-combinability of studies

Cochran Q = 10830.319574 (df = 11) P < 0.0001

Moment-based estimate of between studies variance = 0.136403

I_2_ (inconsistency) = 99.9% (95% CI = 99.9% to 99.9%)

Random effects (DerSimonian-Laird)

Pooled proportion = 0.220558 (95% CI = 0.138317 to 0.315741)

Bias indicators

Begg-Mazumdar: Kendall's -0.212121 P = 0.3108

Egger: bias = 18.679353 (95% CI = 4.461854 to 32.896852) P = 0.0151

Harbord: bias = 77.881288 (92.5% CI = 10.992634 to 144.769943) P = 0.0432

**Proportion meta-analysis Fever**

Method: Stuart-Ord (inverse double arcsine square root)

| Stratum | Responding | Total |  |
| --- | --- | --- | --- |
| 1 | 1255 | 3045 | Madhi 2020 |
| 2 | 0 | 22 | Munoz 2019 |
| 3 | 0 | 48 | Swamy |
| 4 | 4 | 335 | Perrett |
| 5 | 661 | 100000 | Angelo 2014 (Act. S, all) |
| 6 | 15 | 53885 | Kharbanda 2016 |
| 7 | 1 | 57 | Maertens 2016 |
| 8 | 1 | 52 | Hoang 2016 |
| 9 | 1 | 33 | Munoz 2014 |
| 10 | 0 | 361 | Fortner 2016 |
| 11 | 17 | 793 | Petousis-Harris 2016 |
| 12 | 24 | 1257 | Regan 2016 |

| Stratum | Proportion | 95% CI (exact) | |  |
| --- | --- | --- | --- | --- |
| 1 | 0.412151 | 0.394595 | 0.429876 | Madhi 2020 |
| 2 | 0 | 0 | 0.154373 | Munoz 2019 [97.5% one-sided CI] |
| 3 | 0 | 0 | 0.073973 | Swamy [97.5% one-sided CI] |
| 4 | 0.01194 | 0.003263 | 0.030288 | Perrett |
| 5 | 0.00661 | 0.006117 | 0.007132 | Angelo 2014 (Act. S, all) |
| 6 | 0.000278 | 0.000156 | 0.000459 | Kharbanda 2016 |
| 7 | 0.017544 | 0.000444 | 0.093917 | Maertens 2016 |
| 8 | 0.019231 | 0.000487 | 0.102554 | Hoang 2016 |
| 9 | 0.030303 | 0.000767 | 0.157594 | Munoz 2014 |
| 10 | 0 | 0 | 0.010166 | Fortner 2016 [97.5% one-sided CI] |
| 11 | 0.021438 | 0.012537 | 0.034102 | Petousis-Harris 2016 |
| 12 | 0.019093 | 0.012271 | 0.028276 | Regan 2016 |

| Stratum | Standardized effect | Variance | % Weights (fixed, random) | |  |
| --- | --- | --- | --- | --- | --- |
| 1 | 0.412151 | 0.000328 | 1.904699 | 9.433644 | Madhi 2020 |
| 2 | 0 | 0.044444 | 0.014072 | 5.778382 | Munoz 2019 [97.5% one-sided CI] |
| 3 | 0 | 0.020619 | 0.030333 | 7.307584 | Swamy [97.5% one-sided CI] |
| 4 | 0.01194 | 0.002981 | 0.209827 | 9.088021 | Perrett |
| 5 | 0.00661 | 0.00001 | 62.541746 | 9.476904 | Angelo 2014 (Act. S, all) |
| 6 | 0.000278 | 0.000019 | 33.700764 | 9.475736 | Kharbanda 2016 |
| 7 | 0.017544 | 0.017391 | 0.035961 | 7.579272 | Maertens 2016 |
| 8 | 0.019231 | 0.019048 | 0.032834 | 7.437358 | Hoang 2016 |
| 9 | 0.030303 | 0.029851 | 0.020951 | 6.627924 | Munoz 2014 |
| 10 | 0 | 0.002766 | 0.226087 | 9.115013 | Fortner 2016 [97.5% one-sided CI] |
| 11 | 0.021438 | 0.00126 | 0.496266 | 9.309252 | Petousis-Harris 2016 |
| 12 | 0.019093 | 0.000795 | 0.786459 | 9.37091 | Regan 2016 |

Fixed effects (inverse variance)

Pooled proportion = 0.005211 (95% CI = 0.004864 to 0.00557)

Non-combinability of studies

Cochran Q = 5.502.795701 (df = 11) P < 0.0001

Moment-based estimate of between studies variance = 0.069412

I_2_ (inconsistency) = 99.8% (95% CI = 99.8% to 99.8%)

Random effects (DerSimonian-Laird)

Pooled proportion = 0.026849 (95% CI = 0.007226 to 0.058404)

Bias indicators

Begg-Mazumdar: Kendall's -0.030303 P = 0.8406

Egger: bias = 7.085721 (95% CI = -3.16859 to 17.340032) P = 0.1547

Harbord: bias = 28.604079 (92.5% CI = -35.303372 to 92.51153) P = 0.3947

**Proportion meta-analysis headache**

Method: Stuart-Ord (inverse double arcsine square root)

| Stratum | Responding | Total |  |
| --- | --- | --- | --- |
| 1 | 4 | 22 | Munoz 2019 |
| 2 | 6 | 48 | Swamy |
| 3 | 83 | 335 | Perrett |
| 4 | 44322 | 100000 | Angelo 2014 (Act. S, all) |
| 5 | 11 | 33 | Munoz 2014 |
| 6 | 26 | 361 | Fortner 2016 |
| 7 | 31 | 793 | Petousis-Harris 2016 |
| 8 | 35 | 1257 | Regan 2016 |
| 9 | 3 | 132 | Zheteyeva 2012 |

| Stratum | Proportion | 95% CI (exact) | |  |
| --- | --- | --- | --- | --- |
| 1 | 0.181818 | 0.051867 | 0.402846 | Munoz 2019 |
| 2 | 0.125 | 0.047284 | 0.252461 | Swamy |
| 3 | 0.247761 | 0.202449 | 0.297595 | Perrett |
| 4 | 0.44322 | 0.440138 | 0.446306 | Angelo 2014 (Act. S, all) |
| 5 | 0.333333 | 0.179605 | 0.518267 | Munoz 2014 |
| 6 | 0.072022 | 0.047584 | 0.10375 | Fortner 2016 |
| 7 | 0.039092 | 0.026713 | 0.055031 | Petousis-Harris 2016 |
| 8 | 0.027844 | 0.019469 | 0.038513 | Regan 2016 |
| 9 | 0.022727 | 0.004712 | 0.064981 | Zheteyeva 2012 |

| Stratum | Standardized effect | Variance | % Weights (fixed, random) | |  |
| --- | --- | --- | --- | --- | --- |
| 1 | 0.181818 | 0.044444 | 0.021848 | 10.445229 | Munoz 2019 |
| 2 | 0.125 | 0.020619 | 0.047094 | 10.923438 | Swamy |
| 3 | 0.247761 | 0.002981 | 0.325774 | 11.306644 | Perrett |
| 4 | 0.44322 | 0.00001 | 97.101534 | 11.373845 | Angelo 2014 (Act. S, all) |
| 5 | 0.333333 | 0.029851 | 0.032529 | 10.733033 | Munoz 2014 |
| 6 | 0.072022 | 0.002766 | 0.35102 | 11.311467 | Fortner 2016 |
| 7 | 0.039092 | 0.00126 | 0.770497 | 11.345465 | Petousis-Harris 2016 |
| 8 | 0.027844 | 0.000795 | 1.221046 | 11.356004 | Regan 2016 |
| 9 | 0.022727 | 0.007547 | 0.128659 | 11.204874 | Zheteyeva 2012 |

Fixed effects (inverse variance)

Pooled proportion = 0.429176 (95% CI = 0.426154 to 0.4322)

Non-combinability of studies

Cochran Q = 2.936.526131 (df = 8) P < 0.0001

Moment-based estimate of between studies variance = 0.499796

I_2_ (inconsistency) = 99.7% (95% CI = 99.7% to 99.7%)

Random effects (DerSimonian-Laird)

Pooled proportion = 0.143198 (95% CI = 0.023659 to 0.339245)

Bias indicators

Begg-Mazumdar: Kendall's 0.444444 P = 0.1194

Egger: bias = -23.217521 (95% CI = -53.601372 to 7.16633) P = 0.1137

Harbord: bias = -11.653355 (92.5% CI = -19.962524 to -3.344185) P = 0.022

**Proportion meta-analysis gastrointestinal**

Method: Stuart-Ord (inverse double arcsine square root)

| Stratum | Responding | Total |  |
| --- | --- | --- | --- |
| 1 | 2 | 22 | Munoz 2019 |
| 2 | 60 | 335 | Perrett |

| Stratum | Proportion | 95% CI (exact) | |  |
| --- | --- | --- | --- | --- |
| 1 | 0.090909 | 0.011206 | 0.291613 | Munoz 2019 |
| 2 | 0.179104 | 0.139548 | 0.224442 | Perrett |

| Stratum | Standardized effect | Variance | % Weights (fixed, random) | |  |
| --- | --- | --- | --- | --- | --- |
| 1 | 0.090909 | 0.044444 | 6.284916 | 6.284916 | Munoz 2019 |
| 2 | 0.179104 | 0.002981 | 93.715084 | 93.715084 | Perrett |

Fixed effects (inverse variance)

Pooled proportion = 0.175071 (95% CI = 0.137517 to 0.216109)

Non-combinability of studies

Cochran Q = 0.908788 (df = 1) P = 0.3404

Moment-based estimate of between studies variance = 0

I_2_ (inconsistency) = 0% (95% CI = *% to *%)

Random effects (DerSimonian-Laird)

Pooled proportion = 0.175071 (95% CI = 0.137517 to 0.216109)

Bias indicators

Begg-Mazumdar: Kendall's <too few strata> P = *

Egger: bias = * (95% CI = * to *) P = *

Harbord: bias = -1.463603 (92.5% CI = * to *) P = *

**Proportion meta-analysis malaise**

Method: Stuart-Ord (inverse double arcsine square root)

| Stratum | Responding | Total |  |
| --- | --- | --- | --- |
| 1 | 3 | 53885 | Kharbanda 2016 |
| 2 | 4 | 33 | Munoz 2014 |
| 3 | 38 | 361 | Fortner 2016 |
| 4 | 5 | 1257 | Regan 2016 |

| Stratum | Proportion | 95% CI (exact) | |  |
| --- | --- | --- | --- | --- |
| 1 | 0.000056 | 0.000011 | 0.000163 | Kharbanda 2016 |
| 2 | 0.121212 | 0.034033 | 0.282016 | Munoz 2014 |
| 3 | 0.105263 | 0.075574 | 0.141612 | Fortner 2016 |
| 4 | 0.003978 | 0.001293 | 0.009258 | Regan 2016 |

| Stratum | Standardized effect | Variance | % Weights (fixed, random) | |  |
| --- | --- | --- | --- | --- | --- |
| 1 | 0.000056 | 0.000019 | 97.02456 | 27.838062 | Kharbanda 2016 |
| 2 | 0.121212 | 0.029851 | 0.060319 | 18.167398 | Munoz 2014 |
| 3 | 0.105263 | 0.002766 | 0.650906 | 26.536998 | Fortner 2016 |
| 4 | 0.003978 | 0.000795 | 2.264215 | 27.457542 | Regan 2016 |

Fixed effects (inverse variance)

Pooled proportion = 0.000136 (95% CI = 0.000057 to 0.000251)

Non-combinability of studies

Cochran Q = 183.678075 (df = 3) P < 0.0001

Moment-based estimate of between studies variance = 0.056024

I_2_ (inconsistency) = 98.4% (95% CI = 97.8% to 98.7%)

Random effects (DerSimonian-Laird)

Pooled proportion = 0.03069 (95% CI = 0.00288 to 0.086486)

Bias indicators

Begg-Mazumdar: Kendall's 0.333333 P = 0.75

Egger: bias = 3.395311 (95% CI = -2.813485 to 9.604106) P = 0.1429

Harbord: bias = 89.981362 (92.5% CI = -85.268975 to 265.231699) P = 0.2191

**Proportion meta-analysis myalgia**

Method: Stuart-Ord (inverse double arcsine square root)

| Stratum | Responding | Total |  |
| --- | --- | --- | --- |
| 1 | 1 | 22 | Munoz 2019 |
| 2 | 2 | 48 | Swamy |
| 3 | 5 | 33 | Munoz 2014 |
| 4 | 24 | 793 | Petousis-Harris 2016 |
| 5 | 10 | 1257 | Regan 2016 |

| Stratum | Proportion | 95% CI (exact) | |  |
| --- | --- | --- | --- | --- |
| 1 | 0.045455 | 0.00115 | 0.228444 | Munoz 2019 |
| 2 | 0.041667 | 0.005086 | 0.142541 | Swamy |
| 3 | 0.151515 | 0.051089 | 0.31899 | Munoz 2014 |
| 4 | 0.030265 | 0.019485 | 0.044698 | Petousis-Harris 2016 |
| 5 | 0.007955 | 0.003821 | 0.014582 | Regan 2016 |

| Stratum | Standardized effect | Variance | % Weights (fixed, random) | |  |
| --- | --- | --- | --- | --- | --- |
| 1 | 0.045455 | 0.044444 | 1.043841 | 10.310315 | Munoz 2019 |
| 2 | 0.041667 | 0.020619 | 2.250058 | 16.138351 | Swamy |
| 3 | 0.151515 | 0.029851 | 1.554164 | 13.23867 | Munoz 2014 |
| 4 | 0.030265 | 0.00126 | 36.812804 | 29.845528 | Petousis-Harris 2016 |
| 5 | 0.007955 | 0.000795 | 58.339132 | 30.467135 | Regan 2016 |

Fixed effects (inverse variance)

Pooled proportion = 0.017465 (95% CI = 0.012367 to 0.023424)

Non-combinability of studies

Cochran Q = 28.286078 (df = 4) P < 0.0001

Moment-based estimate of between studies variance = 0.021532

I_2_ (inconsistency) = 85.9% (95% CI = 64.1% to 92.2%)

Random effects (DerSimonian-Laird)

Pooled proportion = 0.038757 (95% CI = 0.013705 to 0.075836)

Bias indicators

Begg-Mazumdar: Kendall's 0.2 P = 0.8167

Egger: bias = 1.721684 (95% CI = -1.132191 to 4.575559) P = 0.1507

Harbord: bias = 3.620562 (92.5% CI = -1.942984 to 9.184109) P = 0.1794

**Proportion meta-analysis fatigue**

Method: Stuart-Ord (inverse double arcsine square root)

| Stratum | Responding | Total |  |
| --- | --- | --- | --- |
| 1 | 3 | 22 | Munoz 2019 |
| 2 | 18 | 48 | Swamy |
| 3 | 143 | 335 | Perrett |
| 4 | 1 | 52 | Hoang 2016 |
| 5 | 67 | 793 | Petousis-Harris 2016 |
| 6 | 41 | 1257 | Regan 2016 |

| Stratum | Proportion | 95% CI (exact) | |  |
| --- | --- | --- | --- | --- |
| 1 | 0.136364 | 0.029056 | 0.349122 | Munoz 2019 |
| 2 | 0.375 | 0.239522 | 0.526494 | Swamy |
| 3 | 0.426866 | 0.373261 | 0.481775 | Perrett |
| 4 | 0.019231 | 0.000487 | 0.102554 | Hoang 2016 |
| 5 | 0.084489 | 0.066076 | 0.106058 | Petousis-Harris 2016 |
| 6 | 0.032617 | 0.023506 | 0.043991 | Regan 2016 |

| Stratum | Standardized effect | Variance | % Weights (fixed, random) | |  |
| --- | --- | --- | --- | --- | --- |
| 1 | 0.136364 | 0.044444 | 0.896414 | 14.609851 | Munoz 2019 |
| 2 | 0.375 | 0.020619 | 1.932271 | 16.161121 | Swamy |
| 3 | 0.426866 | 0.002981 | 13.366534 | 17.539806 | Perrett |
| 4 | 0.019231 | 0.019048 | 2.091633 | 16.275061 | Hoang 2016 |
| 5 | 0.084489 | 0.00126 | 31.613546 | 17.686978 | Petousis-Harris 2016 |
| 6 | 0.032617 | 0.000795 | 50.099602 | 17.727183 | Regan 2016 |

Fixed effects (inverse variance)

Pooled proportion = 0.087382 (95% CI = 0.076653 to 0.098743)

Non-combinability of studies

Cochran Q = 327.382293 (df = 5) P < 0.0001

Moment-based estimate of between studies variance = 0.203773

I_2_ (inconsistency) = 98.5% (95% CI = 98.1% to 98.8%)

Random effects (DerSimonian-Laird)

Pooled proportion = 0.153155 (95% CI = 0.045717 to 0.308353)

Bias indicators

Begg-Mazumdar: Kendall's 0.333333 P = 0.4694

Egger: bias = 5.576724 (95% CI = -3.976434 to 15.129882) P = 0.1804

Harbord: bias = 6.812598 (92.5% CI = -11.943156 to 25.568353) P = 0.4339

**Proportion meta-analysis Vomiting**

Method: Stuart-Ord (inverse double arcsine square root)

| Stratum | Responding | Total |  |
| --- | --- | --- | --- |
| 1 | 1 | 22 | Munoz 2019 |
| 2 | 22 | 793 | Petousis-Harris 2016 |
| 3 | 10 | 1257 | Regan 2016 |

| Stratum | Proportion | 95% CI (exact) | |  |
| --- | --- | --- | --- | --- |
| 1 | 0.045455 | 0.00115 | 0.228444 | Munoz 2019 |
| 2 | 0.027743 | 0.017466 | 0.041703 | Petousis-Harris 2016 |
| 3 | 0.007955 | 0.003821 | 0.014582 | Regan 2016 |

| Stratum | Standardized effect | Variance | % Weights (fixed, random) | |  |
| --- | --- | --- | --- | --- | --- |
| 1 | 0.045455 | 0.044444 | 1.085122 | 9.919667 | Munoz 2019 |
| 2 | 0.027743 | 0.00126 | 38.268628 | 44.186596 | Petousis-Harris 2016 |
| 3 | 0.007955 | 0.000795 | 60.64625 | 45.893737 | Regan 2016 |

Fixed effects (inverse variance)

Pooled proportion = 0.015042 (95% CI = 0.010254 to 0.020729)

Non-combinability of studies

Cochran Q = 13.319143 (df = 2) P = 0.0013

Moment-based estimate of between studies variance = 0.011241

I_2_ (inconsistency) = 85% (95% CI = 23.9% to 93.2%)

Random effects (DerSimonian-Laird)

Pooled proportion = 0.020022 (95% CI = 0.004773 to 0.045437)

Bias indicators

Begg-Mazumdar: Kendall's <too few strata> P = *

Egger: bias = * (95% CI = * to *) P = *

Harbord: bias = 2.071001 (92.5% CI = -30.24788 to 34.389883) P = 0.6841

**Proportion meta-analysis Nausea**

Method: Stuart-Ord (inverse double arcsine square root)

| Stratum | Responding | Total |  |
| --- | --- | --- | --- |
| 1 | 0 | 22 | Munoz 2019 |
| 2 | 7 | 48 | Swamy |
| 3 | 22 | 793 | Petousis-Harris 2016 |

| Stratum | Proportion | 95% CI (exact) | |  |
| --- | --- | --- | --- | --- |
| 1 | 0 | 0 | 0.154373 | Munoz 2019 [97.5% one-sided CI] |
| 2 | 0.145833 | 0.060704 | 0.277638 | Swamy |
| 3 | 0.027743 | 0.017466 | 0.041703 | Petousis-Harris 2016 |

| Stratum | Standardized effect | Variance | % Weights (fixed, random) | |  |
| --- | --- | --- | --- | --- | --- |
| 1 | 0 | 0.044444 | 2.60266 | 25.305893 | Munoz 2019 [97.5% one-sided CI] |
| 2 | 0.145833 | 0.020619 | 5.610179 | 32.483397 | Swamy |
| 3 | 0.027743 | 0.00126 | 91.78716 | 42.21071 | Petousis-Harris 2016 |

Fixed effects (inverse variance)

Pooled proportion = 0.032234 (95% CI = 0.021508 to 0.045037)

Non-combinability of studies

Cochran Q = 10.421472 (df = 2) P = 0.0055

Moment-based estimate of between studies variance = 0.063385

I_2_ (inconsistency) = 80.8% (95% CI = 0% to 92%)

Random effects (DerSimonian-Laird)

Pooled proportion = 0.051301 (95% CI = 0.004429 to 0.144798)

Bias indicators

Begg-Mazumdar: Kendall's <too few strata> P = *

Egger: bias = * (95% CI = * to *) P = *

Harbord: bias = 2.208012 (92.5% CI = -28.455487 to 32.87151) P = 0.652

**Proportion meta-analysis Chills**

Method: Stuart-Ord (inverse double arcsine square root)

| Stratum | Responding | Total |  |
| --- | --- | --- | --- |
| 1 | 0 | 22 | Munoz 2019 |
| 2 | 1 | 48 | Swamy |

| Stratum | Proportion | 95% CI (exact) | |  |
| --- | --- | --- | --- | --- |
| 1 | 0 | 0 | 0.154373 | Munoz 2019 [97.5% one-sided CI] |
| 2 | 0.020833 | 0.000527 | 0.110696 | Swamy |

| Stratum | Standardized effect | Variance | % Weights (fixed, random) | |  |
| --- | --- | --- | --- | --- | --- |
| 1 | 0 | 0.044444 | 31.690141 | 31.690141 | Munoz 2019 [97.5% one-sided CI] |
| 2 | 0.020833 | 0.020619 | 68.309859 | 68.309859 | Swamy |

Fixed effects (inverse variance)

Pooled proportion = 0.022844 (95% CI = 0.001254 to 0.070135)

Non-combinability of studies

Cochran Q = 0.287307 (df = 1) P = 0.592

Moment-based estimate of between studies variance = 0

I_2_ (inconsistency) = 0% (95% CI = *% to *%)

Random effects (DerSimonian-Laird)

Pooled proportion = 0.022844 (95% CI = 0.001254 to 0.070135)

Bias indicators

Begg-Mazumdar: Kendall's <too few strata> P = *

Egger: bias = * (95% CI = * to *) P = *

Harbord: bias = -2.024881 (92.5% CI = * to *) P = *

**Proportion meta-analysis Joint Pain**

Method: Stuart-Ord (inverse double arcsine square root)

| Stratum | Responding | Total |  |
| --- | --- | --- | --- |
| 1 | 0 | 22 | Munoz 2019 |
| 2 | 2 | 48 | Swamy |
| 3 | 24 | 793 | Petousis-Harris 2016 |

| Stratum | Proportion | 95% CI (exact) | |  |
| --- | --- | --- | --- | --- |
| 1 | 0 | 0 | 0.154373 | Munoz 2019 [97.5% one-sided CI] |
| 2 | 0.041667 | 0.005086 | 0.142541 | Swamy |
| 3 | 0.030265 | 0.019485 | 0.044698 | Petousis-Harris 2016 |

| Stratum | Standardized effect | Variance | % Weights (fixed, random) | |  |
| --- | --- | --- | --- | --- | --- |
| 1 | 0 | 0.044444 | 2.60266 | 2.60266 | Munoz 2019 [97.5% one-sided CI] |
| 2 | 0.041667 | 0.020619 | 5.610179 | 5.610179 | Swamy |
| 3 | 0.030265 | 0.00126 | 91.78716 | 91.78716 | Petousis-Harris 2016 |

Fixed effects (inverse variance)

Pooled proportion = 0.031184 (95% CI = 0.020647 to 0.043803)

Non-combinability of studies

Cochran Q = 0.945671 (df = 2) P = 0.6232

Moment-based estimate of between studies variance = 0

I_2_ (inconsistency) = 0% (95% CI = 0% to 72.9%)

Random effects (DerSimonian-Laird)

Pooled proportion = 0.031184 (95% CI = 0.020647 to 0.043803)

Bias indicators

Begg-Mazumdar: Kendall's <too few strata> P = *

Egger: bias = * (95% CI = * to *) P = *

Harbord: bias = -0.283592 (92.5% CI = -7.189143 to 6.62196) P = 0.7874

**Proportion meta-analysis Systemic reactions**

Method: Stuart-Ord (inverse double arcsine square root)

| Stratum | Responding | Total |  |
| --- | --- | --- | --- |
| 1 | 6 | 22 | Munoz 2019 |
| 2 | 12 | 110 | Moro 2018 |

| Stratum | Proportion | 95% CI (exact) | |  |
| --- | --- | --- | --- | --- |
| 1 | 0.272727 | 0.107289 | 0.502221 | Munoz 2019 |
| 2 | 0.109091 | 0.057655 | 0.182807 | Moro 2018 |

| Stratum | Standardized effect | Variance | % Weights (fixed, random) | |  |
| --- | --- | --- | --- | --- | --- |
| 1 | 0.272727 | 0.044444 | 16.917293 | 40.695538 | Munoz 2019 |
| 2 | 0.109091 | 0.00905 | 83.082707 | 59.304462 | Moro 2018 |

Fixed effects (inverse variance)

Pooled proportion = 0.136926 (95% CI = 0.084014 to 0.200299)

Non-combinability of studies

Cochran Q = 3.555574 (df = 1) P = 0.0593

Moment-based estimate of between studies variance = 0.068354

I_2_ (inconsistency) = 71.9% (95% CI = *% to *%)

Random effects (DerSimonian-Laird)

Pooled proportion = 0.174462 (95% CI = 0.048023 to 0.357468)

Bias indicators

Begg-Mazumdar: Kendall's <too few strata> P = *

Egger: bias = * (95% CI = * to *) P = *

Harbord: bias = 4.038937 (92.5% CI = * to *) P = *

**Proportion meta-analysis Any systemic reactions**

Method: Stuart-Ord (inverse double arcsine square root)

| Stratum | Responding | Total |  |
| --- | --- | --- | --- |
| 1 | 1255 | 3045 | Madhi 2020 |

| Stratum | Proportion | 95% CI (exact) | |  |
| --- | --- | --- | --- | --- |
| 1 | 0.412151 | 0.394595 | 0.429876 | Madhi 2020 |

| Stratum | Standardized effect | Variance | % Weights (fixed, random) | |  |
| --- | --- | --- | --- | --- | --- |
| 1 | 0.412151 | 0.000328 | 100 | 100 | Madhi 2020 |

Fixed effects (inverse variance)

Pooled proportion = 0.41218 (95% CI = 0.394757 to 0.429713)

Non-combinability of studies

Cochran Q = 1.501547E-028 (df = 0) P = *

Moment-based estimate of between studies variance = 0

I_2_ (inconsistency) = *% (95% CI = *% to *%)

Random effects (DerSimonian-Laird)

Pooled proportion = 0.41218 (95% CI = 0.394757 to 0.429713)

Bias indicators

Begg-Mazumdar: Kendall's <too few strata> P = *

Egger: bias = * (95% CI = * to *) P = *

Harbord: bias = * (92.5% CI = * to *) P = *

**Proportion meta-analysis MAEs**

Method: Stuart-Ord (inverse double arcsine square root)

| Stratum | Responding | Total |  |
| --- | --- | --- | --- |
| 1 | 40 | 49 | Swamy |
| 2 | 20 | 1257 | Regan 2016 |

| Stratum | Proportion | 95% CI (exact) | |  |
| --- | --- | --- | --- | --- |
| 1 | 0.816327 | 0.679779 | 0.91241 | Swamy |
| 2 | 0.015911 | 0.009745 | 0.024467 | Regan 2016 |

| Stratum | Standardized effect | Variance | % Weights (fixed, random) | |  |
| --- | --- | --- | --- | --- | --- |
| 1 | 0.816327 | 0.020202 | 3.787299 | 49.753439 | Swamy |
| 2 | 0.015911 | 0.000795 | 96.212701 | 50.246561 | Regan 2016 |

Fixed effects (inverse variance)

Pooled proportion = 0.027161 (95% CI = 0.019047 to 0.036664)

Non-combinability of studies

Cochran Q = 187.428854 (df = 1) P < 0.0001

Moment-based estimate of between studies variance = 1.957247

I_2_ (inconsistency) = 99.5% (95% CI = *% to *%)

Random effects (DerSimonian-Laird)

Pooled proportion = 0.339022 (95% CI = 0.117924 to 0.999483)

Bias indicators

Begg-Mazumdar: Kendall's <too few strata> P = *

Egger: bias = * (95% CI = * to *) P = *

Harbord: bias = 42.947966 (92.5% CI = * to *) P = *

**Proportion meta-analysis SAEs**

Method: Stuart-Ord (inverse double arcsine square root)

| Stratum | Responding | Total |  |
| --- | --- | --- | --- |
| 1 | 906 | 3045 | Madhi 2020 |
| 2 | 8 | 22 | Munoz 2019 |
| 3 | 12 | 110 | Moro 2018 |
| 4 | 0 | 49 | Swamy |
| 5 | 1 | 341 | Perrett |
| 6 | 11 | 57 | Maertens 2016 |

| Stratum | Proportion | 95% CI (exact) | |  |
| --- | --- | --- | --- | --- |
| 1 | 0.297537 | 0.281335 | 0.314128 | Madhi 2020 |
| 2 | 0.363636 | 0.171979 | 0.593423 | Munoz 2019 |
| 3 | 0.109091 | 0.057655 | 0.182807 | Moro 2018 |
| 4 | 0 | 0 | 0.072519 | Swamy [97.5% one-sided CI] |
| 5 | 0.002933 | 0.000074 | 0.01623 | Perrett |
| 6 | 0.192982 | 0.100472 | 0.31911 | Maertens 2016 |

| Stratum | Standardized effect | Variance | % Weights (fixed, random) | |  |
| --- | --- | --- | --- | --- | --- |
| 1 | 0.297537 | 0.000328 | 83.967466 | 17.351443 | Madhi 2020 |
| 2 | 0.363636 | 0.044444 | 0.620347 | 15.463129 | Munoz 2019 |
| 3 | 0.109091 | 0.00905 | 3.046595 | 16.942424 | Moro 2018 |
| 4 | 0 | 0.020202 | 1.364764 | 16.446677 | Swamy [97.5% one-sided CI] |
| 5 | 0.002933 | 0.002928 | 9.415495 | 17.227461 | Perrett |
| 6 | 0.192982 | 0.017391 | 1.585332 | 16.568866 | Maertens 2016 |

Fixed effects (inverse variance)

Pooled proportion = 0.241238 (95% CI = 0.227454 to 0.255296)

Non-combinability of studies

Cochran Q = 377.670802 (df = 5) P < 0.0001

Moment-based estimate of between studies variance = 0.360932

I_2_ (inconsistency) = 98.7% (95% CI = 98.4% to 98.9%)

Random effects (DerSimonian-Laird)

Pooled proportion = 0.123098 (95% CI = 0.012743 to 0.322404)

Bias indicators

Begg-Mazumdar: Kendall's 0.466667 P = 0.2722

Egger: bias = 5.88772 (95% CI = -18.030271 to 29.805711) P = 0.5319

Harbord: bias = -4.579001 (92.5% CI = -11.985985 to 2.827983) P = 0.2133

## **5 VACCINES CONTAINING ALUMINUM IN ANIMALS & HUMANS + ANIMALS**

**Proportion meta-analysis Abortion Both (humans and animals)**

Method: Stuart-Ord (inverse double arcsine square root)

| Stratum | Responding | Total |  |
| --- | --- | --- | --- |
| 1 | 3 | 40 | Kushner 2020 |
| 2 | 43 | 378 | Celzo 2020 (H) |
| 3 | 57 | 339 | Celzo 2020 (E) |
| 4 | 26 | 194 | Celzo 2020 (T) |
| 5 | 23 | 110 | Moro 2018 |
| 6 | 0 | 8 | Glenn 2015 |
| 7 | 15 | 139 | Moro 2014 |
| 8 | 24 | 207 | Baril 2015 |
| 9 | 53 | 593 | Dana 2009 |
| 10 | 16 | 137 | Angelo 2014 |
| 11 | 5 | 9 | Angelo 2014 (Pass. S) |
| 12 | 10 | 160 | Angelo 2014 (Act. S, all) |
| 13 | 4 | 138 | Shakib 2013 |
| 14 | 22 | 132 | Zheteyeva 2012 |

| Stratum | Proportion | 95% CI (exact) | |  |
| --- | --- | --- | --- | --- |
| 1 | 0.075 | 0.015742 | 0.203865 | Kushner 2020 |
| 2 | 0.113757 | 0.08356 | 0.15016 | Celzo 2020 (H) |
| 3 | 0.168142 | 0.129907 | 0.21229 | Celzo 2020 (E) |
| 4 | 0.134021 | 0.08945 | 0.190175 | Celzo 2020 (T) |
| 5 | 0.209091 | 0.137403 | 0.297035 | Moro 2018 |
| 6 | 0 | 0 | 0.369417 | Glenn 2015 [97.5% one-sided CI] |
| 7 | 0.107914 | 0.06167 | 0.171736 | Moro 2014 |
| 8 | 0.115942 | 0.075716 | 0.167587 | Baril 2015 |
| 9 | 0.089376 | 0.067665 | 0.115276 | Dana 2009 |
| 10 | 0.116788 | 0.068249 | 0.182721 | Angelo 2014 |
| 11 | 0.555556 | 0.212009 | 0.863004 | Angelo 2014 (Pass. S) |
| 12 | 0.0625 | 0.030375 | 0.111935 | Angelo 2014 (Act. S, all) |
| 13 | 0.028986 | 0.007953 | 0.07255 | Shakib 2013 |
| 14 | 0.166667 | 0.107496 | 0.241391 | Zheteyeva 2012 |

| Stratum | Standardized effect | Variance | % Weights (fixed, random) | |  |
| --- | --- | --- | --- | --- | --- |
| 1 | 0.075 | 0.024691 | 1.563103 | 4.622916 | Kushner 2020 |
| 2 | 0.113757 | 0.002642 | 14.608259 | 9.493066 | Celzo 2020 (H) |
| 3 | 0.168142 | 0.002946 | 13.103049 | 9.357378 | Celzo 2020 (E) |
| 4 | 0.134021 | 0.005141 | 7.506754 | 8.480373 | Celzo 2020 (T) |
| 5 | 0.209091 | 0.00905 | 4.264763 | 7.267964 | Moro 2018 |
| 6 | 0 | 0.117647 | 0.328059 | 1.461653 | Glenn 2015 [97.5% one-sided CI] |
| 7 | 0.107914 | 0.007168 | 5.384022 | 7.80509 | Moro 2014 |
| 8 | 0.115942 | 0.004819 | 8.008491 | 8.598588 | Baril 2015 |
| 9 | 0.089376 | 0.001685 | 22.906214 | 9.947967 | Dana 2009 |
| 10 | 0.116788 | 0.007273 | 5.306831 | 7.773251 | Angelo 2014 |
| 11 | 0.555556 | 0.105263 | 0.366654 | 1.608159 | Angelo 2014 (Pass. S) |
| 12 | 0.0625 | 0.006231 | 6.194519 | 8.103665 | Angelo 2014 (Act. S, all) |
| 13 | 0.028986 | 0.00722 | 5.345426 | 7.789253 | Shakib 2013 |
| 14 | 0.166667 | 0.007547 | 5.113856 | 7.690677 | Zheteyeva 2012 |

Fixed effects (inverse variance)

Pooled proportion = 0.114499 (95% CI = 0.102527 to 0.127042)

Non-combinability of studies

Cochran Q = 54.691493 (df = 13) P < 0.0001

Moment-based estimate of between studies variance = 0.018288

I_2_ (inconsistency) = 76.2% (95% CI = 57.1% to 84.6%)

Random effects (DerSimonian-Laird)

Pooled proportion = 0.118081 (95% CI = 0.09138 to 0.147694)

Bias indicators

Begg-Mazumdar: Kendall's 0.230769 P = 0.2792

Egger: bias = 1.899061 (95% CI = -0.596047 to 4.39417) P = 0.1231

Harbord: bias = 1.189813 (92.5% CI = -1.506318 to 3.885945) P = 0.4065

**Proportion meta-analysis Abortion in Animal**

Method: Stuart-Ord (inverse double arcsine square root)

| Stratum | Responding | Total |  |
| --- | --- | --- | --- |
| 1 | 0 | 8 | Glenn 2015 |

| Stratum | Proportion | 95% CI (exact) | |  |
| --- | --- | --- | --- | --- |
| 1 | 0 | 0 | 0.369417 | Glenn 2015 [97.5% one-sided CI] |

| Stratum | Standardized effect | Variance | % Weights (fixed, random) | |  |
| --- | --- | --- | --- | --- | --- |
| 1 | 0 | 0.117647 | 0.328059 | 1.461653 | Glenn 2015 [97.5% one-sided CI] |

I_2_ (inconsistency) = *% (95% CI = *% to *%)

Random effects (DerSimonian-Laird)

Pooled proportion = 0 (95% CI = 0 to 0.369417)

**Proportion meta-analysis Stillbirth Both (humans and animals)**

Method: Stuart-Ord (inverse double arcsine square root)

| Stratum | Responding | Total |  |
| --- | --- | --- | --- |
| 1 | 15 | 3045 | Madhi 2020 |
| 2 | 1 | 40 | Kushner 2020 |
| 3 | 2 | 378 | Celzo 2020 (H) |
| 4 | 3 | 339 | Celzo 2020 (E) |
| 5 | 2 | 194 | Celzo 2020 (T) |
| 6 | 2 | 110 | Moro 2018 |
| 7 | 0 | 28 | Glenn 2015 |
| 8 | 1 | 139 | Moro 2014 |
| 9 | 0 | 49 | Swamy |
| 10 | 3 | 330 | Baril 2015 |
| 11 | 0 | 341 | Perrett |
| 12 | 0 | 160 | Angelo 2014 (Act. S, all) |
| 13 | 0 | 52 | Hoang 2016 |
| 14 | 12 | 6185 | Donegan 2014 |
| 15 | 0 | 134 | Shakib 2013 |
| 16 | 0 | 650 | Berenson 2016 |
| 17 | 25 | 7152 | Morgan 2015 |
| 18 | 1 | 793 | Petousis-Harris 2016 |
| 19 | 2 | 132 | Zheteyeva 2012 |

| Stratum | Proportion | 95% CI (exact) | |  |
| --- | --- | --- | --- | --- |
| 1 | 0.004926 | 0.00276 | 0.008112 | Madhi 2020 |
| 2 | 0.025 | 0.000633 | 0.131586 | Kushner 2020 |
| 3 | 0.005291 | 0.000641 | 0.018981 | Celzo 2020 (H) |
| 4 | 0.00885 | 0.001829 | 0.025643 | Celzo 2020 (E) |
| 5 | 0.010309 | 0.001251 | 0.036743 | Celzo 2020 (T) |
| 6 | 0.018182 | 0.00221 | 0.064138 | Moro 2018 |
| 7 | 0 | 0 | 0.123436 | Glenn 2015 [97.5% one-sided CI] |
| 8 | 0.007194 | 0.000182 | 0.039431 | Moro 2014 |
| 9 | 0 | 0 | 0.072519 | Swamy [97.5% one-sided CI] |
| 10 | 0.009091 | 0.001879 | 0.026336 | Baril 2015 |
| 11 | 0 | 0 | 0.01076 | Perrett [97.5% one-sided CI] |
| 12 | 0 | 0 | 0.022792 | Angelo 2014 (Act. S, all) [97.5% one-sided CI] |
| 13 | 0 | 0 | 0.068482 | Hoang 2016 [97.5% one-sided CI] |
| 14 | 0.00194 | 0.001003 | 0.003387 | Donegan 2014 |
| 15 | 0 | 0 | 0.027153 | Shakib 2013 [97.5% one-sided CI] |
| 16 | 0 | 0 | 0.005659 | Berenson 2016 [97.5% one-sided CI] |
| 17 | 0.003496 | 0.002263 | 0.005156 | Morgan 2015 |
| 18 | 0.001261 | 0.000032 | 0.007006 | Petousis-Harris 2016 |
| 19 | 0.015152 | 0.00184 | 0.05366 | Zheteyeva 2012 |

| Stratum | Standardized effect | Variance | % Weights (fixed, random) | |  |
| --- | --- | --- | --- | --- | --- |
| 1 | 0.004926 | 0.000328 | 15.031712 | 14.498919 | Madhi 2020 |
| 2 | 0.025 | 0.024691 | 0.199896 | 0.709527 | Kushner 2020 |
| 3 | 0.005291 | 0.002642 | 1.868167 | 5.095159 | Celzo 2020 (H) |
| 4 | 0.00885 | 0.002946 | 1.675674 | 4.695654 | Celzo 2020 (E) |
| 5 | 0.010309 | 0.005141 | 0.959996 | 2.996011 | Celzo 2020 (T) |
| 6 | 0.018182 | 0.00905 | 0.545396 | 1.82212 | Moro 2018 |
| 7 | 0 | 0.035088 | 0.140668 | 0.504698 | Glenn 2015 [97.5% one-sided CI] |
| 8 | 0.007194 | 0.007168 | 0.688532 | 2.245658 | Moro 2014 |
| 9 | 0 | 0.020202 | 0.244318 | 0.860295 | Swamy [97.5% one-sided CI] |
| 10 | 0.009091 | 0.003026 | 1.631253 | 4.600325 | Baril 2015 |
| 11 | 0 | 0.002928 | 1.685546 | 4.716674 | Perrett [97.5% one-sided CI] |
| 12 | 0 | 0.006231 | 0.792182 | 2.540004 | Angelo 2014 (Act. S, all) [97.5% one-sided CI] |
| 13 | 0 | 0.019048 | 0.259125 | 0.910019 | Hoang 2016 [97.5% one-sided CI] |
| 14 | 0.00194 | 0.000162 | 30.529849 | 16.72244 | Donegan 2014 |
| 15 | 0 | 0.007435 | 0.663853 | 2.174076 | Shakib 2013 [97.5% one-sided CI] |
| 16 | 0 | 0.001537 | 3.210681 | 7.380941 | Berenson 2016 [97.5% one-sided CI] |
| 17 | 0.003496 | 0.00014 | 35.302683 | 17.065623 | Morgan 2015 |
| 18 | 0.001261 | 0.00126 | 3.916488 | 8.316579 | Petousis-Harris 2016 |
| 19 | 0.015152 | 0.007547 | 0.653982 | 2.145278 | Zheteyeva 2012 |

Fixed effects (inverse variance)

Pooled proportion = 0.003378 (95% CI = 0.002627 to 0.004224)

Non-combinability of studies

Cochran Q = 32.162147 (df = 18) P = 0.021

Moment-based estimate of between studies variance = 0.000925

I_2_ (inconsistency) = 44% (95% CI = 0% to 66.3%)

Random effects (DerSimonian-Laird)

Pooled proportion = 0.004128 (95% CI = 0.002607 to 0.005996)

Bias indicators

Begg-Mazumdar: Kendall's 0.134503 P = 0.4467

Egger: bias = 0.344513 (95% CI = -0.234643 to 0.923669) P = 0.2264

Harbord: bias = 0.789713 (92.5% CI = -0.094757 to 1.674183) P = 0.1086

**Proportion meta-analysis Stillbirth Animal**

Method: Stuart-Ord (inverse double arcsine square root)

| Stratum | Responding | Total |  |
| --- | --- | --- | --- |
| 1 | 0 | 28 | Glenn 2015 |

| Stratum | Proportion | 95% CI (exact) | |  |
| --- | --- | --- | --- | --- |
| 1 | 0 | 0 | 0.123436 | Glenn 2015 [97.5% one-sided CI] |

| Stratum | Standardized effect | Variance | % Weights (fixed, random) | |  |
| --- | --- | --- | --- | --- | --- |
| 1 | 0 | 0.035088 | 0.140668 | 0.504698 | Glenn 2015 [97.5% one-sided CI] |

I_2_ (inconsistency) = *% (95% CI = *% to *%)

Random effects (DerSimonian-Laird)

Pooled proportion = 0 (95% CI = 0 to 0.123436)

**Proportion meta-analysis any congenital malformation ANIMALS**

Method: Stuart-Ord (inverse double arcsine square root)

| Stratum | Responding | Total |  |
| --- | --- | --- | --- |
| 1 | 1 | 164 | Segal 2011 |
| 2 | 41 | 326 | Wise |

| Stratum | Proportion | 95% CI (exact) | |  |
| --- | --- | --- | --- | --- |
| 1 | 0.006098 | 0.000154 | 0.033504 | Segal 2011 |
| 2 | 0.125767 | 0.091785 | 0.166728 | Wise |

| Stratum | Standardized effect | Variance | % Weights (fixed, random) | |  |
| --- | --- | --- | --- | --- | --- |
| 1 | 0.006098 | 0.006079 | 33.503055 | 49.48326 | Segal 2011 |
| 2 | 0.125767 | 0.003063 | 66.496945 | 50.51674 | Wise |

Fixed effects (inverse variance)

Pooled proportion = 0.073083 (95% CI = 0.05176 to 0.097743)

Non-combinability of studies

Cochran Q = 31.925056 (df = 1) P < 0.0001

Moment-based estimate of between studies variance = 0.141356

I_2_ (inconsistency) = 96.9% (95% CI = *% to *%)

Random effects (DerSimonian-Laird)

Pooled proportion = 0.052231 (95% CI = 0.001164 to 0.225885)

Bias indicators

Begg-Mazumdar: Kendall's <too few strata> P = *

Egger: bias = * (95% CI = * to *) P = *

Harbord: bias = -20.253083 (92.5% CI = * to *) P = *

**Proportion meta-analysis any congenital malformation Both (humans and animals)**

Method: Stuart-Ord (inverse double arcsine square root)

| Stratum | Responding | Total |  |
| --- | --- | --- | --- |
| 1 | 6 | 3008 | Madhi 2020 |
| 2 | 1 | 22 | Munoz 2019 |
| 3 | 1 | 40 | Kushner 2020 |
| 4 | 19 | 378 | Celzo 2020 (H) |
| 5 | 29 | 339 | Celzo 2020 (E) |
| 6 | 10 | 194 | Celzo 2020 (T) |
| 7 | 5 | 110 | Moro 2018 |
| 8 | 2 | 139 | Moro 2014 |
| 9 | 7 | 48 | Swamy |
| 10 | 9 | 210 | Baril 2015 |
| 11 | 1 | 164 | Segal 2011 |
| 12 | 41 | 326 | Wise |
| 13 | 9 | 341 | Perrett |
| 14 | 20 | 593 | Dana 2009 |
| 15 | 13 | 160 | Angelo 2014 (Act. S, all) |
| 16 | 5 | 134 | Shakib 2013 |
| 17 | 15 | 650 | Berenson 2016 |
| 18 | 84 | 7152 | Morgan 2015 |

| Stratum | Proportion | 95% CI (exact) | |  |
| --- | --- | --- | --- | --- |
| 1 | 0.001995 | 0.000732 | 0.004336 | Madhi 2020 |
| 2 | 0.045455 | 0.00115 | 0.228444 | Munoz 2019 |
| 3 | 0.025 | 0.000633 | 0.131586 | Kushner 2020 |
| 4 | 0.050265 | 0.03053 | 0.077384 | Celzo 2020 (H) |
| 5 | 0.085546 | 0.058039 | 0.120547 | Celzo 2020 (E) |
| 6 | 0.051546 | 0.024992 | 0.092751 | Celzo 2020 (T) |
| 7 | 0.045455 | 0.014921 | 0.102892 | Moro 2018 |
| 8 | 0.014388 | 0.001747 | 0.051008 | Moro 2014 |
| 9 | 0.145833 | 0.060704 | 0.277638 | Swamy |
| 10 | 0.042857 | 0.019781 | 0.079795 | Baril 2015 |
| 11 | 0.006098 | 0.000154 | 0.033504 | Segal 2011 |
| 12 | 0.125767 | 0.091785 | 0.166728 | Wise |
| 13 | 0.026393 | 0.012138 | 0.049509 | Perrett |
| 14 | 0.033727 | 0.020721 | 0.051609 | Dana 2009 |
| 15 | 0.08125 | 0.043972 | 0.134932 | Angelo 2014 (Act. S, all) |
| 16 | 0.037313 | 0.012225 | 0.084928 | Shakib 2013 |
| 17 | 0.023077 | 0.012972 | 0.037777 | Berenson 2016 |
| 18 | 0.011745 | 0.009379 | 0.014521 | Morgan 2015 |

| Stratum | Standardized effect | Variance | % Weights (fixed, random) | |  |
| --- | --- | --- | --- | --- | --- |
| 1 | 0.001995 | 0.000332 | 21.463223 | 6.731177 | Madhi 2020 |
| 2 | 0.045455 | 0.044444 | 0.160519 | 2.63771 | Munoz 2019 |
| 3 | 0.025 | 0.024691 | 0.288935 | 3.624817 | Kushner 2020 |
| 4 | 0.050265 | 0.002642 | 2.700293 | 6.22534 | Celzo 2020 (H) |
| 5 | 0.085546 | 0.002946 | 2.422059 | 6.164466 | Celzo 2020 (E) |
| 6 | 0.051546 | 0.005141 | 1.387601 | 5.757154 | Celzo 2020 (T) |
| 7 | 0.045455 | 0.00905 | 0.788328 | 5.151337 | Moro 2018 |
| 8 | 0.014388 | 0.007168 | 0.99522 | 5.426185 | Moro 2014 |
| 9 | 0.145833 | 0.020619 | 0.346008 | 3.927895 | Swamy |
| 10 | 0.042857 | 0.004751 | 1.501748 | 5.825658 | Baril 2015 |
| 11 | 0.006098 | 0.006079 | 1.173575 | 5.59918 | Segal 2011 |
| 12 | 0.125767 | 0.003063 | 2.329314 | 6.141261 | Wise |
| 13 | 0.026393 | 0.002928 | 2.436327 | 6.167894 | Perrett |
| 14 | 0.033727 | 0.001685 | 4.234144 | 6.425434 | Dana 2009 |
| 15 | 0.08125 | 0.006231 | 1.145038 | 5.574465 | Angelo 2014 (Act. S, all) |
| 16 | 0.037313 | 0.007435 | 0.959549 | 5.385483 | Shakib 2013 |
| 17 | 0.023077 | 0.001537 | 4.640793 | 6.457452 | Berenson 2016 |
| 18 | 0.011745 | 0.00014 | 51.027324 | 6.777092 | Morgan 2015 |

Fixed effects (inverse variance)

Pooled proportion = 0.015529 (95% CI = 0.013549 to 0.017643)

Non-combinability of studies

Cochran Q = 287.200276 (df = 17) P < 0.0001

Moment-based estimate of between studies variance = 0.028092

I_2_ (inconsistency) = 94.1% (95% CI = 92.4% to 95.2%)

Random effects (DerSimonian-Laird)

Pooled proportion = 0.041264 (95% CI = 0.025918 to 0.05998)

Bias indicators

Begg-Mazumdar: Kendall's -0.03268 P = 0.8228

Egger: bias = 2.807603 (95% CI = 1.580112 to 4.035095) P = 0.0002

Harbord: bias = 5.309312 (92.5% CI = 2.223336 to 8.395287) P = 0.0047

**Proportion meta-analysis Neonatal death animals**

Method: Stuart-Ord (inverse double arcsine square root)

| Stratum | Responding | Total |  |
| --- | --- | --- | --- |
| 1 | 8 | 326 | Wise |
| 2 | 0 | 15 | Warda 2021 |

| Stratum | Proportion | 95% CI (exact) | |  |
| --- | --- | --- | --- | --- |
| 1 | 0.02454 | 0.010653 | 0.047779 | Wise |
| 2 | 0 | 0 | 0.218019 | Warda 2021 [97.5% one-sided CI] |

| Stratum | Standardized effect | Variance | % Weights (fixed, random) | |  |
| --- | --- | --- | --- | --- | --- |
| 1 | 0.02454 | 0.003063 | 95.467836 | 95.467836 | Wise |
| 2 | 0 | 0.064516 | 4.532164 | 4.532164 | Warda 2021 [97.5% one-sided CI] |

Fixed effects (inverse variance)

Pooled proportion = 0.025462 (95% CI = 0.011461 to 0.044789)

Non-combinability of studies

Cochran Q = 0.074695 (df = 1) P = 0.7846

Moment-based estimate of between studies variance = 0

I_2_ (inconsistency) = 0% (95% CI = *% to *%)

Random effects (DerSimonian-Laird)

Pooled proportion = 0.025462 (95% CI = 0.011461 to 0.044789)

Bias indicators

Begg-Mazumdar: Kendall's <too few strata> P = *

Egger: bias = * (95% CI = * to *) P = *

Harbord: bias = -0.768115 (92.5% CI = * to *) P = *

**Proportion meta-analysis Neonatal death Both (humans and animals)**

Method: Stuart-Ord (inverse double arcsine square root)

| Stratum | Responding | Total |  |
| --- | --- | --- | --- |
| 1 | 17 | 3008 | Madhi 2020 |
| 2 | 0 | 22 | Munoz 2019 |
| 3 | 1 | 110 | Moro 2018 |
| 4 | 8 | 326 | Wise |
| 5 | 0 | 341 | Perrett |
| 6 | 1 | 593 | Dana 2009 |
| 7 | 0 | 15 | Warda 2021 |
| 8 | 2 | 6185 | Donegan 2014 |
| 9 | 2 | 7152 | Morgan 2015 |
| 10 | 2 | 793 | Petousis-Harris 2016 |

| Stratum | Proportion | 95% CI (exact) | |  |
| --- | --- | --- | --- | --- |
| 1 | 0.005652 | 0.003296 | 0.009033 | Madhi 2020 |
| 2 | 0 | 0 | 0.154373 | Munoz 2019 [97.5% one-sided CI] |
| 3 | 0.009091 | 0.00023 | 0.049611 | Moro 2018 |
| 4 | 0.02454 | 0.010653 | 0.047779 | Wise |
| 5 | 0 | 0 | 0.01076 | Perrett [97.5% one-sided CI] |
| 6 | 0.001686 | 0.000043 | 0.00936 | Dana 2009 |
| 7 | 0 | 0 | 0.218019 | Warda 2021 [97.5% one-sided CI] |
| 8 | 0.000323 | 0.000039 | 0.001168 | Donegan 2014 |
| 9 | 0.00028 | 0.000034 | 0.00101 | Morgan 2015 |
| 10 | 0.002522 | 0.000306 | 0.009081 | Petousis-Harris 2016 |

| Stratum | Standardized effect | Variance | % Weights (fixed, random) | |  |
| --- | --- | --- | --- | --- | --- |
| 1 | 0.005652 | 0.000332 | 16.218329 | 15.686456 | Madhi 2020 |
| 2 | 0 | 0.044444 | 0.121294 | 1.377134 | Munoz 2019 [97.5% one-sided CI] |
| 3 | 0.009091 | 0.00905 | 0.595687 | 5.137389 | Moro 2018 |
| 4 | 0.02454 | 0.003063 | 1.760108 | 9.546586 | Wise |
| 5 | 0 | 0.002928 | 1.84097 | 9.734315 | Perrett [97.5% one-sided CI] |
| 6 | 0.001686 | 0.001685 | 3.199461 | 11.896388 | Dana 2009 |
| 7 | 0 | 0.064516 | 0.083558 | 0.973192 | Warda 2021 [97.5% one-sided CI] |
| 8 | 0.000323 | 0.000162 | 33.345013 | 16.343703 | Donegan 2014 |
| 9 | 0.00028 | 0.00014 | 38.557951 | 16.431847 | Morgan 2015 |
| 10 | 0.002522 | 0.00126 | 4.277628 | 12.87299 | Petousis-Harris 2016 |

Fixed effects (inverse variance)

Pooled proportion = 0.001187 (95% CI = 0.000743 to 0.001734)

Non-combinability of studies

Cochran Q = 60.557244 (df = 9) P < 0.0001

Moment-based estimate of between studies variance = 0.003913

I_2_ (inconsistency) = 85.1% (95% CI = 73.6% to 90.3%)

Random effects (DerSimonian-Laird)

Pooled proportion = 0.003366 (95% CI = 0.001073 to 0.006929)

Bias indicators

Begg-Mazumdar: Kendall's 0.111111 P = 0.7275

Egger: bias = 1.060812 (95% CI = -0.093671 to 2.215296) P = 0.0669

Harbord: bias = 2.923092 (92.5% CI = -1.71061 to 7.556793) P = 0.2329
